# Highly Diastereoselective Construction of Carbon– Heteroatom Quaternary Stereogenic Centers in the Synthesis of Analogs of Bioactive Compounds: From Monofluorinated Epoxyalkylphosphonates to α-Fluoro-, β-, or γ-Amino Alcohol Derivatives of Alkylphosphonates

**DOI:** 10.3389/fchem.2021.613633

**Published:** 2021-06-02

**Authors:** Magdalena Rapp, Klaudia Margas-Musielak, Patrycja Kaczmarek, Agnieszka Witkowska, Tomasz Cytlak, Tomasz Siodła, Henryk Koroniak

**Affiliations:** ^1^Faculty of Chemistry, Adam Mickiewicz University in Poznań, Poznań, Poland; ^2^Centre for Advanced Technologies Adam Mickiewicz University in Poznań, Poznań, Poland

**Keywords:** diastereoselectivity, quaternary stereogenic center, aminophosphonates, fluorinated phosphonates, aminoalcohols, epoxyalkylphosphonates

## Abstract

The synthesis of the stable surrogates of an important amino acid (*R*)-4-amino-3-hydroxybutyric acid (GABOB) such as substituted hydroxy aminophosphonic acids bearing a quaternary stereogenic center is presented. Highly diastereoselective formations of fluorinated spiroepoxy alkylphosphonate or related tertiary carbon-containing oxiranes from β-keto phosphonates possessing methyl, phenyl, or cyclohexenyl substituents, are reported. Stereoselective acid-promoted epoxide opening by bromide or azide followed by reduction/protection afforded tertiary bromides or *N*-Boc derivatives of β-amino-γ-hydroxy alkylphosphonates in most cases, while the reactions of oxiranes with different amines yielded their β-hydroxy-γ-amino regioisomers. Surprisingly, during the synthesis of amino phosphonic acids, we observe that the acid-induced rearrangement proceeded in a high diastereospecific manner, leading finally to substituted β-hydroxy-γ-aminoalkylphosphonic acids.

**Graphical Abstract:**
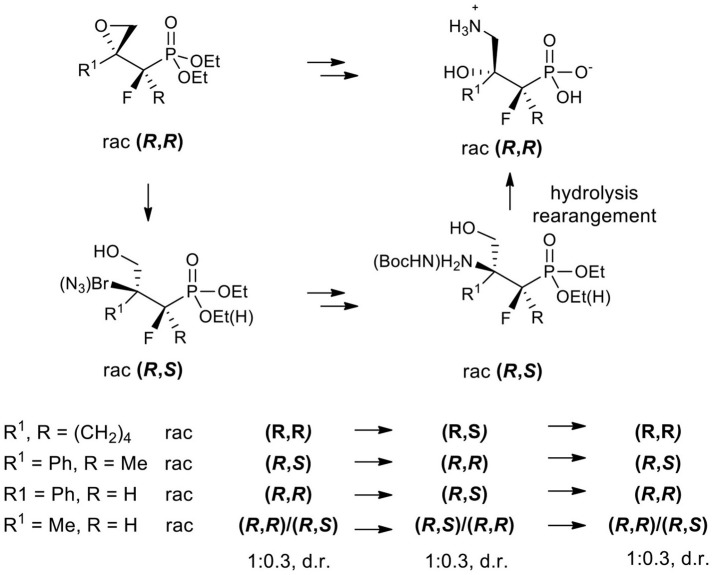
Diastereoselective transformations of fluorinated epoxyalkylphosphonates.

## Introduction

**Figure 1:**
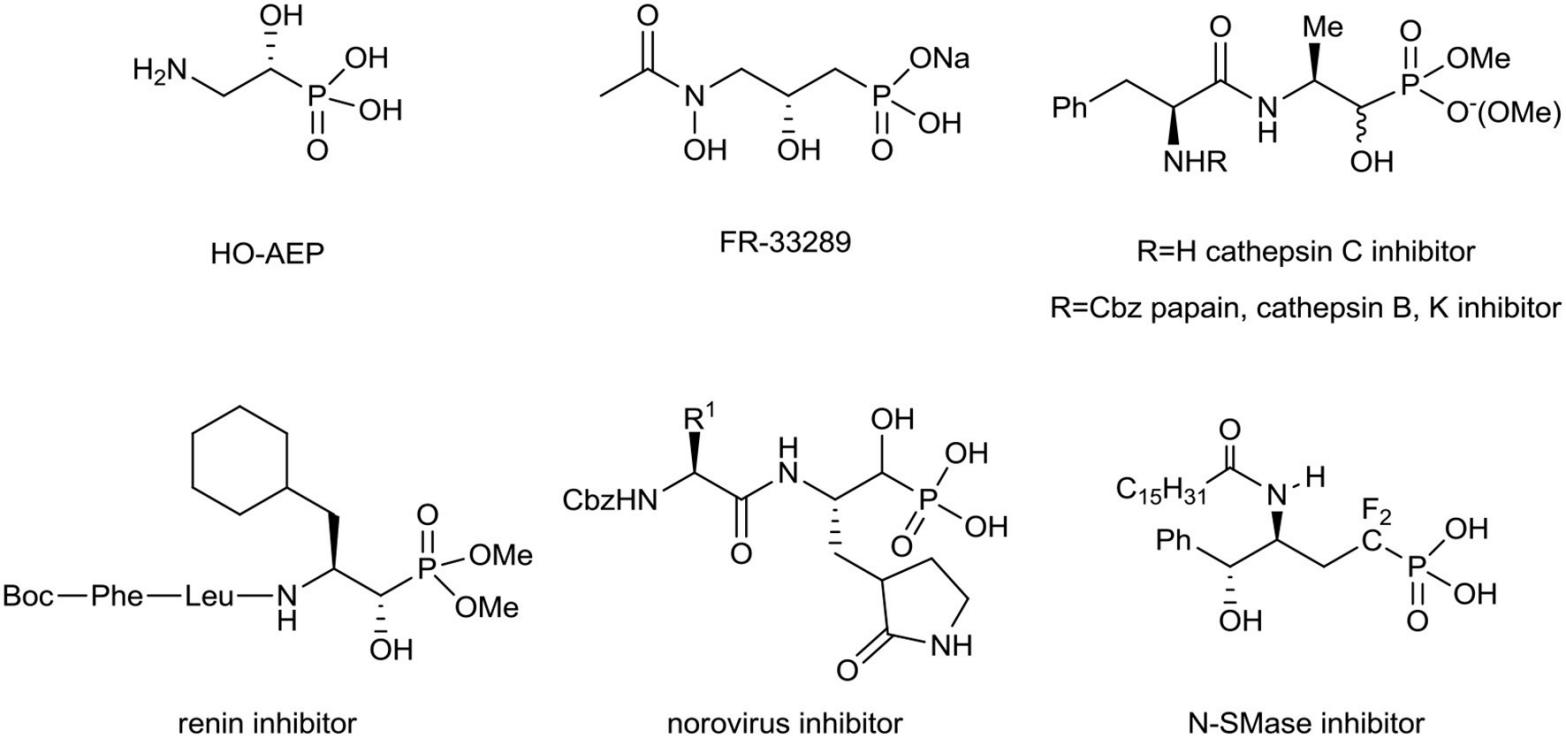
Structures of different bioactive amino alcohol derivatives of alkylphosphonates.

Aminophosphonates can be considered as good amino acid surrogates when the tetrahedral phosphonic acid moiety corresponds to the planar carboxylic group (Kukhar and Hudson, 2000; Kafarski and Lejczak, 2001; Palacios et al., 2005; Ordóñez et al., 2009, 2015; Naydenova et al., 2010; Orsini et al., 2010; Kafarski, 2020). The C–P bond is stable in different biochemical and thermal conditions and resists photochemical decomposition (Fields, 1999; Horsman and Zechel, 2017). For these reasons, in medicinal as well as in organic chemistry the synthesis and applications of α-, β-, or γ-amino phosphonates are of special interest (Kafarski and Lejczak, 1991; Fields, 1999; Foss et al., 2007; Mucha et al., 2011; Bera et al., 2013; Gu et al., 2018; Kafarski, 2020). Among them, phosphorus-containing amino alcohols frequently exhibit various biological interactions (Patel et al., 1990; Drag et al., 2013; Mandadapu et al., 2013). As a representative example (*R*)-2-amino-1-hydroxyethylphosphonic acid (HO-AEP), has been recognized as a component of the protozoal plasma membrane as well as in marine invertebrates and microorganisms (Korn et al., 1973; Watanabe et al., 2001), while FR-33289 with antibiotic properties was first isolated from *Streptomyces rubellomurinus* subsp., *indigoferus* (Iguchi et al., 1980). To compare, the obtained various 2-amino-1-hydroxy-alkanephosphonate dipeptides have been found as potent, non-covalent organophosphonate inhibitors of cathepsin C, papain, cathepsin B, and cathepsin K (Drag et al., 2013), renin (Patel et al., 1990), or norovirus (Mandadapu et al., 2013). Furthermore, the α-fluorinated δ-hydroxy-γ-aminophosphonates have been applied as the inhibitors of neutral Sphingomyelinase (N-SMase) (Yokomatsu et al., 2003; Figure 1).

**Figure 2:**
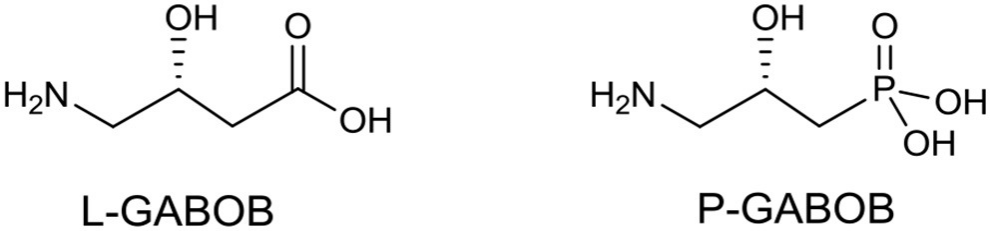
Structures of (*R*)-β-hydroxy-γ-aminobutanoic acid (L-GABOB) and its organophosphorus analogs P-GABOB.

In medicinal chemistry, the introduction of a phosphonate group to biologically significant compounds is a frequently applied method to modify its physicochemical properties that could play an important role in a biological environment. The group of biologically important amino alcohols includes the attractive target 4-amino-3-hydroxybutyric acid (GABOB). (*R*)-(–)-γ-Amino-β-hydroxybutanoic acid (L-GABOB), also known as (*R*)-(–)-β-hydroxy-γ-aminobutanoic acid, is an important amino acid acting as an agonist of neurotransmitter γ-aminobutyric acid (GABA) (Roberts et al., 1981; Falch et al., 1986; Kristiansen and Fjalland, 1991). It is used in numerous illness treatments including epilepsy therapy (De Maio and Pasquariello, 1963; Banfi et al., 1983; García-Flores and Farías, 1997; Melis et al., 2014). Among several GABA and GABOB analogs the γ-amino-β-hydroxy phosphonic acid (P-GABOB) and its organophosphorus derivatives constitute an interesting group of compounds (Dingwall, 1983; Yuan et al., 2002; Ordóñez et al., 2003, 2010; Wang et al., 2003; Wróblewski and Hałajewska-Wosik, 2003; Tadeusiak, 2004; Figure 2).

Their syntheses have been successfully accomplished by the transformation of chiral precursors (Ordóñez et al., 2003; Wróblewski and Hałajewska-Wosik, 2003; Tadeusiak, 2004), the application of racemic starting materials (Dingwall, 1983; Ordóñez et al., 2003), and kinetic resolution (Wang et al., 2003; Wróblewski and Hałajewska-Wosik, 2003) or biocatalysis (Tadeusiak, 2004). Some GABA analogs are known as GABA receptor agonists and are used as drugs, e.g., β-phenyl-γ-aminobutanoic acid (phenibut) (Lapin, 2001), β-(4-chlorophenyl)-γ-aminobutyric acid (baclofen) (Brogden et al., 1974; Leggio et al., 2010), or 1-(aminomethyl)cyclohexane acetic acid (gabapentin) (Goa and Sorkin, 1993; Smith et al., 2016). On the other hand, two enantiomers of monofluorinated analogs of GABOB (with a fluorine instead of hydroxyl group) have been synthesized in order to determine the conformation of GABA when binding to specific protein receptors (Clift et al., 2007; Deniau et al., 2007; Yamamoto et al., 2011). In these types of compounds, due to a charge-dipole interaction between fluorine and a charged nitrogen atom, the *gauche* alignment F-C-C-N^+^ is preferred (Briggs et al., 2004). These properties revealed the distinct structural features of GABA binding sites to GABA_A_ synopsis receptors comparing them to GABA-aminotransferase which may be of importance in Alzheimer's and Parkinson's diseases treatment (Clift et al., 2007; Deniau et al., 2007; Yamamoto et al., 2011). Analogously, in protonated vicinal fluorohydrine the *gauche* conformers are strongly favored lowering its energy (Clift et al., 2007; Deniau et al., 2007; Yamamoto et al., 2011). This effect has reinforced or destabilized chain conformations influencing binding affinities as it was reported for the fluorinated analogs of Indinavir (HIV protease inhibitor) (Myers et al., 2001). Other electronic and steric impacts mirroring an enzyme-substrate interaction have already been applied in medicinal chemistry (O'Hagan and Rzepa, 1997; Bégué and Bonnet-Delpon, 2008). What is more, electronegative fluorine increases the acidity of neighboring carboxylic acid (Koppel et al., 1994) or phosphonic acid (O'Hagan and Rzepa, 1997), and in the same manner, lowers the basicity of amines (Abraham et al., 1990), inducing a remarkable effect upon the physical and biochemical properties of fluorine-containing molecules. Similarly, the introduction of a phosphonate group to organic compounds modifies its physicochemical properties and could play an important role in the biological environment.

On the basis of a combination of reactivity and synthetic application of oxiranes in organic synthesis (Parker and Isaacs, 1959; Ready and Jacobsen, 2002; Azoulay et al., 2005; Wu and Xia, 2005; Padwaa and Murphree, 2006; Fustero et al., 2011; Singh et al., 2013; Zhao and Weix, 2014; Faiz and Zahoor, 2016), we decided to apply those three-membered heterocycles toward aminophosphonates. Recently we developed the method for the synthesis of two types of monofluorinated α, β-epoxyphosphonates with the vicinal and geminal arrangement of fluorine and phosphorus atoms via a Michaelis-Becker addition or by an intramolecular ring closure reaction (Rapp et al., 2015). Herein, we report our results concerning the diastereoselective synthesis and application of fluorinated epoxyalkylphosphonates toward α-fluoro γ-amino-β-hydroxybutanoic acid (GABOB) as well as β-amino-γ-hydroxyalkylphosphonic acid analogs. We expect that the interaction between fluorine and charged nitrogen or oxygen (*gauche* effect) in α-fluoro β,γ-amino alcohol derivatives of alkylphosphonates will play an important role and may be used in the future to reveal different binding sites when inhibiting certain enzymes.

## Results and Discussion

The first aim of our study was the synthesis of α-fluoro-β,γ-epoxy alkylphosphonates as valuable intermediates for the construction of tertiary hydroxy- or aminophosphonates. We started from the convenient racemic diethyl α-fluoro-β-ketophosphonates bearing methyl, phenyl, or cyclohexyl substituents (compounds **1–4**). The oxiranes **5–8** were received by the treatment of corresponding substrates with diazomethane. In the case of **1**, the reaction led to the formation of oxiranes: **5** (93%, 3.3:1 d.r.), while from ketones **2–4** the epoxides **6–8** were obtained (yields from 90 to 95%) (Table 1).

**Table 1:**
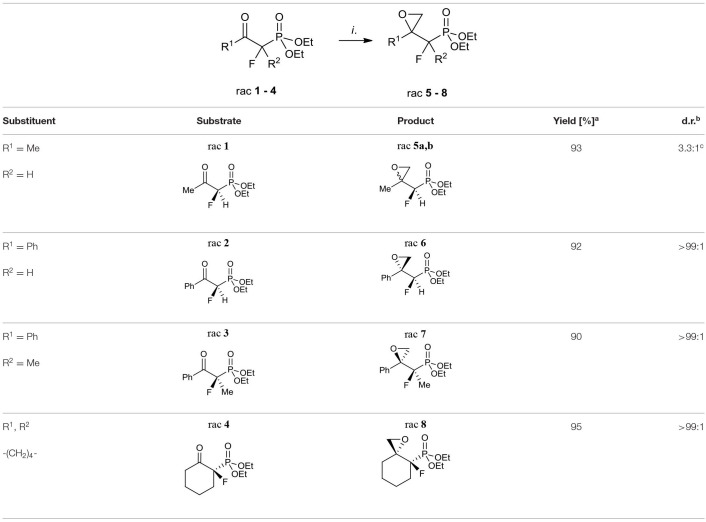
Synthesis of fluorinated epoxy alkylphosphonates **5–8**.

*i. CH_2_N_2_, 3 eq, Et_2_O, rt, 48 h.*

*^a^Isolated yield.*

*^b^Crude reaction mixture (^19^F NMR and ^31^P NMR).*

*^c^Configuration (1R, 2R)/(1R, 2S)*.

**Figure 3:**
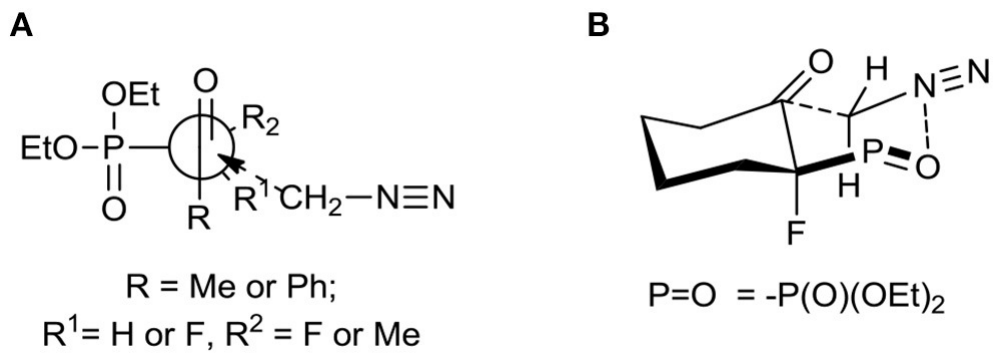
**(A)** Unhindered attack of CH_2_N_2_ to β-ketophosphonates **1**–**3** during formation of **5–7**; **(B)** Proposed transition state leading to **8**.

The diastereoselective addition of CH_2_N_2_ to acyclic β-ketophosphonate occurred contrary to the Felkin-Anh model with the bulky diethylphosphonate moiety as the biggest substituent perpendicular to C=O, with the medium–sized group R^2^ (e.g., F for **1, 2**, and Me in the case of **3**) and smallest substituents R^1^ (e.g., H for **1, 2**, and F for **3**), respectively (Sano et al., 2006) leading to **5a** and **6–7** and could be explained by the addition to the carbonyl group as presented in Figure 3A. Thus, the steric interactions between the bulky diethylphosphonate moiety (perpendicular), fluorine, and other substituents and the approaching nucleophile are minimized. On the other hand, the equatorial attack and coordination of diazomethane with phosphonyl oxygen, in the six-membered chair-like transition state, led to compound **8** (Figure 3B). We also observed the influence of ketone substituents on diastereoselectivity. Thus, in the case of the addition to **2–4** [R^1^ = Ph or -(CH_2_)_4_-] the diastereomerically pure oxiranes **6–8** (>99:1 d.r. as determined by NMR) were formed, while the reaction of **1** (R^1^ = CH_3_, R^2^ = H) with diazomethane gave two diastereoisomers of **5** (**5a:5b**, 3.3:1, d.r.).

**Scheme 1:**
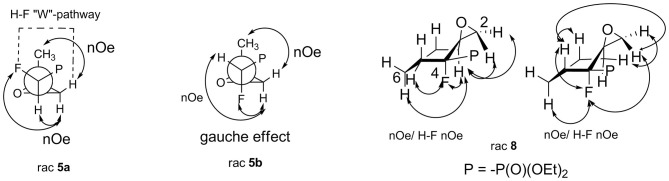
Nuclear Overhauser effects (nOe effects) and diagnostic coupling constants in oxiranes **5a,b** and **8**.

The assignment of the relative configuration of spiroepoxide or related tertiary carbon-containing oxiranes can be troublesome and difficult to achieve. However, the structure and stereochemistry of compounds **5–8** were confirmed by NMR spectra, analysis of nOe effect between H-H (1D or 2D NOESY), and H-F correlations (2D or 1D HOESY). Thus, the 2D NOESY and 2D H-F HOESY experiments registered for both diastereoisomers **5a** and **5b**, indicated correlations between one of the diastereotopic protons of the oxirane ring and proton derived from the CHFP moiety as well as with fluorine. At the same time, the nOe effects between the remaining oxirane proton and methyl group as well as its long-range interactions “W-pathway” with fluorine (^4^*J*_FH_ 5.6/5.8 Hz, in the case of major **5a**) (Dolbier, 2009) or phosphorous atoms (^4^*J*_HP_ 2.1 Hz, for minor **5b**) (Zymańczyk-Duda et al., 1995) indicated stereochemistry rac (1*R*, 2*R*) for major **5a** and rac (1*R*, 2*S*) in the case of minor **5b**. At the same time, the correlation of signals derived from fluorine and the methyl group for **5a** compared to the weak nOe effect for F-Me in the case of **5b** additionally confirm the assigned configurations (Scheme 1). Additionally, the slightly different enhancements in nOe effects for the C*HF* proton and fluorine with phenyl as well as one of the protons of the oxirane ring were detected in the NOESY and HOESY spectra of **6**. The second epoxide proton was involved in the “W-pathway” (^4^*J*_FH_ 5.6Hz). In the case of **7**, similar interactions between the methyl group and fluorine (2D NOESY and 2D H-F HOESY) together with one of the oxirane protons were observed. These analyses in comparison with spectral properties of **5a, b** allow us to assign the configuration for **6** as rac (1*R*, 2*R*) and **7** as rac (1*R*, 2*S*). The analysis of the nOe effects (2D NOESY, H-F HOESY) for spiroepoxide **8** revealed the correlations of epoxide protons C2*H*H with axial proton C5H and equatorial C7H of cyclohexane, while the nOe effects between fluorine and the closer proton of oxirane as well as the contacts between fluorine and the axial protons of the cyclohexane ring (C6H and C8H), as presented in Scheme 1, indicated configuration (3*R*, 4*R*) of **8**, and were the most informative in the stereochemical assignment. Additionally in the ^13^C NMR spectra of **5–8**, the typical *C*FP(O)(OEt)_2_ chemical shifts and values of ^1^*J*_C−P_ are from 166 to 172 Hz, while ^1^*J*_C−F_ varying from 185 to 191 Hz, according to literature, were observed (Dolbier, 2009). Interestingly, the coupling of fluorine and phosphorus to the carbon atom is readily observable in ^13^C NMR and allowed us to distinguish the proximity of each carbon atom to these heteroatoms (Gorenstein, 1984; Hesse et al., 1997; Dolbier, 2009). At the same time, values of coupling constants ^2^*J*_C−P_ were <9 Hz while analogous ^2^*J*_C−F_ appeared usually around 20 Hz (with an exception of 12 Hz for **6**). Moreover, ^3^*J*_C−F_ and ^3^*J*_C−P_ were c.a. 6–8 Hz and 2–3 Hz for **5a** and **6–7** while for cyclohexane **8** ^3^*J* was 7–10 and 2 Hz, respectively. The spectral properties of the obtained compounds (IR, ^1^H NMR) as well as chemical shift values in the ^13^C NMR spectra are consistent with the data reported for epoxyalkylphosphonates (Griffin and Kundu, 1969; Wróblewski and Bak-Sypień, 2007) as well as with spiroepoxides (Zanardi et al., 2016).

The oxiranes **5a and 5b** exist in [D] chloroform predominantly as their *gauche* conformers (respectively to the C1-C2 bond) as can be judged from the analysis of nOe interactions as well as W-pathway effects. This conclusion was further supported by the comparison of relative energies calculated for each conformers of **5a, b** (Tables 2, 3). In the case of compound **5a**, the DFT conformation analysis showed the preference of conformation **1** (Table 2—conformations **1Y** and **1Z** have the lowest energies). We also observed the influence of geometries of the bulky diethoxyphosphoryl group and the relative energies of calculated conformers. In the case of compound **5b**, the conformation analysis suggested that the most stable were two conformers **2Y** and **3X** (Table 3), while from the NMR spectra conformation **3** seemed to be the major one. Such an inconsistency may be related to the fact that DFT calculations were performed “in vacuum” and do not reflect any interactions with solvent.

**Table 2:**
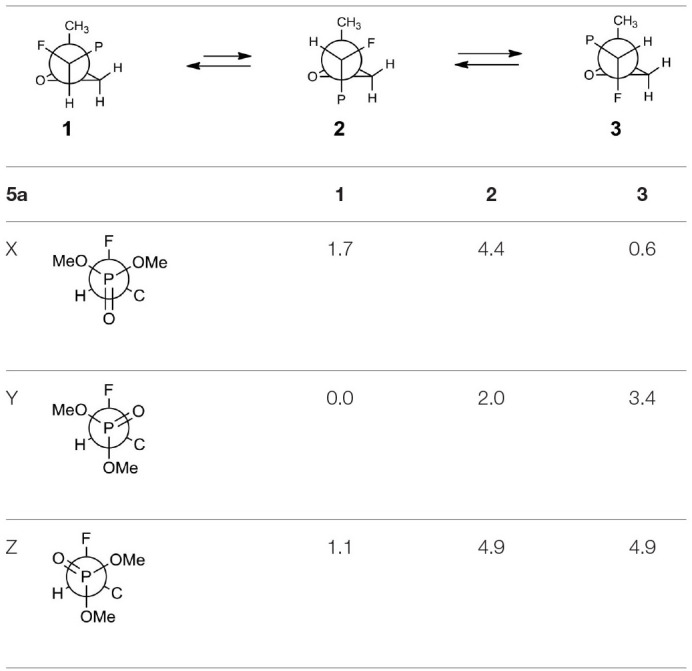
The geometries and relative energies [kcal/mol] of nine energy minima for the compound **5a**.

*The structure has been resected to simplify the conformation analysis (there are methoxyl instead of ethoxyl groups)*.

**Table 3:**
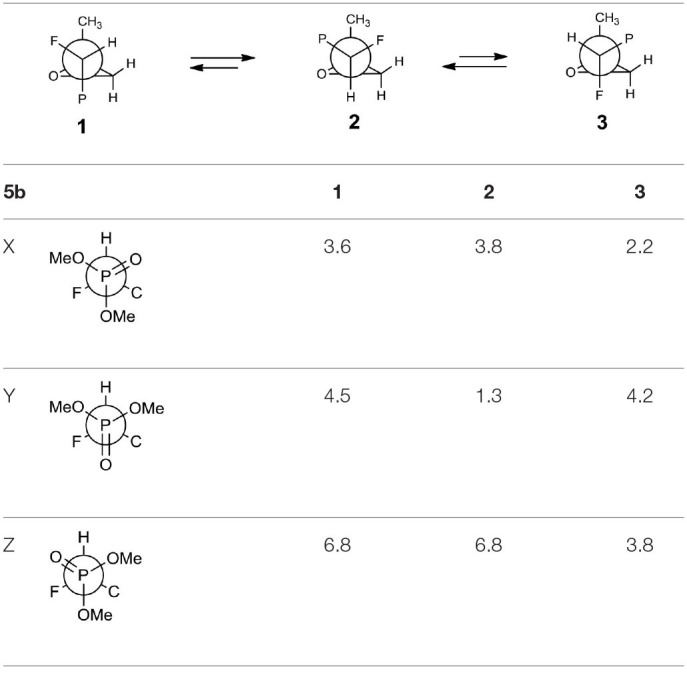
The geometries and relative energies [kcal/mol] of nine energy minima for the compound **5b**.

*The structure has been resected to simplify the conformation analysis (there are methoxyl instead of ethoxyl groups)*.

Stereoselective addition of a nucleophile to α-fluorophosphonoacetates approaching opposite to the phosphonates trajectory was already reported and applied in tandem reduction-olefination (Sano et al., 2006). Moreover, the similar diastereoselectivity of the diazomethane addition and its coordination in the case of the reaction of chiral α-alkyl/aryl substituted β-keto (*Rs*)-sulfoxides were presented by Sorochinsky and Soloshonok (2010). Also, the addition of the Grignard reagent to (2-oxocyclohexyl)phosphonate resulted in a hydroxy phosphonate with a cyclohexane ring possessing both OH and phosphonate groups in *cis* geometry as the major isomer [*cis*/*trans*, 95:5, respectively] (Lentsch and Wiemer, 1999). To compare, the additions of diazomethane or ethyl(iodomethyl)zinc to non-fluorinated β-ketophosphonates such as 2-oxo-2-phenylethyl- or 2-oxopropyl phosphonates were already reported. Thus, the chain extensions yielding γ-ketophosphonates were observed (Arbuzov et al., 1963; Verbicky and Zercher, 2000).

Considering the existing literature on the different reactions of epoxides, we decided to use them as building blocks in the synthesis of fluorinated β,γ-amino alcohol derivatives of alkylphosphonates. The first approach involves the application of amines to the epoxide ring opening with further transformations. The second strategy is based on the use of azides followed by the reduction and phosphonates hydrolysis. The results concerning the applied reaction in the case of compounds **5–8** with different amines are presented in Table 4.

**Table 4:**
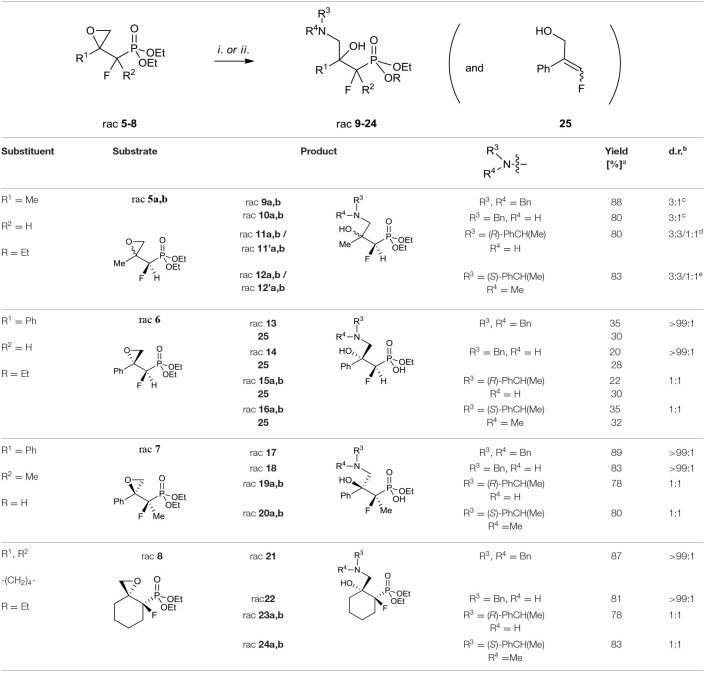
Synthesis of γ-amino-β-hydroxy alkylphosphonates **9–24** and **25**.

*i. or ii. Primary or secondary amine, TEA, 60°C, 24 or 48 h (see: Experimental part).*

*^a^Isolated yield.*

*^b^Crude reaction mixture (^19^F NMR and ^31^P NMR).*

*^c^Configuration (1R, 2R)/(1R, 2S).*

*^d^Configuration (S, 1R, 2R):(S, 1R, 2S)/(S, 1S, 2S):(S, 1S, 2R).*

*^e^Configuration (R, 1R, 2R):(R, 1R, 2S)/(R, 1S, 2S):(R, 1S, 2R)*.

**Scheme 2:**
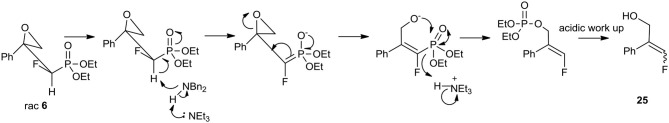
Mechanism of transformation of **6** to **25** during reaction with Bn_2_NH and TEA.

The use of secondary amines [dibenzylamine (Bn_2_NH), *N*,α-(*R*)-dimethylbenzylamine (*R*)-PhCH(Me)NH(Me)] or primary amines [benzylamine (BnNH_2_), or (*S*)-methylbenzylamine [(*S*)-PhCH(Me)NH_2_] with the addition of triethylamine (TEA) led to oxirane opening from the unhindered side to give one regioisomer of γ-amino-β-alcohols **9**–**24**. The applications of secondary amines gave products with higher yields than with primary amines. Moreover, the reactions with chiral (*S*)-methylbenzylamine and *N*,α-(*R*)-dimethylbenzylamine gave **11a, b/11'a, b** or **12a, b/12'a, b**, respectively, as a mixture of diastereoisomers [3:3/1:1, d.r.]. To compare, we observed the formation of two stereoisomers **15–16** and **19–20**, and **23–24** [1:1, d.r.] in the case of reaction with **6–8**, as judged by NMR (Table 4). However, to open oxirane **7** with amines, an extended reaction time was necessary, yielding phosphonate monoesters **17–20**. Base-mediated hydrolysis of dialkyl phosphonates to their monoalkyl phosphonic acids salts was already reported (Westheimer et al., 1988). Usually this reaction requires drastic experimental conditions (high pH and prolongated heating time). To compare, in the case of the reaction of oxirane **6** with amine, the mixtures of unreacted **6**, appropriate amino alcohols **13–16** and **25** with poor yields were obtained. The addition of Bn_2_NH to epoxide **6** was studied in detail by ^19^F and ^31^P NMR. Thus, reaction of **6** with one equivalent of Bn_2_NH led to **13** with low yields (18%), while 35% yield was obtained when applying five equiv. of dibenzylamine and TEA (5 equiv.). During the experiment we observed formation of **25** (McDonald et al., 1985; Huleatt et al., 2015) as a mixture of isomers *E/Z*: 28:1 (Table 4). Apparently, due to acidic α-proton abstraction (by amide formed from primary or secondary amine and TEA-both amines are necessary), followed by E1cb elimination leading to epoxide-ring opening with subsequent intramolecular phosphonate rearrangement, the phosphate was formed. This compound during acidic work-up transformed into allylic alcohols **25** (Scheme 2).

The E1cb reactions occurring with *syn* or *anti* orientation were already applied in the stereoselective olefin syntheses (Clayden et al., 2008). Moreover, the saponification and elimination of a mixtures of *syn* and *anti* diastereoisomers (1:1) of β-hydroksyphosphonates led to the *E*/*Z* mixture of olefins (1:1) or vinyl phosphonates, while application of pure *anti* isomer caused formation of *Z* stereoisomer via oxaphosphetane, exclusively (Reichwein and Pagenkopf, 2003). Also, the hydrolysis of the C–P bond under basic conditions yielding phosphonate/phosphate conversion has been already reported (McKenna and Shen, 1981; Piettre and Cabanas, 1996; Beier et al., 2008).

Taking into account the low yields of amino alcohols **13–16**, difficulties in the purification of the decomposing mixture of **6**, **25**, and amino alcohols formed from **6** (^19^F NMR, ^31^P NMR) the use of the phenyl series toward GABOB analogs synthesis was abandoned.

**Figure 4:**
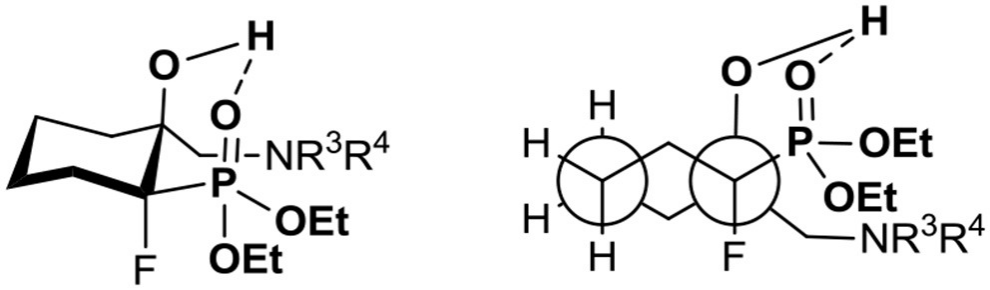
The proposed predominant conformation of γ-amino-β-hydroxyphosphonates **21–24**.

The analysis of the NMR spectra of **9–24** confirmed the formation of aminohydroxyphosphonates possessing the tertiary hydroxyl group. Thus, the signals of the tertiary carbon atom (*C*-OH) were located around δ: 72–78 (^2^*J*_C−F_ 19–21 Hz) while signals of *C*H_2_NR^3^R^4^ appeared at δ: 53–59 similarly to tertiary alcohols (Duangdee et al., 2012) and *N*-benzyl or trityl protected amines (Verbruggen et al., 1996; Wróblewski and Hałajewska-Wosik, 2003). The stereochemistry of the obtained amino alcohol was a consequence of configuration in starting epoxides and was distinguished by spectroscopic data analysis. The examination of the ^1^H NMR spectra combined with 2D NOESY and 1D H-F HOESY correlations for amino alcohols **9a, b** confirmed the configurations as rac (1*R*, 2*R*/1*R*, 2*S*). Thus, the nOe interactions between the proton as well as fluorine (C*HF*P) and both oxirane protons in major **9a** were observed, while the different enhancements of nOe effects for the C*HF* proton and fluorine and analogous epoxide protons in the case of minor **9b** were noticeable. By analogy, the stereochemistry of one diastereoisomer of **17** as rac (1*R*, 2*S*) and tentatively as rac (1*R*, 2*R*) for **13** has been assigned. The analysis of nOe effects (2D NOESY, 1D H-F HOESY) for **21** showed, similar to those found on the spectra of **8**, the correlations of the methylene (C*H*_2_OH) protons with both protons C3H (slightly different nOe effect) of cyclohexane, while the nOe effects between fluorine and both C*H*_2_OH protons as well as the contacts between fluorine and axial protons of the cyclohexane ring (C3H and C5H) and vicinal C6 protons indicated configuration (3*R*,4*R*) of **21**, in the mixture with the predominant conformation. The 2-hydroxyalkylphosphonates are known to exist in CDCl_3_ in major “frozen” conformation where intramolecular hydrogen bonding between hydroxyl hydrogen and phosphonate oxygen allows them to form a six-membered ring in chair conformation and increase its stability (Zymańczyk-Duda et al., 1995; Genov et al., 1998; Gancarz et al., 2000; Wróblewski and Hałajewska-Wosik, 2002). In the case of cyclohexane derivatives, amino alcohols **21–24** can adopt a conformation similar to *trans*-decalin as presented in Figure 4.

The proposed predominant conformation of compounds **21–24** based on the values of ^3^*J*_C−P_ 3–5 Hz in a signal derived from the CH_2_N group in the ^13^C NMR spectra indicate *gauche* conformation (regarding phosphonate moiety, P-Cγ). To compare, the other Cγ carbon atoms of the cyclohexane ring are in *anti*-arrangement as can be demonstrated by ^3^*J*_C−P_ 8–9 Hz visible in signals appearing around δ: 32–35 and 19–20 ppm (Wróblewski and Hałajewska-Wosik, 2002).

The obtained regiochemistry is in agreement with the stereochemical outcome of the ring opening of other terminal epoxides at the least hindered position (Parker and Isaacs, 1959; Azoulay et al., 2005; Wu and Xia, 2005; Padwaa and Murphree, 2006; Fustero et al., 2011; Singh et al., 2013). The analogous reaction of optically active (*S*)-2,3-epoxypropylphosphonate with Bn_2_NH almost exclusively gave one (*S*)-enantiomer of amino alcohol (Wróblewski and Hałajewska-Wosik, 2002). Similar reactions of 2,3-oxiranepropylphosphonates with *N*-tritylamine as well as *N*-benzhydrylamine were already applied for organophosphorus analog of (*R*)-4-amino-3-hydroxybutyric acid (L-GABOB) synthesis (Wróblewski and Hałajewska-Wosik, 2003). Moreover, the reaction of dimenthyl-(*S*)-2,3-oxiranopropylophosphonate with dibenzylamine serve for the synthesis of the non-fluorinated analog of P-GABOB (Nesterov and Kolodiazhnyi, 2007). An alternative method of oxirane **6** opening with aqueous ammonia as conveniently applied in the case of the reaction with epoxy vinyl phosphonate (Cristau et al., 2000) gave a complicated mixture of products decomposing during column chromatography.

Next, the synthesis of γ-amino-β-hydroxy alkylphosphonic acids was performed. Thus, the hydrogenation with concomitant introduction of the Boc group (Pd//C, Boc_2_O, Table 5, method *i*.) of dibenzyl derivatives **9**, **17**, and **21** were applied resulting in the *N*-Boc (*N*-*tert*-butoxycarbonyl)-protected phosphonates **26** and **28**. This step, necessary to avoid decomposition of the amino alcohol, was already used in the synthesis of organophosphorus analogs of GABOB (Wróblewski and Hałajewska-Wosik, 2003). On the other hand, due to the presence of one free acidic group (R = H), the debenzylation of **17** to get **27** required only hydrogen and a palladium catalyst as reagents (Table 5, method *ii*.). The examination of the ^1^H NMR spectra combined with 1D NOE difference spectrometry and the ^1^D H-F HOESY spectra for amino alcohols **27** indicated the nOe interactions between methyl protons and one of the diastereotopic epoxide protons at the same time without nOe effect with a phenyl group, while in the case of fluorine spectra (1D HOESY) the interactions for both oxirane rings were detected. Taking into account the predominant “frozen” conformation where intramolecular hydrogen bonding between hydroxyl hydrogen and phosphonate oxygen were formed, the configuration of **27** as rac (2*R*, 3*S*) was assigned, additionally confirming the stereochemistry of starting oxirane **7**. In the last step, acidic hydrolysis of **26–28** (conc HCl, reflux, 2–3 days) followed by propylene oxide treatment (Wróblewski and Hałajewska-Wosik, 2003; Table 5, method *iii*.) gave target compounds as salts **29–31**.

**Table 5:**
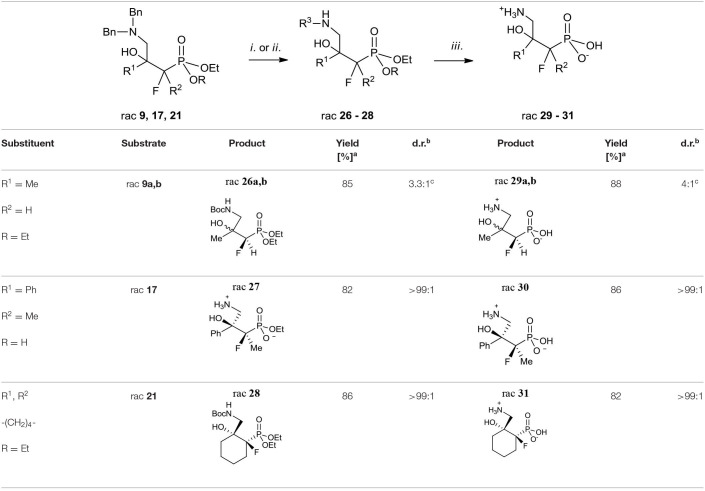
Synthesis of fluorinated γ-amino-β-hydroxy alkylphosphonic acids **29–31**.

*i. H_2_, Pd//C, Boc_2_O, EtOH; ii. H_2_, Pd//C, EtOH; iii. conc. HCl, reflux, 2d, propylene oxide, EtOH.*

*^a^Isolated yield.*

*^b^Crude reaction mixture (^19^F NMR and ^31^P NMR).*

*^c^Configuration (1R, 2R)/ (1R, 2S)*.

In the case of substrate **26a, b**, two diastereoisomers of **29a, b** (4:1, d.r.) were formed, while the reaction of **27**–**28** gave the single diastereoisomers of **30–31** (>99:1, d.r.). The configurations in the obtained γ-amino-β-alcohols **29–31** resulted from the stereochemistry of starting amino alcohols and as rac (1*R*, 2*R*) for major **29a**, as rac (1*R*, 2*S*) for minor **29b**, and as rac (2*R*, 3*S*) for **30** were assigned (Table 5). Additionally, the analysis of one- and two-dimensional NMR spectra allowed us to confirm the structure of **31** as rac (1*R*, 2*R*). Importantly, in the acidic conditions, we did not observe rearrangement or racemization, but we noticed that compounds were contaminated with elimination products as can be judged by NMR.

Our strategy for the synthesis of β-amino-γ-hydroxy phosphonates relied on the construction of tertiary amines via bromides or azides.

Considering oxiranes as useful intermediates for the syntheses of halogenohydrines, we decided to react obtained epoxides **5–8** with hydrogen bromide (generated from AcBr and MeOH). The reactions led to vicinal bromohydrines **32–35** with yields from 86% for **33** to 94% for **34** (method *i*. Table 6).

**Table 6:**
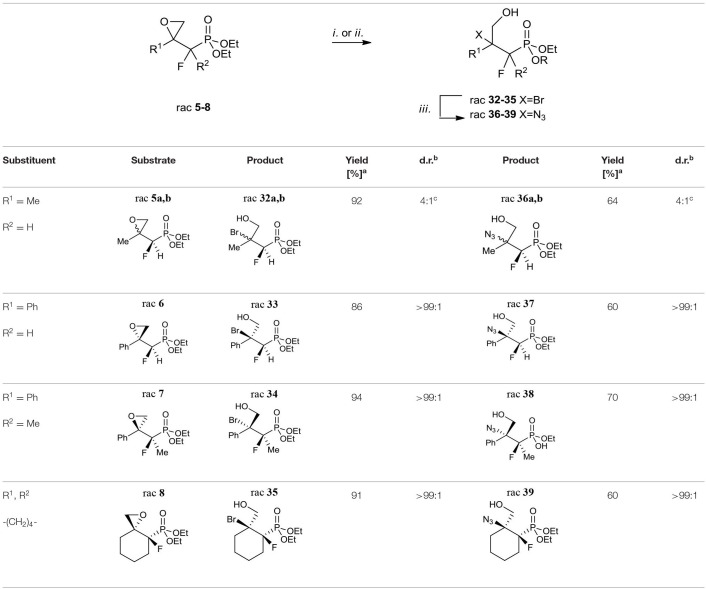
Synthesis of fluorinated β-bromo- or β-azido-γ-hydroxy- alkylphosphonates **32–35** or **36–39**.

*i. AcBr, MeOH, 0°C, 2 h; ii. NaN_3_, (NH_4_)_2_SO_4_, EtOH/H_2_0; iii. NaN_3_, DMF (see: Experimental part).*

*^a^Isolated yield.*

*^b^Crude reaction mixture (^19^F NMR and ^31^P NMR).*

*^c^Configuration (1R, 2S)/(1R, 2R)*.

As a result of the halide-induced oxirane opening, the only one regioisomer, tertiary bromide was observed in all cases, even in the case of derivatives of styrene and more substituted terminal cyclohexane oxiranes. The diastereoselectivity of the bromohydrine formations was analogous to those observed for the synthesis of compounds **5–8**. We observed the formation of both diastereoisomers of bromohydrine in the case of compound **32** (**32a:32b**, 4:1, d.r.), while single diastereoisomers (>99:1, d.r.) were obtained in the case of **33–35**. The analysis of the 2D NOESY and 1D H-F HOESY difference spectra of **32a** indicated that fluorine interacts with only one of the diastereotopic protons of CH_2_OH, while its geminal proton derived from the C*H*F moiety had a slightly different nOe effect with both methylene protons. To compare, in minor **32b** the nuclei of the C*HF* group correlated with both protons of the hydroxymethyl group indicating an opposite tendency compared to **9a, b**, and configurations rac (1*R*, 2*S*) for major **32a** and rac (1*R*, 2*S*) for minor **32b** were assigned. Moreover, the different nOe effects (1D NOE, 1D HOESY) with both methylene protons of CH_2_OH and the fluorine atom as well as the Me group indicated a similarity to interactions characterizing minor **32b**. To compare, the correlation between one of the methylene protons derived from C*H*_2_OH with C3 and axial C4&C6 together with a different enhancement of signals of C*HH*OH with fluorine observed on HOESY spectra suggested the axial arrangement of both fluorine and hydroxymethyl moiety together with the bulky equatorial phosphonate group as a predominant conformer of **35** with (1*R*, 2*S*) configuration. By analogy, the stereochemistry of one diastereoisomer of **33** as rac (1*R*, 2*S*) and **34** as rac (2*R*, 3*R*) were assigned. The additional diagnostic signals confirming tertiary bromide formation appeared in the ^13^C NMR spectra. Thus, signals of atom C-2 (Br) in the spectra of **32–35** were located around δ: 72–78 (^2^*J*_C−F_ 18–21 Hz), while in starting oxiranes C-2 signals appeared in the range δ: 55–63, and were slightly shifted compared to tertiary bromides such as 2-bromo-2-phenylpropane (Atack et al., 2014). Altogether, this allows us to suggest that the introduction of bromide via (protonated) oxirane ring-opening occurred through an S_N_2-like (mixed S_N_1/S_N_2) mechanism with the nucleophilic attack on a more substituted carbon atom. Usually, in the case of acid-mediated opening of derivatives of styrene oxide and more substituted terminal cyclohexane oxirane the formation of the mixture of regioisomers (with the predominance of a more substituted product) or partial racemization through carbocation takes place (Parker and Isaacs, 1959; Costantino et al., 1982; Bell and Ciaccio, 1986; Lin and Whalen, 1994; Padwaa and Murphree, 2006; Singh et al., 2007; Morton et al., 2009).

Similarly, the reactions of oxiranes **5–8** with sodium azides [NaN_3_, (NH_4_)_2_SO_4_, EtOH/H_2_0] (Głowacka, 2009) were conducted to give azidohydrine **36–39** as the single regioisomers with yields from 60% for **37** and **39** to 70% for **38** and diastereoselectivity from (4:1, d.r.) for **36** to the sole product in the case of **37–39** (>99:1, d.r; Table 6 method *ii*.). The application of ammonium chloride (Głowacka, 2009) instead of (NH_4_)_2_SO_4_ resulted in the formation of a mixture of appropriate chlorides and hydrolysis products. Additionally, it is worth noting that the introduction of azide in the case of **7** had to be conducted for 2 days, and gave monoester **38** (R = H). The analysis of IR (band at 2,100 cm^−1^) (Hesse et al., 1997) as well as the ^13^C NMR spectra of **36–39** confirmed the formation of tertiary azides. Thus, the signals of (*C-*OH) carbon atoms were located around δ: 73–79 (^3^*J*_C−P_ 19–21 Hz) while signals of *C*N_3_ appeared at δ: 57, similarly to 2-azido-2-phenylpropan-1-ol (Prasad et al., 2015). Although 3-hydroxyphosphonates **36–39** can rotate freely around the P-C1 and C1-C2 bonds, we would like to emphasize that in [D] chloroform they exist predominantly as their *anti*-conformers (arrangement of phosphonate and CH_2_OH moieties regarding C1-C2 bonds) as can be judged from the large (18–21 Hz) values of their ^3^*J*_C−P_ couplings together with the analysis of the NOESY and H-F HOESY spectra. In the case of major **36a**, similar interactions between the proton of the CHF group and fluorine (2D NOESY and 2D H-F HOESY) with both methylene C*H*_2_OH protons were observed, while in the case of the minor isomer **36b** the different enhancements of analogous signals due to nOe effects were detected, similarly to interactions in **32a, b**. In the case of **37**, we observed nOe effects (1D NOESY, 1D HOESY) related to **36a** nOe effects, while the interactions between fluorine and other protons in the **38** spectra corresponded to these detected for **36b**, as expected. Moreover, the different nOe effects (1D NOE, 1D HOESY) with both methylene protons of CH_2_OH and fluorine as well as the Me group indicated a similarity to interactions characterizing minor **32b**. Analogously to **35**, the correlation between one of the methylene protons derived from C*H*_2_OH with both C3 protons and axial C4 together with a different enhancement of signals of C*HH*OH with fluorine and the interaction of F with axial C3&C5 protons observed on the HOESY spectra of **39** indicated the (1*R*, 2*S*) configuration. These results are analogical to the stereochemistry determined for bromides **32–35** (Table 6). These observations confirmed that the oxirane ring-opening happened with inversion of configuration (S_N_2-like mechanism) and substitution occurred at the tertiary carbon atom in each case.

**Scheme 3:**
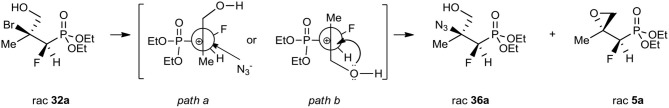
The preferred trajectory of nucleophilic attack during transformation of **32a**.

A conventional way to get the vicinal azido alcohols involves epoxides ring-opening (Benedetti et al., 1998; Fringuelli et al., 1999; Amantini et al., 2002; Badalassi et al., 2004; Nesterov and Kolodiazhnyi, 2007; Głowacka, 2009) or displacement of halohydrins (Draper, 1983; Iacazio and Réglier, 2005) under acidic/basic conditions with the application of different azide sources as well as chemoselectivity by the reduction of α-azido ketones (Rao et al., 1992; Ordóñez et al., 2003; Ankati et al., 2008). As an example, the opening of diethyl (*S*)-2,3-oxiranepropylphosphonate with sodium azide in the presence of (NH_4_)_2_SO_4_ resulted in the formation of single regioisomer (*R*)-3-azido-2-hydroxypropylphosphonate as well (Głowacka, 2009). To compare, the alternative reactions of bromohydrines **32–35** with sodium azide in dimethylformamide as a solvent (Table 6, method *iii*.) gave the mixture of azidohydrines **36–37**, **39**, or **38′** (R = Et) as major compounds, as well as appropriate starting oxiranes **5–8** (ratio: see *Experimental part*). To explain the observed diastereoselectivity, the reaction of bromide **32a, b** (4:1, d.r.) with NaN_3_ in DMF was carried out. As a result, the azide **36a, b** (3:1, d.r.; δ = 17.6:18.6 in ^31^P NMR) and epoxide **5a, b** (14:1, d.r.; δ 14.1:14.9 in ^31^P NMR) with a ratio for **36**/**5** as 5:1, were formed. Apparently, the formation of carbocation took place. Stereochemistry of the addition of azide ${\text{N}}_{3}^{-}$ to carbocation (like in carbonyl compound) was a consequence of the steric hindrance on the adjacent stereogenic center allowing them to attack from opposite the fluorine side (*path a*, Scheme 3). Moreover, partial racemization leading to both diastereoisomers of azide and formation of both diastereoisomeric epoxides as a result of the attack of a lone-pair nucleophile such as the hydroxyl group from the alternative side (*path b*, Scheme 3) supported the proposed course of the reaction.

Next, the obtained azidohydrines were applied to afford the vicinal β-amino-γ-hydroxy phosphonates. The reduction of azides **36–39** followed by protection of the nitrogen group [H_2_, Pd//C, (Boc_2_O) method *i*. or *ii*. Table 7] led to the *N*-Boc derivatives **40–42** or compound **27**. Subsequent acidic hydrolysis (conc HCl, reflux, 2–3 days, propylene oxide) (Wróblewski and Hałajewska-Wosik, 2003; Table 7, method *iii*.) surprisingly gave γ-amino-β-hydroxy phosphonates **29–31**, and **43** instead of their β-amino-γ-hydroxy regioisomers. Analysis of spectra confirmed that as the products compounds **29a, b** as a mixture (4:1, d.r.) and **30**–**31** as well as **43** [with tentatively assigned stereochemistry (1*R*,2*S*)] as single diastereoisomers were obtained.

**Table 7:**
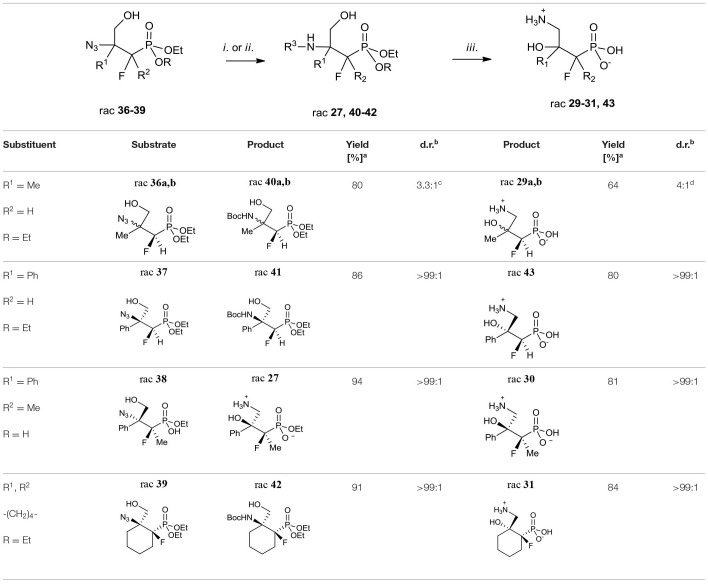
Synthesis of fluorinated γ-amino-β-hydroxy alkylphosphonic acids **29–31** and **43**.

*i. H_2_, Pd//C, Boc_2_O, EtOH. ii. H_2_, Pd//C, EtOH. iii. HCl conc., reflux, 3d, propylene oxide, EtOH.*

*^a^Isolated yield.*

*^b^Crude reaction mixture (^19^F NMR and ^31^P NMR).*

*^c^Configuration (1R, 2S)/(1R, 2R).*

*^d^Configuration (1R, 2R)/(1R, 2S)*.

**Scheme 4:**
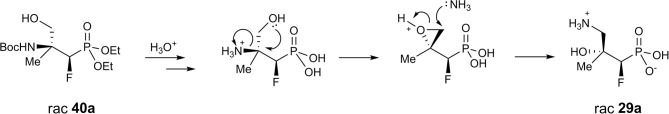
Proposed mechanism of the rearrangement of **40a** to **29a** during acid-mediated hydrolysis.

Apparently, during acid treatment the protonation of a more basic amino group takes place. Next, due to the attack of the neighboring group from the opposite side, the protonated oxirane is formed. Subsequent oxirane ring-opening by ammonia leads to rearrangement products such as γ-amino-β-hydroxy alkylphosphonic acid (Scheme 4).

Moreover, the formation of compound **27** supports the proposed mechanism. We already observed the participation of the neighboring group (substituted amines) during deoxyfluorination of α-hydroxy-β-aminophosphonate derivatives of amino acids, leading to β-fluoro-α*-*aminophosphonates (Kazmierczak and Koroniak, 2012; Kaczmarek et al., 2018).

## Conclusion

The diastereoselective reaction of monofluorinated β-ketophosphonates bearing methyl, phenyl, or cycloalkane substituents with diazomethane lead to oxiranes possessing a tertiary carbon atom. The heteronucleophiles-induced ring-opening, followed by acidic hydrolysis allowed us to obtain designed γ-amino-β-hydroxy alkylphosphonic acids. Moreover, epoxide ring-opening with HBr or NaN_3_ yielded substituted tertiary β-bromohydrines or β-azidoalcohol phosphonate derivatives. Subsequent azide reduction and acidic hydrolysis resulted in the formation of γ-amino-β-hydroxy alkylphosphonic acids, as the rearrangement products. Interestingly, in the cases of phenyl and cyclohexane derivatives, we observed excellent regio- and diastereoselectivity in the reactions.

We expect that our aminophosphonates and the observed rearrangement will found an application for drug design. To verify our assumptions concerning interactions of fluorine with neighboring groups in aminophosphonates, further syntheses will be carried out. We believe our methods will find tremendous application in the synthesis of biologically active molecules and useful intermediates.

## Materials and Methods

### General Information

^1^H NMR, ^13^C NMR, ^19^F NMR, and ^31^P NMR spectra were performed on Bruker ASCEND 400 (400 MHz), Bruker ASCEND 600 (600 MHz), and Varian Mercury (300 MHz) spectrometers, as is noted. The 2D and 1D selective NMR spectra (1D NOESY and 1D H-F HOESY) were recorded on Bruker ASCEND 600 (600 MHz) or Bruker ASCEND 400 (400 MHz) spectrometers. Chemical shifts of ^1^H NMR were expressed in parts per million downfield from tetramethylsilane (TMS) as an internal standard (δ = 0) in CDCl_3_. Chemical shifts of ^13^C NMR were expressed in parts per million downfield and upfield from CDCl_3_ as an internal standard (δ 77.16) or CD_3_OD (δ 49.00) or CF_3_COOD (δ 164.2) or traces of solvent. Chemical shifts of ^19^F NMR were expressed in parts per million upfield from CFCl_3._ The ethereal solution of diazomethane was prepared as described (Vogel et al., 1989). Compounds **1** (Radwan-Olszewska et al., 2011), **2** (Cox et al., 2005), **3** (Hamashima et al., 2005), and **4** (Kim, 2005) were prepared as described. The NMR data for **25** (McDonald et al., 1985; Huleatt et al., 2015) was in good agreement. For more information see Supplementary Materials.

### Theoretical Calculations

The quantum mechanical calculations of potential energy under vacuum at the M06/6-31+G^**^ (Hehre et al., 1986; Zhao and Truhlar, 2008) level of theory were performed using the GAUSSIAN09 program (Frisch et al., 2013), in order to systematically search for possible conformations. The vibrational frequencies were calculated using the same method, and then their positivity was applied to confirm that each of the calculated structures corresponded to a minimum on the potential energy surface. To simplify the calculations, the ethoxyl substituents were replaced with methoxyl substituents for which several conformations were calculated with the aim of choosing the global minimum-energy structure.

### Procedures

#### Procedure for Oxirane 5–8 Preparation

The ethereal solution of diazomethane was cooled in an ice-bath [distilled from the mixture of diazald (964 mg, 4.5 mmol)] in diethyl ether (15 mL) and KOH (252 mg, 4.5 mmol) [dissolved in EtOH (4.5 mL); yield 65–70%] (Vogel et al., 1989) was quickly cooled in an ice-bath and carefully added to the flask containing β-ketophosphonates **1–4** (1.5 mmol). Next, the tightly closed reaction mixture (with glass stopper) was stirred for 48 h at room temperature (monitored by NMR). The solvent was evaporated and the residue was purified by column chromatography to give compounds **5–8**.

***rac Diethyl ((R)-fluoro((R)-2-methyloxiran-2-yl)methyl)phosphonate*** (rac **5a)**, major isomer: Isolated as a mixture with **5b**, which could not be separated by the chromatography techniques employed in this study transparent oil (315 mg, 93%, 3.3:1 d.r.): ^1^H NMR (600 MHz, CDCl_3_) δ = 4.29 (dd, *J* = 45.8, 7.8 Hz, 1H, C*H*F), 4.16–4.06 (m, 4H, OC*H*_2_), 2.78 (“d,” *J* = 4.4 Hz, 1H, C*H*H), 2.66 (dd, *J* = 5.8, 4.4 Hz, 1H, CH*H*), 1.41 (s, 3H, C*H*_3_), 1.25 (t, *J* = 7.3 Hz, 3H, OCH_2_C*H*_3_), 1.24 (td, *J* = 7.3 Hz, 3H, OCH_2_C*H*_3_). ^13^C NMR (151 MHz, CDCl_3_) δ = 92.23 (dd, *J* = 187.7, 166.2 Hz, *C*FP), 63.47 (d, *J* = 6.8 Hz, O*C*H_2_), 62.97 (d, *J* = 6.6 Hz, O*C*H_2_), 55.28 (dd, *J* = 22.4, 9.2 Hz, *C*(OC)Me), 50.62 (d, *J* = 7.2 Hz, *C*H_2_), 16.36 (d, *J* = 3.7 Hz, *C*H_3_), 16.27 (d, *J* = 5.5 Hz, OCH_2_*C*H_3_), 16.29 (d, *J* = 5.5 Hz, OCH_2_*C*H_3_). ^19^F NMR (565 MHz, CDCl_3_) δ = −213.83 (ddd, *J* = 79.8, 45.8, 5.6 Hz). ^31^P{/^1^H} NMR (243 MHz, CDCl_3_) δ = 14.12 (d, *J* = 79.8 Hz). ^31^P NMR (122 MHz, CDCl_3_) δ = 14.12 (dh, *J* = 79.8, 7.9 Hz). MS (EI) *m/z* = 227.1 [M+H] ^+^ IR (film): ν = 2,985, 2,934, 1,445, 1,368, 1,256, 1,163, 1,017, and 971 cm^−1^.

***rac Diethyl ((R)-fluoro((S)-2-methyloxiran-2-yl)methyl)phosphonate*** (rac **5b)**, minor isomer: ^1^H NMR (600 MHz, CDCl_3_) δ = 4.52 (dd, *J* = 45.9, 5.7 Hz, 1H, C*H*F), 4.16–4.06 (m, 4H, OC*H*_2_), 2.92 (d, *J* = 4.8 Hz, 1H, C*H*H), 2.58 (dd, *J* = 4.8, 2.1 Hz, 1H, CH*H*), 1.40 (d, *J* = 1.5 Hz, 3H, C*H*_3_), 1.26 (t, *J* = 7.3 Hz, 3H, OCH_2_C*H*_3_), 1.24 (t, *J* = 7.3 Hz, 3H, OCH_2_C*H*_3_). ^13^C NMR (151 MHz, CDCl_3_) δ = 88.75 (dd, *J* = 187.6, 166.2 Hz, *C*FP), 63.47 (d, *J* = 6.8 Hz, O*C*H_2_), 62.97 (d, *J* = 6.6 Hz, O*C*H_2_), 54.56 (dd, *J* = 21.9, 5.3 Hz, *C*(OC)Me), 51.43 (t, *J* = 4.9 Hz, *C*H_2_), 17.86 (t, *J* = 1.9 Hz, *C*H_3_), 16.31 (d, *J* = 4.9 Hz, OCH_2_*C*H_3_), 16.26 (d, *J* = 5.1 Hz, OCH_2_*C*H_3_). ^19^F NMR (565 MHz, CDCl_3_) δ = −214.21 (dd, *J* = 74.0, 45.9 Hz). ^31^P{/^1^H} NMR (243 MHz, CDCl_3_) δ = 14.96 (d, *J* = 74.4 Hz). ^31^P NMR (122 MHz, CDCl_3_) δ = 14.96 (br dh, *J* = 74.2, 7.4 Hz).

***rac Diethyl ((R)-fluoro((R)-2-phenyloxiran-2-yl)methyl)phosphonate*** (rac **6)**, transparent oil (398 mg, 92%): ^1^H NMR (300 MHz, CDCl_3_) δ = 7.53–7.48 (m, 2H, Ph), 7.39–7.32 (m, 3H, Ph), 5.05 (dd, *J* = 45.4, 8.1 Hz, 1H, C*H*F), 4.19–4.04 (m, 2H, OC*H*_2_), 4.04–3.93 (m, 2H, OC*H*_2_), 3.40 (d, *J* = 5.2 Hz, 1H, C*H*H), 2.93 (td, *J* = 5.6, 0.7 Hz 1H, CH*H*), 1.26 (td, *J* = 7.1, 0.5 Hz, 3H, OCH_2_C*H*_3_), 1.15 (td, *J* = 7.1, 0.6 Hz, 3H, OCH_2_C*H*_3_). ^13^C NMR (75 MHz, CDCl_3_) δ = 136.45 (d, *J* = 1.9 Hz, Ph), 128.98, 128.54, 127.40 (3 × s, Ph), 90.75 (dd, *J* = 190.9 Hz, 168.1 Hz, *C*FP), 63.19 (dd, *J* = 12.5, 6.6 Hz, *C*(OC)Ph), 62.61 (d, *J* = 6.5 Hz, O*C*H_2_), 51.80 (dd, *J* = 7.9, 2.9 Hz, *C*H_2_), 16.19 (d, *J* = 6.0 Hz, OCH_2_*C*H_3_), 16.18 (d, *J* = 6.0 Hz, OCH_2_*C*H_3_). ^19^F NMR (282 MHz, CDCl_3_) δ = −210.19 (ddd, *J* = 78.3, 45.5, 5.5 Hz). ^31^P{/^1^H} NMR (121 MHz, CDCl_3_) δ = 12.79 (d, *J* = 78.3 Hz). ^31^P NMR (122 MHz, CDCl_3_) δ = 12.73 (dp, *J* = 78.4, 7.9 Hz). MS (EI) *m/z* = 288.0 [M^+^]. IR (film): ν = 2,985, 2,927, 1,642, 1,447, 1,391, 1,371, 1,258, 1,161, 1,017, and 974 cm^−1.^

***rac Diethyl ((R)-1-fluoro-1-((S)-2-phenyloxiran-2-yl)ethyl)phosphonate*** (rac **7)**, transparent oil (408 mg, 90%): ^1^H NMR (400 MHz, CDCl_3_) δ = 7.54–7.49 (m, 2H, Ph), 7.39–7.28 (m, 3H, Ph), 4.17–4.08 (m, 2H, OC*H*_2_), 4.05 (“quint,” *J* = 7.0 Hz, 2H, OC*H*_2_), 3.39 (dd, *J* = 5.1, 0.8 Hz, 1H, C*H*H), 2.88 (dd, *J* = 5.1, 2.7 Hz, 1H, CH*H*), 1.62 (dd, *J* = 24.7, 14.0 Hz, 3H, C*H*_3_), 1.23 (t, *J* = 7.1 Hz, 3H, OCH_2_C*H*_3_), 1.18 (t, *J* = 7.1 Hz, 3H, OCH_2_C*H*_3_). ^13^C NMR (101 MHz, CDCl_3_) δ = 135.95 (t, *J* = 1.8 Hz, Ph), 129.13 (d, *J* = 1.4 Hz, Ph), 128.35, 127.61 (2 × s, Ph), 95.91 (dd, *J* = 184.9, 171.7 Hz, *C*FP), 63.25 (d, *J* = 7.2 Hz, O*C*H_2_), 63.23 (dd, *J* = 7.0, 1.7 Hz, O*C*H_2_), 61.87 (dd, *J* = 23.4, 8.3 Hz, *C*(OC)Ph), 50.84 (dd, *J* = 6.2, 2.7 Hz, *C*H_2_), 19.26 (dd, *J* = 22.5, 2.2 Hz, *C*H_3_), 16.25 (t, *J* = 5.8 Hz, OCH_2_*C*H_3_), 16.19 (t, *J* = 5.8 Hz, OCH_2_*C*H_3_). ^19^F NMR (376 MHz, CDCl_3_) δ = −170.39 (dqd, *J* = 89.9, 24.9, 2.8 Hz). ^31^P{/^1^H} NMR (162 MHz, CDCl_3_) δ = 16.46 (d, *J* = 89.2 Hz). ^31^P NMR (122 MHz, CDCl_3_) δ = 16.47 (dqd, *J* = 89.6, 14.2, 7.1 Hz). MS (EI) *m/z* = 303.2 [M+H] ^+^.

***rac Diethyl ((3R,4R)-4-fluoro-1-oxaspiro[2.5]octan-4-yl)phosphonate*** (rac **8)**, transparent oil (379 mg, 95%): ^1^H NMR (400 MHz, CDCl_3_) δ = 4.29–4.24 (m, 2H, OC*H*_2_CH_3_), 4.24–4.19 (m, 2H, OC*H*_2_CH_3_), 3.30–3.27 (m, 1H, C*H*H, C2*H*), 2.53 (q, *J* = 4.7 Hz, CH*H*, C2*H*), 2.45–2.37 (m, 1H, C5*H*), 2.32–2.26 (m, 1H, C8*H*), 1.88–1.79 (m, 2H, C5*H*&C6*H*), 1.82–1.73 (m, 2H, C6*H*&C7*H*), 1.56–1.50 (m, 2H, C7*H*&C8*H*), 1.34 (t, *J* = 7.0 Hz, 3H, OCH_2_C*H*_3_), 1.33 (t, *J* = 7.0 Hz, 3H, OCH_2_C*H*_3_). ^1^H{/^19^F} NMR (400 MHz, CDCl_3_) δ = 4.23 (quint, *J* = 7.2 Hz, 2H), 4.16 (quint, *J* = 7.2 Hz, 2H), 3.24 (d, *J* = 5.3 Hz, 1H), 2.49 (d, *J* = 4.9 Hz, 1H), 2.45–2.35 (m, 1H), 2.26 (m, 1H), 1.88–1.79 (m, 1H), 1.81 (br d, *J* = 10.0 Hz, 1H), 1.75–1.63 (m, 2H), 1.54–1.48 (m, 2H), 1.34 (t, *J* = 7.0 Hz, 3H), 1.33 (t, *J* = 7.0 Hz, 3H). ^1^H{/^31^P} NMR (400 MHz, CDCl_3_) δ = 4.23 (q, *J* = 7.1 Hz, 2H), 4.16 (q, *J* = 7.1 Hz, 2H), 3.24 (d, *J* = 5.3 Hz, 1H), 2.48 (t, *J* = 4.9 Hz, 1H), 2.45–2.37 (m, 1H), 2.32–2.26 (m, 1H), 1.88–1.79 (m, 1H), 1.81 (br dd, *J* = 13.0, 10.0 Hz, 1H), 1.75–1.63 (m, 2H), 1.54–1.48 (m, 2H), 1.33 (t, *J* = 7.0 Hz, 3H), 1.32 (t, *J* = 7.0 Hz, 3H). ^13^C NMR (101 MHz, CDCl_3_) δ = 93.47 (dd, *J* = 191.1, 168.8 Hz, *C*FP, C4), 63.19 (d, *J* = 6.5 Hz, O*C*H_2_), 62.94 (d, *J* = 7.2 Hz, O*C*H_2_), 59.95 (d, *J* = 20.2 Hz, *C*(OC)C), 50.25 (dd, *J* = 10.3, 7.1 Hz, *C*H_2_O), 33.72 (dd, *J* = 18.8, 2.3 Hz, C5), 31.58 (d, *J* = 1.4 Hz, *C*8), 24.12 (s, *C*7), 21.64 (dd, *J* = 8.3, 1.7 Hz, *C*6), 16.30 (d, *J* = 6.3 Hz, OCH_2_*C*H_3_), 16.24 (d, *J* = 6.4 Hz, OCH_2_*C*H_3_). ^19^F NMR (376 MHz, CDCl_3_) δ = −173.04 (br d, *J* = 90.3 Hz). ^31^P{/^1^H} NMR (162 MHz, CDCl_3_) δ = 16.88 (d, *J* = 88.4 Hz). ^31^P NMR (122 MHz, CDCl_3_) δ = 16.89 (ddq, *J* = 87.6, 24.5, 8.2 Hz). MS (EI) *m/z* = 268.1 [M+H]^+^.

#### General Procedure (Procedure A) for Oxiranes 5, 7–8 Opening by Secondary or Primary Amine

To the mixture of secondary or primary amine (0.48 mmol) and triethylamine (56 μL, 40 mg, 0.4 mmol) dissolved in EtOH (2 mL), epoxides **5–8** (0.4 mmol) were added. Next, the reaction mixture was heated in an oil bath at 60°C for 24–60 h (monitoring by TLC). Then, the reaction mixture was evaporated and purified by flash column chromatography (1 cm layer of silica gel) with chloroform CHCl_3_ → 5% MeOH/CHCl_3_ (v:v) to give appropriate amino alcohols **9–12** and **17–24**. For the spectroscopic properties of compounds **10–12**, **18–20**, and **22–24** see Supplementary Materials.

#### General Procedure (Procedure B) for Oxirane **6** Opening by Secondary or Primary Amine

To the mixture of secondary or primary amine (2 mmol) and triethylamine (279 μL, 200 mg, 2 mmol) dissolved in EtOH (2 mL), epoxide **6** (0.4 mmol) was added. Next, the reaction mixture was heated in an oil bath at 60°C for 24–60 h (monitoring by TLC). Then the reaction mixture was diluted with CH_2_Cl_2_ (20 mL) and aqueous HCl (1 M, 10 mL) was added. The mixture was extracted with CH_2_Cl_2_ (3 ×20 mL). The combined extracts were washed with aqueous sodium bicarbonate, brine, dried over Na_2_SO_4_, filtrated, and concentrated under reduced pressure. The residue was purified by flash column chromatography (1 cm layer of silica gel) with CHCl_3_ → 5% MeOH/CHCl_3_ (v:v) to give a mixture of **6**, appropriate amino alcohols **13–16**, and allylic alcohol **25**. For spectroscopic properties of compounds **14–16** see Supplementary Materials.

***rac Diethyl ((1R,2R)-3-(dibenzylamino)-1-fluoro-2-hydroxy-2-methylpropyl)phosphonate*** (rac **9a)**, procedure A (Bn_2_NH, TEA). Major isomer: isolated as a mixture with **9b**, which could not be separated by the chromatography techniques employed in this study; transparent oil (145 mg, 85%, 3:1 d.r.): ^1^H NMR (300 MHz, CDCl_3_) δ = 7.35–7.31 (m, 10H, Ph), 4.54 (dd, *J* = 44.9, 4.9 Hz, 1H, C*H*F), 4.26–4.06 (m, 4H, OC*H*_2_), 3.86 (d, *J* = 13.9 Hz, 2H, C*H*HPh), 3.54 (d, *J* = 13.7 Hz, 2H, CH*H*Ph), 2.86–2.82 (m, 1H, C*H*H), 2.79 (dd, *J* =14.1, 2.0 Hz, 1H, CH*H*), 1.36–1.33 (m, 3H, C*H*_3_), 1.33–1.30 (m, 3H, OCH_2_C*H*_3_), 1.30–1.28 (m, 3H, OCH_2_C*H*_3_). ^13^C NMR (101 MHz, CDCl_3_) δ = 140.34, 128.51, 128.27, 127.06 (4 × s, Ph), 91.79 (dd, *J* = 188.3, 162.5 Hz, *C*FP), 73.64 (dd, *J* = 17.9, 2.2 Hz, *C*OH), 64.01 (dd, *J* = 6.9, 1.9 Hz, O*C*H_2_), 62.65 (d, *J* = 6.8 Hz, O*C*H_2_), 59.79 (d, *J* = 3.9 Hz, *C*H_2_Ph), 59.05 (dd, *J* = 7.9, 4.6 Hz *C*H_2_N), 23.08 (*C*H_3_), 16.51 (d, *J* = 5.3 Hz, OCH_2_*C*H_3_), 16.46 (d, *J* = 5.3 Hz, OCH_2_*C*H_3_). ^19^F NMR (283 MHz, CDCl_3_) δ = −213.28 (dd, *J* = 78.5, 44.8 Hz). ^31^P{/^1^H} NMR (122 MHz, CDCl_3_) δ = 16.81 (d, *J* = 78.6 Hz). IR (film): ν = 3,331, 3,061, 2,980, 2,925, 2,850, 2,797, 1,643, 1,604, 1,494, 1,453, 1,366, 1,258, 1,056, 1,028, and 976 cm^−1^ GC–MS *m/z* = 424 [M+H]^+^ *t*_*R*_ = 14.60 min.

***rac Diethyl ((1R,2S)-3-(dibenzylamino)-1-fluoro-2-hydroxy-2-methylpropyl)phosphonate*** (rac **9b)**, minor isomer: ^1^H NMR (300 MHz, CDCl_3_) δ = 7.35–7.31 (m, 10H, Ph), 4.87 (dd, *J* = 44.7, 2.6 Hz, 1H, C*H*F), 4.26–4.06 (m, 4H, OC*H*_2_), 3.85 (d, *J* = 13.9 Hz, 2H, C*H*HPh), 3.65 (d, *J* = 13.7 Hz, 2H, CH*H*Ph), 2.88 (dt, *J* = 13.4, 1.4 Hz, 1H, C*H*H), 2.61 (ddd, *J* = 14.2, 3.1, 2.0 Hz, 1H, CH*H*), 1.33–1.30 (m, 3H, C*H*_3_), 1.30–1.28 (m, 3H, OCH_2_C*H*_3_), 1.28–1.26 (m, 3H, OCH_2_C*H*_3_). ^19^F NMR (283 MHz, CDCl_3_) δ = −209.25 (ddd, *J* = 71.4, 44.8, 2.0 Hz). ^31^P{/^1^H} NMR (122 MHz, CDCl_3_) δ = 18.21 (d, *J* = 71.5 Hz).

***rac Diethyl ((1R,2R)-3-(dibenzylamino)-1-fluoro-2-hydroxy-2-phenylpropyl phosphonate*** (rac **13)**, procedure B (Bn_2_NH, TEA). Isolated as a mixture with **6** and **25**, which could not be separated by the chromatography techniques employed in this study, (**6**/**13/25** crude ratio: 20/40/40, NMR) transparent oil (rac **13** 68 mg, 35%; **25** 18 mg, 30%): ^1^H NMR (300 MHz, CDCl_3_): δ = 7.20–7.51 (m, 15H, Ph), 5.1 (s, 1H, OH), 4.79 (dd, *J* = 45.0, 5.9 Hz, 1H, C*H*F), 4.0–4.15 (m, 2H, OCH_2_), 3.7–3.8 (m, 2H, OCH_2_), 3.59 (d, *J* = 12.7 Hz, 1H, C*H*H), 3.37 (d, *J* = 13.5 Hz, 2H, C*H*HPh), 3.32 (dd, *J* = 13.6, 1.8 Hz, 1H, CH*H*Ph), 3.12 (dd, *J* = 13.6, 1.2 Hz, 1H, CH*H*), 1.23 (td, *J* = 7.1, 0.5 Hz, 3H, OCH_2_C*H*_3_), 1.02 (td, *J* = 7.1, 0.4 Hz, 3H, OCH_2_C*H*_3_). ^19^F NMR: δ = −212.15 (dd, *J* = 81.8, 44.9 Hz). ^31^P{/^1^H} NMR: δ = 15.96 (d, *J* = 81.9 Hz). EI–MS *m/z* = 396.2 [M-Bn+2H]^+.^

***rac Ethyl hydrogen ((2R,3S)-4-(dibenzylamino)-2-fluoro-3-hydroxy-3-phenylbutan-2-yl)phosphonate*** (rac **17)**, procedure A (Bn_2_NH, TEA), reaction time 60 h; white solid (132 mg, 89%): ^1^H NMR (400 MHz, CDCl_3_) δ = 7.62–7.53 (m, 1H, Ph), 7.42–7.31 (m, 12H, Ph), 7.32–7.26 (m, 2H, Ph), 4.12–4.04 (m, 2H, OC*H*_2_CH_3_), 3.85 (s, 4H, NC*H*_2_Ph), 3.43 (d, *J* = 5.1 Hz, 1H, C*H*H), 2.93 (dd, *J* = 5.2, 2.7 Hz, 1H, CH*H*), 1.67 (dd, *J* = 24.7, 14.0 Hz, 3H, C*H*_3_), 1.28 (t, *J* = 7.1 Hz, 3H, OCH_2_C*H*_3_). ^13^C NMR (101 MHz, CDCl_3_) δ = 139.58 (s, Ph), 136.01 (d, *J* = 1.8 Hz, Ph), 129.17, 128.45, 128.34, 127.66, 127.12 (4 × s, Ph) 95.97 (dd, *J* = 185.0, 171.6 Hz, *C*FP), 63.29 (dd, *J* = 7.0, 2.4 Hz, O*C*H_2_), 61.91 (dd, *J* = 23.5, 8.3 Hz, *C*OH), 52.84 (s, *C*H_2_Ph), 50.91 (dd, *J* = 6.2, 2.7 Hz, *CN*), 19.32 (dd, *J* = 22.7, 2.1 Hz, *C*H_3_), 16.27 (t, *J* = 5.9 Hz, OCH_2_*C*H_3_). ^19^F NMR (376 MHz, CDCl_3_) δ = −170.28 (dqd, *J* = 89.4, 24.8, 2.5 Hz). ^31^P{/^1^H} NMR (162 MHz, CDCl_3_) δ = 16.51 (d, *J* = 90.1 Hz). MS(ESI) C_26_H_32_FNO_4_P [M+H]^+^ calc. 472.21, found 472.26.

***rac Diethyl ((1R,2R)-2-((dibenzylamino)methyl)-1-fluoro-2-hydroxycyclohexyl) phosphonate*** (rac **21)**, procedure A (Bn_2_NH, TEA), slightly yellow oil (166 mg, 87%): ^1^H NMR (400 MHz, CDCl_3_): δ = 7.26–7.21 (m, 10H, Ph), 4.06–3.94 (m, 4H, OC*H*_2_), 3.94 (d, *J* = 14.3 Hz, 2H, NC*H*_2_Ph), 3.51 (d, *J* = 13.6 Hz, 2H, NC*H*_2_Ph), 3.31 (d, *J* = 14.0 Hz, 1H, C*H*H), 2.79 (d, *J* = 14.1 Hz, 1H, CH*H*), 2.18–2.10 (m, 1H, C6H), 2.15–2.01 (m, 2H, C6&3H), 1.92–1.86 (m, 1H, C3H), 1.70–1.65 (br s, 1H, OH), 1.68–1.64 (m, 1H, C4H), 1.54–1.46 (m, 2H, C4&5H), 1.46–1.40 (m, 2H, C4&5H), 1.40–1.36 (m, 1H, C3H), 1.30 (t, *J* = 7.2 Hz, 3H, OCH_2_C*H*_3_), 1.26 (t, *J* = 7.3 Hz, 3H, OCH_2_C*H*_3_). ^13^C NMR (101 MHz, CDCl_3_): δ = 139.51, 129.15, 128.47, 127.02 (4 × s, Ph), 97.37 (dd, *J* = 190.1, 161.7 Hz, *C*FP, C1), 73.60 (dd, *J* = 21.6, 2.2 Hz, *C*OH, C2), 63.18 (d, *J* = 6.4 Hz, O*C*H_2_), 63.16 (d, *J* = 6.4 Hz, O*C*H_2_), 60.01 (s, *C*H_2_Ph), 55.77–55.69 (m, *C*N), 32.37 (dd, *J* = 8.1, 0.6 Hz, *C*H_2_, C3), 29.11 (dd, *J* = 20.2, 2.2 Hz, *C*H_2_, C6), 20.21 (s, *C*H_2_, C4), 20.05 (dd, *J* = 9.1, 3.1 Hz, *C*H_2_, C5), 16.48 (d, *J* = 5.9 Hz, OCH_2_*C*H_3_), 16.47 (d, *J* = 6.0 Hz, OCH_2_*C*H_3_). ^19^F NMR (377 MHz, CDCl_3_): δ = −180.09 (br s). ^31^P{/^1^H} NMR (162 MHz, CDCl_3_): δ = 20.09 (d, *J* = 83.6 Hz). IR (film): ν = 3,688, 3,609, 3,054, 2,985, 2,871, 1,456, 1,368, 1,263, and 1,027 cm^−1^. GC–MS (EI) *m/z* = 374.2 [M-Bn+2H]^+^, *t*_*R*_ = 18.32 min.

#### General Procedure (Procedure C) for Hydrogenation and N-Boc Protection of γ-Amino-β-Hydroxyphosphonates

A solution of *N, N*-dibenzyl-protected hydroxyphosphonate (0.3 mmol) in absolute EtOH (2 mL) containing Boc_2_O (98 mg, 0.45 mmol) was hydrogenated over 10% Pd-C (30 mg) under atmospheric pressure for 48 h. Then, the catalyst was filtrated through Celite with MeOH, the solution was concentrated on vacuum, and purified by flash column chromatography CHCl_3_ → 5% MeOH/CHCl_3_ (v:v) (1 cm layer of silica gel), to give appropriate *N*-Boc-protected amino hydroxyphosphonate.

#### General Procedure (Procedure D) for Hydrogenation of Monoesters of Hydroxyalkylphosphonic Acids

A solution of monoester of *N, N*-dibenzyl-protected or azido-hydroxyphosphonic acids **17** or **38** (0.3 mmol) in absolute EtOH (2 mL) was hydrogenated over 10% Pd-C (30 mg) under atmospheric pressure for 48 h. Then, the catalyst was filtrated through Celite with MeOH, the solution was concentrated on vacuum, and purified by flash column chromatography CHCl_3_ → 5% MeOH/CHCl_3_ (v:v) (1 cm layer of silica gel) to give monoester of amino hydroxyphosphonic acid **27**. For spectroscopic properties of compounds **26–28** *(procedures C, D)* see Supplementary Materials.

#### General Procedure (Procedure E) for Acidic Hydrolysis of Hydroxyphosphonates

To the appropriated hydroxyphosphonates **26–28** and **40–42** (0.25 mmol), aqueous HCl (12 M, 2 ml) was added. Next, the mixture was refluxed for 48 h, then evaporated and co-evaporated with ethanol (3 × 4 mL). To the obtained residue, ethanol (1 mL) was added, and the mixture was treated dropwise with propylene oxide (to pH 7). The residue was filtered off, evaporated, dissolved in methanol, and evaporated to give **29–31**, or **43** as white amorphous, low-soluble (in polar solvents at rt) powder, which decomposed before melting.

***rac Hydrogen ((1R,2R)-3-ammonio-1-fluoro-2-hydroxy-2-methylpropyl)phosphonate*** (rac **29a**), procedure E, major isomer. Isolated as a mixture with **29b**, which could not be separated, white solid (41 mg, 88%, 3:1, d.r.): ^1^H NMR (300 MHz, D_2_O) δ = 4.55 (dd, *J* = 44.6, 6.4 Hz, 1H, C*H*FP), 3.22 (d, *J* = 13.1 Hz, 1H, C*H*H), 3.10 (d, *J* = 13.1 Hz, 1H, CH*H*), 1.38 (p, *J* = 2.5 Hz, 3H, C*H*_3_). ^13^C NMR (101 MHz, D_2_O) δ = 93.50 (dd, *J* = 181.4, 152.5 Hz, *C*FP), 70.62 (d, *J* = 18.8 Hz, *C*OH), 45.70 (d, *J* = 11.1 Hz, *C*N), 20.81 (t, *J* = 3.5 Hz*, C*H_3_). ^19^F NMR (283 MHz, D_2_O) δ = −209.12 (dd, *J* = 69.6, 45.9 Hz). ^31^P{/^1^H} NMR (122 MHz, D_2_O) δ = 9.95 (d, *J* = 69.2 Hz). IR (film): ν = 3,349, 2,980, 2,933, 1,712, 1,518, 1,392, 1,367, 1,167, 1,029, and 974 ${\text{cm}}_{.}^{-1}$ HRMS calc for C_4_H_12_FNO_4_P 188.0488; found 188.0483 [M+H]^+^.

***rac Hydrogen ((1R,2S)-3-ammonio-1-fluoro-2-hydroxy-2-methylpropyl)phosphonate*** (rac **29b**), minor isomer: ^19^F NMR (283 MHz, D_2_O) δ = −207.23 (dd, *J* = 69.1, 44.9 Hz).

^31^P{/^1^H} NMR (122 MHz, D_2_O) δ = 9.39 (d, *J* = 67.1 Hz).

***rac Hydrogen ((2R,3S)-4-ammonio-2-fluoro-3-hydroxy-3-phenylbutan-2-yl)phosphonate*** (rac **30**), procedure E, slightly cream-colored solid (57 mg, 86%): ^1^H NMR (300 MHz, D_2_O) δ = 7.47–7.39 (m, 2H, Ph), 7.34–7.24 (m, 3H, Ph), 3.78 (d, *J* = 13.3 Hz, 1H, C*H*H), 3.55 (d, *J* = 13.4 Hz, 1H, CH*H*), 1.16 (ddd, *J* = 25.5, 12.7, 1.4 Hz, 3H, C*H*_3_). ^13^C NMR (76 MHz, D_2_O) δ = 137.21 (d, *J* = 7.2 Hz, Ph), 128.84 (s, Ph), 128.74 (s, Ph), 127.27 (d, *J* = 2.8 Hz, Ph), 97.36 (dd, *J* = 186.4, 152.2 Hz, *C*FP), 76.79 (d, *J* = 19.5 Hz, *C*OH), 45.73 (s, *C*N), 18.56 (d, *J* = 21.4 Hz, *C*H_3_). ^13^C NMR (101 MHz, CF_3_COOD) δ = 136.94 (d, *J* = 8.4 Hz, Ph), 132.28, 131.59, 128.30 (3 × s, Ph), 98.66 (dd, *J* = 188.3, 165.2 Hz, *C*FP), 79.81 (d, *J* = 21.3 Hz), 48.83 (s, *C*N), 19.70 (d, *J* = 21.3 Hz, C*H*_3_). ^19^F NMR (283 MHz, D_2_O) δ = −169.07 (dtd, *J* = 74.5, 30.1, 22.5 Hz). ^31^P{/^1^H} NMR (122 MHz, D_2_O) δ = 14.78 (d, *J* = 77.7 Hz). IR (film): ν = 3,182 (br), 2,938, 2,868, 1,447, 1,404, 1,205, 1,044, and 940 cm^−1^. HRMS calc for C_10_H_16_FNO_4_P 264.0801; found 264.0799 [M+H]^+^.

***rac Hydrogen ((1R,2R)-2-(ammoniomethyl)-1-fluoro-2-hydroxycyclohexyl)phosphonate*** (rac **31**), procedure E, white solid (47 mg, 82%). Alternatively, compound rac **31** can be obtain from rac **42** by procedure E (45 mg, 81%): ^1^H NMR (401 MHz, CD_3_OD) δ = 3.45 (d, *J* = 13.2 Hz, 1H, C*H*HOH), 2.91 (d, *J* = 12.8 Hz, 1H, C*H*HOH), 2.26–2.00 (m, 2H, C*H*_2_), 2.23–1.63 (m, 2H, C*H*_2_), 1.64–1.49 (m, 4H, C*H*_2_). ^13^C NMR (101 MHz, D_2_O) δ = 96.14 (dd, *J* = 183.2, 153.7 Hz, *C*FP, C1), 70.96 (dd, *J* = 22.9, 2.1 Hz, *C*OH, C2), 45.80 (d, *J* = 2.0 Hz, CN), 31.46 (d, *J* = 6.5 Hz, C3), 28.73 (dd, *J* = 20.0, 2.9 Hz, C6), 19.47 (s, C4), 19.21 (dd, *J* = 8.3, 3.3 Hz, C5). ^13^C NMR (101 MHz, CF_3_COOD) δ = 97.06 (dd, *J* = 181.1, 169.0 Hz, *C*FP, C1), 74.21 (d, *J* = 24.5 Hz, *C*OH, C2), 48.14 (*C*N), 35.48 (C3), 30.19 (d, *J* = 19.9 Hz, C6), 21.25 (C4), 20.52 (C5). ^19^F NMR (283 MHz, D_2_O) δ = −178.24 (dd, *J* = 74.2, 43.6 Hz). ^19^F NMR (283 MHz, CD_3_OD) δ = −179.99 (dd, *J* = 75.7, 47.1 Hz). ^31^P{/^1^H} NMR (122 MHz, D_2_O) δ = 14.95 (d, *J* = 77.1 Hz). ^31^P{/^1^H} NMR (122 MHz, CD_3_OD) δ = 16.18 (d, *J* = 75.9 Hz). IR (film): ν = 3,185 (br), 2,936, 2,865, 1,454, 1,338, 1,147, 1,052, 1,033, 1,012, and 956 ${\text{cm}}_{.}^{-1}$ HRMS calc for C_7_H_16_FNO_4_P 228.0801; found 228.0797 [M+H]^+^.

#### General Procedure (Procedure F) for Oxiranes 5–8 Opening by HBr

After being dissolved in chloroform (1.5 mL), acethyl bromide (44 μL, 74 mg, 0.6 mmol) and methanol (24 μL, 19 mg, 0.6 mmol) were mixed at 0°C and the reaction mixture was stirred for 20 min. Then, oxiranes **5–8** (0.5 mmol) in chloroform (1.5 mL) were added, and stirring was continued at 0°C for 2 h. Next, the crude reaction mixture was extracted with CH_2_Cl_2_ (NaHCO_3aq_/brine), dried with anhydrous Na_2_SO_4_, and evaporated to give bromohydrines **32–35**. Flash column chromatography CHCl_3_ → 5% MeOH/CHCl_3_ (v:v) (1 cm layer of silica gel) gave compounds with lower yields. For spectroscopic properties of compounds **32–35** see Supplementary Materials.

#### General Procedure (Procedure G) for Oxiranes 5–8 Opening by Sodium Azide

To the dissolved mixture of EtOH:H_2_O (8:1, v:v) oxiranes **5–8** (0.5 mmol), sodium azide (150 mg, 2.3 mmol) and ammonium sulfate (132 mg, 1 mmol) were added. Then, the obtained mixture was refluxed for 24/48 h (monitored by TLC). Next, the crude reaction mixture was extracted with CH_2_Cl_2_ (NaHCO_3aq_/brine), dried (Na_2_SO_4_), evaporated, and purified by flash column chromatography CHCl_3_ → 5% MeOH/CHCl_3_ (v:v) (1 cm layer of silica gel) to give compounds **36–39**.

#### General Procedure (Procedure H) for Reaction of Bromides 32–35 With Sodium Azide

Bromides **32–35** (0.3 mmol) were dissolved in DMF (2 mL), then sodium azide (91 mg, 1.4 mmol) was added. Then, the obtained mixture was stirred for 48 h (monitored by TLC). Next, the crude reaction mixture was extracted with CH_2_Cl_2_ (NaHCO_3aq_/brine), dried (Na_2_SO_4_), evaporated, and purified by flash column chromatography to give the mixture of appropriate azides **36–39** and oxiranes **5–8** which could not be separated by the chromatography techniques employed in this study (^31^P NMR).

***rac Diethyl ((1R,2S)-2-azido-1-fluoro-3-hydroxy-2-methylpropyl)phosphonate*** (rac **36a**), major isomer. Procedure G (24 h): isolated as a mixture with **36b**, which could not be separated by the chromatography techniques employed in this study; slightly yellow oil (86 mg, 64%, 4:1, d.r.). Procedure H gave a mixture **36a, b** (3:1, d.r.) / **5a, b** (5:1, d.r.) with ratio 1.5:1. ^1^H NMR (300 MHz, CDCl_3_) δ = 4.67 (dd, *J* = 45.1, 5.8 Hz, 1H, C*H*F), 4.33–4.17 (m, 4H, OC*H*_2_), 3.78 (br s, 1H, OH), 3.49 (d, *J* = 12.1, 1.8 Hz, 1H, C*H*H), 3.45 (d, *J* = 12.4 Hz, 1H, CH*H*), 1.42–1.39 (m, 6H, C*H*_3_, OCH_2_C*H*_3_), 1.39–1.38 (m, 3H, OCH_2_C*H*_3_). ^13^C NMR (101 MHz, CDCl_3_) δ = 90.18 (dd, *J* = 188.4, 163.7 Hz, *C*FP), 73.30 (dd, *J* = 18.3, 2.4 Hz, *C*OH), 64.30 (dd, *J* = 6.8, 2.0 Hz, O*C*H_2_), 62.83 (d, *J* = 7.0 Hz, O*C*H_2_CH_3_), 56.59 (dd, *J* = 7.9, 5.6 Hz, *C*N), 21.95 (t, *J* = 3.3 Hz, *C*H_3_), 16.27 (d, *J* = 6.0 Hz, OCH_2_*C*H_3_), 16.26 (d, *J* = 6.0 Hz, OCH_2_*C*H_3_). ^19^F NMR (376 MHz, CDCl_3_) δ = −213.89 (dd, *J* = 76.9, 44.8 Hz). ^31^P{/^1^H} NMR (243 MHz, CDCl_3_) δ = 17.57 (d, *J* = 77.5 Hz). IR (film): ν = 3,368, 2,985, 2,923, 2,853, 2,103, 1,237, 1,163, 1,023, 973, and 549 cm^−1^. HRMS (EI-MS) calcd for C_8_H_18_FN_3_O_4_P [M]^+^: 270.1019, found: 270.1033.

***rac Diethyl ((1R,2R)-2-azido-1-fluoro-3-hydroxy-2-methylpropyl)phosphonate*** (rac **36b)**, minor isomer: ^1^H NMR (300 MHz, CDCl_3_) δ = 4.82 (dd, *J* = 44.9, 3.6 Hz, 1H, C*H*F), 4.33–4.17 (m, 2H, OC*H*_2_), 4.13–4.08 (m, 2H, OC*H*_2_), 3.85 (br s, 1H, OH), 3.41 (dt, *J* = 12.7, 2.0 Hz, 1H, C*H*H), 3.36 (d, *J* = 12.6, 2.3 Hz, 1H, CH*H*), 1.39–1.38 (m, 3H, C*H*_3_), 1.38–1.32 (m, 6H, OCH_2_C*H*_3_). ^19^F NMR (376 MHz, CDCl_3_) δ = −210.73 (dd, *J* = 71.7, 44.6 Hz). ^31^P{/^1^H} NMR (243 MHz, CDCl_3_) δ = 18.60 (d, *J* = 71.5 Hz).

***rac Diethyl ((1R,2S)-2-azido-1-fluoro-3-hydroxy-2-phenylpropyl)phosphonate*** (rac **37)**, procedure G (24 h): transparent oil (99 mg, 60%). Procedure H gave a mixture **37**/ **6** with a ratio of 3.8:1. ^1^H NMR (300 MHz, CDCl_3_) δ = 7.57–7.34 (m, 5H, Ph), 5.07 (dd, *J* = 45.5, 6.6 Hz, 1H, C*H*F), 4.22 (“quint,” *J* = 7.6 Hz, 2H, OC*H*_2_), 3.74 (ddd, *J* = 12.3, 2.4, 0.8 Hz, 1H, C*H*H), 3.69 (dd, *J* = 12.5, 1.2 Hz, 1H, CH*H*), 3.75–3.64 (m, 1H, OC*H*HCH_3_), 3.36–3.26 (m, 1H, OCH*H*), 1.33 (t, *J* = 7.0 Hz, 3H, OCH_2_C*H*_3_), 0.90 (t, *J* = 7.1 Hz, 3H, OCH_2_C*H*_3_). ^13^C NMR (101 MHz, CDCl_3_) δ = 138.70 (d, *J* = 4.7 Hz, Ph), 128.36, 128.32, 126.02 (3 × s, Ph), 89.55 (dd, *J* = 191.8, 166.0 Hz, *C*FP), 76.86 (s, COH masked by CHCl_3_), 64.44 (dd, *J* = 6.6, 2.5 Hz, O*C*H_2_), 62.44 (d, *J* = 6.9 Hz, O*C*H_2_), 57.33 (dd, *J* = 12.1, 5.5 Hz, *C*N), 16.33 (d, *J* = 5.8 Hz, OCH_2_*C*H_3_), 15.77 (d, *J* = 6.1 Hz, OCH_2_*C*H_3_). ^19^F NMR (282 MHz, CDCl_3_) δ = −214.83 (dd, *J* = 82.2, 45.5 Hz). ^31^P{/^1^H} NMR (121 MHz, CDCl_3_) δ = 16.29 (d, *J* = 82.2 Hz). IR (film): ν = 3,369, 2,985, 2,933, 2,102, 1,530, 1,520, 1,231, 1,162, 1,020, 976, and 702 cm^−1^. MS (EI) *m*/*z* = 332.2 [M+H]^+^.

***rac Ethyl hydrogen ((2R,3R)-3-azido-2-fluoro-4-hydroxy-3-phenylbutan-2-yl)phosphonate*** **(**rac **38)** and ***rac diethyl ((2R,3R)-3-azido-2-fluoro-4-hydroxy-3-phenylbutan-2-yl)phosphonate*** **(**rac **38′)**, procedure G (48 h, evaporation, flush column chromatography 95:5 → 9:1 CHCl_3_/MeOH, v:v) gave **38** Compound **38** had: ^1^H NMR (300 MHz, DMSO-*d*_6_) δ = 7.51–7.19 (m, 6H, Ph, OH), 4.33 (d, *J* = 13.2 Hz, 1H, C*H*H), 3.89 (quintet, *J* = 7.0 Hz, 2H, OC*H*_2_), 3.70 (dd, *J* = 13.2, 2.6 Hz, 1H, CH*H*), 1.16 (t, *J* = 7.1 Hz, 3H, OCH_2_C*H*_3_), 1.04 (dd, *J* = 24.9, 11.4 Hz, 3H, C*H*_3_). ^13^C NMR (76 MHz, DMSO-*d*_6_) δ = 140.65 (d, *J* = 10.0 Hz, Ph), 127.34 (s, Ph), 127.11 (d, *J* = 3.0 Hz, Ph), 126.73 (s, Ph), 96.99 (dd, *J* = 190.6, 144.3 Hz, *C*FP), 79.04 (dd, *J* = 19.7, 5.0 Hz, *C*OH), 60.85 (d, *J* = 5.9 Hz, O*C*H_2_), 56.93 (s, *C*H_2_), 19.19 (dd, *J* = 21.5, 4.0 Hz, *C*H_3_), 16.94 (d, *J* = 5.6 Hz, OCH_2_*C*H_3_). ^19^F NMR (283 MHz, DMSO-*d*_6_) δ = −170.77 (dq, *J* = 68.9, 24.8 Hz). ^31^P{/^1^H} NMR (122 MHz, DMSO-*d*_6_) δ = 12.43–14.36 (m). MS (ESI) calc for C_12_H_16_FNO_4_P^−^ [M-N_2_]^−^ 288.081 obtained 288.09. Compound **38'**: ^19^F NMR (376 MHz, CDCl_3_) δ = −171.86 (dq, *J* = 84.3, 25.0 Hz). ^31^P{/^1^H} NMR (162 MHz, CDCl_3_) δ = 19.55 (d, *J* = 84.1 Hz).

***rac Diethyl ((1R,2S)-2-azido-1-fluoro-2-(hydroxymethyl)cyclohexyl)phosphonate*** (rac **39)**, procedure G (24 h): transparent oil (93 mg, 60%). Procedure H gave a mixture **39**/**8** with a ratio **of** 1:1.^1^H NMR (400 MHz, CDCl_3_) δ = 4.31–4.18 (m, 4H, OC*H*_2_), 3.82 (d, *J* = 12.9 Hz, 1H, C*H*H), 3.34 (dd, *J* = 12.9, 2.0 Hz, 1H, CH*H*), 2.27–2.13 (m, 1H, C6H), 2.12–2.04 (m, 1H, C6H), 2.04–1.92 (m, 1H, C3H), 1.78–1.65 (m, 1H, C4H), 1.65–1.55 (m, 2H, C3&C5H), 1.55–1.50 (m, 2H, C4&C5H), 1.39 (dt, *J* = 7.0,1.1 Hz, 6H, OCH_2_C*H*_3_). ^13^C NMR (101 MHz, CDCl_3_) δ = 95.9 (dd, *J* = 193.1, 160.5 Hz, *C*FP, C1), 73.6 (dd, *J* = 21.1, 2.4 Hz, *C*OH), 64.16 (d, *J* = 7.2 Hz, O*C*H_2_), 63.98(d, *J* = 7.9, 1.7 Hz, O*C*H_2_), 57.7 (d, *J* = 1.7 Hz, *C*N, C2), 30.0 (d, *J* = 8.5 Hz, *C*H_2_, C3), 28.3 (dd, *J* = 20.5, 2.8 Hz, *C*H_2_, C6), 19.4 (s, *C*H_2_, C4), 19.6 (dd, *J* = 10.1, 2.5 Hz, *C*H_2_, C5), 16.5 (d, *J* = 5.7 Hz, OCH_2_*C*H_3_), 16.4 (d, *J* = 5.9 Hz, OCH_2_*C*H_3_). ^19^F NMR (377 MHz, CDCl_3_) δ = −180.95 (dd, *J* = 78.2, 43.4 Hz). ^31^P{/^1^H} NMR (162 MHz, CDCl_3_) δ = 19.51 (d, *J* = 79.8 Hz). IR (film): ν = 3,364, 2,938, 2,869, 2,102, 1,234, 1,165, 1,023, and 973 cm^−1^. MS (EI) *m*/*z* = 309.2 [M]^+^.

#### General Procedure (Procedure I) for Hydrogenation and N-Boc Protection of β-Azido-γ-Hydroxyphosphonates

A solution of azidohydroxyphosphonates **36–37** and **39** (0.3 mmol) in absolute EtOH (2 mL) containing Boc_2_O (98 mg, 0.45 mmol) was hydrogenated over 10% Pd-C (30 mg) under atmospheric pressure for 48 h. Then, the catalyst was filtrated through Celite with MeOH, the solution was concentrated on vacuum, and purified by flash column chromatography CHCl_3_ → 5% MeOH/CHCl_3_ (v:v) (1 cm layer of silica gel) to give appropriate *N*-Boc-protected amino hydroxyphosphonate. For spectroscopic properties of compounds **40–42** see Supplementary Materials.

***rac Hydrogen ((1R,2R)-2-ammonio-1-fluoro-3-hydroxy-2-phenylpropyl)phosphonate*** (rac **43**), procedure E, white solid (52 mg, 84%): ^1^H NMR (300 MHz, D_2_O) δ = 7.53–7.49 (m, 2H, Ph), 7.44–7.35 (m, 3H, Ph), 5.05 (dd, *J* = 44.9, 6.1 Hz, 1H, C*H*F), 3.66 (br d, *J* = 13.0 Hz, 1H, C*H*H), 3.38 (d, *J* = 13.2 Hz, 1H, CH*H*). ^13^C NMR (101 MHz, D_2_O) δ = 137.17, 128.45, 128.33, 125.44 (4 × s, Ph), 92.05 (dd, *J* = 185.1, 153.3 Hz, *C*FP), 74.23 (d, *J* = 18.2 Hz, *C*OH), 46.22 (s, *C*N). ^31^P{/^1^H} NMR (122 MHz, D_2_O) δ = 9.65 (d, *J* = 71.2 Hz). ^19^F NMR (283 MHz, D_2_O) δ = −207.56 (dd, *J* = 71.3, 45.0 Hz). IR (film): ν = 3,391, 3,146, 3,050, 2,937, 2,864, 1,454, 1,410, 1,042, and 955 cm^−1^. HRMS calc for C_9_H_14_FNO_4_P^+^ 250.0639; found 250.0643 [M+H]^+^.

## Data Availability Statement

The original contributions presented in the study are included in the article/Supplementary Material, further inquiries can be directed to the corresponding author/s.

## Author Contributions

MR and KM-M carried out chemical synthesis, characterization, and manuscript writing. AW carried out chemical synthesis and compounds characterization. PK participated to structure determination, manuscript writing, and revision. TC performed part of the NMR experiments and contributed to the structure determination, manuscript writing, and revision. TS carried out the DFT calculations. MR and HK designed and managed the study. All authors listed have made a substantial, direct and intellectual contribution to the work, and approved it for publication.

## Conflict of Interest

The authors declare that the research was conducted in the absence of any commercial or financial relationships that could be construed as a potential conflict of interest.
