# Supplementary material for: Highly Diastereoselective Construction of Carbon– Heteroatom Quaternary Stereogenic Centers in the Synthesis of Analogs of Bioactive Compounds: From Monofluorinated Epoxyalkylphosphonates to α-Fluoro-, β-, or γ-Amino Alcohol Derivatives of Alkylphosphonates
Source: Front Chem. 2021 Jun 2;9:613633. doi: 10.3389/fchem.2021.613633 (PMC8208234; doi:10.3389/fchem.2021.613633)
Supplement: Supplementary file 1 [file Data_Sheet_1.pdf]

Supplementary Material

1 Supplementary Data

1.1. *General procedures* 1

2.1. Procedure A for oxiran **5,7-8** *opening by secondary or primary amine* 2

2.2. Procedure B for oxiran **6** *opening by secondary or primary amine* 2

2.3. Spectroscopic properties of compounds **10-12 14-16, 18-20, 22-24** 2

3.1. Procedure C for hydrogenation and N-Boc protection of γ-amino-β-hydroxyphosphonates. 7

3.2. Procedure D for hydrogenation of monoesters of hydroxyalkylphosphonic acids 7

3.3. Spectroscopic properties of compounds **26-28** 7

4.1. Procedure F for oxiran **5-8** *opening by HBr* 8

4.2. Spectroscopic properties of compounds **32-35** 8

5.1. Procedure I for hydrogenation and N-Boc protection of β-azido-γ-hydroxyphosphonates 10

5.2. Spectroscopic properties of compounds **40-42** 10

6. ^{1}H, ^{13}C, ^{19}F NMR and ^{31}P NMR Spectra of Compounds 12

1.1. *General informations*

^{1}H NMR, ^{13}C NMR, ^{19}F NMR and ^{31}P NMR spectra were performed on Bruker ASCEND 400 (400 MHz), Bruker ASCEND 600 (600 MHz), Varian Mercury (300 MHz), spectrometers, as is noted. The 2D and 1D selective NMR spectra were recorded on Bruker ASCEND 600 (600 MHz) or Bruker ASCEND 400 (400 MHz) spectrometers. Chemical shifts of ^{1}H NMR were expressed in parts per million downfield from tetramethylsilane (TMS) as an internal standard (δ = 0) in $CDCl_3$. Chemical shifts of ^{13}C NMR were expressed in parts per million downfield and upfield from $CDCl_3$ as an internal standard (δ 77.16) or CD_3OD (δ 49.00) or CF_3COOD (δ 164.2) or traces of solvent. Chemical shifts of ^{19}F NMR were expressed in parts per million upfield from $CFCl_3$ as an internal standard (δ 0) in $CDCl_3$. Chemical shifts of ^{31}P NMR were expressed in parts per million in $CDCl_3$. The yields of reaction and purity of the obtained products as well as diastereoisomers ratio (in crude reaction mixture) were conveniently evaluated by ^{19}F or/and ^{31}P NMR in $CDCl_3$ or by GC–MS method. GC–MS spectra were performed on Varian GC–MS 4000 spectrometer (conditions: flow rate of 1 mL/min, injector temperature = 220 °C, column oven temperature 40 °C (3 min) → 15 °C/min →

280 °C (10 min), using chloroform as the solvent. MS (ESI) spectra were performed on ZQ4000 Waters Mass Spectrometer. High-resolution mass spectra were recorded by electron spray (MS-ESI) techniques using QToF Impact HD Bruker spectrometer. IR analysis on spectrometer JASCO FT/IR-4600 were performed. Reagent grade chemicals were used and solvents were dried by refluxing with CaH_2 (CH_3CN) and distilled under an argon atmosphere. All moisture sensitive reactions were carried out under an argon atmosphere using oven-dried glassware. TLC was performed on Merck Kieselgel 60-F_{254} with EtOAc/hexane or $CHCl_3$/MeOH as developing systems, and products were detected by inspection under UV light (254 nm) and a standard procedure (solution of phosphomolybdenic acid or $KMnO_4$). Merck Kieselgel 60 (230–400 mesh) was used for column chromatography. All starting materials (with an exception of Selectfluor) were supplied by Sigma Aldrich. Selectfluor was supplied by Apollo Scientific (UK). Sodium hydride as 60% dispersion in mineral oil was used. Absolute ethanol was stored under argon and over molecular sieves 3Å. All moisture sensitive reactions were carried out under argon atmosphere using oven dried glassware. Compounds from methyl series (R^1=Me, R^2 = H) have to be carefully evaporated, dried and stored at around 4 °C. The etheral solution of diazomethane was prepared as described [52]. Compounds **1** [53], **2** [54], **3** [55], **4** [56] were prepared as described. The NMR data for **25** [35] was in good agreement.

1.2. Theoretical calculations

The quantum mechanical calculations of potential energy under vacuum at the M06/6-31+G** [57, 58] level of theory have been performed using the GAUSSIAN09 program,[59] in order to systematically search for possible conformations. The vibrational frequencies were calculated using the same method, and then their positivity was applied to confirm that each of the calculated structures corresponds to a minimum on the potential energy surface. To simplified the calculations, the ethoxyl substituents were replaced with methoxyl substituents for which several conformations were calculated with the aim of choosing the global minimum-energy structure.

*2.1. General procedure (procedure A) for oxiran **5,7-8** opening by secondary or primary amine*

*General procedure (procedure A) for oxiran **5,7-8** ring opening by secondary or primary amine* To the mixture of secondary or primary amine (0.48 mmol) and triethylamine (56 μL, 40 mg, 0.4 mmol) dissolved in EtOH (2 mL) epoxide **5-8** (0.4 mmol) was added. Next, the reaction mixture was heated in an oil bath at 60°C during 24-60 h (monitoring by TLC). Then, reaction mixture was evaporated and purified by flash column chromatography (1 cm layer of silica gel) with $CHCl_3$→5% MeOH/$CHCl_3$ (v:v) to give appropriate amino alcohols **9-12**, **17-24**.

*2.2. General procedure (procedure B) for oxiran **6** opening by secondary or primary amine*

To the mixture of secondary or primary amine (2 mmol) and triethylamine (279 μL, 200 mg, 2 mmol) dissolved in EtOH (2 mL) epoxide **6** (0.4 mmol) was added. Next, the reaction mixture was heated in an oil bath at 60°C during 24-60 h (monitoring by TLC). Then the reaction mixture was diluted with CH_2Cl_2 (20 mL) and aqueous HCl (1M, 10 mL) was added. The mixture was extracted with CH_2Cl_2 (3 x 20 mL). The combined extracts were washed with aqueous sodium bicarbonate, brine, dried over Na_2SO_4, filtrated and concentrated under reduced pressure. The residue was purified by flash column chromatography (1 cm layer of silica gel) with $CHCl_3$→5% MeOH/$CHCl_3$ (v:v) to give a mixture of **6**, appropriate aminoalcohols **13-16** and allylic alcohol **25**.

2.3. Spectroscopic properties of compounds **10-12**, **14-16**, **18-20** and **22-24**

rac Diethyl ((1R,2R)-3-(benzylamino)-1-fluoro-2-hydroxy-2-methylpropyl)phosphonate **(**rac**10a)** Procedure A ($BnNH_2$, TEA), major isomer. Isolated as a mixture with **10b**, which could not be separated by the chromatography techniques employed in this study; transparent oil (107 mg, 80%, 3:1 d.r.): ^{1}H NMR (400 MHz, $CDCl_3$) δ = 7.35 – 7.27 (m, 5H, Ph), 4.69 (dd, *J* = 44.9, 5.5 Hz, 1H, C*H*F), 4.27 – 4.10 (m, 4H, OC*H*$_2$), 3.85 (s, 2H, NCH_2), 3.04 (d, *J* = 12.6 Hz, 1H, C*H*H), 2.63 (dd, *J* = 12.6, 2.2 Hz, 1H, C*H*H), 1.39 – 1.33 (m, 9H, C*H*$_3$, OCH_2C*H*$_3$).^{13}C NMR (101 MHz, $CDCl_3$) δ = 139.08, 128.52, 128.24, 127.33 (4 x s, Ph), 92.48 (dd, *J* = 187.2, 162.8 Hz, *C*FP), 72.01 (dd, *J* = 18.7, 2.9 Hz, *C*OH), 63.73 (dd, *J* = 6.7, 1.5 Hz, O*C*H$_2$), 62.81 (d, *J* = 6.7 Hz O*C*H$_2$), 54.59 – 54.29 (m, CN), 54.13 (s, CH_2Ph), 23.35 (d, *J* = 4.3 Hz, *C*H$_3$), 16.41 (d, *J* = 6.2 Hz, OCH_2*C*H$_3$), 16.34 (d, *J* = 6.1 Hz, OCH_2*C*H$_3$). ^{19}F NMR (376 MHz, $CDCl_3$) δ = -213.98 (dd, *J* = 77.0, 44.9 Hz, 1F). ^{31}P{/^{1}H} NMR (162 MHz, $CDCl_3$) δ = 16.61 (d, *J* = 77.1 Hz, 1P). MS (EI) *m/z* = 333.3 $[M]^+$

rac Diethyl ((1R,2S)-3-(benzylamino)-1-fluoro-2-hydroxy-2-methylpropyl)phosphonate **(**rac **10b)** minor isomer:^{1}H NMR (400 MHz, $CDCl_3$) δ = 7.35 – 7.27 (m, 5H, Ph), 4.96 (dd, *J* = 44.6, 3.0 Hz, 1H, C*H*F), 4.27 – 4.10 (m, 4H, OC*H*$_2$), 3.84 (s, 2H, NCH_2), 2.90 (dd, *J* = 12.4, 2.1 Hz, 1H, C*H*H), 2.64 (dd, *J* = 12.3, 2.3 Hz, 1H, C*H*H), 1.39 – 1.33 (m, 9H, C*H*$_3$, OCH_2C*H*$_3$). ^{19}F NMR (377 MHz, $CDCl_3$) δ = -210.74 (dd, *J* = 71.5, 44.4 Hz). ^{31}P{/^{1}H} NMR (162 MHz, $CDCl_3$) δ = 17.69 (d, *J* = 71.6 Hz).

rac Diethyl ((1R,2R)-1-fluoro-2-hydroxy-2-methyl-3-(((S)-1-phenylethyl)amino)propyl) phosphonate **(**rac **11a)** ***and diethyl ((1S,2S)-1-fluoro-2-hydroxy-2-methyl-3-(((S)-1-phenylethyl)amino)propyl)phosphonate*** **(**rac **11b)** Procedure A ((*S*)-PhCH(Me)NH_2, TEA), major isomers: isolated as a mixture with **11'a,b**, which could not be separated by the chromatography techniques employed in this study; transparent oil (111 mg, 80%, crude **11a:11b/11'a:11'b**, 3:3/1:1, d.r.): ^{1}H NMR (400 MHz, $CDCl_3$) δ = 7.36 – 7.30 (m, 10H, Ph), 4.65 (dd, *J* = 44.9, 5.5 Hz, 1H, C*H*F), 4.63 (dd, *J* = 44.8, 5.7 Hz, 1H, C*H*F), 4.34 – 4.14 (m, 8H, OC*H*$_2$), 4.12 – 3.99 (m, 2H, C*H*H), 3.97 (dt, *J* = 14.4, 6.8 Hz, 2H, CH*H*), 3.79 (q, *J* = 6.6 Hz, 1H, C*H*Me), 3.79 (d, *J* = 6.6 Hz, 1H, C*H*Me), 1.44 – 1.29 (m, 24H, OCH_2C*H*$_3$, C*H*$_3$). ^{13}C NMR (101 MHz, $CDCl_3$) δ 144.79, 144.59 (2 x s, Ph), 128.70 (d, *J* = 2.4 Hz, Ph), 128.61, 127.29, 127.23, 126.80, 126.67 (5 x s, Ph), 92.80 (dd, *J* = 187.2, 163.1 Hz, *C*FP), 92.74 (dd, *J* = 187.4, 162.8 Hz, *C*FP), 72.03 (dd, *J* = 18.5, 2.9 Hz, *C*OH), 71.77 (dd, *J* = 18.9, 2.5 Hz, *C*OH), 63.80 (d, *J* = 6.8 Hz, O*C*H$_2$), 63.74 (d, *J* = 7.1 Hz O*C*H$_2$), 62.91 (d, *J* = 6.9 Hz, O*C*H$_2$), 62.80 (d, *J* = 7.3 Hz, O*C*H$_2$), 59.16 (s, *C*H(CH_3)), 58.84 (s, *C*H(CH_3)), 53.45 – 53.01 (m, 2 x CN), 24.25 (s, *C*H$_3$), 23.97 (s, *C*H$_3$), 23.15 (d, *J* = 4.1 Hz, *C*H$_3$), 23.11 (d, *J* = 4.1 Hz, *C*H$_3$), 16.50 (d, *J* = 6.0 Hz, OCH_2*C*H$_3$), 16.44 (d, *J* = 6.0 Hz, OCH_2*C*H$_3$). ^{19}F NMR (377 MHz, $CDCl_3$) δ = -213.63 (dd, *J* = 77.5, 44.8 Hz, 1F), -214.06 (dd, *J* = 77.4, 44.9 Hz, 1F). ^{31}P{/^{1}H} NMR (162 MHz, $CDCl_3$) δ = 16.72 (d, *J* = 77.6 Hz, 1P), 16.61 (d, *J* = 77.4 Hz, 1P). MS (EI) *m/z* = 348.1 $[M+H]^+$

rac Diethyl ((1R,2S)-1-fluoro-2-hydroxy-2-methyl-3-(((S)-1-phenylethyl)amino)propyl) phosphonate **(**rac **11'a)** and ***diethyl ((1S,2R)-1-fluoro-2-hydroxy-2-methyl-3-(((S)-1-phenylethyl)amino)propyl) phosphonate*** **(**rac **11'b)** Minor isomers: **11'a:11'b** (1:1, d.r.): ^{31}P{/^{1}H} NMR (162 MHz, $CDCl_3$) δ = 18.06 (d, *J* = 71.0 Hz, 1P), 17.61 (d, *J* = 72.5 Hz, 1P). ^{19}F NMR (377 MHz, $CDCl_3$) δ = -210.47 (ddd, *J* = 72.3, 44.8, 1.5 Hz, 1F), -210.49 (ddd, *J* = 70.8, 44.4, 1.5 Hz, 1F).

rac Diethyl ((1R,2R)-1-fluoro-2-hydroxy-2-methyl-3-(methyl((R)-1-phenylethyl)amino) propyl)phosphonate **(rac 12a) and *diethyl ((1S,2S)-1-fluoro-2-hydroxy-2-methyl-3-(methyl((R)-1-phenylethyl)amino)propyl)phosphonate* (rac 12b)** Procedure A ((*R*)-PhCH(Me)NH(Me), TEA),

major isomers: isolated as a mixture with 1**2'a,b**, which could not be separated by the chromatography techniques employed in this study; transparent oil (120 mg, 83%), crude 1**2a:12b/12'a:12'b**, 3:3/1:1, d.r.): ^{1}H NMR (400 MHz, $CDCl_3$) δ = 7.54 – 7.48 (m, 4H, Ph), 7.42 – 7.33 (m, 6H, Ph), 4.67 (ddd, *J* = 44.7, 4.8, 2.7 Hz, 1H, C*H*FP), 4.62 (ddd, *J* = 45.7, 14.2, 5.4 Hz, 1H, C*H*FP), 4.30 – 4.14 (m, 4H, OC*H*$_2$, C*H*CH$_3$), 4.13 – 4.01 (m, 2H, OC*H*$_2$), 4.04 – 3.90 (m, 2H, OC*H*$_2$), 3.91 – 3.83 (m, 2H, OC*H*$_2$), 2.87 – 2.52 (m, 2H, C*H*H), 2.84 (d, *J* = 14.1 Hz, 1H, CH*H*), 2.78 (d, *J* = 14.1 Hz, 1H, CH*H*), 2.35 (s, 6H, C*H*$_3$), 2.29 (s, 1H, OH), 2.27 (s, 1H, OH), 1.68 (d, *J* = 6.7 Hz, 6H, C*H*$_3$), 1.43 – 1.36 (m, 12H, OCH$_2$C*H*$_3$), 1.34 – 1.27 (m, 6H, C*H*$_3$). ^{13}C NMR (151 MHz, $CDCl_3$): δ = 135.99, 129.20, 129.03, 128.04 (4 x s, Ph), 92.32 (dd, *J* = 187.8, 166.3 Hz, *C*FP), 92.10 (dd, *J* = 188.4, 163.6 Hz, *C*FP), 72.01 (d, *J* = 17.4 Hz, *C*OH), 71.67 (d, *J* = 20.2 Hz, *C*OH), 63.57 (d, *J* = 7.6 Hz, *CH*CH$_3$), 63.56 (d, *J* = 7.6 Hz, *CH*CH$_3$), 63.42 (d, *J* = 6.9 Hz, O*C*H$_2$), 63.07 (d, *J* = 6.7 Hz, O*C*H$_2$), 55.45 (d, *J* = 9.2 Hz, *C*N), 55.30 (d, *J* = 9.2 Hz, *C*N), 50.73 (s, *C*HCH$_3$), 50.71 (s, *C*HCH$_3$), 43.32 (s, *C*H$_3$), 41.34 (s, *C*H$_3$), 22.75 (s, *C*H$_3$), 22.70 (s, *C*H$_3$), 21.96 (d, *J* = 2.0 Hz, *C*H$_3$), 21.94 (d, *J* = 1.7 Hz, *C*H$_3$), 16.43 (d, *J* = 3.8 Hz, OCH$_2$*C*H$_3$), 16.37-16.33 (m, OCH$_2$*C*H$_3$), 16.31 (d, *J* = 5.8 Hz, OCH$_2$*C*H$_3$). ^{19}F NMR(376 MHz, $CDCl_3$) δ = -213.42 (dd, *J* = 79.9, 45.8 Hz, 1F), -213.43 (dd, *J* = 79.9, 45.8 Hz, 1F). ^{31}P{/^{1}H} NMR (162 MHz, $CDCl_3$) δ = 13.40 (d, *J* = 80.0 Hz, 1P), 16.11 (br d, *J* = 78.2 Hz, 1P). GC–MS (EI) *m/z* = 362.2 $[M+H]^+$.

rac* *Diethyl ((1R,2S)-1-fluoro-2-hydroxy-2-methyl-3-(methyl((R)-1-phenylethyl)amino) propyl) phosphonate **(**rac **12'a)** and ***diethyl ((1S,2R)-1-fluoro-2-hydroxy-2-methyl-3-(methyl((R)-1-phenylethyl)amino)propyl)phosphonate*** **(**rac **12'b)** Minor isomers: **12'a:12'b** (1:1, d.r.): ^{19}F NMR (376 MHz, $CDCl_3$) δ = -215.41 (dd, *J* = 78.3, 44.7 Hz, 2F). ^{31}P{/^{1}H} NMR (162 MHz, $CDCl_3$) signals masked by major isomers.

rac Diethyl ((1R,2R)-3-(benzylamino)-1-fluoro-2-hydroxy-2-phenylpropyl)phosphonate **(**rac **14)** Procedure B ($BnNH_2$, TEA). Isolated as a mixture with **6** and **25**, which could not be separated by the chromatography techniques employed in this study (**6**/**14/25** crude ratio: 50/20/30, NMR), slightly creamy-colored oil (rac **14,** 32 mg, 20%; **25** 17 mg, 28%): ^{1}H NMR (300 MHz, $CDCl_3$): δ = 7.20 – 7.51 (m, 15H, Ph), 5.10 (s, 1H, OH), 4.79 (dd, *J* = 45.0, 5.9 Hz, 1H, C*H*F), 4.03 – 4.15 (m, 2H, OCH$_2$), 3.82 – 3.75 (m, 2H, OCH$_2$), 3.59 (d, *J* = 12.7 Hz, 1H, C*H*HPh), 3.37 (d, *J* = 13.5 Hz, 1H, C*H*HPh), 3.32 (dd, *J* = 13.6, 1.8 Hz, 1H, C*H*H), 3.12 (dd, *J* = 13.6, 1.2 Hz, 1H, CH*H*), 1.23 (td, *J* = 7.1, 0.5 Hz, 3H, OCH$_2$C*H*$_3$), 1.02 (td, *J* = 7.1, 0.4 Hz, 3H, OCH$_2$C*H*$_3$). ^{19}F NMR: δ = -212.15 (dd, *J* = 81.8, 45.0 Hz); ^{31}P{/^{1}H} NMR δ = 15.96 (d, *J* = 81.8 Hz). MS (EI) *m/z* = 395.3 $[M]^+$

rac Diethyl ((1R,2R)-1-fluoro-2-hydroxy-2-phenyl-3-(((S)-1-phenylethyl)amino)propyl) phosphonate **(**rac **15a)** ***and diethyl ((1S,2S)-1-fluoro-2-hydroxy-2-phenyl-3-(((S)-1-phenylethyl)amino)propyl)phosphonate*** **(**rac **15b)** Procedure B ((*S*)-PhCH(Me)NH$_2$, TEA). Isolated as a mixture with **6** and **25**, which could not be separated by the chromatography techniques employed in this study, oil **6/15a,b/25** with crude ratio 40/20/35%, NMR (rac **15a,b** 36 mg, 22%; **25** 18 mg, 30%): ^{1}H NMR: (300 MHz, CDCl3) δ = 7.70 - 7.58 (m, 4H, Ph), 7.42 - 7.28 (m, 16H, Ph), 4.88 (dd, *J* = 45.1, 5.9 Hz, 1H, CHF), 4.74 (dd, *J* = 45.3, 5.5 Hz, 1H, CHF), 4.18 (q, *J* = 7.1Hz, 4H, OCH2), 4.13-4.02 (m, 4H, OCH$_2$), 3.99 - 3.88 (m, 2H, C*H*H), 3.84-3.81 (m, 2H, CH*H*), 2.94 („q", *J* = 7.0 Hz, 2H, C*H*CH$_3$), 1.26 (t, *J* = 7.1 Hz, 6H, OCH$_2$C*H*$_3$), 1.25 (t, *J* = 6.9 Hz, 6H, OCH$_2$C*H*$_3$), 1.18 (d, *J* = 6.8 Hz, 6H, C*H*$_3$).^{19}F NMR (283 MHz, $CDCl_3$): δ -212.32 (dd, *J* = 79.9, 44.6 Hz, 1F), -212.10 (dd, *J* = 78.0, 44.5 Hz, 1F).^{31}P NMR (162 MHz, $CDCl_3$)**:** δ 16.24 (d, *J* = 80.0 Hz), 15.97 (d, *J* = 78.3 Hz). MS (EI) *m/z* = 394.2 $[M-Me]^+$.

rac* *Diethyl ((1R,2R)-1-fluoro-2-hydroxy-3-(methyl((R)-1-phenylethyl)amino)-2-phenylpropyl) phosphonate and ***rac diethyl ((1S,2S)-1-fluoro-2-hydroxy-3-(methyl((R)-1-phenylethyl)amino)-2-phenylpropyl)phosphonate*** **(*rac*16a,b)** Procedure B ((*R*)-PhCH(Me)NH(Me), TEA). Isolated as a mixture with **6** and **25**, which could not be separated by the chromatography techniques employed in this study, oil **6**/**16a,b**/**25** with crude ratio 20/40/40%, NMR) (rac**16a,b** 59 mg, 35%, 1:1, d.r.; **25** 19 mg, 32%): ^{1}H NMR (300 MHz, $CDCl_3$): δ = 7.65 –7.55 (m, 8H), 7.38 – 7.28 (m, 12H), 5.02 (dd, *J* = 46.1, 5.9 Hz, 1H, C*H*F), 4.94 (dd, *J* = 46.3, 5.5 Hz, 1H, C*H*F), 4.15 (dq, *J* = 7.1, 0.6 Hz, 2H, OCH_2), 4.13 (q, *J* = 7.1 Hz, 2H, OCH_2), 3.99 – 3.88 (m, 2H, C*H*H), 3.84 (q, *J* = 6.7 Hz, 4H, OCH_2), 3.64 – 3.60 (m, 1H, CH*H*), 2.94 (q, *J* = 7.0 Hz, 2H, C*H*CH_3), 2.95 – 2.93 (m, 1H, CH*H*), 1.28 (td, *J* = 7.1, 0.6 Hz, 6H, OCH_2CH_3), 1.27 (t, *J* = 6.9 Hz, 6H, OCH_2CH_3), 1.19 (d, *J* = 6.8 Hz, 6H, CH_3); ^{19}F NMR (376 MHz, $CDCl_3$): δ = -211.32 (dd, *J* = 80.0, 44.6 Hz, 1F), -211.60 (dd, *J* = 79.0, 44.5 Hz, 1F); ^{31}P{/^{1}H} NMR (162 MHz, $CDCl_3$): δ = 15.84 (d, *J* = 80.9 Hz), 15.97 (d, *J* = 81.3 Hz). MS (EI) *m*/*z* = 408.4 $[M-Me]^+$.

rac ***Ethyl hydrogen ((2R,3S)-4-(benzylamino)-2-fluoro-3-hydroxy-3-phenylbutan-2-yl)phosphonate*** **(**rac **18)** Procedure A ($BnNH_2$, TEA), reaction time 60h; white solid (127 mg, 83%): ^{1}H NMR (400 MHz, $CDCl_3$) δ = 7.44 – 7.20 (m, 10H, Ph), 6.18 (s, 2H, N*H*, O*H*), 4.05 (q, *J* = 7.3 Hz, 2H, OCH_2), 3.95 – 3.71 (m, 3H, C*H*H, NCH_2Ph), 3.17 (d, *J* = 12.9 Hz, 1H, CH*H*), 1.24 (t, *J* = 7.0 Hz, 3H, OCH_2CH_3), 1.06 (dd, *J* = 25.2, 11.6 Hz, 3H, CH_3); ^{13}C NMR (101 MHz, $CDCl_3$) δ = 142.09 (d, *J* = 9.3 Hz, Ph), 140.11, 129.41, 128.75, 128.39, 128.07, 127.77 (6 x s, Ph), 126.04 (d, *J* = 3.1 Hz, Ph), 98.03 (dd, *J* = 188.2, 150.8 Hz, *C*FP), 77.29 (d, *J* = 11.2 Hz, *C*OH), 62.48 (dd, *J* = 6.4, 3.0 Hz, O*C*H_2), 52.68 (s, CH_2Ph), 51.99 (s, CN), 20.07 (dd, *J* = 20.9, 3.2 Hz, *C*H_3), 16.85 (d, *J* = 6.1 Hz, OCH_2CH_3); ^{19}F NMR (377 MHz, $CDCl_3$) δ = -170.16 (dq, *J* = 73.0, 25.1 Hz); ^{31}P{/^{1}H} NMR (162 MHz, $CDCl_3$) δ = 15.50 (d, *J* = 72.9 Hz). MS (ESI) calc. for $C_{19}H_{26}FNO_4P$ 382.151, found 382.30.

Ethyl hydrogen ((2R,3S)-2-fluoro-3-hydroxy-3-phenyl-4-(((S)-1-phenylethyl)amino)butan-2-yl)phosphonate **(**rac **19a)** and ***ethyl hydrogen ((2S,3R)-2-fluoro-3-hydroxy-3-phenyl-4-(((S)-1-phenylethyl)amino)butan-2-yl)phosphonate*** **(**rac **19b)** Procedure A ((*S*)-PhCH(Me)NH_2, TEA), reaction time 60h; white solid (123 mg, 78%, 1:1, d.r.): ^{1}H NMR (300 MHz, $CDCl_3$) δ = 7.45 – 7.24 (m, 20H, Ph), 4.24 – 4.01 (m, 4H, OCH_2), 4.04 – 3.91 (m, 2H, C*H*CH_3), 3.80 (d, *J* = 14.4 Hz, 1H, C*H*H), 3.69 (d, *J* = 13.3 Hz, 1H, C*H*H), 2.96 (dd, *J* = 13.2, 2.8 Hz, 1H, CH*H*), 2.91 (dd, *J* = 13.2, 2.8 Hz, 1H, CH*H*), 2.37 (s, 2H, O*H*), 1.68 (d, *J* = 6.6 Hz, 3H, CH_3), 1.65 (d, *J* = 6.6 Hz, 3H, CH_3), 1.29 (t, *J* = 7.4 Hz, 3H, OCH_2CH_3), 1.28 (t, *J* = 7.4 Hz, 3H, OCH_2CH_3), 1.16 (d, d, *J* = 25.2, 11.4 Hz, 3H, CH_3), 1.04 (dd, *J* = 25.1, 11.3 Hz, 1H, CH_3). ^{13}C NMR (76 MHz, CDCl3) δ = 142.81 (d, *J* = 9.1 Hz, Ph), 142.69 (d, *J* = 9.2 Hz, Ph), 138.34, 137.63, 129.08, 129.03, 128.87, 128.71, 128.67, 128.48, 128.44, 128.27, 127.71, 127.63 (12 x s, Ph), 125.57 (d, *J* = 2.9 Hz, Ph), 125.45 (d, *J* = 3.0 Hz, Ph), 98.54 (dd, *J* = 187.9, 151.1 Hz, *C*FP), 98.37 (dd, *J* = 188.1, 150.8 Hz, *C*FP), 77.36 (COH), 62.75 (d, *J* = 6.7 Hz, O*C*H_2), 62.70 (d, *J* = 6.5 Hz, O*C*H_2), 58.94 (s, *C*HCH_3), 58.59 (*C*HCH_3), 51.33 (d, *J* = 6.3 Hz, *C*N), 50.73 (d, *J* = 6.3 Hz, *C*N), 21.45 (br s, *C*H_3), 20.88 (d, *J* = 20.8 Hz, *C*H_3), 20.89 (d, *J* = 20.8 Hz, *C*H_3), 16.98 (d, *J* = 6.2 Hz, OCH_2CH_3). ^{19}F NMR (283 MHz, $CDCl_3$) δ = -170.22 (dq, *J* = 73.5, 25.2 Hz, 1F), -171.54 (dq, *J* = 73.9, 25.1 Hz, 1F). ^{31}P {/^{1}H} NMR (122 MHz, CDCl3) δ = 15.48 (d, *J* = 71.4 Hz, 1P), 15.06 (d, *J* = 72.1 Hz, 1P). MS (ESI) calc for $C_{20}H_{28}FNO_4P^+$ 396.173, found 396.23.

rac Ethyl hydrogen ((2R,3S)-2-fluoro-3-hydroxy-4-(methyl((R)-1-phenylethyl)amino)-3-phenylbutan-2-yl)phosphonate **(rac 20a)** and ***ethyl hydrogen ((2S,3R)-2-fluoro-3-hydroxy-4-(methyl((R)-1-phenylethyl)amino)-3-phenylbutan-2-yl)phosphonate*** **(**rac **20b)** Procedure A ((*R*)-PhCH(Me)NH(Me), TEA), reaction time 72h, precipitating solid from oil (131 mg, 80%, 1:1 d.r.): ^{1}H

NMR (300 MHz, $CDCl_3$) δ 7.57 – 7.52 (m, 4H, Ph), 7.41 – 7.27 (m, 16H, Ph), 4.22 – 3.95 (m, 6H, OCH_2, OH), 3.48 (q, *J* = 7.0 Hz, 2H, C*H*CH_3), 2.95 – 2.86 (m, 2H, C*H*H), 2.56 – 2.52 (m, 2H, CH*H*), 2.38 (s, 6H, CH_3), 1.72 (d, *J* = 6.8 Hz, 6H, CH_3), 1.31 (t, *J* = 7.0 Hz, 3H, OCH_2CH_3), 1.21 (t, *J* = 7.0 Hz, 3H, OCH_2CH_3), 1.17 – 1.01 (m, 6H, CH_3). ^{13}C NMR (101 MHz, $CDCl_3$) δ 144.12 (d, *J* = 7.8 Hz), 142.65 (d, *J* = 8.9 Hz), 141.51, 140.52, 128.45, 128.39, 128.23, 127.92, 127.88, 127.60, 127.14, 126.98, 126.76 (11 x s, Ph), 98.95 (dd, *J* =, 180.5, 156.9 Hz, *C*FP), 98.78 (dd, *J* = 185.4, 154.1 Hz, *C*FP), 78.21 (s, *C*OH), 75.82 (s, *C*OH), , 62.34 (d, *J* = 6.5 Hz, OCH_2), 61.87 (d, *J* = 6.5 Hz, OCH_2), 58.19 (s, *C*HCH_3), 58.18 (s, *C*HCH_3), 57.68 (br d, *J* = 5.3 Hz, *C*N), 37.94 (s, CH_3), 36.68 (s, CH_3), 23.31 – 22.59 (m, CH_3), 20.82 (d, *J* = 20.5 Hz, CH_3), 20.11 (d, *J* = 21.0 Hz, CH_3), 16.67 (d, *J* = 6.7 Hz, OCH_2CH_3), 16.51 (d, *J* = 7.9 Hz, OCH_2CH_3). ^{19}F NMR (283 MHz, $CDCl_3$) δ -171.73 (ddt, *J* = 99.9, 50.3, 25.0 Hz), -172.94 (tt, *J* = 75.0, 25.2 Hz). ^{19}F NMR (283 MHz, $CDCl_3$) δ -170.12 (dq, *J* = 75.2, 25.1 Hz), -171.45 (dq, *J* = 75.9, 25.0 Hz). ^{31}P{/^{1}H} NMR (122 MHz, $CDCl_3$) δ 16.56 (dd, *J* = 75.3, 3.6 Hz), 16.35 (d, *J* = 74.3 Hz). MS (ESI) calc. for $C_{21}H_{30}FNO_4P$ 410.19, found 410.20 $[M+H]^+$.

rac Diethyl ((1R,2R)-2-((benzylamino)methyl)-1-fluoro-2-hydroxycyclohexyl)phosphonate **(**rac **22)** Procedure A ($BnNH_2$, TEA), slightly yellow oil (119 mg, 81%): ^{1}H NMR (400 MHz, $CDCl_3$): δ = 7.32 – 7.22 (m, 5H, Ph), 4.19 – 4.10 (m, 4H, OCH_2), 3.87 (d, *J* = 13.4 Hz, 1H, C*H*HPh), 3.84 (d, *J* = 13.5 Hz, 1H, CH*H*Ph), 3.45 (dd, *J* = 13.0, 1.6 Hz, 1H, C*H*H), 2.32 (d, *J* = 13.0 Hz, 1H, CH*H*), 2.25 – 2.11 (m, 1H, C*H*H), 2.09 – 2.00 (m, 1H, C*H*H), 1.75 – 1.68 (m, 2H, CH_2), 1.68 – 1.61 (m, 2H, CH_2), 1.60 – 1.52 (m, 2H, CH_2), 1.53 – 1.43 (m, 2H, CH_2), 1.31 (t, *J* = 7 Hz, 3H, OCH_2CH_3), 1.30 (t, *J* = 7 Hz, 3H, OCH_2CH_3). ^{13}C NMR (101 MHz, $CDCl_3$): δ = 140.28, 128.10, 127.1, 126.1 (4 x s, Ph), 96.14 (dd, *J* = 185.3, 165 Hz, *C*FP, C1), 71.24 (dd, *J* = 24.9, 2.1 Hz, *C*OH, C2), 63.80 (dd, *J* = 6.5 Hz, OCH_2), 62.2 (d, *J* = 6.0 Hz, OCH_2), 52.10– 52.04 (m, *C*N), 51.74 (s, CH_2Ph), 35.43 (d, *J* = 8.4 Hz, CH_2, C3), 28.67 (dd, *J* = 19.5, 2 Hz, CH_2, C6), 19.69 (s, CH_2, C4), 19.16 (dd, *J* = 9.1, 3.1 Hz, CH_2, C5), 15.4 (d, *J* = 5.7 Hz, OCH_2CH_3), 15.3 (d, *J* = 6.1 Hz, OCH_2CH_3). ^{19}F NMR (377 MHz, $CDCl_3$) δ = -181.26 (d, *J* = 86.3 Hz,). ^{31}P{/^{1}H} NMR (162 MHz, $CDCl_3$) δ = 20.53 (d, *J* = 88.6 Hz). GC–MS (EI) *m/z* = 354.2 $[M-F]^+$, t_R = 15.48 min.

rac Diethyl ((1R,2R)-1-fluoro-2-hydroxy-2-((((S)-1-phenylethyl)amino)methyl)cyclohexyl) phosphonate **(**rac **23a)** *and* ***diethyl ((1S,2S)-1-fluoro-2-hydroxy-2-((((S)-1-phenylethyl)amino)methyl)cyclohexyl)phosphonate*** **(**rac **23b**) Procedure A ((*S*)-PhCH(Me)NH_2, TEA), slightly yellow oil (121 mg, 78%, 1:1 d.r.): ^{1}H NMR (400 MHz, $CDCl_3$): δ = 7.29 – 7.18 (m, 10H, Ph), 4.16 – 4.09 (m, 8H, OCH_2), 3.80 (q, *J* = 7 Hz, 1H, C*H*CH_3), 3.69 (q, *J* = 7 Hz, 1H, C*H*CH_3), 3.33 (dd, *J* = 13.1, 1.7 Hz, 1H, C*H*H), 3.24 (dd, *J* = 13.1, 1.2 Hz, 1H, C*H*H), 2.13 (d, *J* = 13.0 Hz, CH_2, 1H, CH*H*), 2.07 (d, *J* = 13.0 Hz, CH_2, 1H, CH*H*), 2.10 – 1.99 (m, 4H, CH_2), 1.73 – 1.59 (m, 4H, CH_2), 1.56 – 1.50 (m, 4H, CH_2), 1.49 – 1.43 (m, 4H, CH_2), 1.38 (d, *J* = 7 Hz, 6H, CH_3), 1.34 – 1.27 (m, 12H, OCH_2CH_3). ^{13}C NMR (101 MHz, $CDCl_3$): δ = 145.58, 145.2.6, 128.47, 128.37, 126.79, 126.29 (6 x s, Ph), 97.77 (dd, *J* = 187.3,165.2 Hz, *C*FP, C1), 97.67 (dd, *J* = 185.4, 164.9 Hz, *C*FP, C1), 70.28 (dd, *J* = 20.0, 2 Hz, *C*OH, C2), 70.22 (dd, *J* = 19.9, 2.1 Hz, *C*OH, C2), 63.16 (dd, *J* =6.8, 2.0 Hz, OCH_2), 63.13 (dd, *J* = 7.1, 0.9 Hz, OCH_2CH_3), 62.74 (d, *J* = 7.2 Hz, OCH_2), 62.65 (d, *J* = 6.8 Hz, OCH_2), 59.30 (s, CH_3), 58.30 (s, CH_3), 52.75 (dd, *J* = 3.1, 1.3 Hz, *C*N), 52.05 (dd, *J* = 3.5, 2.3 Hz, *C*N), 34.70 (d, *J* = 8.9 Hz, CH_2 C3), 34.55 (d, *J* = 8.7 Hz, CH_2 C3), 29.10 (dd, *J* = 18.7 Hz, 2.1 Hz, CH_2, C6), 28.90 (dd, *J* = 18.5, 2.0 Hz, CH_2, C6), 24.46 (s, CH_3), 24.30 (s, CH_3), 20.53 (d, *J* = 1.0 Hz, CH_2, C4), 20.52 (d, *J* = 1.3 Hz, CH_2, C4), 19.76 (dd, *J* = 8.7, 2.7 Hz, CH_2, C5), 19.67 (dd, *J* = 9.0, 3.0 Hz, CH_2, C5), 16.46 (d, *J* = 5.6 Hz, OCH_2CH_3) 16.44 (d, *J* = 5.8 Hz, OCH_2CH_3), 16.43 (d, *J* = 5.6 Hz, OCH_2CH_3), 16.42 (d, *J* = 6.0 Hz, OCH_2CH_3). ^{19}F NMR (377 MHz, $CDCl_3$): δ = -181.0 – -

182.0 (m, 2F).$^{31}P\{/^1H\}$ NMR (162 MHz, $CDCl_3$): δ = 20.63 (d, *J* = 89.1 Hz), 20.61 (d, *J* = 90.0 Hz). GC–MS (EI) *m/z* = 388.2 $[M+H]^+$; t_R 18.8 min.

***rac* Diethyl ((1R,2R)-1-fluoro-2-hydroxy-2-((methyl((R)-1-phenylethyl)amino)methyl) cyclohexyl) phosphonate (*rac* 24a) and diethyl ((1S,2S)-1-fluoro-2-hydroxy-2-((methyl((R)-1-phenylethyl)amino)methyl)cyclohexyl)phosphonate (*rac* 24b)** Procedure A ((*R*)-PhCH(Me)NH(Me), TEA), slightly yellow oil (133 mg, 83% yield, 1:1 d.r.): ^1H NMR (400 MHz, $CDCl_3$): δ = 7.27 – 7.22 (m, 10H, Ph), 4.15 – 4.04 (m, 8H, OCH_2), 3.98 (s, 1H, OH), 3.96 (s, 1H, OH), 3.81 (q, *J* = 7.2 Hz, 1H, C*H*CH_3), 3.64 (q, *J* = 7.2 Hz, 1H, C*H*CH_3), 3.49 (d, *J* = 13.4 Hz, 1H, C*H*H), 3.29 (d, *J* = 13.7 Hz, 1H, C*H*H), 2.43 (d, *J* = 15.7 Hz, 1H, CH*H*), 2.39 (d, *J* = 14.8 Hz, 1H, CH*H*), 2.21 (d, *J* = 6.7 Hz, 6H, CH_3), 2.19 (s, 6H, CH_3), 2.05 – 1.85 (m, 8H, CH_2), 1.63 – 1.40 (m, 12H, CH_2), 1.33 (d, *J* = 6.6 Hz, 6H, CH_3), 1.29 – 1.23 (m, 12H, OCH_2CH_3). ^{13}C NMR (101 MHz, $CDCl_3$): δ = 141.79, 141.45, 127.48 (3 x s, Ph), 127.02 (d, *J* = 2.0 Hz, Ph), 126.93 (d, *J* = 1.9 Hz, Ph), 126.61 (s, Ph), 126.19 (s, Ph), 96.98 (dd, *J* = 196.6, 155.2 Hz, *C*FP, C1), 97.00 (dd, *J* = 196.5, 156.0 Hz, *C*FP, C1), 70.93 (dd, *J* = 22.7, 1.2 Hz, *C*OH, C2), 70.27 (dd, *J* = 23, 1 Hz, *C*OH, C2), 62.65 (s, CH_3), 61.98 (s, CH_3), 61.87 (d, *J* =6.5 Hz, OCH_2), 61.78 (d, *J* = 6.3 Hz, OCH_2), 61.74 (d, *J* = 7.0 Hz, OCH_2), 61.73 (d, *J* = 7.8 Hz, OCH_2), 58.63 (br s, *C*N), 58.36 (br s, *C*N), 38.39 (s, CH_3), 38.14 (s, CH_3), 34.18 (dd, *J* = 7.7 Hz, 1 Hz, CH_2, C3), 33.23 (dd, *J* = 7.3 Hz, 0.9 Hz, CH_2, C3), 28.83 (dd, *J* = 20.0 Hz, 2.1 Hz, CH_2, C6), 28.56 (dd, *J* = 19.5 Hz, 2.7 Hz, CH_2, C6), 22.30 (s, 2 x CH_3), 19.57 (d, *J* =0.8 Hz, CH_2, C4), 19.43 (d, *J* =0.6 Hz, CH_2, C4), 19.21 (br s, CH_2, C5), 19.15 (br s, CH_2, C5), 15.48 (d, *J* = 5.6 Hz, OCH_2CH_3), 15.44 (d, *J* = 5.5 Hz, OCH_2CH_3), 15.43 (d, *J* = 5.6 Hz, OCH_2CH_3), 15.41 (d, *J* = 5.6 Hz, OCH_2CH_3). ^{19}F NMR (377 MHz, $CDCl_3$): δ = -180.45 (br s). $^{31}P\{/^1H\}$ NMR (162 MHz, $CDCl_3$): δ = 20.11 (d, *J* = 85.8 Hz). GC–MS *m/z* = 402 $[M+H]^+$; R_t 19.2 min., *m/z* = 402 $[M+H]^+$; t_R =19.3 min.

3.1. General procedure (procedure C) for hydrogenation and N-Boc protection of γ-amino-β-hydroxyphosphonates.

A solution of *N*,*N*-dibenzyl protected hydroxyphosphonate (0.3 mmol) in EtOH (2 mL) containing Boc_2O (98 mg, 0.45 mmol) was hydrogenated over 10% Pd-C (30 mg) under atmospheric pressure for 48h. Then, the catalyst was filtrated through Celite with MeOH, the solution was concentrated on vacuum, and purified by flash column chromatography $CHCl_3$→5% MeOH/$CHCl_3$ (v:v), (1 cm layer of silica gel) to give appropriate *N*-Boc protected amino hydroxyphosphonate.

3.2. General procedure (procedure D) for hydrogenation of monoesters of hydroxyalkylphosphonic acids.

A solution of monoester of *N*,*N*-dibenzyl protected or azido- hydroxyphosphonic acid **17** or **38** (0.3 mmol) in absolute EtOH (2 mL) was hydrogenated over 10% Pd-C (30 mg) under atmospheric pressure for 48h. Then, the catalyst was filtrated through Celite with MeOH, the solution was concentrated on vacuum, and purified by flash column chromatography $CHCl_3$→5% MeOH/$CHCl_3$ (v:v) (1 cm layer of silica gel) to give monoester of amino hydroxyphosphonic acid **27**.

3.3. Spectroscopic properties of compounds **26-28** *(procedures C,D)*

***rac* Tert-butyl ((2R,3R)-3-(diethoxyphosphoryl)-3-fluoro-2-hydroxy-2-methylpropyl)carbamate** (rac **26a)** Procedure C, major isomer. Isolated as a mixture with **26b**, which could not be separated by the chromatography techniques employed in this study transparent oil (88 mg, 85%, 4:1 d.r.): ^1H NMR (300 MHz, $CDCl_3$) δ = 5.18 (br s, 1H, N*H*), 4.63 (dd, *J* = 45.1, 5.5 Hz, 1H, C*H*FP), 4.29 – 4.19 (m,

3H, OCH_2), 4.11 (dq, *J* = 7.9, 7.1 Hz, 1H, OCH*H*), 3.51 – 3.39 (m, 1H, C*H*HN), 3.41 – 3.33 (m, 1H, CH*H*N), 1.45 (s, 9H, $C(CH_3)_3$), 1.42 – 1.34 (m, 9H, CH_3, 2 x OCH_2CH_3). ^{13}C NMR (101 MHz, $CDCl_3$) δ = 157.20 (s, *C*O), 91.87 (dd, *J* = 188.9, 161.8 Hz, *C*FP), 79.87 (s, $C(CH_3)_3$), 73.59 (d, *J* = 18.3 Hz, *C*OH), 64.25 (d, *J* = 7.1 Hz, OCH_2), 63.08 (d, *J* = 6.9 Hz, OCH_2), 47.22 – 46.91 (m, *C*N), 28.45 (s, $C(CH_3)_3$), 21.97 (t, *J* = 4.1 Hz, CH_3), 16.49 (d, *J* = 5.5 Hz, OCH_2CH_3), 16.47 (d, *J* = 5.5 Hz, OCH_2CH_3).^{19}F NMR (283 MHz, $CDCl_3$) δ = -215.57 (dd, *J* = 78.4, 45.2 Hz). ^{31}P{/^{1}H} NMR (122 MHz, $CDCl_3$) δ = 17.06 (d, *J* = 76.3 Hz). IR (film): ν = 3349, 2980, 2933, 1712, 1518, 1392, 1367, 1246, 1167, 1029, 974 cm $^{-1}$. MS (EI) *m*/*z* = 343.5 $[M]^+$.

rac Tert-butyl ((2S,3R)-3-(diethoxyphosphoryl)-3-fluoro-2-hydroxy-2-methylpropyl)carbamate (rac **26b**) minor isomer: ^{1}H NMR (300 MHz, $CDCl_3$) δ = 5.18 (br s, 1H, N*H*), 4.42 (dd, *J* = 45.8, 7.8 Hz, 1H, C*H*FP), 4.29 – 4.19 (m, 4H, OCH_2), 3.41 – 3.33 (m, 1H, C*H*HN), 3.20 – 3.10 (m, 1H, CH*H*N), 1.45 (s, 9H, $C(CH_3)_3$), 1.42 – 1.34 (m, 9H, CH_3, OCH_2CH_3). ^{19}F NMR (283 MHz, $CDCl_3$) δ = -212.13 (dd, *J* = 69.4, 45.3 Hz). ^{31}P{/^{1}H} NMR (122 MHz, $CDCl_3$) δ = 17.81 (d, *J* = 71.7 Hz).
rac Ethyl ((2R,3S)-4-ammonio-2-fluoro-3-hydroxy-3-phenylbutan-2-yl)phosphonate (rac **27**) Procedure D. white solid (72 mg, 82%). Alternatively, compound rac **27** can be obtain from rac **38** by procedure D (65 mg, 75%): ^{1}H NMR (300 MHz, CD_3OD) δ = 7.60 (d, *J* = 7.4 Hz, 2H, Ph), 7.50 – 7.36 (m, 3H, Ph), 4.11 – 4.05 (m, 3H, OCH_2, OH), 4.01 (br d, *J* = 13.5 Hz, 1H, C*H*H), 3.61 (br d, *J* = 12.9 Hz, 1H, CH*H*), 1.28 (t, *J* = 7.1 Hz, 3H, OCH_2CH_3), 1.18 (d, *J* = 25.0, 11.8 Hz, 3H, CH_3). ^{13}C NMR (101 MHz, CD_3OD) δ = 139.28 (d, *J* = 9.7 Hz, Ph), 130.20 (s, Ph), 129.37 (d, *J* = 3.6 Hz, Ph), 128.36 (d, *J* = 3.2 Hz, Ph), 98.29 (dd, *J* = 189.7, 152.7 Hz, *C*FP), 77.25 (dd, *J* = 20.4, 4.3 Hz, *C*(OH)), 63.55 (d, *J* = 6.1 Hz, OCH_2), 47.10 (s, *C*N) 19.52 (dd, *J* = 21.3, 3.3 Hz, CH_3), 17.20 (d, *J* = 5.6 Hz, OCH_2CH_3). ^{19}F NMR (283 MHz, CD_3OD) δ = -171.21 (dq, *J* = 75.1, 25.1 Hz). ^{31}P{/^{1}H} NMR (122 MHz, CD_3OD) δ = 16.39 (d, *J* = 75.2 Hz). IR (film): ν = 3360 (br), 3164, 2982, 2930, 1524, 1496, 1451, 1391, 1370, 1212, 1198, 1061, 951 cm $^{-1}$. MS (ESI) *m*/*z* = 292.3 $[M+H]^+$
rac Tert-butyl (((1R,2R)-2-(diethoxyphosphoryl)-2-fluoro-1-hydroxycyclohexyl)methyl) carbamate (rac **28**) Procedure C, white solid (99 mg, 86%): ^{1}H NMR (300 MHz, $CDCl_3$) δ = 5.33 (s, 1H, OH), 4.27 – 4.15 (m, 4H, OCH_2), 3.49 – 3.45 (m, 2H, CH_2N), 2.25 – 2.12 (m, 1H, C*H*H), 2.09 – 1.99 (m, 1H, C*H*H), 1.76 – 1.64 (m, 3H, CH*H*,CH_2), 1.61 – 1.51 (m, 3H, CH_2), 1.42 (s, 9H, $C(CH_3)_3$), 1.36 (t, *J* = 7.1 Hz, 6H, OCH_2CH_3). ^{13}C NMR (101 MHz, $CDCl_3$) δ = 157.30 (s, *C*O), 96.62 (dd, *J* = 189.7, 161.7 Hz, *C*FP, C2), 79.44 (s, $C(CH_3)_3$), 73.32 (d, *J* = 21.6 Hz, *C*OH, C1), 66.94 – 61.01 (m, OCH_2), 47.00 (s, *C*N), 30.94 (d, *J* = 8.7 Hz, C6), 28.54 (s, $C(CH_3)_3$), 19.81 (d, *J* = 7.2 Hz, C4), 19.77 (s, C5), 16.56 (d, *J* = 5.7 Hz, OCH_2CH_3), 16.55 (d, *J* = 5.7 Hz, OCH_2CH_3). ^{19}F NMR (283 MHz, $CDCl_3$) δ = -181.62 (ddd, *J* = 85.2, 41.3, 13.3 Hz). ^{31}P{/^{1}H} NMR (122 MHz, $CDCl_3$) δ = 20.69 (d, *J* = 83.7 Hz). IR (film): ν = 3350, 2977, 2935, 2868, 1712, 1517, 1393, 1366, 1246, 1170, 1026, 973 cm $^{-1}$. MS (EI) *m*/*z* = 383.3 $[M]^+$.

4.1. General procedure (procedure F) for oxiran **5-8** *opening by HBr:*

To the dissolved in chloroform (1.5 mL) acethyl bromide (44 μL, 74 mg, 0.6 mmol) methanol (24 μL, 19 mg, 0.6 mmol) was added at 0°C and reaction mixture was stirred for 20 min. Then, oxirane **5-8** (0.5 mmol) in chloroform (1.5 mL) was added, and stirring was continued at 0°C for 2h. Next, crude reaction mixture was extracted with CH_2Cl_2 ($NaHCO_{3\ aq}$/brine), dried with anhydrous Na_2SO_4 and evaporated to give bromohydrine **32-35**. Flash column chromatography $CHCl_3$→5% MeOH/$CHCl_3$ (v:v), (1 cm layer of silica gel) gave compounds with lower yields.

4.2. Spectroscopic properties of compounds **32-35**

***rac* Diethyl ((1R,2S)-2-bromo-1-fluoro-3-hydroxy-2-methylpropyl)phosphonate** (rac **32a)**, major isomer, isolated as a mixture with **32b**, which could not be separated by the chromatography techniques employed in this study; transparent oil (crude: 141 mg, 92%, 4:1 dr). Additional column chromatography gave **32a/32b** (88 mg, 57%, 4:1, d.r.): ^{1}H NMR (400 MHz, $CDCl_3$) δ = 4.85 (dd, *J* = 44.9, 5.9 Hz, 1H, C*H*F), 4.31 – 4.16 (m, 4H, OC*H*$_2$), 3.92 (s, 1H, OH), 3.60 (d, *J* = 10.4 Hz, 1H, C*H*H), 3.49 (dt, *J* = 10.4, 2.1 Hz, 1H, CH*H*), 1.50 (d, *J* = 2.0 Hz, 3H, C*H*$_3$), 1.37 (t, *J* = 7.1 Hz, 3H, OCH_2CH_3), 1.36 (t, *J* = 7.1 Hz, 3H, OCH_2CH_3). ^{13}C NMR (101 MHz, $CDCl_3$) δ = 89.99 (dd, *J* = 189.1, 164.4 Hz, *C*FP), 72.72 (dd, *J* = 18.2, 2.7 Hz, *C*Br), 64.39 (dd, *J* = 6.8, 1.9 Hz, O*C*H$_2$), 62.90 (d, *J* = 7.0 Hz, O*C*H$_2$), 37.94 (dd, *J* = 9.1, 6.7 Hz, *C*OH), 22.80 (dd, *J* = 3.4, 2.3 Hz, *C*H$_3$), 16.37 (d, *J* = 6.3 Hz, OCH_2CH_3). 16.31 (d, *J* = 6.3 Hz, OCH_2CH_3). ^{19}F NMR (376 MHz, $CDCl_3$) δ = -214.59 (ddq, *J* = 78.1, 45.0, 1.4 Hz). ^{31}P{/^{1}H} NMR (162 MHz, $CDCl_3$) δ = 16.04 (d, *J* = 78.0 Hz). IR (film): ν = 3348, 2984, 2929, 1243, 1163, 1019, 973, 544 cm $^{-1}$; GC–MS *m/z* = 307.0/309.1 $[M+H]^+$;t_R = 13.23/13.35 min;

***rac* Diethyl ((1R,2R)-2-bromo-1-fluoro-3-hydroxy-2-methylpropyl)phosphonate** (rac **32b)**, minor isomer: ^{1}H NMR (400 MHz, $CDCl_3$) δ = 4.90 (dd, *J* = 44.9, 4.5 Hz, 1H, C*H*F), 4.31 – 4.16 (m, 4H, OC*H*$_2$), 3.90 (s, 1H, OH), 3.56 (d, *J* = 1.5 Hz, 2H, C*H*2), 1.52 (d, *J* = 2.2 Hz, 3H, C*H*$_3$), 1.37 (t, *J* = 7.1 Hz, 3H, OCH_2CH_3), 1.36 (t, *J* = 7.1 Hz, 3H, OCH_2CH_3). ^{13}C NMR (101 MHz, $CDCl_3$) δ = 89.23 (dd, *J* = 192.2, 162.8 Hz, *C*FP), 71.69 (dd, *J* = 19.6, 2.9 Hz, *C*Br), 63.63 (d, *J* = 5.8 Hz, OCH_2CH_3), 63.22 (d, *J* = 6.8 Hz, OCH_2CH_3), 39.42 (dd, *J* = 9.9, 3.0 Hz, *C*OH), 21.92 (dd, *J* = 4.6, 2.5 Hz, *C*H$_3$), 16.31 (d, *J* = 6.3 Hz, OCH_2CH_3), 16.10 (d, *J* = 6.7 Hz, OCH_2CH_3). ^{19}F NMR (376 MHz, $CDCl_3$) δ = -212.45 (ddq, *J* = 72.6, 44.9, 1.2 Hz).^{31}P{/^{1}H} NMR (162 MHz, $CDCl_3$) δ = 16.29 (d, *J* = 72.7 Hz).

***rac* Diethyl ((1R,2S)-2-bromo-1-fluoro-3-hydroxy-2-phenylpropyl)phosphonate** (rac **33)** slightly yellow oil (slowly decomposing on air), (159 mg, 86%): ^{1}H NMR (300 MHz, $CDCl_3$) δ = 7.47 – 7.36 (m, 5H, Ph), 5.12 (dd, *J* = 45.1, 5.8 Hz, 1H, C*H*F), 4.20 (q, *J* =7.1 Hz, 2H, OC*H*$_2$), 3.93 (dd, *J* = 10.8, 1.1 Hz, 1H, C*H*H), 3.85 (d, *J* = 10.5 Hz, 1H, CH*H*), 3.76 (q, *J* = 7.5 Hz, 1H, OC*H*H), 3.44 (q, *J* = 7.5 Hz, 1H, OCH*H*), 1.33 (t, *J* = 6.8 Hz, 3H, OCH_2CH_3), 0.97 (t, *J* = 6.9 Hz, 3H, OCH_2CH_3). ^{13}C NMR (75 MHz, $CDCl_3$) δ = 138.96 (d, *J* = 2.0 Hz, Ph), 128.32, 128.20, 126.28 (3 x s, Ph), 90.19 (dd, *J* = 192.7, 164.4 Hz, *C*FP), 76.03 (d, *J* = 19.0 Hz, *C*Br), 64.45 (d, *J* = 5.1 Hz, O*C*H$_2$), 62.79 (d, *J* = 6.1 Hz, O*C*H$_2$), 39.07 (dd, *J* = 11.8, 5.3 Hz, *C*OH), 16.35 (dd, *J* = 4.9 Hz, OCH_2CH_3), 15.87 (dd, *J* = 5.4 Hz, OCH_2CH_3). ^{19}F NMR (282 MHz, $CDCl_3$) δ = -213.44 (dd, *J* = 81.3, 45.3 Hz). ^{31}P{/^{1}H} NMR (121 MHz, $CDCl_3$) δ = 15.84 (d, *J* = 81.2 Hz). MS (EI) *m/z* = 369.1/371.1 $[M+H]^+$.

***rac* Diethyl ((2R,3R)-3-bromo-2-fluoro-4-hydroxy-3-phenylbutan-2-yl)phosphonate** (rac **34)** transparent oil (180 mg, 94%):^{1}H NMR (600 MHz, $CDCl_3$) δ = 7.49 (d, *J* = 7.6 Hz, 2H, Ph), 7.40 (t, *J* = 7.6 Hz, 2H, Ph), 7.35 (t, *J* = 7.3 Hz, 1H, Ph), 4.66 (d, *J* = 11.0 Hz, 1H, C*H*H), 4.13 – 4.30 (m, 4H, OC*H*$_2$), 4.21 (dd, *J* = 11.1, 3.1 Hz, 1H, CH*H*), 4.06 (s, 1H, OH), 1.39 (t, *J* = 7.1 Hz, 3H, OCH_2CH_3), 1.34 (dd, *J* = 25.1, 13.9 Hz, 3H, C*H*$_3$), 1.33 (dd, *J* = 7.1, 0.5 Hz, 3H, OCH_2CH_3). ^{13}C NMR (151 MHz, $CDCl_3$) δ = 139.22 (d, *J* = 9.8 Hz, Ph), 127.97 (s, Ph), 127.94 (s, Ph), 126.67 (d, *J* = 2.3 Hz, Ph), 97.84 (dd, *J* = 191.2, 163.6 Hz, *C*FP), 78.29 (dd, *J* = 21.5, 4.2 Hz, *C*Br), 63.93 (dd, *J* = 7.1, 2.1 Hz, O*C*H$_2$), 63.71 (d, *J* = 6.9 Hz, O*C*H$_2$), 40.95 (dd, *J* = 4.3, 1.3 Hz, *C*OH), 19.99 (dd, *J* = 21.2, 3.1 Hz, *C*H$_3$), 16.45 (d, *J* = 5.7 Hz, OCH_2CH_3), 16.36 (d, *J* = 5.8 Hz, OCH_2CH_3). ^{19}F NMR (565 MHz, $CDCl_3$) δ = -171.15 (dq, *J* = 83.8, 25.1 Hz). ^{31}P{/^{1}H} (242 MHz, $CDCl_3$) δ = 19.36 (d, *J* = 83.9 Hz). MS (EI) *m/z* =368.1/370.1 $[M-Me]^+$

***rac* Diethyl ((1R,2S)-2-bromo-1-fluoro-2-(hydroxymethyl)cyclohexyl)phosphonate** (rac **35)** Slightly yellow oil (158 mg, 91%):^{1}H NMR (600 MHz, $CDCl_3$) δ = 4.26 – 4.18 (m, 4H, OC*H*$_2$), 4.12 (d, *J* = 11.0 Hz, 1H, C*H*HOH), 3.65 (dd, *J* = 11.0, 2.7 Hz, 1H, CH*H*OH), 3.62 (s, 1H, OH), 2.26 – 2.18 (m,

1H, C*H*H, C6H), 2.16 – 2.11 (m, 1H CH*H*, C3H), 2.09 – 2.03 (m, 1H, C*H*H, C6H), 1.71 – 1.65 (m, 1H, C*H*H, C4H), 1.61 – 1.50 (m, CH_2, 4H, C3&C4H&C5H_2), 1.38 (t, *J* = 7.1 Hz, 6H, OCH_2CH_3). ^{13}C NMR (151 MHz, $CDCl_3$) δ = 96.6 (dd, *J* = 193.5, 161.3 Hz, *C*FP, C1), 72.0 (dd, *J* =21.5, 2.4 Hz, *C*Br, C2), 64.0 (d, *J* = 7.1, O*C*H_2), 63.9 (dd, *J* = 7.9, 1.8 Hz, O*C*H_2), 41.9 (s, *C*H_2OH), 31.5 (d, *J* = 8.0 Hz, *C*H_2, *C*3), 29.1 (dd, *J* = 20.4, 2.8 Hz, *C*H_2, *C*6), 19.7 (s, *C*H_2, C4), 19.6 (dd, *J* = 9.8, 2.7 Hz, *C*H_2, C5), 16.41 (d, *J* = 5.9 Hz, OCH_2*C*H_3), 16.40 (d, *J* = 5.9 Hz, OCH_2*C*H_3). ^{19}F NMR (565 MHz, $CDCl_3$) δ = -179.50 (dd, *J* = 78.9, 43.3 Hz). ^{31}P{/^{1}H} NMR (242 MHz, $CDCl_3$) δ = 19.31 (d, *J* = 80.7 Hz). GC–MS *m*/*z* = 347.3/349.3 $[M+H]^+$; R_t 15.9 min.

5.1. General procedure (procedure I) for hydrogenation and N-Boc protection of β-azido-γ-hydroxyphosphonates.

General procedure (procedure I) for hydrogenation and N-Boc protection of β-azido-γ-hydroxyphosphonates. A solution of azidohydroxyphosphonate **36-37, 39** (0.3 mmol) in absolute EtOH (2 mL) containing Boc_2O (98 mg, 0.45 mmol) was hydrogenated over 10% Pd-C (30 mg) under atmospheric pressure for 48h. Then, the catalyst was filtrated through Celite with MeOH, the solution was concentrated on vacuum, and purified by flash column chromatography $CHCl_3$→5% MeOH/$CHCl_3$ (v:v), (1 cm layer of silica gel) to give appropriate *N*-Boc protected amino hydroxyphosphonate.

5.2. Spectroscopic properties of compounds **40-42**

rac Tert-butyl ((1R,2S)-1-(diethoxyphosphoryl)-1-fluoro-3-hydroxy-2-methylpropan-2-yl)carbamate **(**rac **40a)** Procedure I, major isomer. Isolated as a mixture with **40b**, which could not be separated by the chromatography techniques employed in this study, transparent oil (82 mg, 80%, 3:1 d.r.): ^{1}H NMR (400 MHz, $CDCl_3$) δ = 4.61 (dd, *J* = 45.1, 5.3 Hz, 1H, C*H*F), 4.30 – 4.16 (m, 4H, OCH_2), 3.43 (dd, *J* = 15.5, 7.3 Hz, 1H, C*H*H) 3.36 (dd, *J* = 14.3, 5.0 Hz, 1H, CH*H*), 1.44 (s, 9H, C(CH_3)$_3$), 1.42 – 1.31 (m, 9H, CH_3, 2 x OCH_2CH_3). ^{13}C NMR (101 MHz, $CDCl_3$) δ = 157.21 (s, *C*O), 91.86 (dd, *J* = 189.0, 162.0 Hz, *C*FP), 79.93 (s, *C*(CH_3)$_3$), 73.60 (d, *J* = 18.2 Hz, *C*OH), 64.31 (d, *J* = 7.2 Hz, O*C*H_2), 63.12 (d, *J* = 7.0 Hz, O*C*H_2), 47.13 (*C*N), 28.48 (s, C(*C*H_3)$_3$), 22.04 (t, *J* = 4.1 Hz, *C*H_3), 16.52 (d, *J* = 5.4 Hz, OCH_2*C*H_3), 16.51 (d, *J* = 5.4 Hz, OCH_2*C*H_3). ^{19}F NMR (283 MHz, $CDCl_3$) δ = -215.52 (dd, *J* = 76.3, 45.2 Hz). ^{31}P{/^{1}H} NMR (122 MHz, $CDCl_3$) δ = 16.60 (d, *J* = 76.5 Hz). IR (film): ν = 3395, 2982, 2932, 1714, 1518, 1393, 1366, 1249, 1166, 1026, 964 cm $^{-1}$. MS (EI) *m*/*z* = 343.6 $[M]^+$.

rac Tert-butyl ((1R,2R)-1-(diethoxyphosphoryl)-1-fluoro-3-hydroxy-2-methylpropan-2-yl)carbamate **(**rac **40b)**, minor isomer: ^{1}H NMR (400 MHz, $CDCl_3$) δ = 4.70 (dd, *J* = 45.4, 6.7 Hz, 1H, C*H*F), 4.30 – 4.16 (m, 4H, OCH_2), 3.29 – 3.23 (m, 1H, C*H*H), 3.18 (ddd, *J* = 11.2, 4.1, 2.1 Hz, 1H, CH*H*), 1.47 (s, 9H, C(CH_3)$_3$), 1.42 – 1.31 (m, 9H, CH_3, 2 xOCH_2CH_3). ^{19}F NMR (283 MHz, $CDCl_3$) δ = -211.01 (dd, *J* = 71.4, 45.0 Hz). ^{31}P{/^{1}H} NMR (122 MHz, $CDCl_3$) δ = 17.36 (d, *J* = 71.7 Hz).

rac Tert-butyl ((1R,2S)-1-(diethoxyphosphoryl)-1-fluoro-3-hydroxy-2-phenylpropan-2-yl)carbamate **(**rac **41)** Procedure I. white solid (100 mg, 82%): ^{1}H NMR (300 MHz, $CDCl_3$) δ = 7.54 – 7.46 (m, 2H, Ph), 7.42 – 7.30 (m, 2H, Ph), 7.33 – 7.27 (m, 1H, Ph), 5.10 (s, 1H, OH), 4.98 (dd, *J* = 45.5, 6.2 Hz, 1H, C*H*F), 4.19 (quintet, *J* = 7.2 Hz, 2H, OCH_2), 3.75 (“br d”, *J* = 6.4 Hz, 2H, CH_2OH), 3.73 – 3.66 (m, 1H, OC*H*H), 3.39 (q, *J* = 8.0 Hz, 1H, OCH*H*), 1.31 (td, *J* = 7.1, 0.7 Hz, 3H,

OCH_2CH_3), 1.28 (s, 9H, $C(CH_3)_3$), 0.94 (t, *J* = 7.1 Hz, 3H, OCH_2CH_3). ^{13}C NMR (101 MHz, $CDCl_3$) δ = 156.51 (s, *C*O), 139.14, 128.30, 128.05, 126.52 (4 x s, Ph), 91.19 (dd, *J* = 192.6, 165.2 Hz, *C*FP), 79.47 (s, $C(CH_3)_3$), 77.16 (d, *J* = 19.3 Hz, $CHCl_3$, *C*OH), 64.41 (d, *J* = 7.0 Hz, OCH_2), 62.59 (d, *J* = 6.7 Hz, OCH_2), 47.64 (dd, *J* = 11.3, 5.2 Hz, *C*N), 28.31 (s, $C(CH_3)_3$), 16.51 (d, *J* = 5.9 Hz, OCH_2CH_3), 16.02 (d, *J* = 6.0 Hz, OCH_2CH_3). ^{13}C NMR (101 MHz, CD_3OD) δ = 158.72 (s, *C*O), 140.82, 129.03, 128.69, 127.63 (4 x s, Ph), 93.41 (dd, *J* = 189.0, 168.7 Hz, *C*FP), 80.35 (s, $C(CH_3)_3$), 78.29 (d, *J* = 18.2 Hz, *C*N), 64.70 (d, *J* = 6.9 Hz, OCH_2), 64.07 (d, *J* = 6.8 Hz, OCH_2), 28.59 (s, $C(CH_3)_3$), 16.50 (d, *J* = 6.0 Hz, OCH_2CH_3), 16.48 (d, *J* = 6.0 Hz, OCH_2CH_3). ^{19}F NMR (283 MHz, $CDCl_3$) δ = -213.80 (dd, *J* = 82.0, 45.6 Hz). $^{31}P\{/^1H\}$ NMR (122 MHz, $CDCl_3$) δ = 16.11 (d, *J* = 82.1 Hz). IR (film): ν = 3377, 2979, 2925, 2854, 1712, 1513, 1450, 1392, 1367, 1247, 1168, 1030, 977 cm^{-1}. MS (EI) *m/z* = 405.5 $[M]^+$.

rac Tert-butyl ((1S,2R)-2-(diethoxyphosphoryl)-2-fluoro-1-(hydroxymethyl)cyclohexyl) carbamate **(**rac **42)** Procedure I, precipitating solid from oil (92 mg, 80%): ^1H NMR (300 MHz, $CDCl_3$) δ 4.37 – 4.15 (m, 4H, OCH_2), 3.48 (d, *J* = 5.9 Hz, 2H, CH_2N), 2.15 – 1.98 (m, 2H, C*H*H), 1.76 – 1.50 (m, 6H, HC*H*, CH_2), 1.44 (s, 9H, $C(CH_3)_3$), 1.38 (t, *J* = 7.1 Hz, 3H, OCH_2CH_3), 1.37 (t, *J* = 7.1 Hz, 3H, OCH_2CH_3). ^{13}C NMR (101 MHz, $CDCl_3$) δ 156.27 (s, *C*O), 95.55 (dd, *J* = 189.6, 161.5 Hz, *C*FP, C2), 78.44 (s, $C(CH_3)_3$), 72.29 (br d, *J* = 21.4 Hz, *C*OH, C1), 62.89 (dd, *J* = 7.5, 2.0 Hz, OCH_2), 62.77 (d, *J* = 6.8 Hz, OCH_2), 45.94 (s, *C*N), 29.85 (d, *J* = 8.6 Hz, C6), 27.56 (dd, *J* = 20.2, 2.5 Hz, C3), 27.51 (s, $C(CH_3)_3$), 20.20 – 17.32 (m, C4,5), 15.58 (d, *J* = 5.8 Hz, OCH_2CH_3), 15.49 (d, *J* = 5.8 Hz, OCH_2CH_3). ^{19}F NMR (283 MHz, $CDCl_3$) δ -181.58 (ddd, *J* = 83.6, 40.1, 12.4 Hz). $^{31}P\{/^1H\}$ NMR (122 MHz, $CDCl_3$) δ 20.12 (d, *J* = 83.8 Hz). IR (film): ν = 3353, 2978, 2936, 2869, 1713, 1680, 1517, 1449, 1392, 1366, 1245, 1169, 1025, 974 cm^{-1}. MS (EI) *m/z* = 383.6 $[M]^+$.

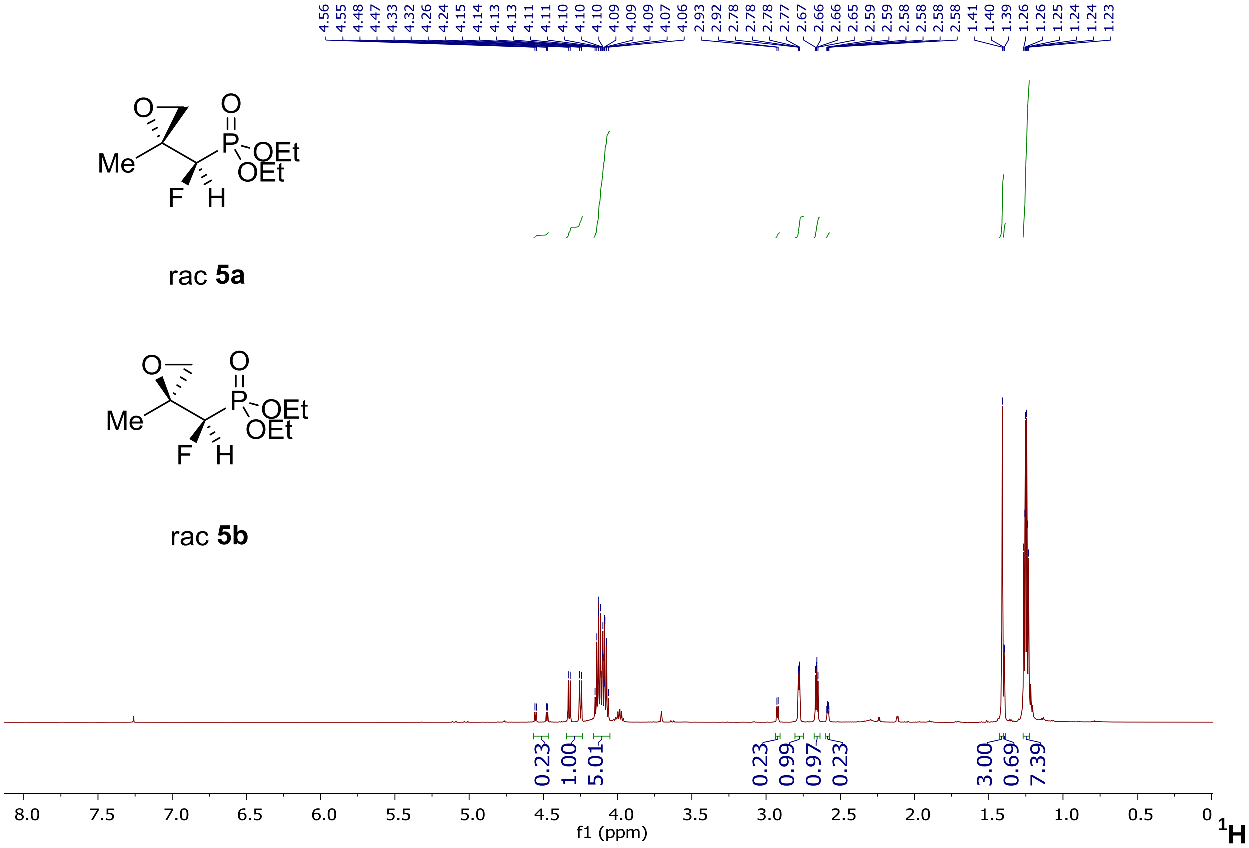

NMR of rac **5a,b** (4.3:1, d.r.)

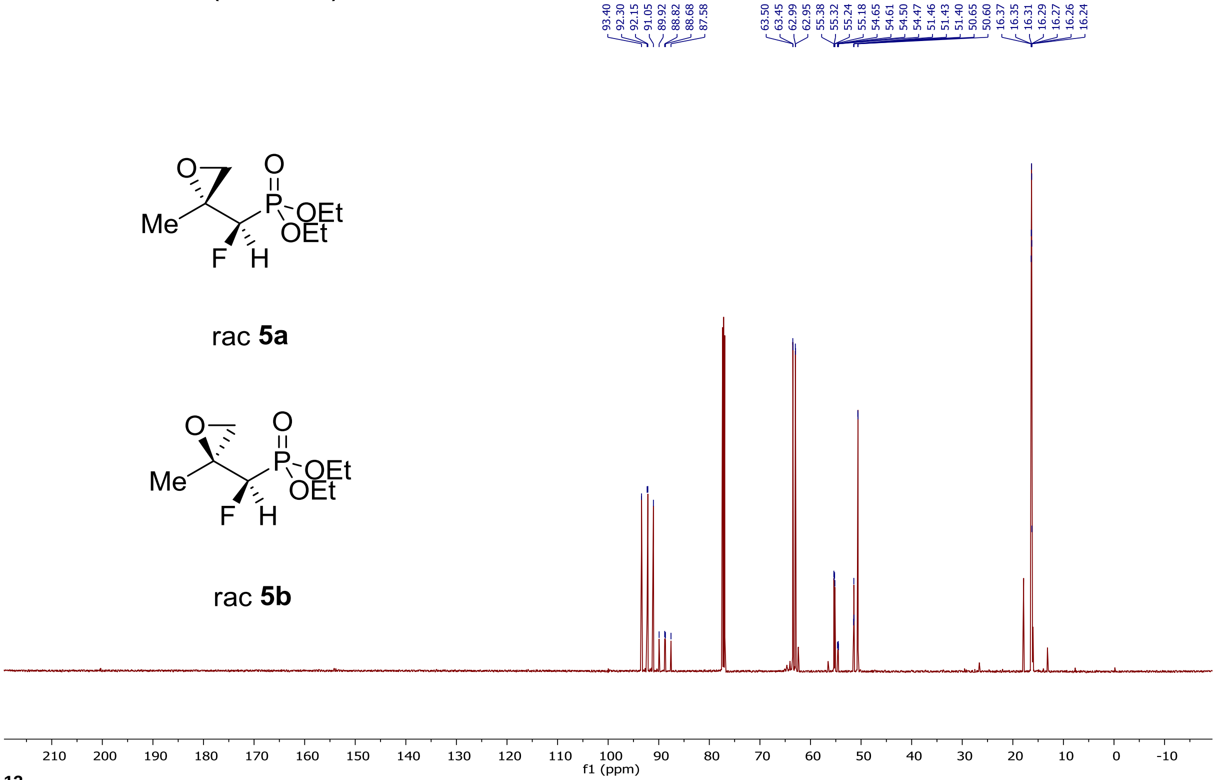

^{13}C NMR of rac 5a,b (4.3:1, d.r.)

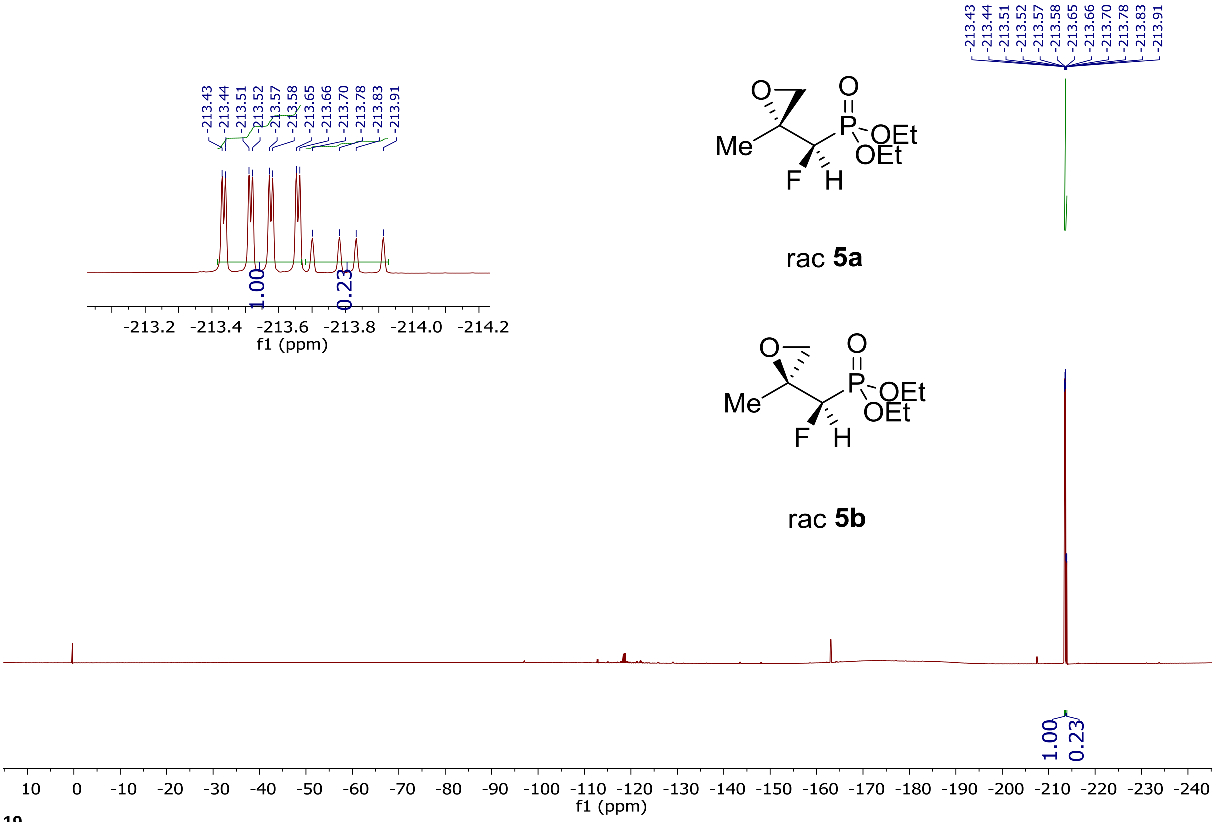

^{19}F NMR of rac **5a,b** (4.3:1, d.r.)

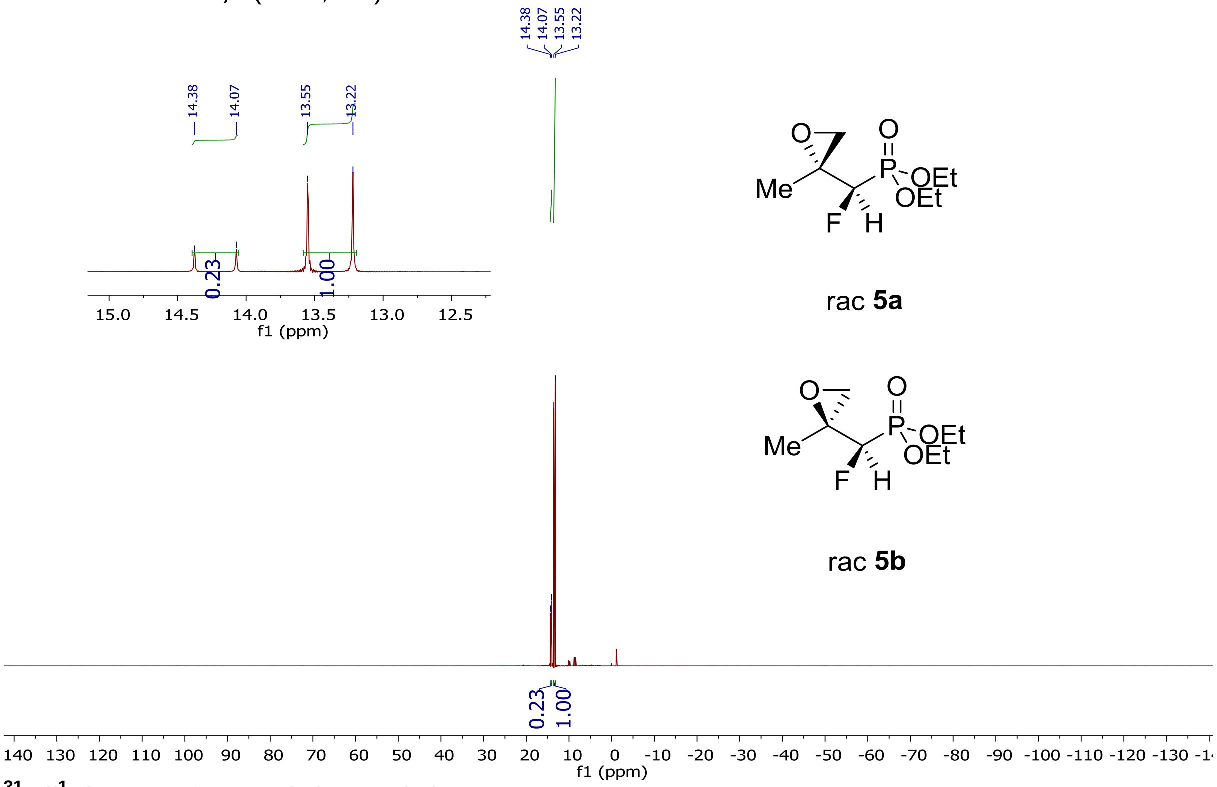

^{31}P{/^{1}H} NMR of rac **5a,b** (4.3:1, d.r.)

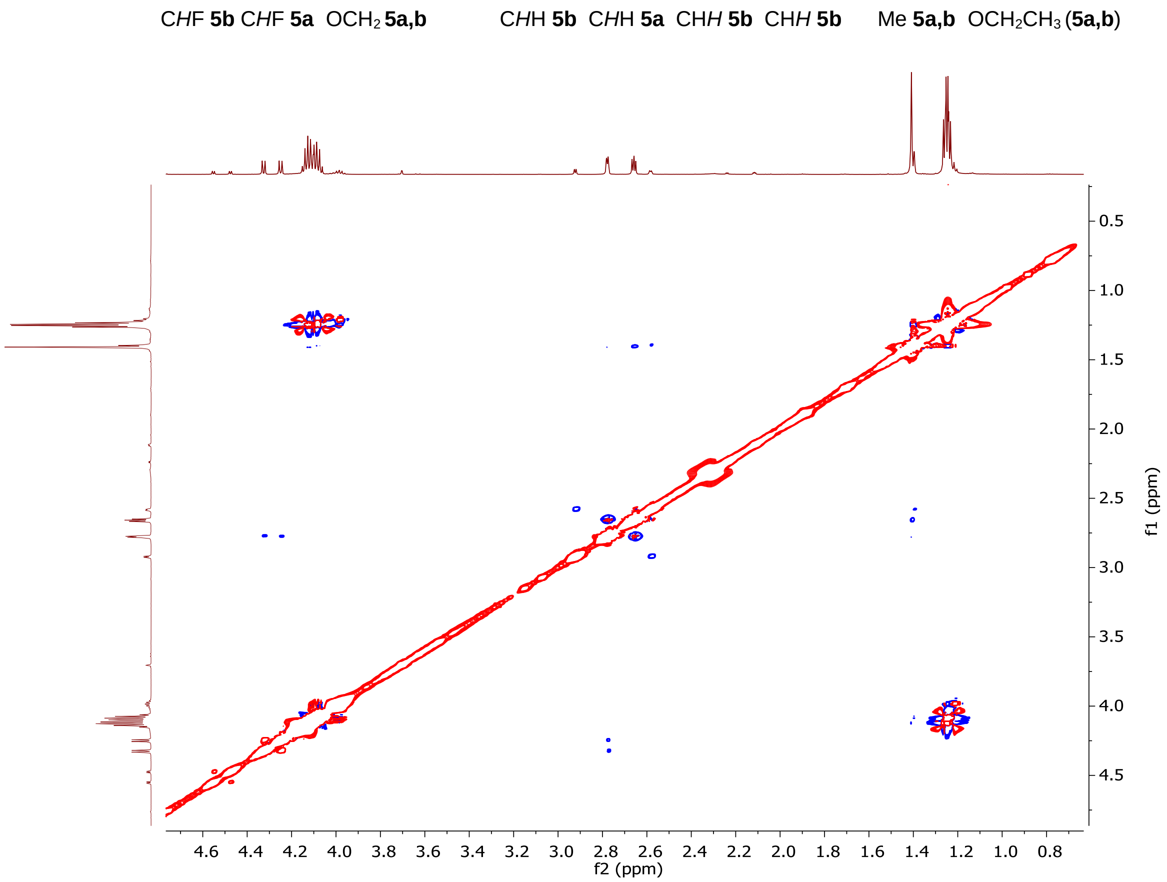

2D NOESY of rac **5a,b** (4.3:1, d.r.)

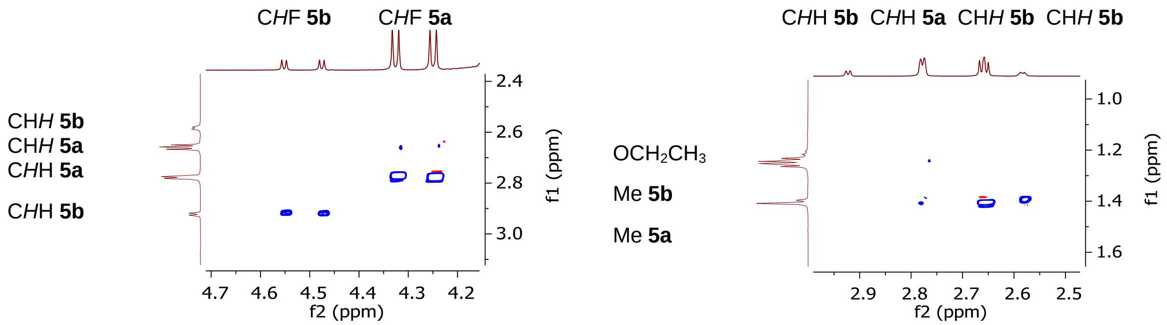

Diagnostic fragments of **2D NOESY of** rac **5a,b** (4.3:1, d.r.)

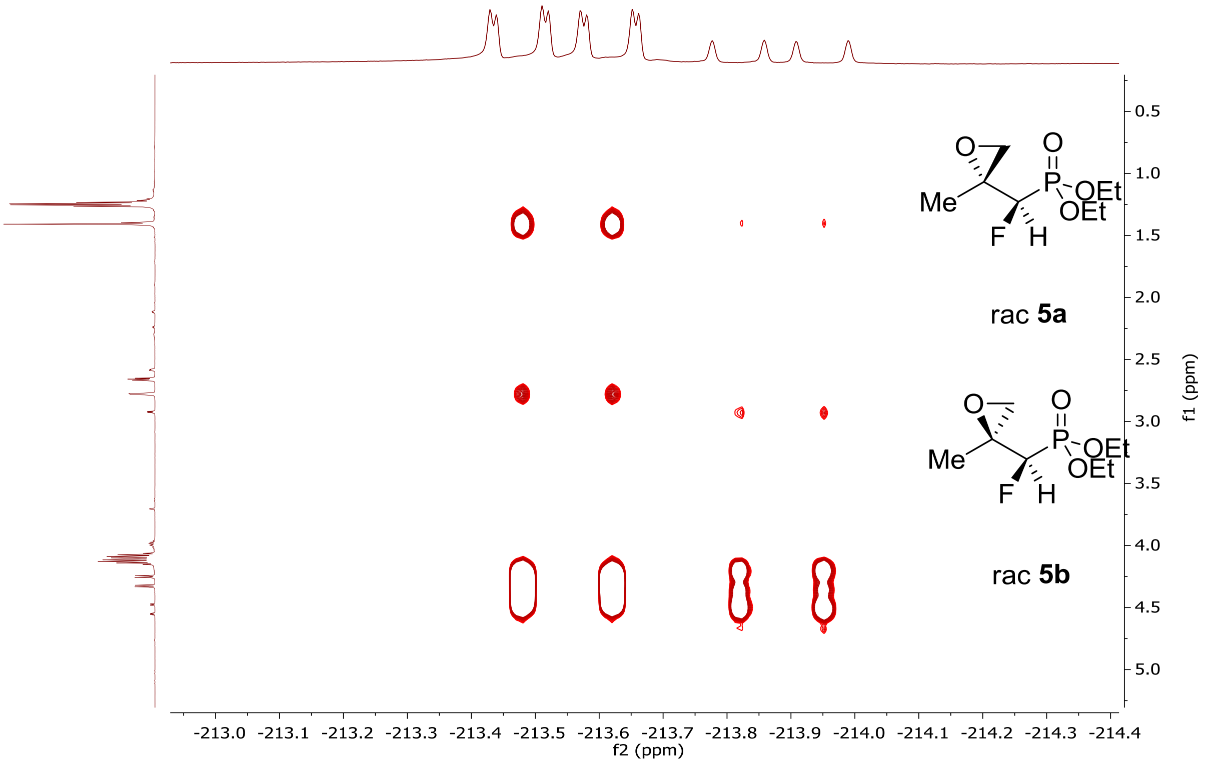

2D H-F HOESY of rac **5a,b** (4.3:1, d.r.)

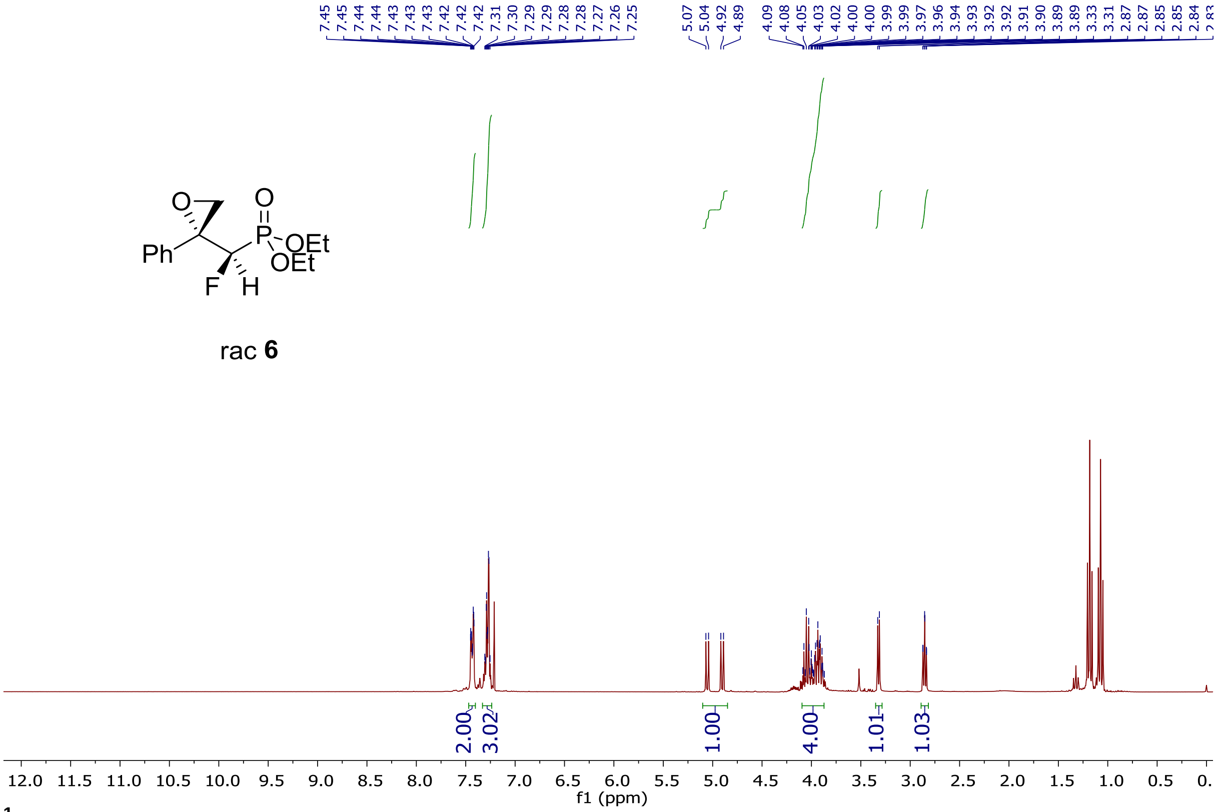

^{1}H NMR of rac **6**

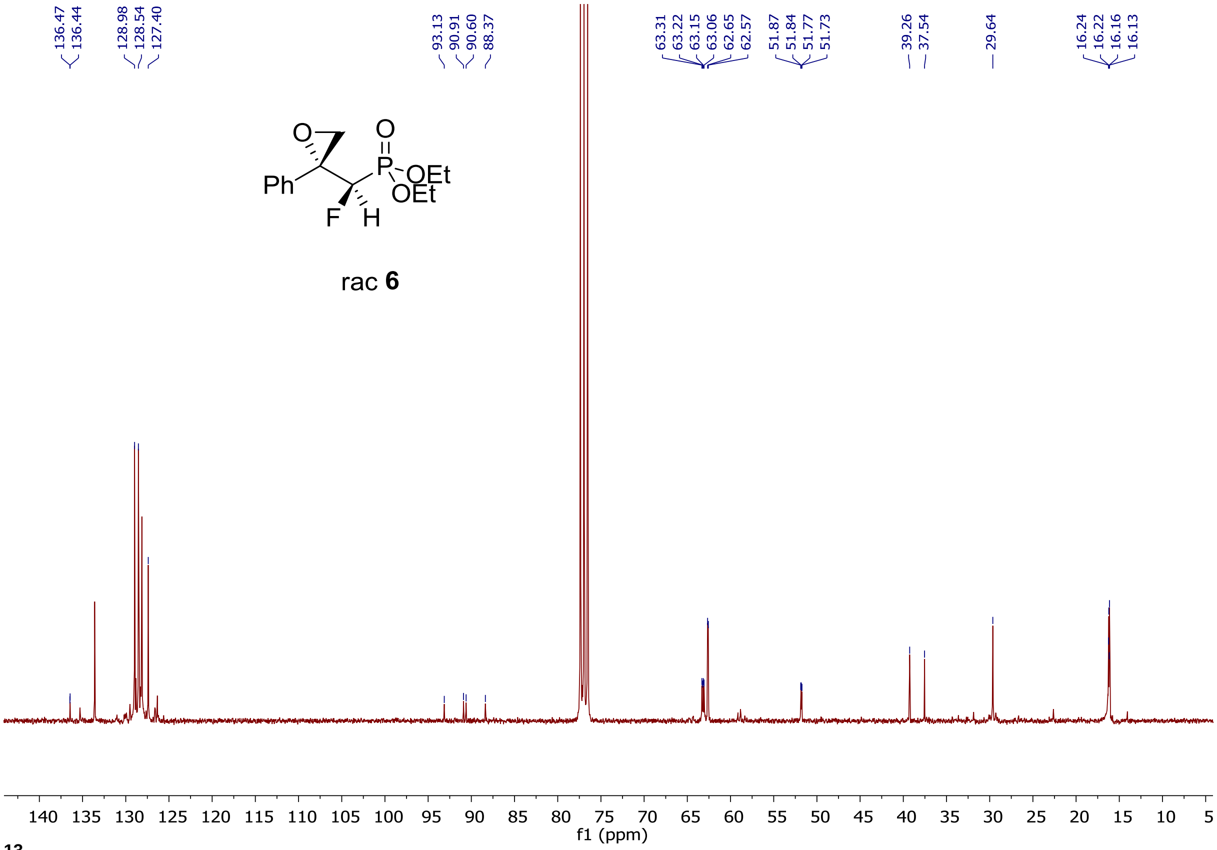

^{13}C NMR of rac **6**

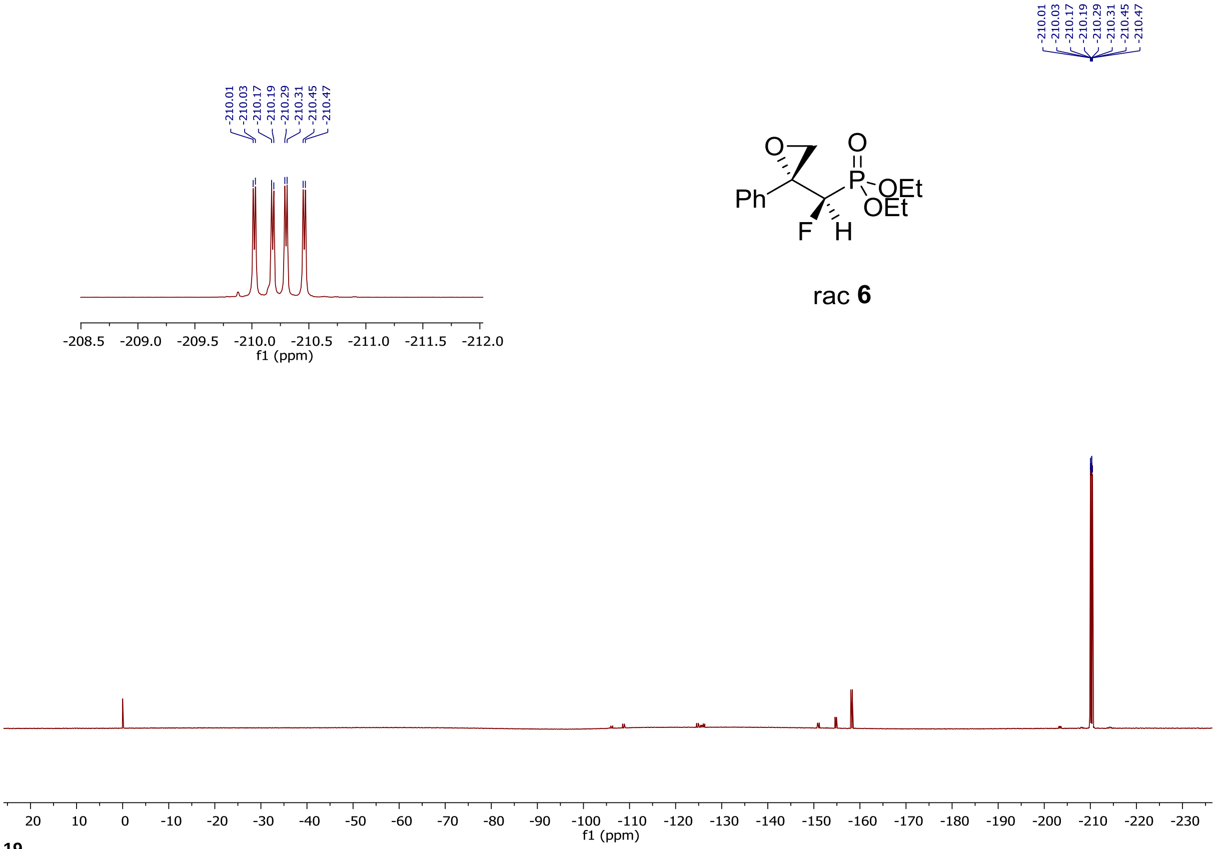

^{19}F NMR of rac **6**

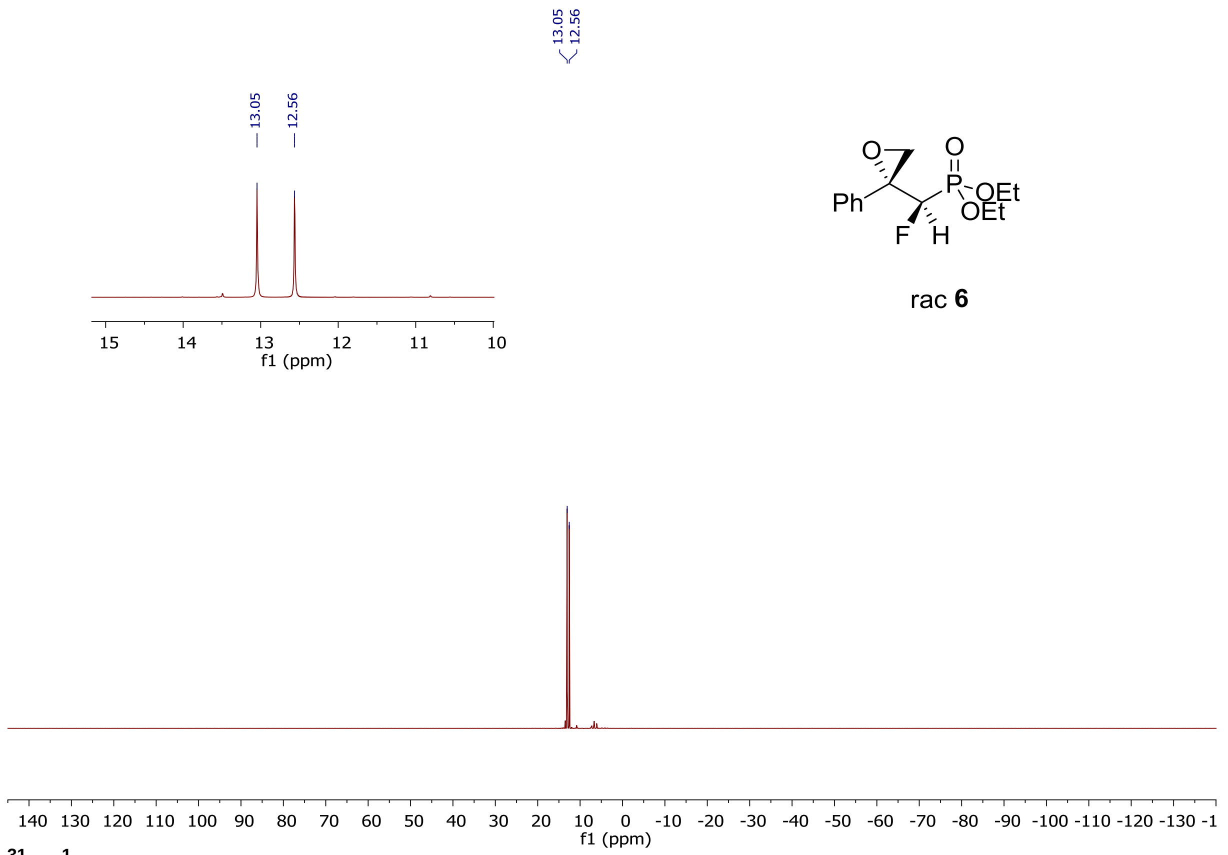

^{31}**P{/^{1}H} NMR of** rac **6**

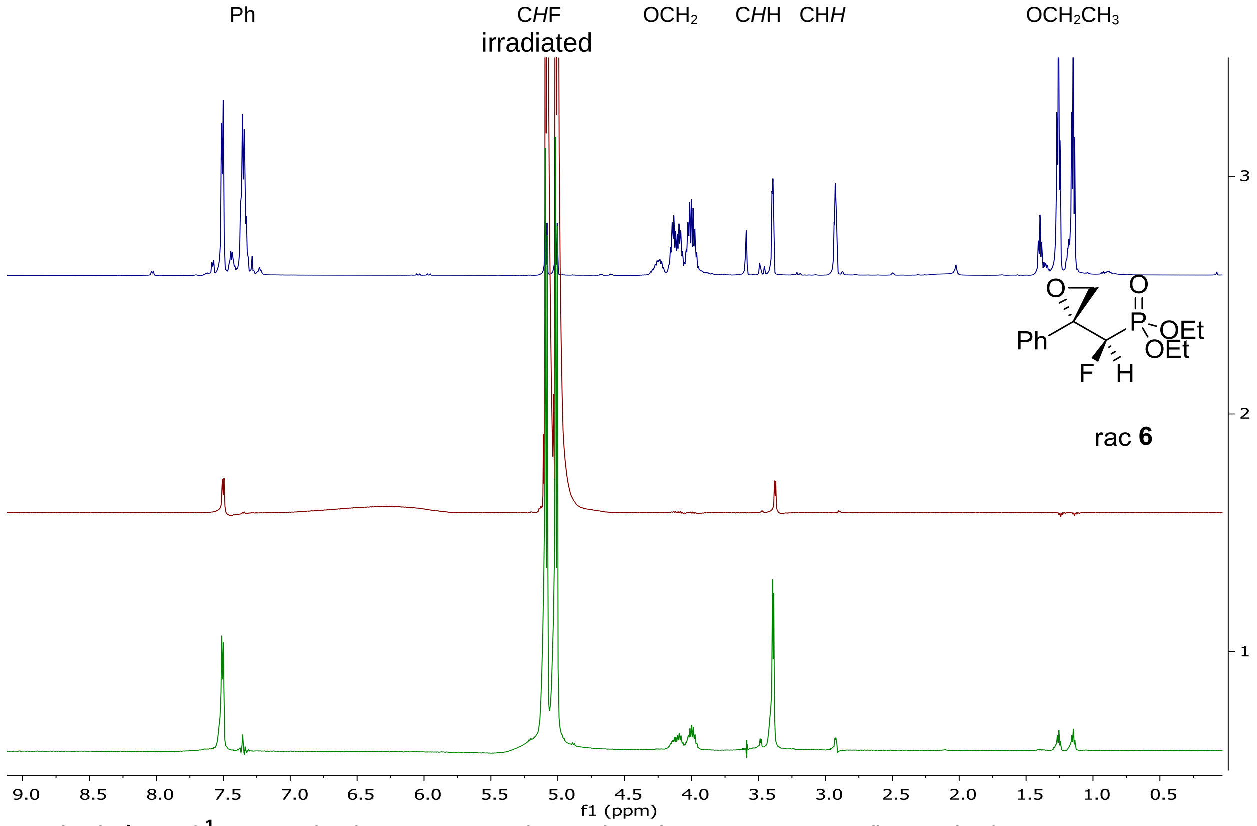

Stacked plots of ^{1}**H NMR** (top), **1D NOESY** (center) and **1D H-F HOESY** (bottom) of rac **6**

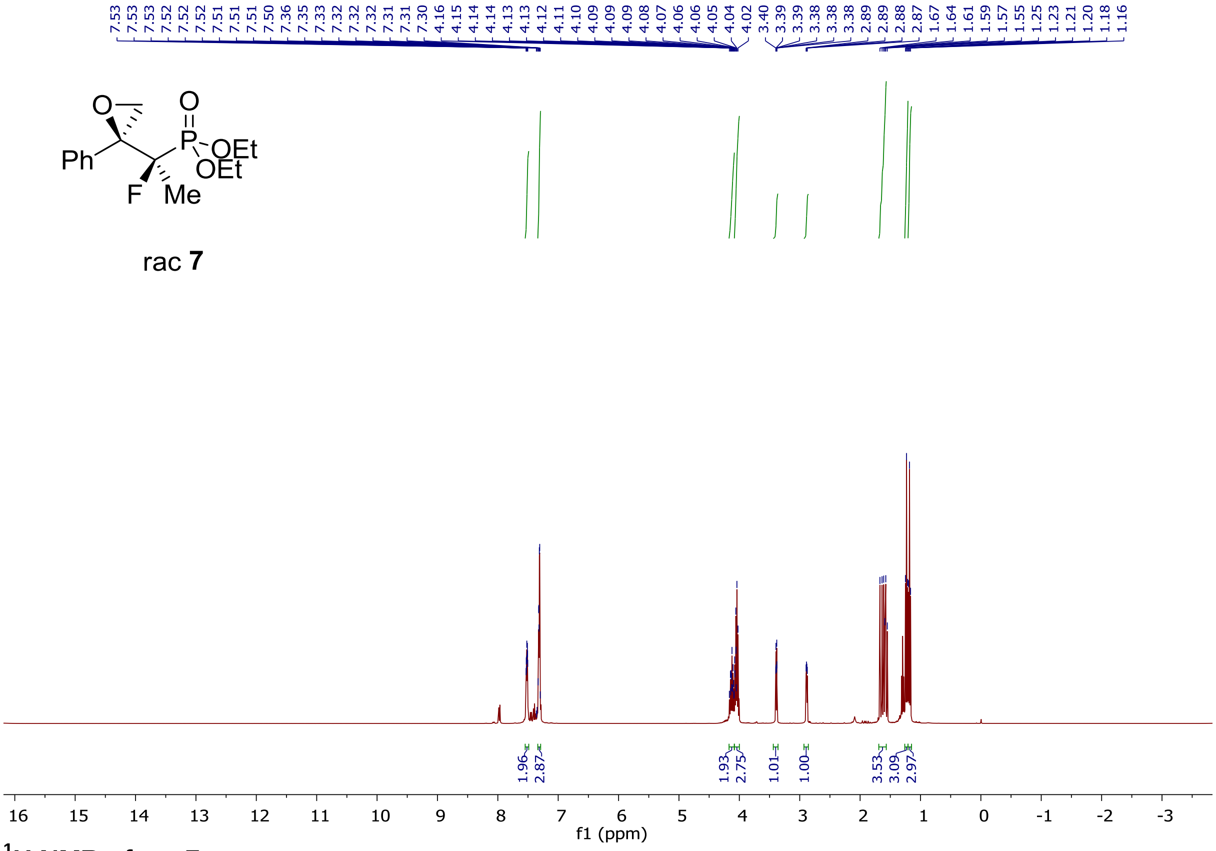

^{1}H NMR of rac **7**

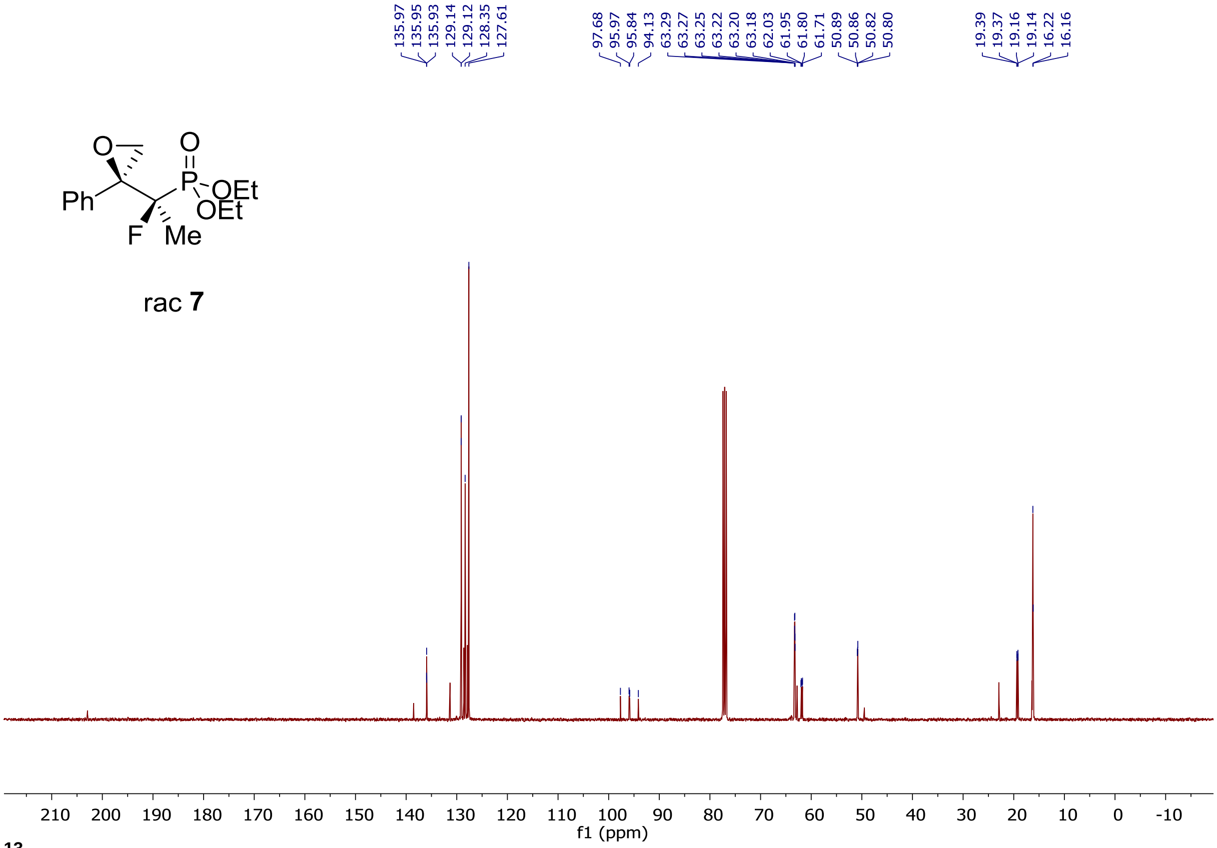

^{13}C NMR of rac **7**

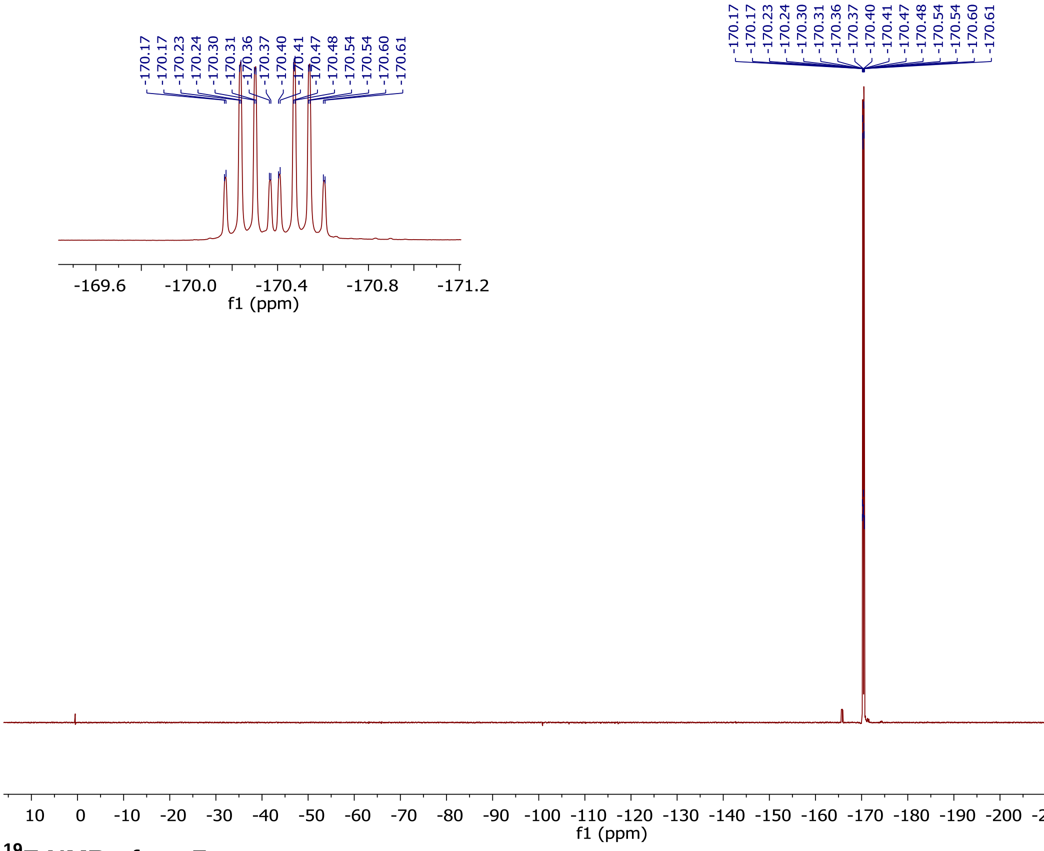

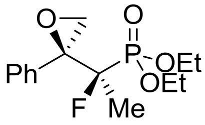

rac **7**

^{19}F NMR of rac **7**

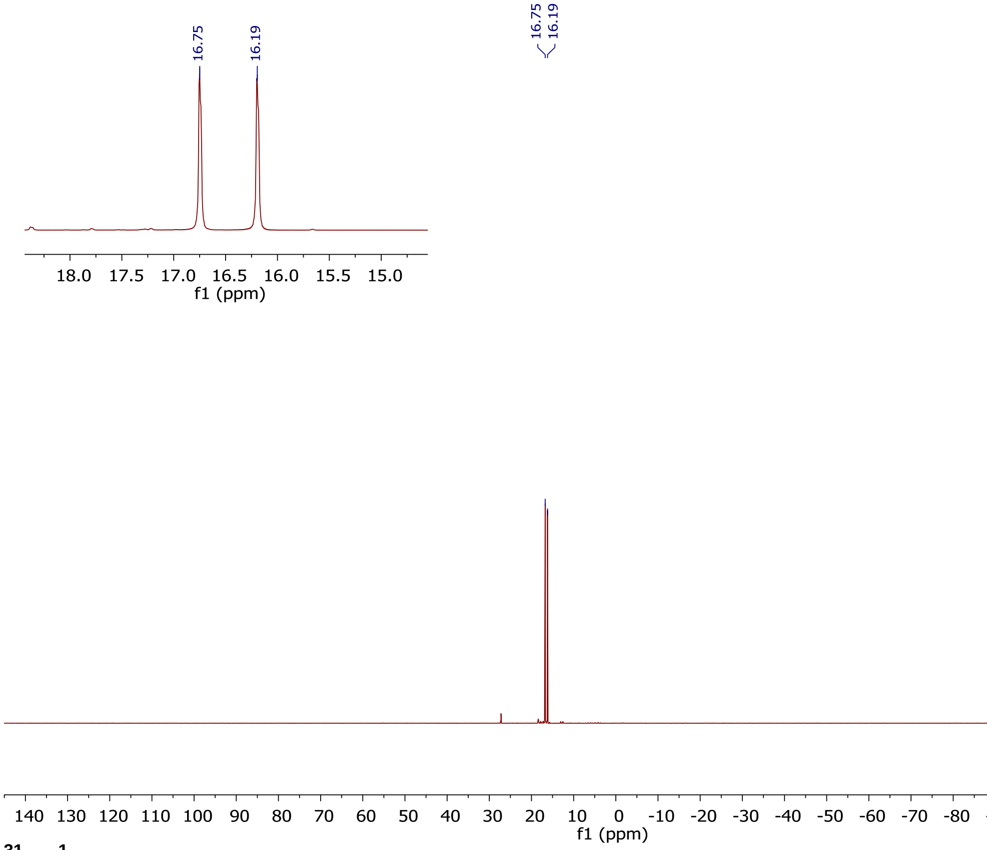

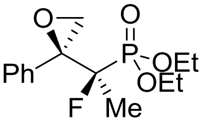

rac **7**

^{31}P{/^{1}H} NMR of rac **7**

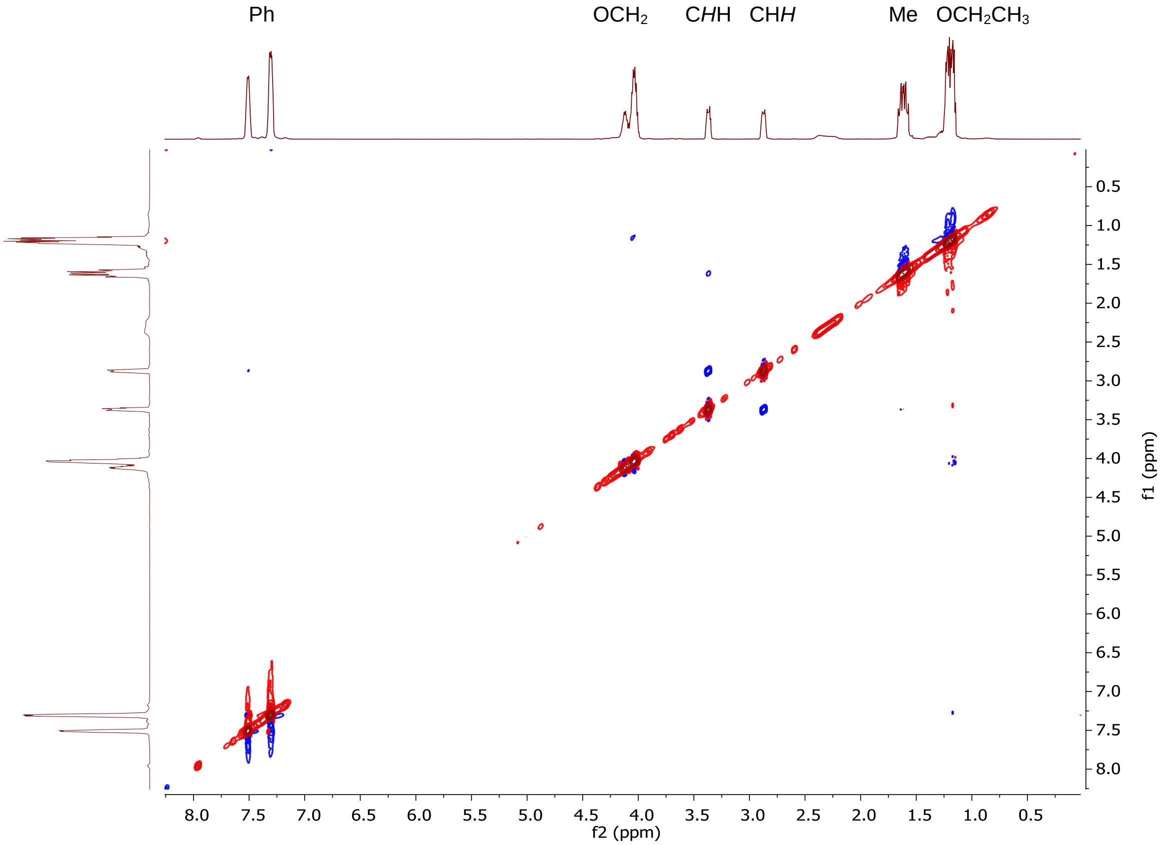

2D NOESY of rac **7**

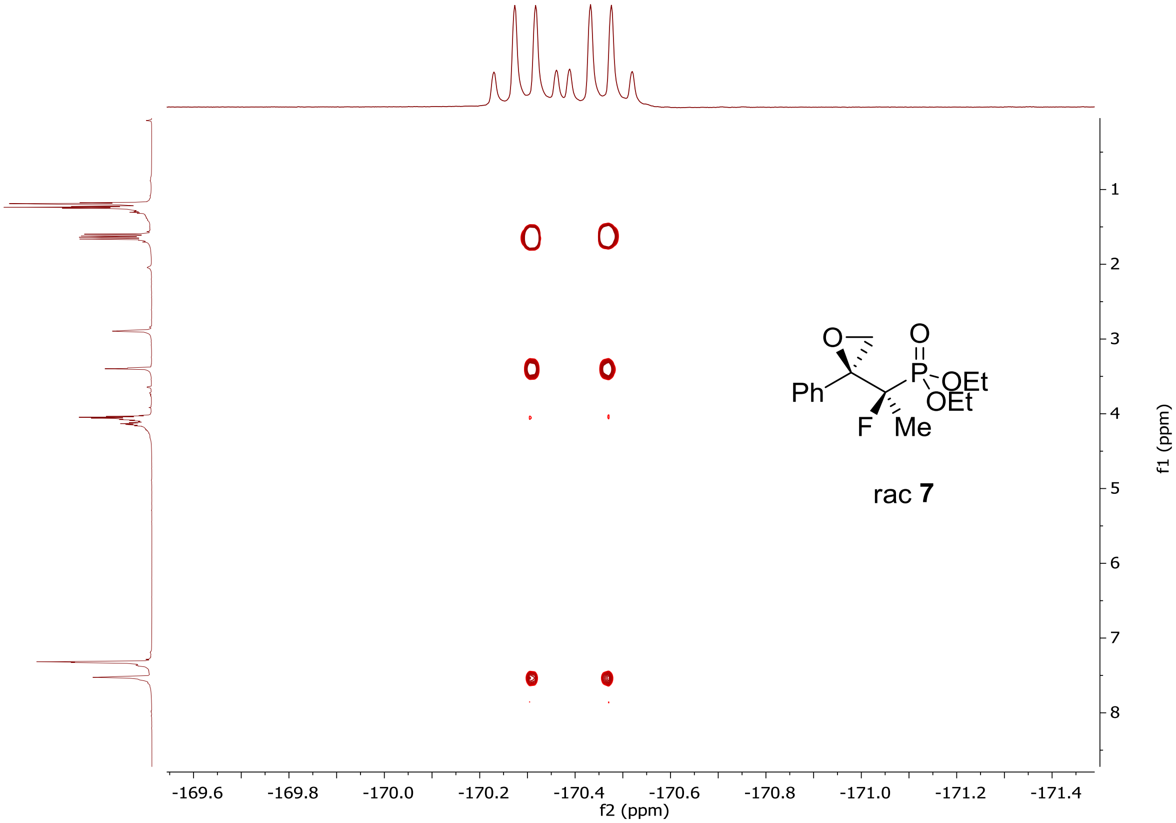

2D H-F HOESY of rac **7**

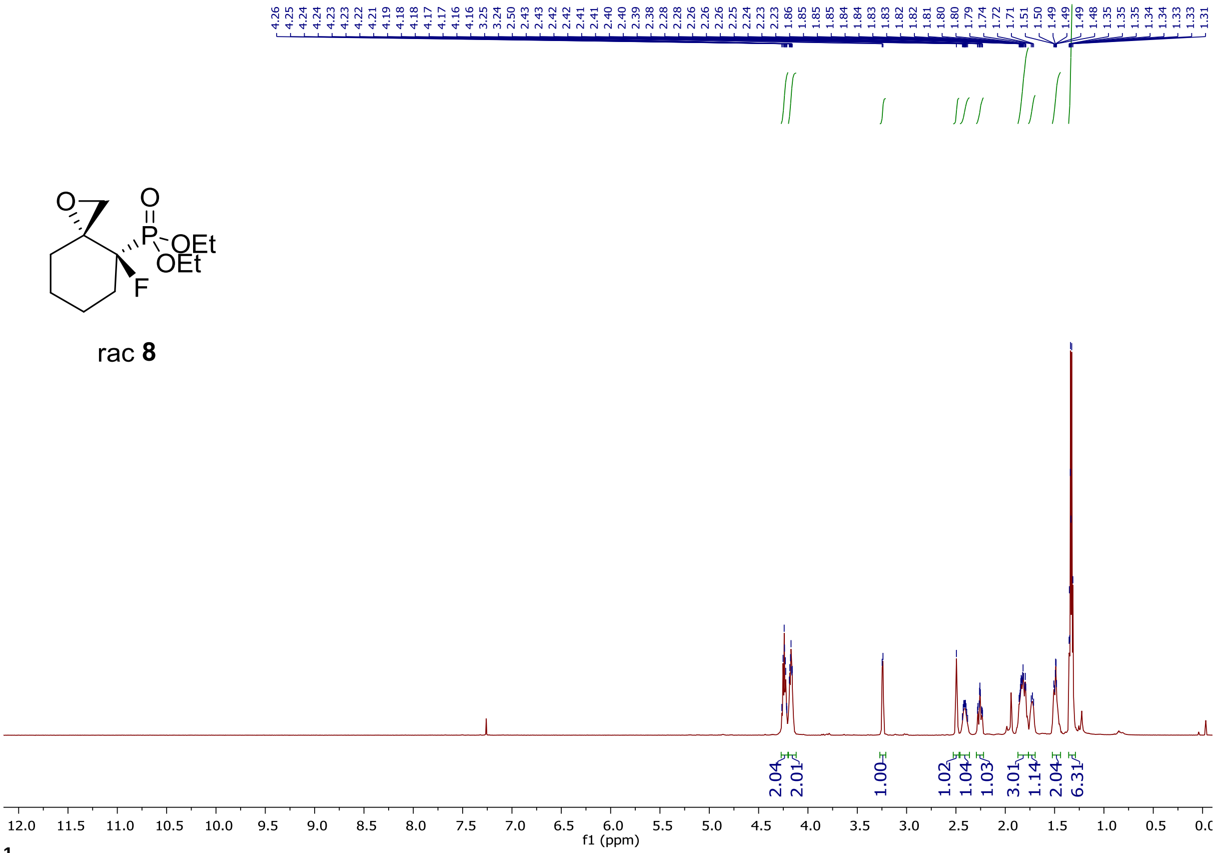

^{1}H NMR of rac **8**

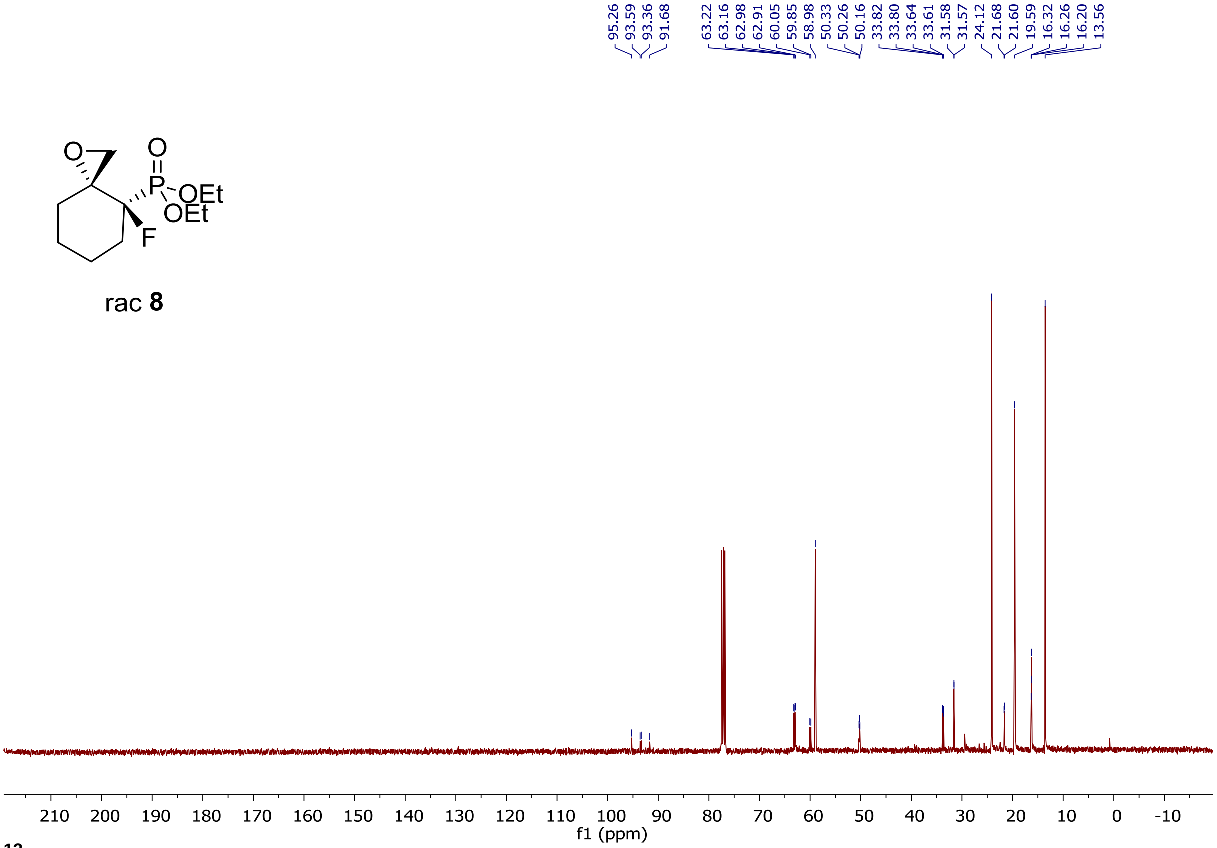

^{13}C NMR of rac **8**

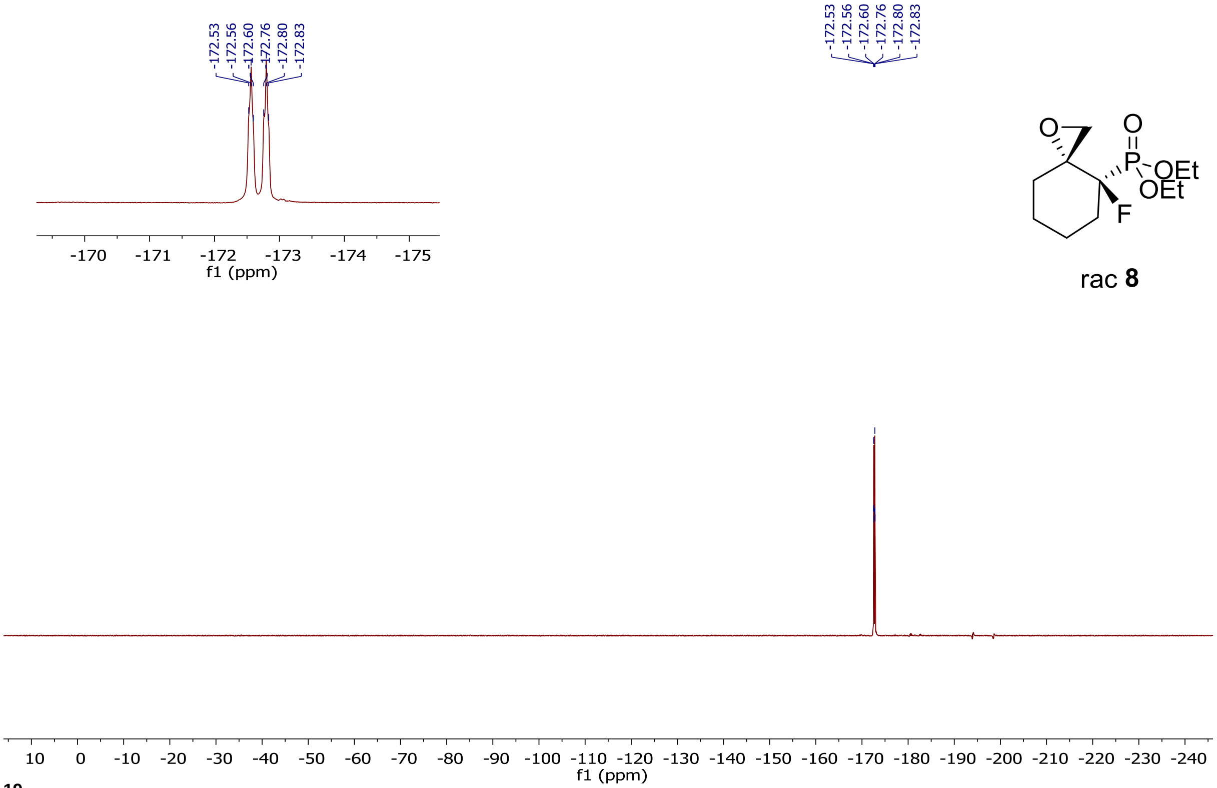

^{19}F NMR of rac **8**

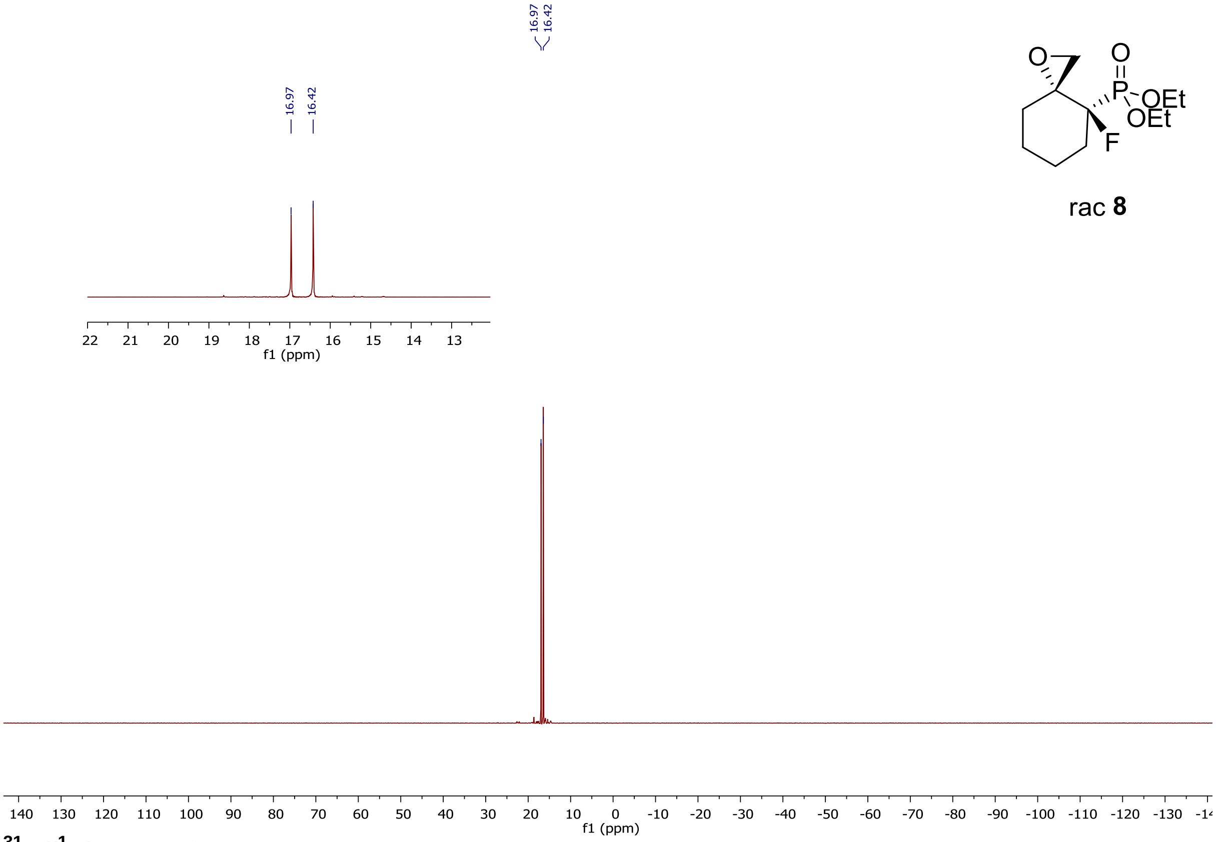

^{31}P{/^{1}H} NMR of rac **8**

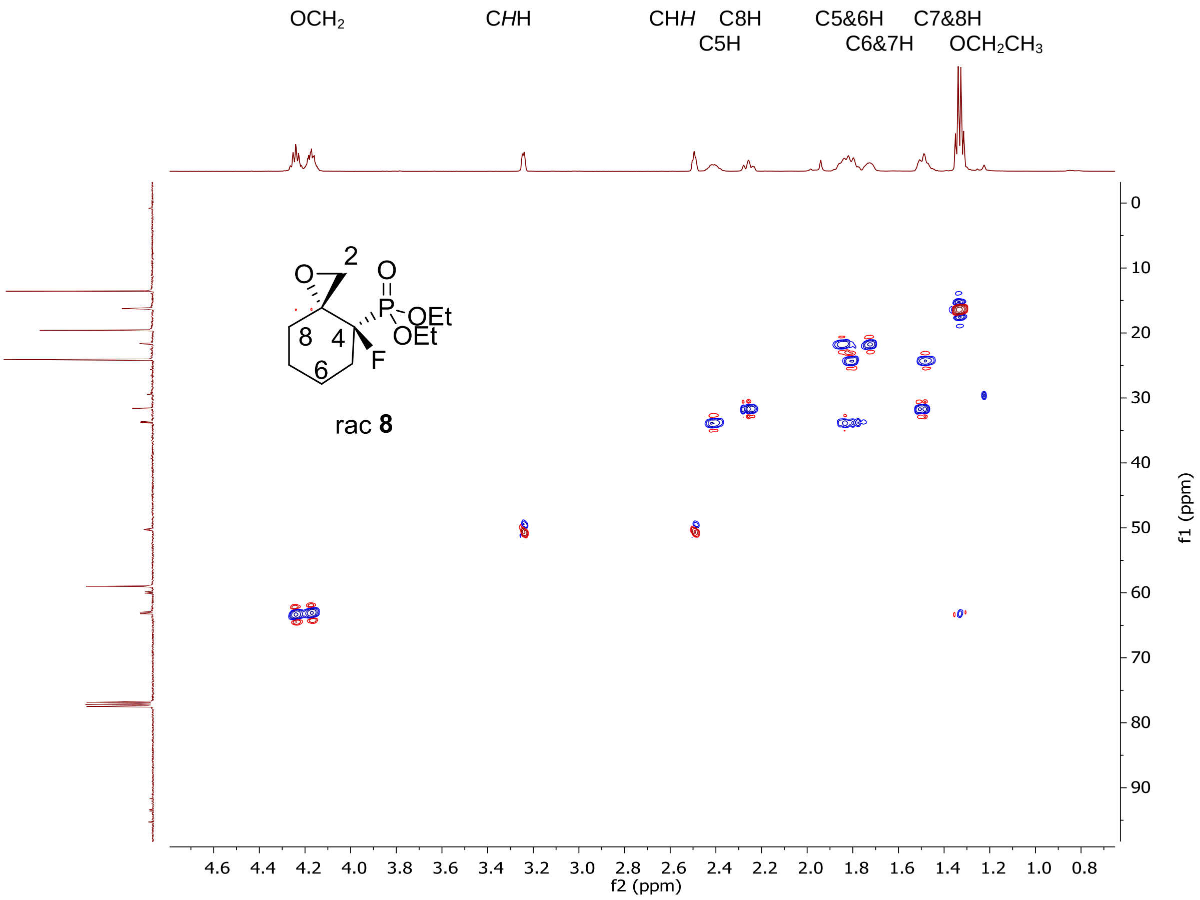

HSQC of rac **8**

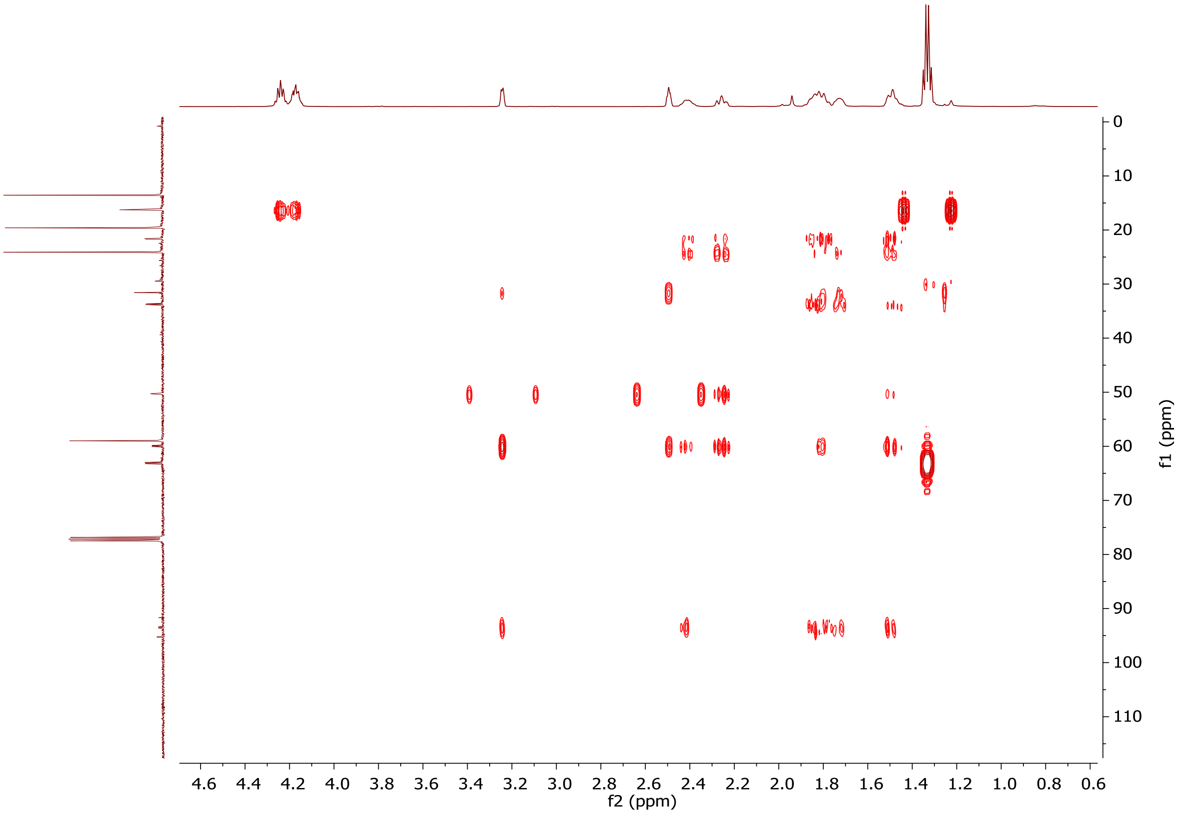

HMBC of rac **8**

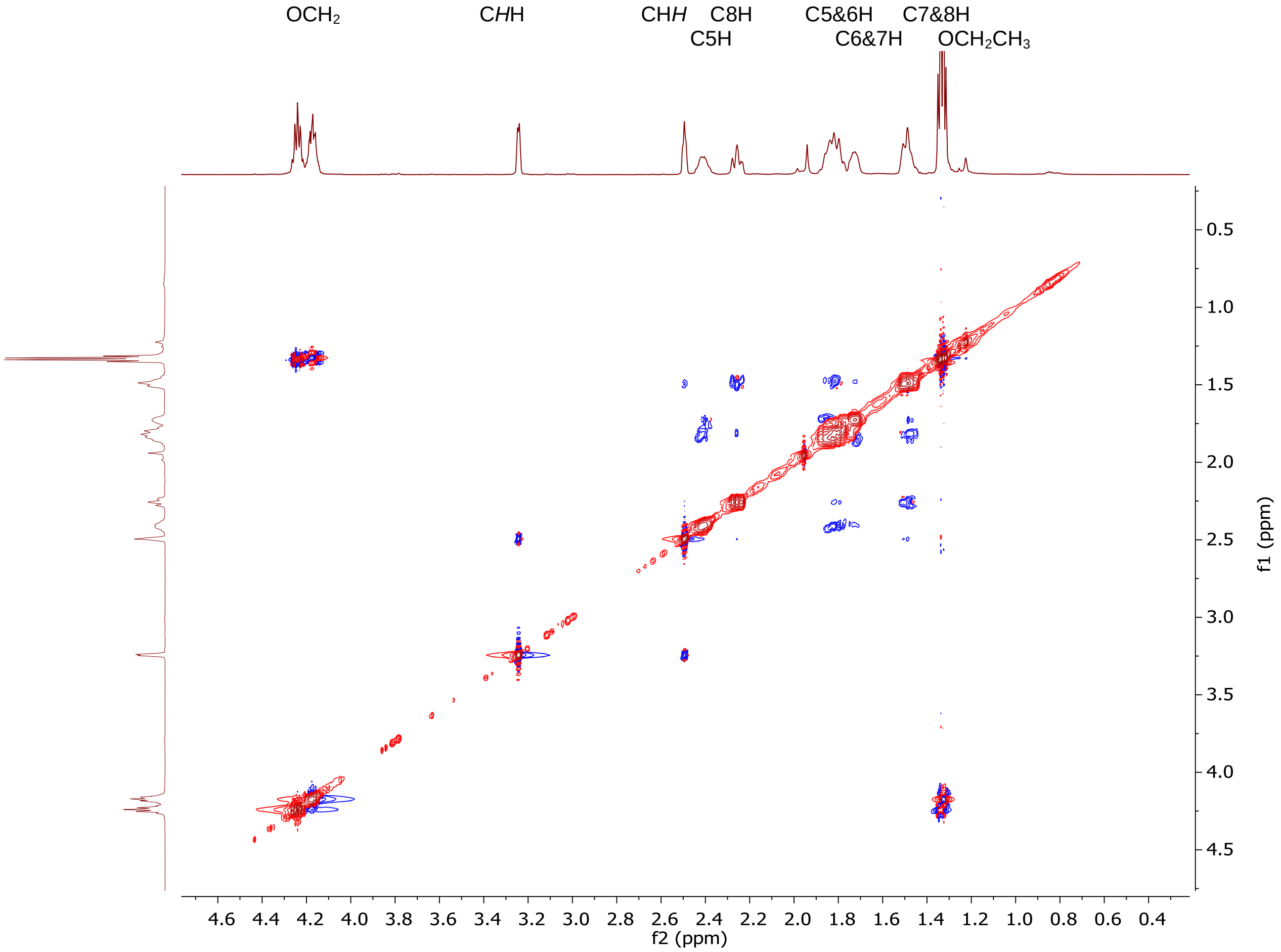

NOESY of rac **8**

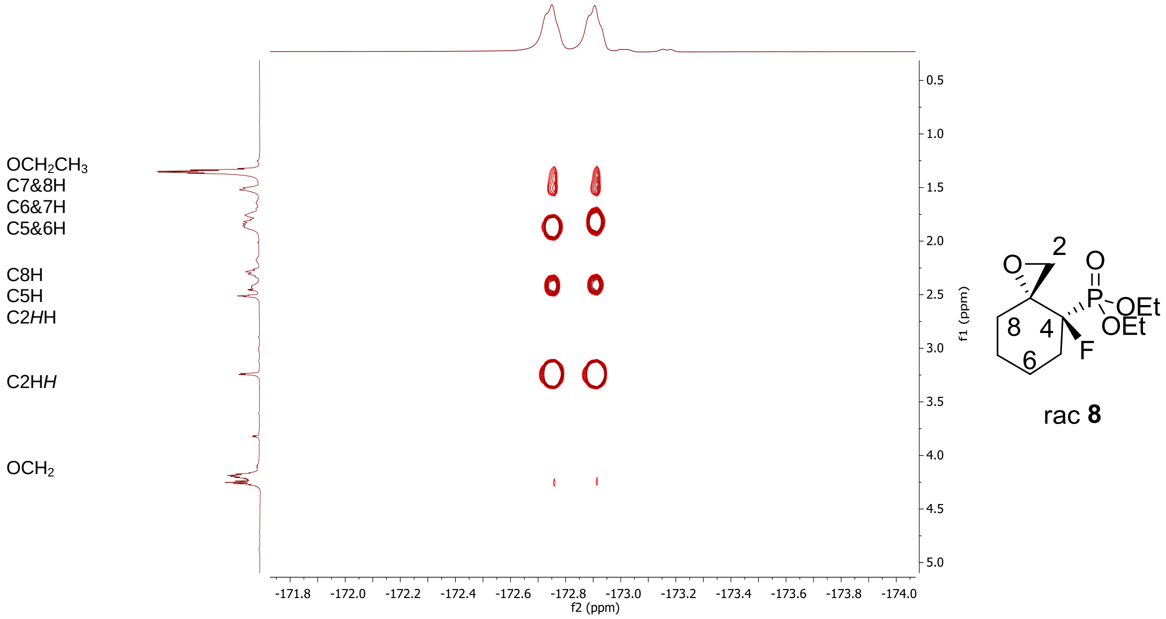

rac **8**

HOESY of rac **8**

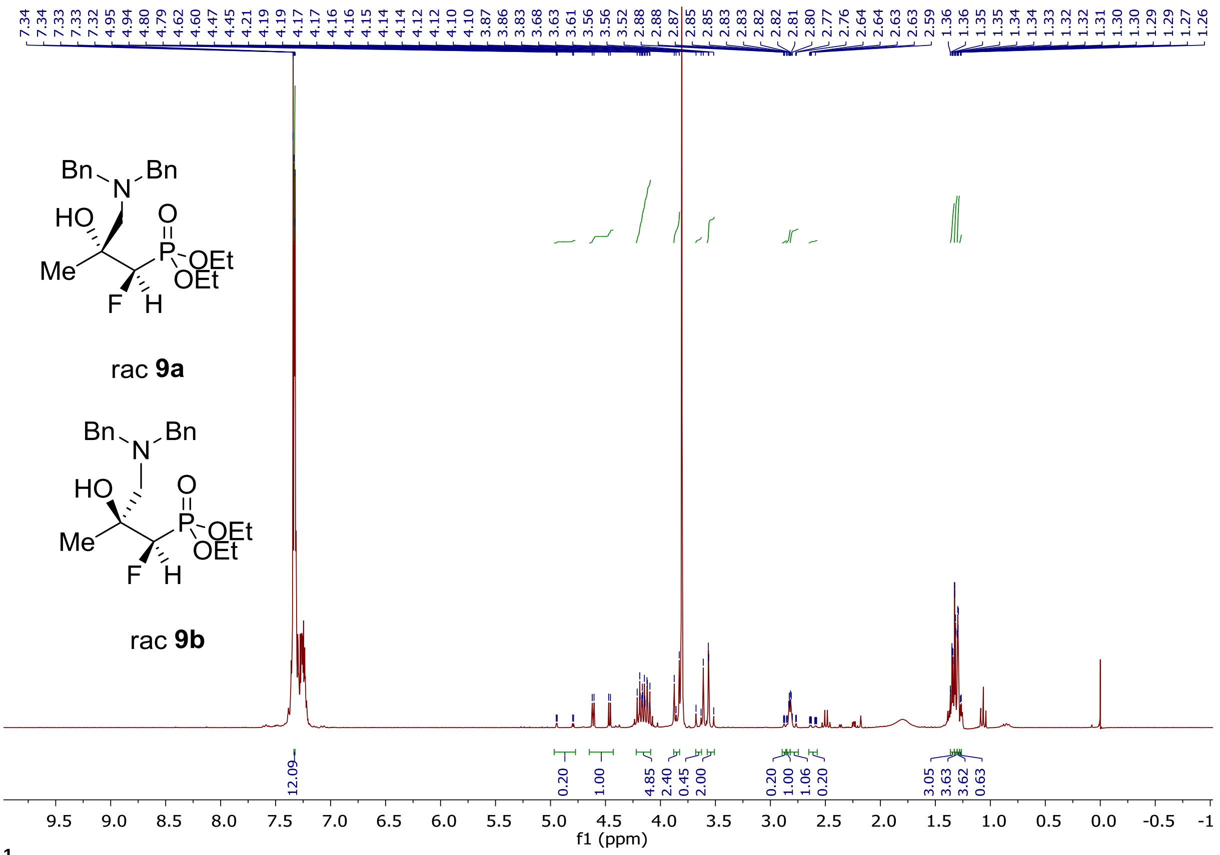

^{1}H NMR of rac **9a,b** (5:1, d.r.)

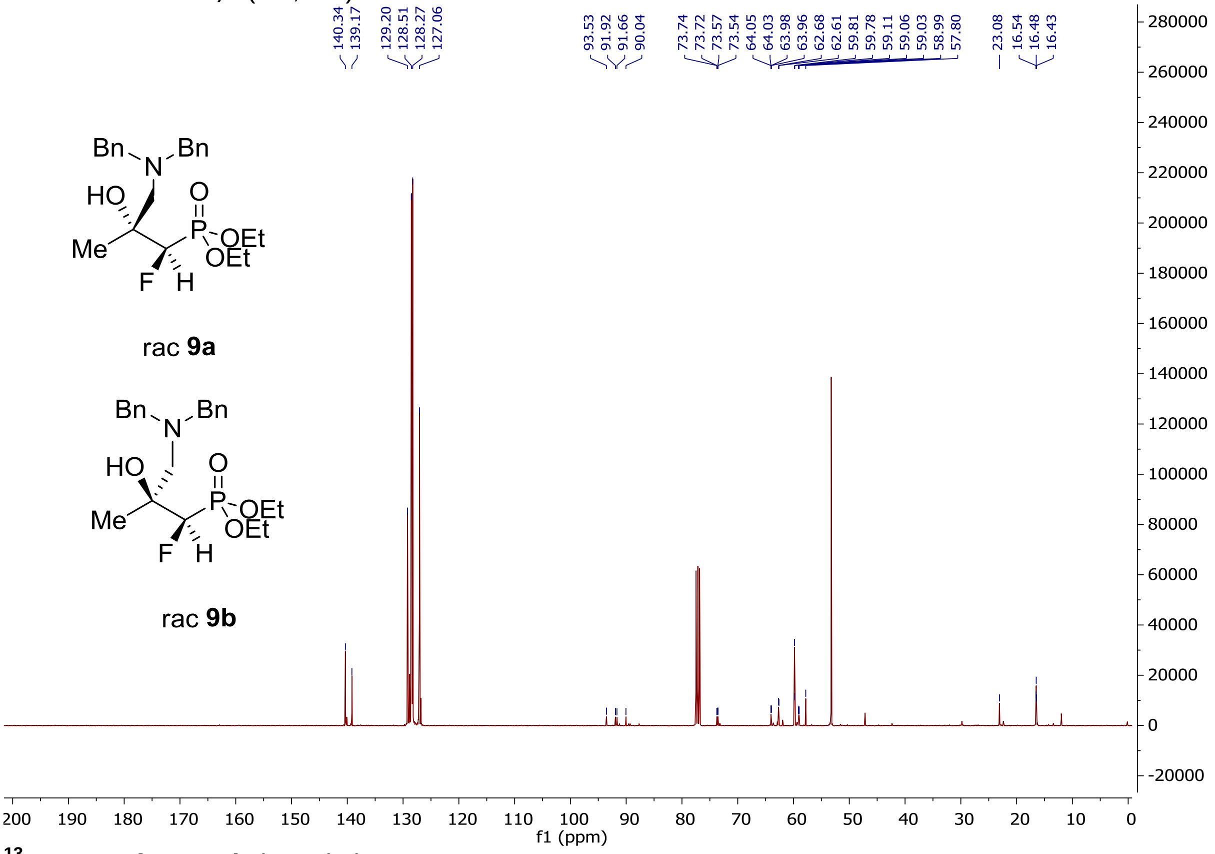

^{13}C NMR of rac **9a,b** (5:1, d.r.)

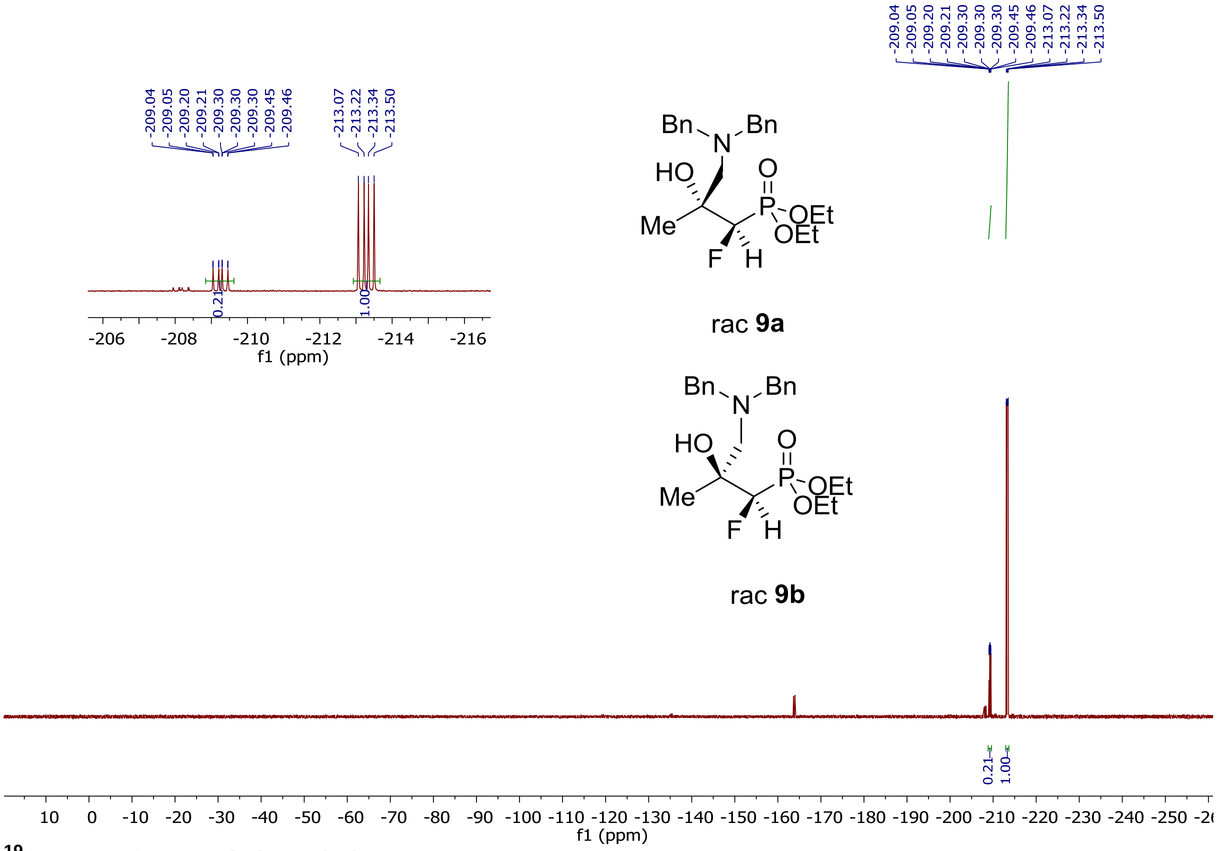

^{19}F NMR of rac **9a,b** (5:1, d.r.)

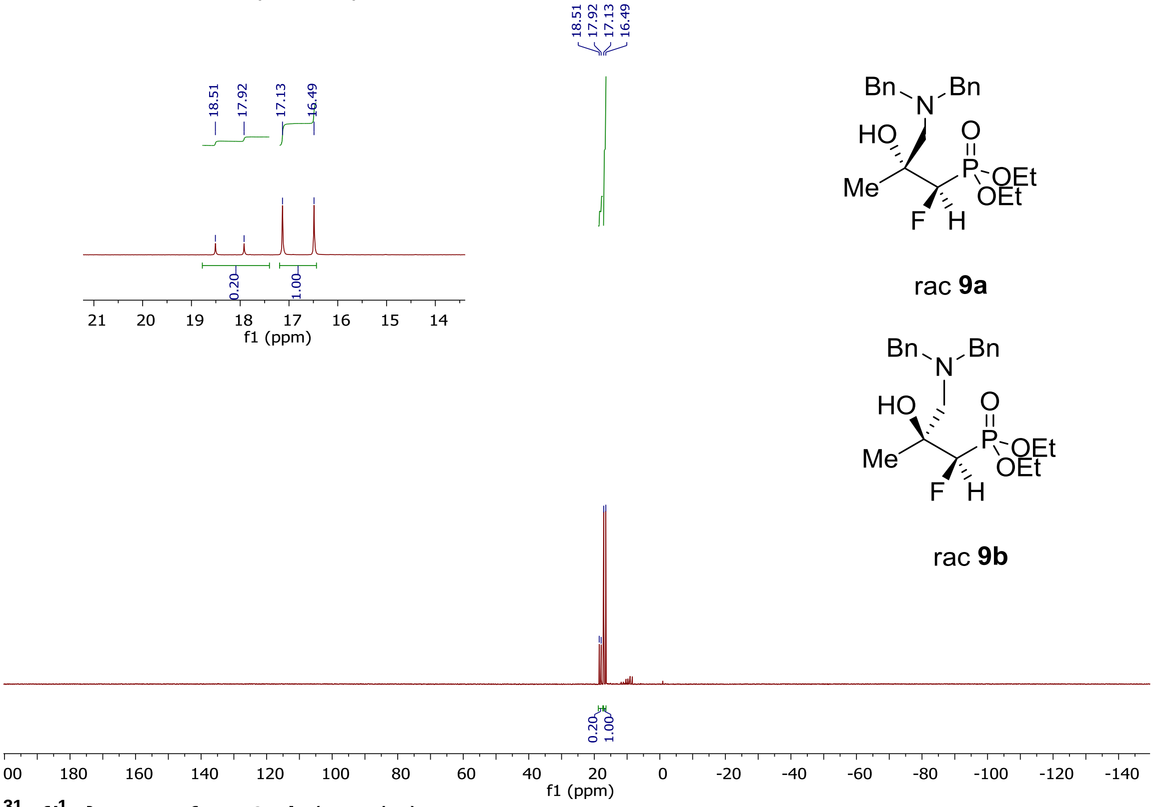

^{31}P{/^{1}H} NMR of rac **9a,b** (5:1, d.r.)

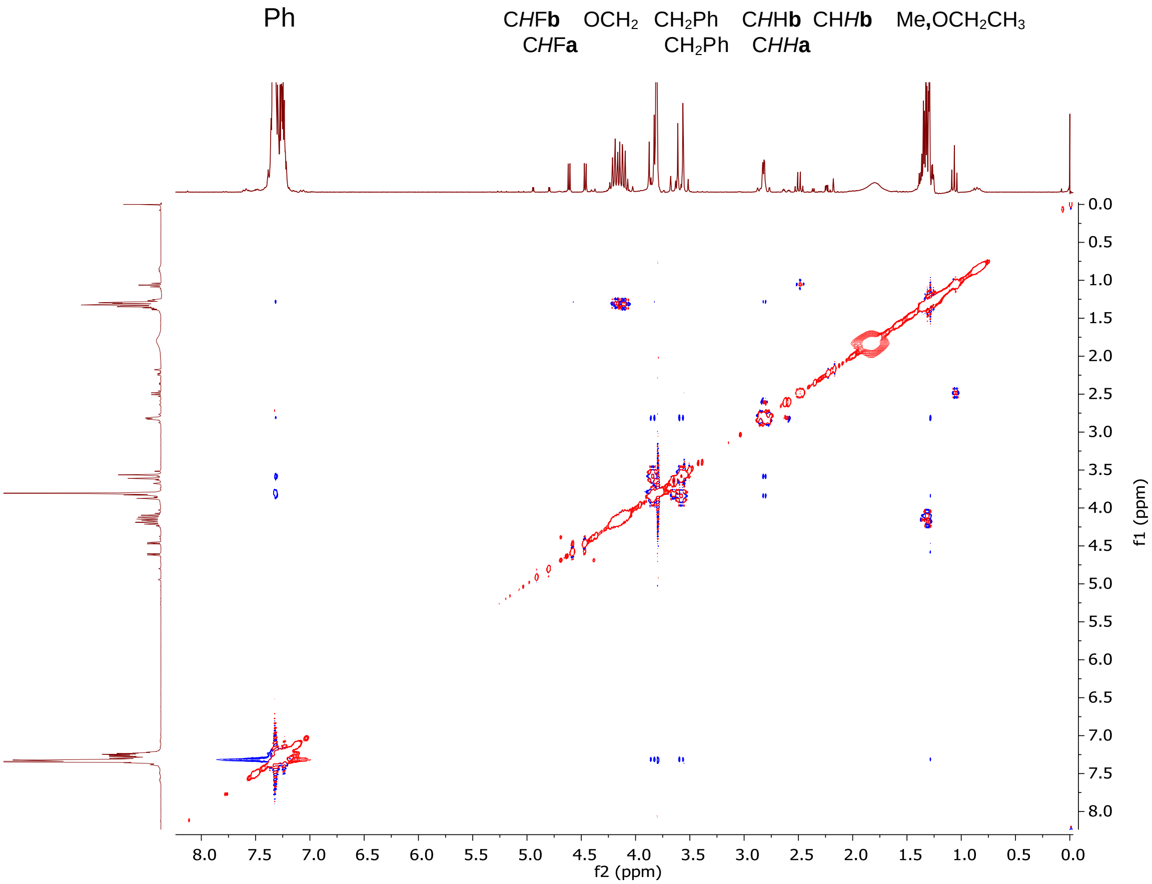

2D NOESY of rac **9a,b** (5:1, d.r.)

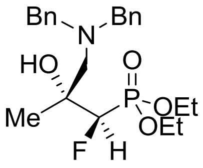

rac **9a**

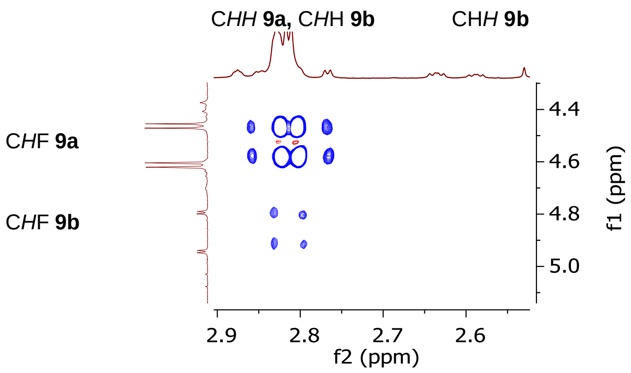

Diagnostic fragments of **2D NOESY of** rac **9a,b** (4.3:1, d.r.)

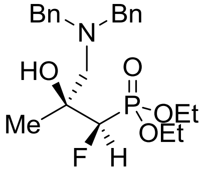

rac **9b**

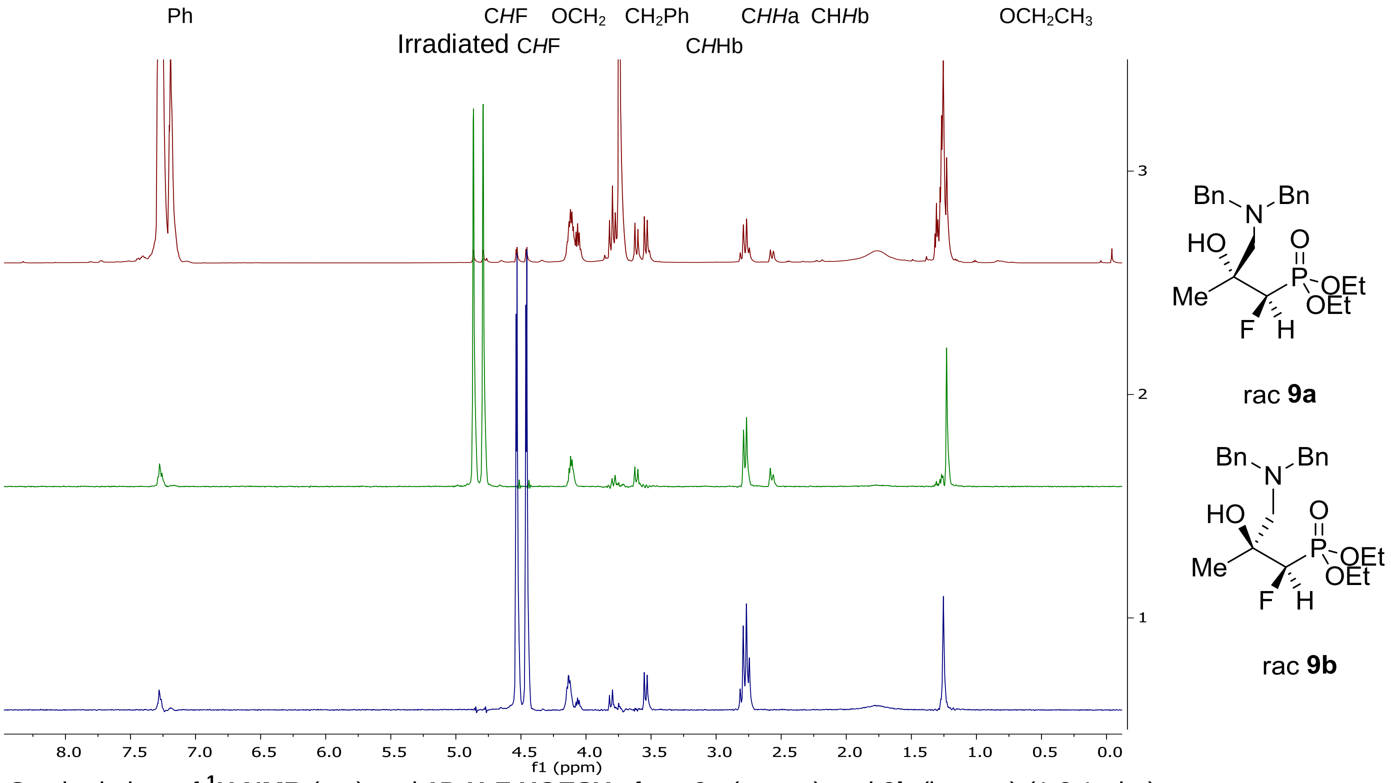

Stacked plots of [1]**H NMR** (top) and **1D H-F HOESY** of rac **9a** (center) and **9b** (bottom) (1.2:1, d.r.)

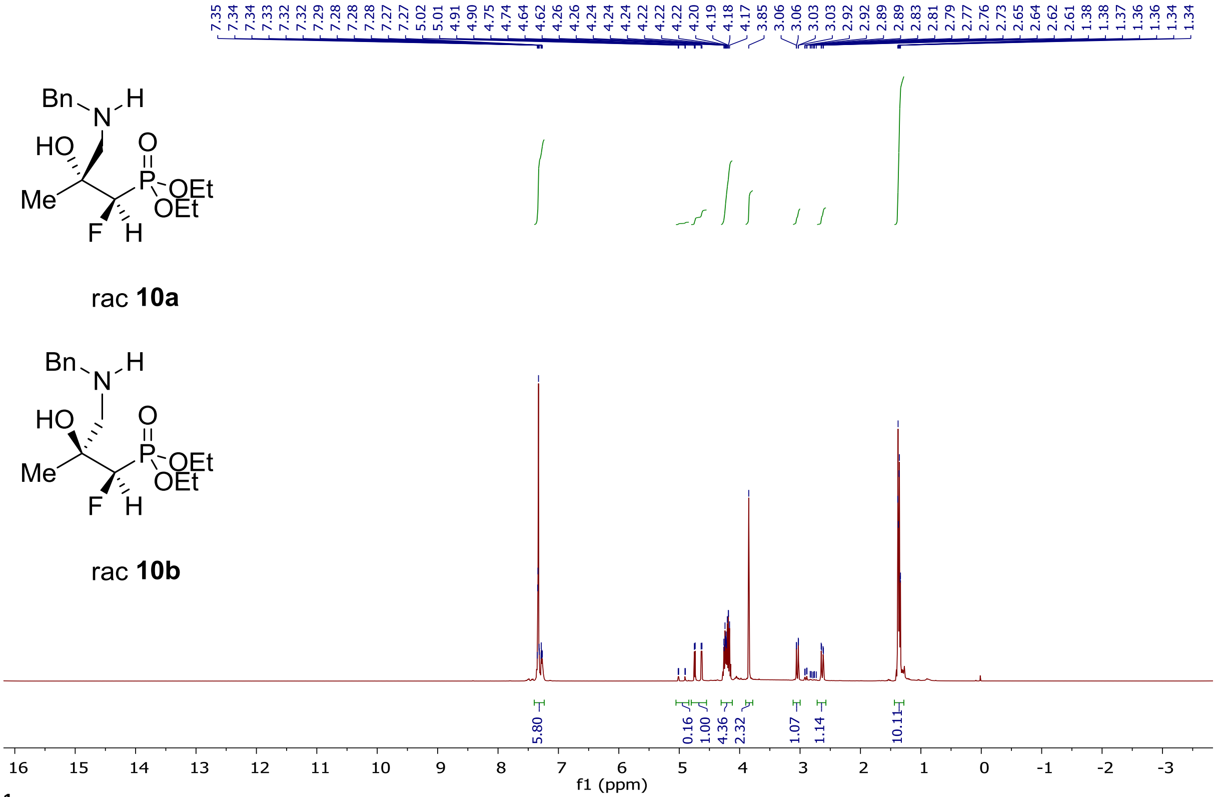

[1]**H NMR of** rac **10a,b** (6.2:1, d.r.)

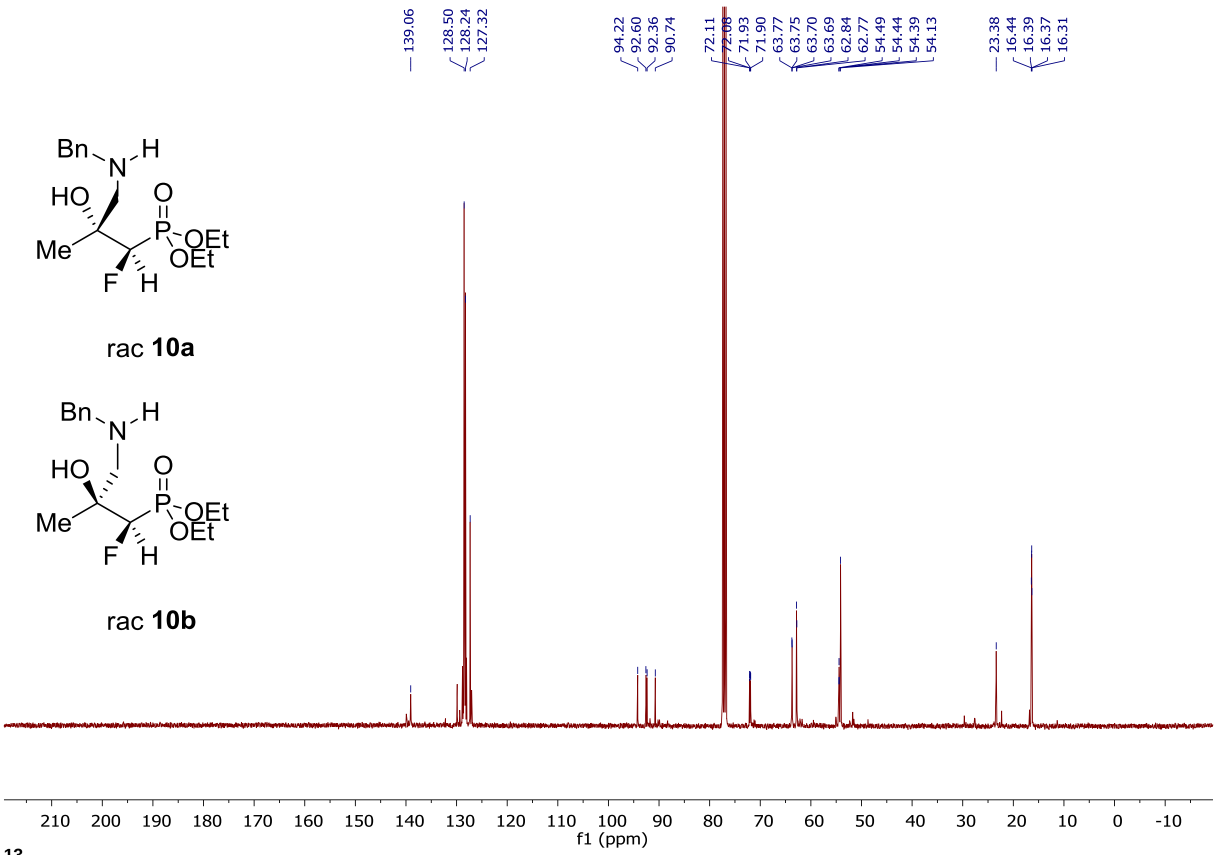

^{13}C NMR of rac **10a,b** (6.2:1, d.r.)

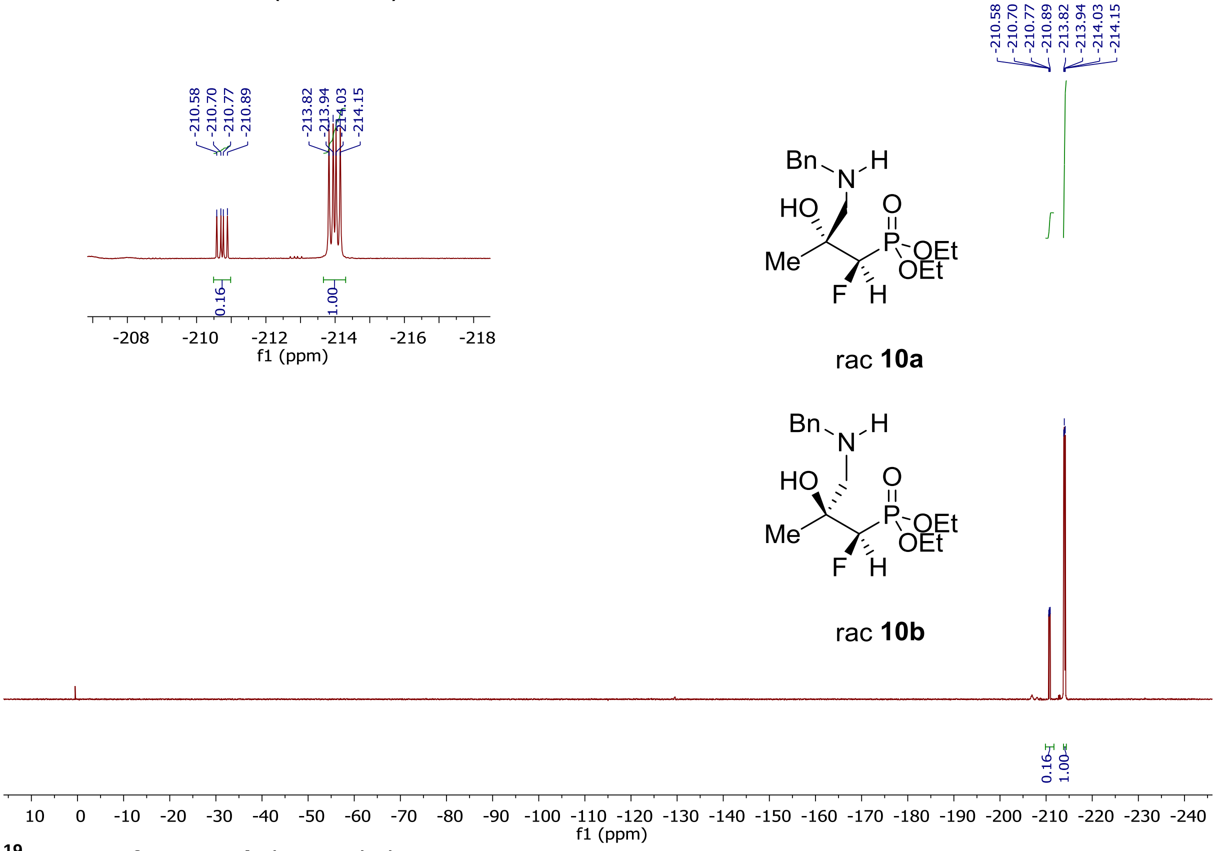

^{19}F NMR of rac **10a,b** (6.2:1, d.r.)

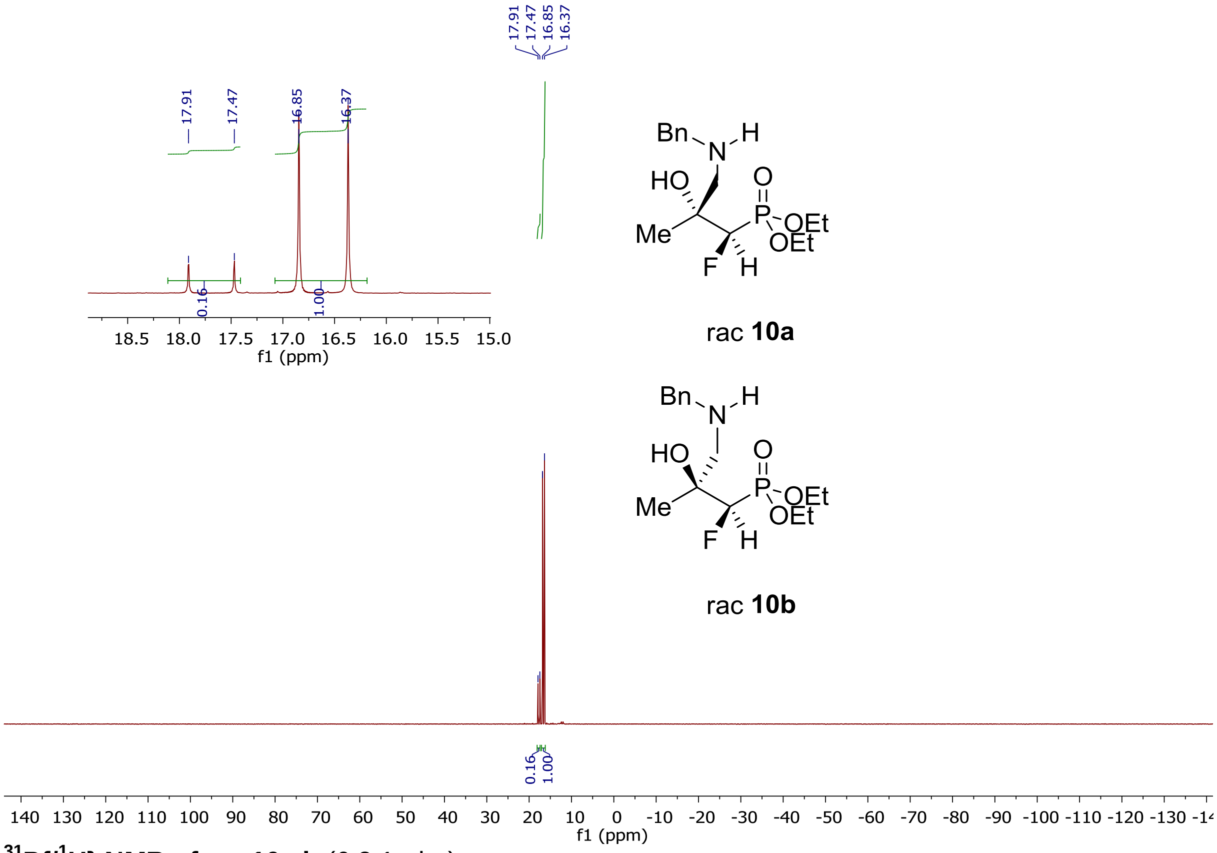

^{31}**P{/^{1}H} NMR of** rac **10a,b** (6.2:1, d.r.)

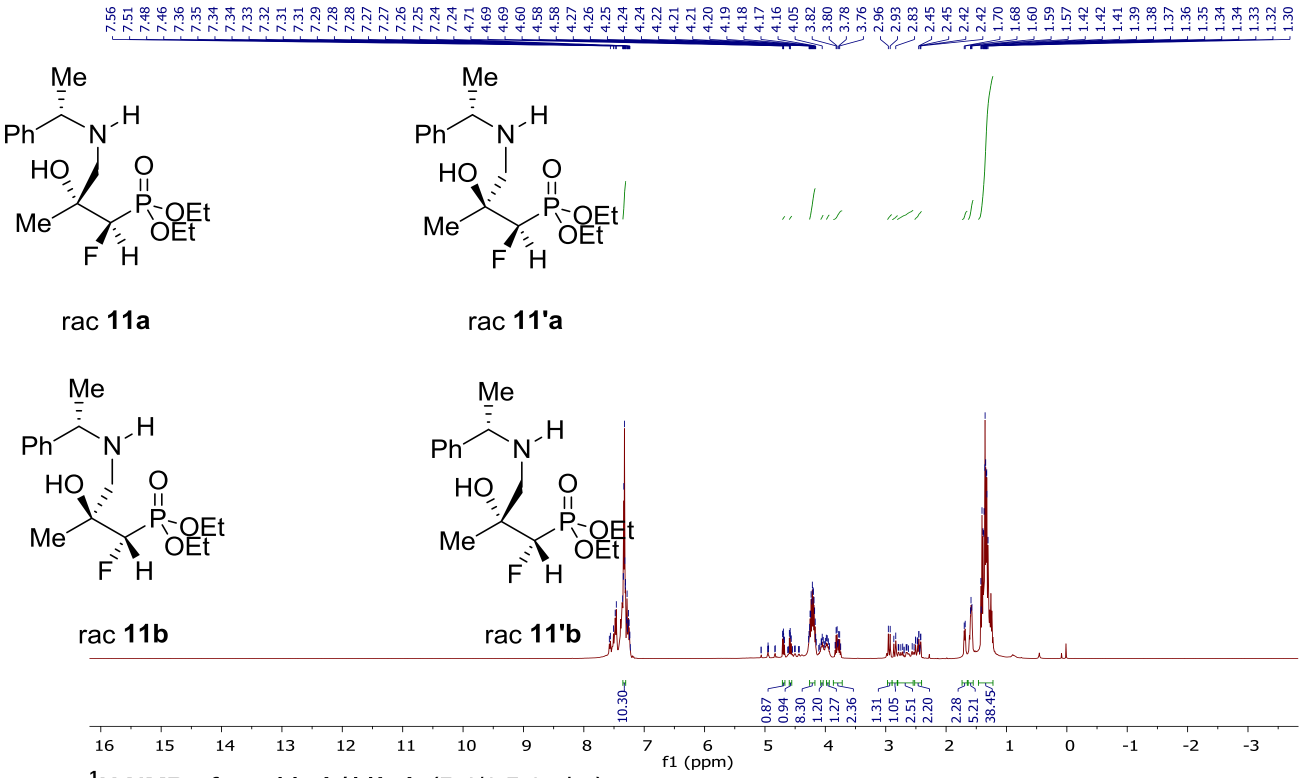

^{1}**H NMR of** rac **11a,b/11’a,b** (5:4/1.5:1, d.r.)

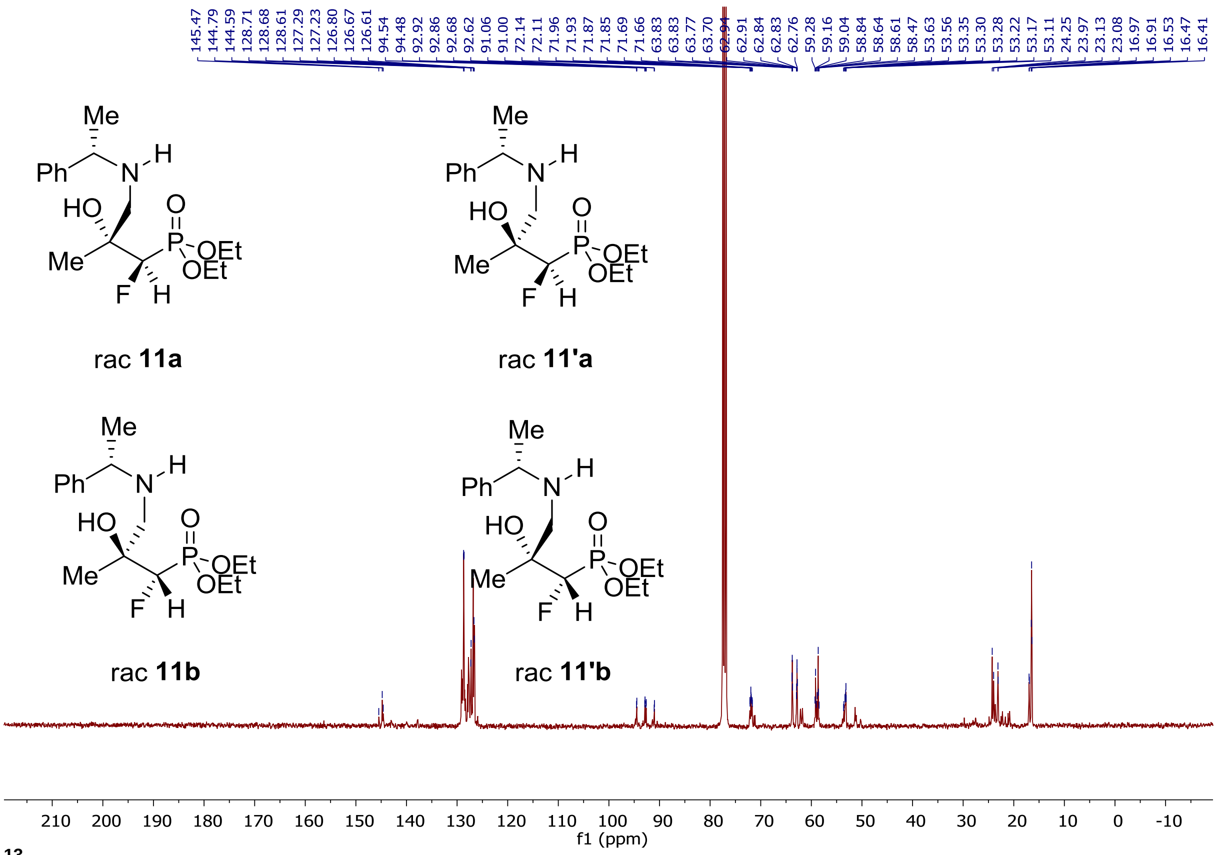

^{13}**C NMR of** rac **11a,b/11’a,b** (5:4/1.5:1, d.r.)

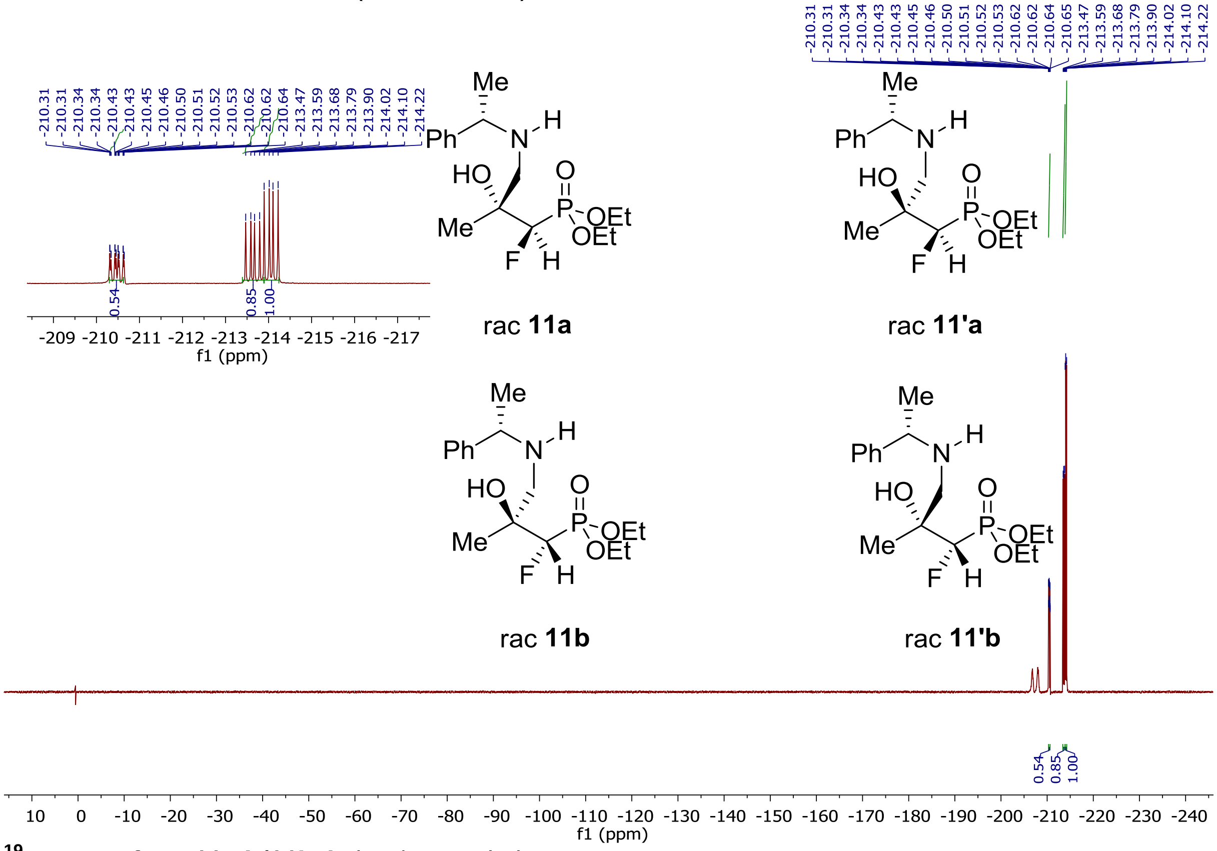

^{19}**F NMR of** rac **11a,b/11’a,b** (5:4/1.5:1, d.r.)

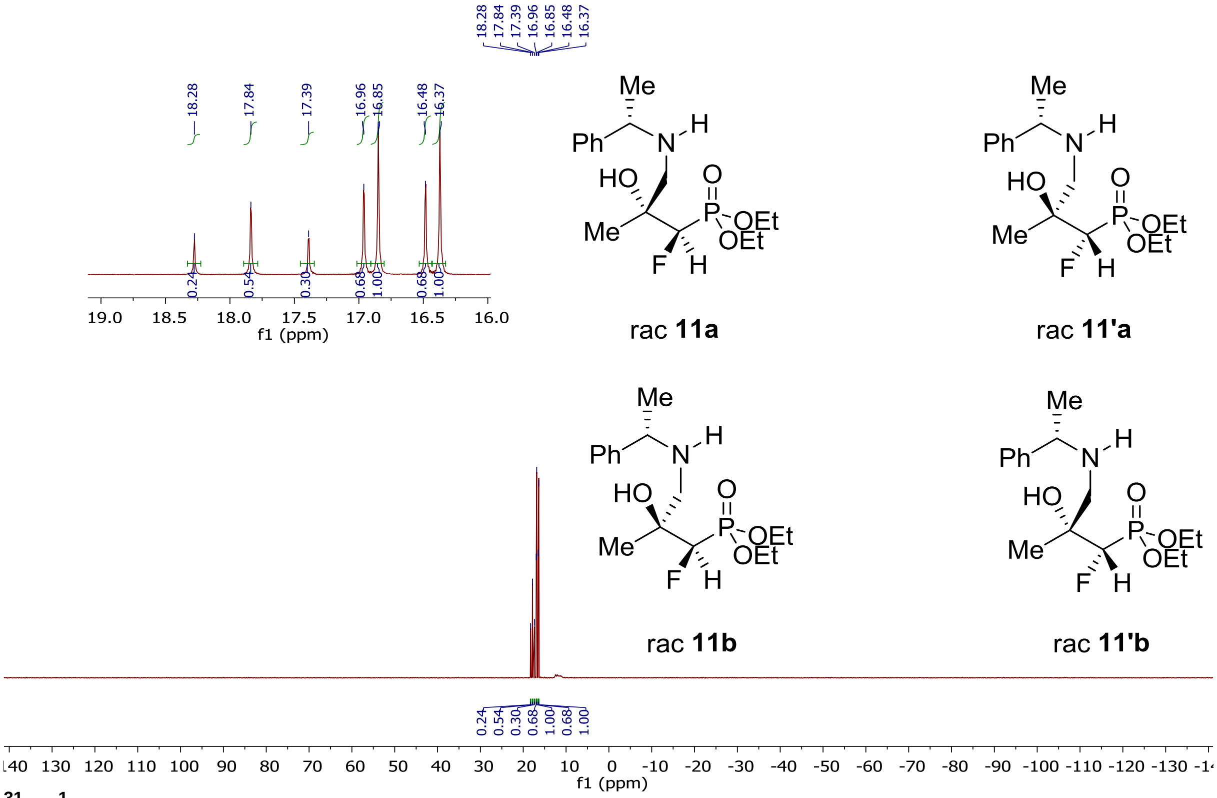

^{31}P{/^{1}H} NMR of rac 11a,b/11’a,b (5:4/1.5:1,, d.r.)

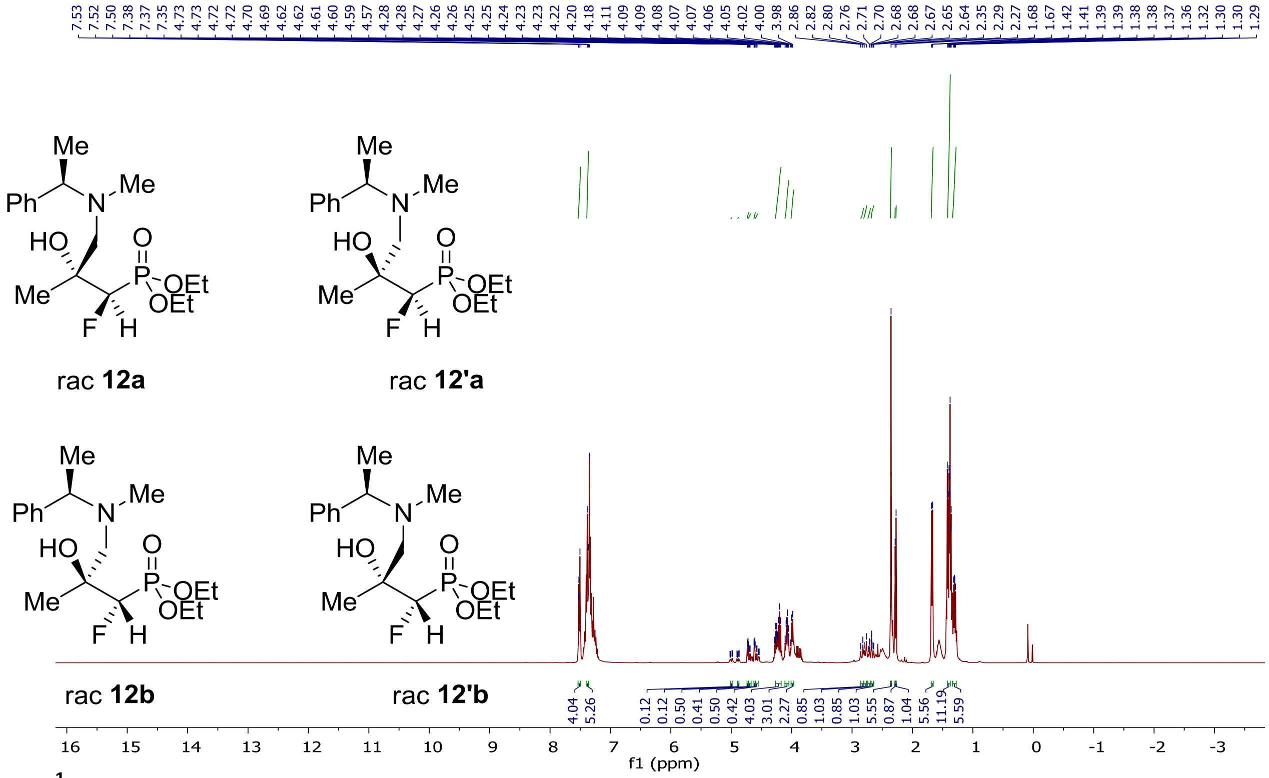

^{1}H NMR of rac **12a:12b12’a:12’b** (17:14/1:1, d.r.)

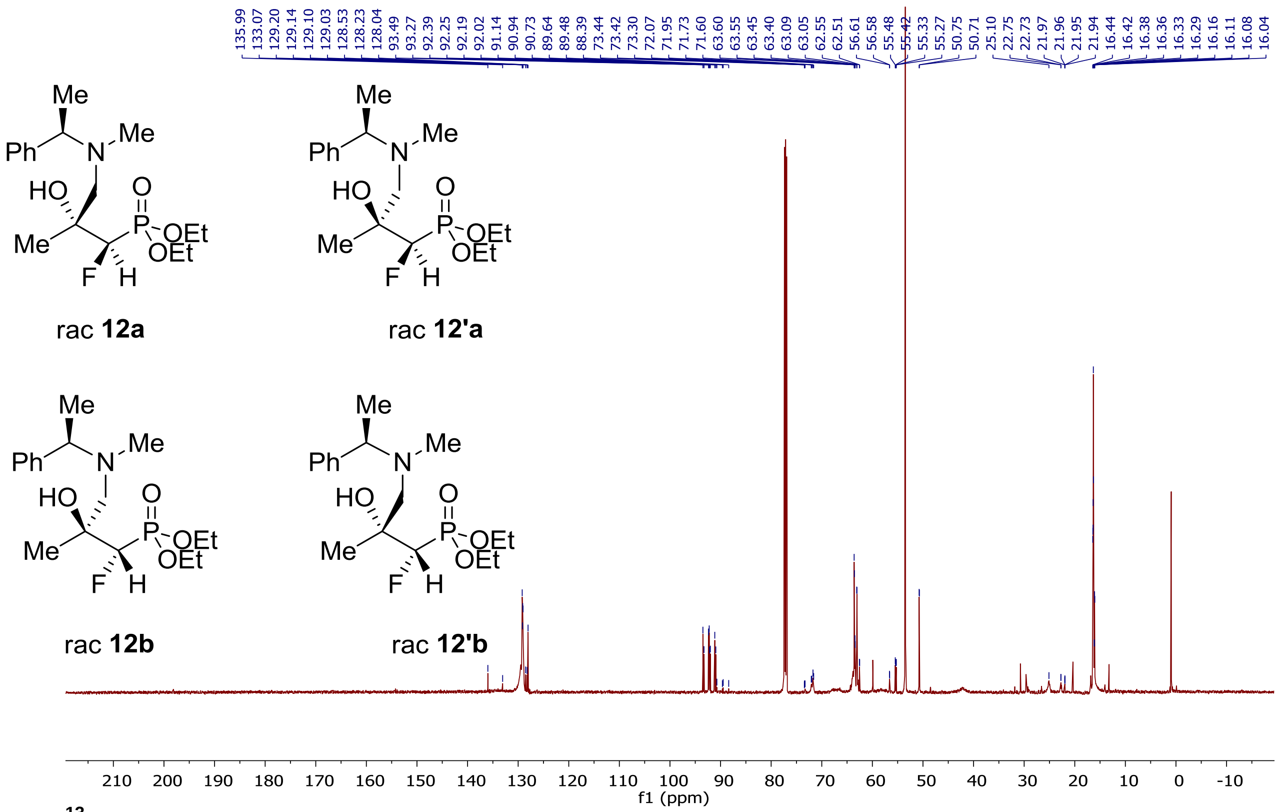

^{13}**C NMR of** rac **12a:12b12'a:12'b** (17:14/1:1, d.r.)

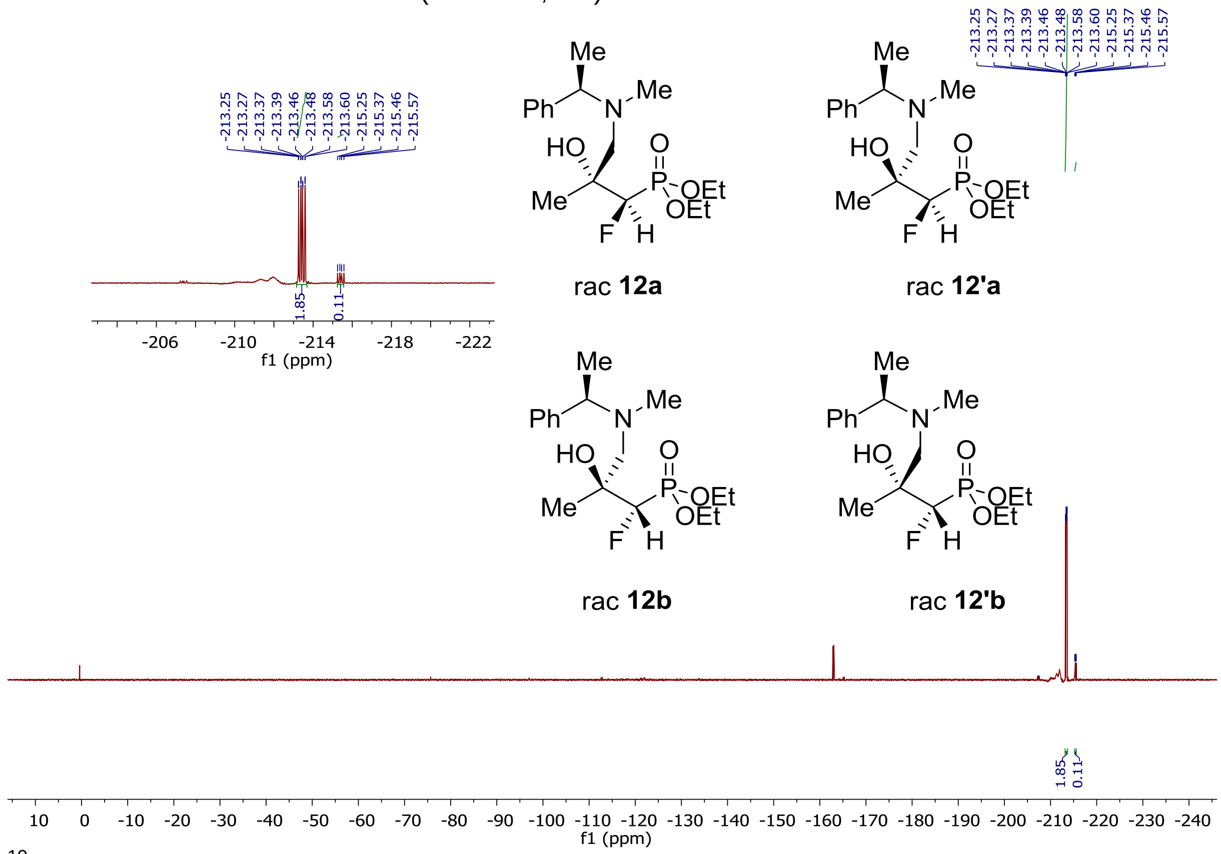

^{19}F NMR of **rac** 12a:12b12'a:12'b **(17:14/1:1, d.r.)**

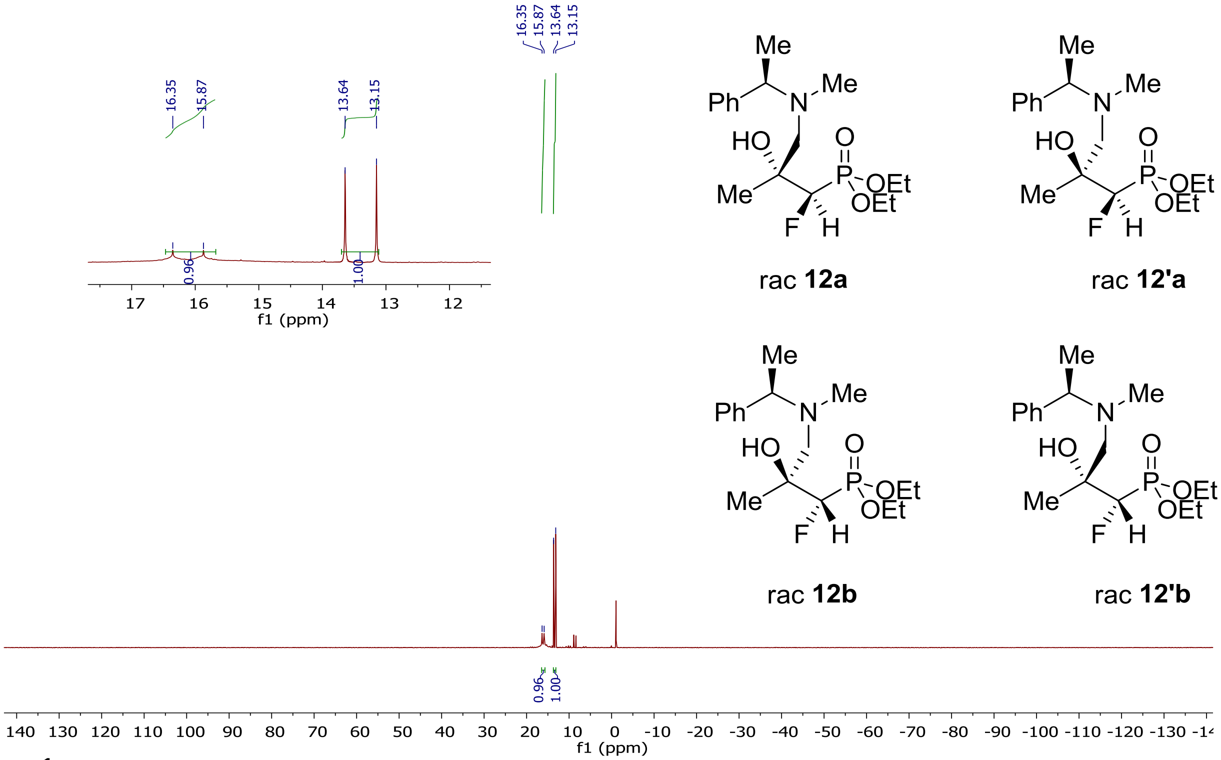

P{*l*^{1}H} NMR of rac **12a:12b12’a:12’b** (17:14/1:1, d.r.)

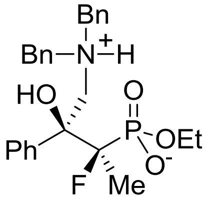

rac **17**

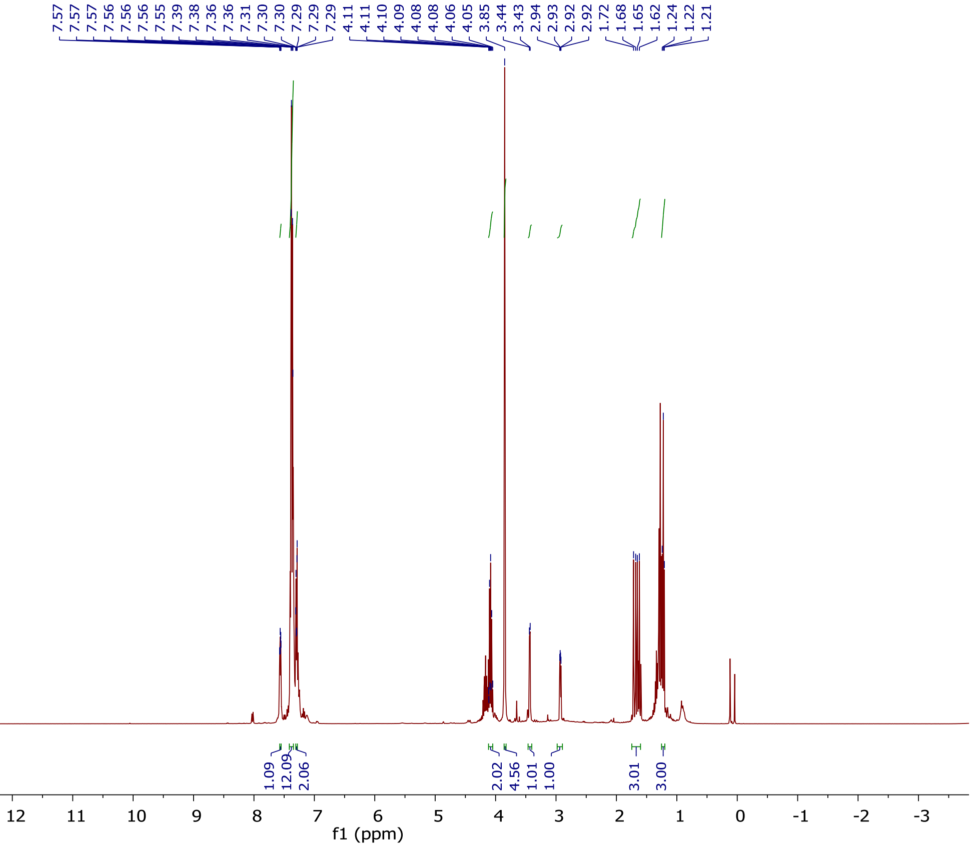

^{1}H NMR of rac **17**

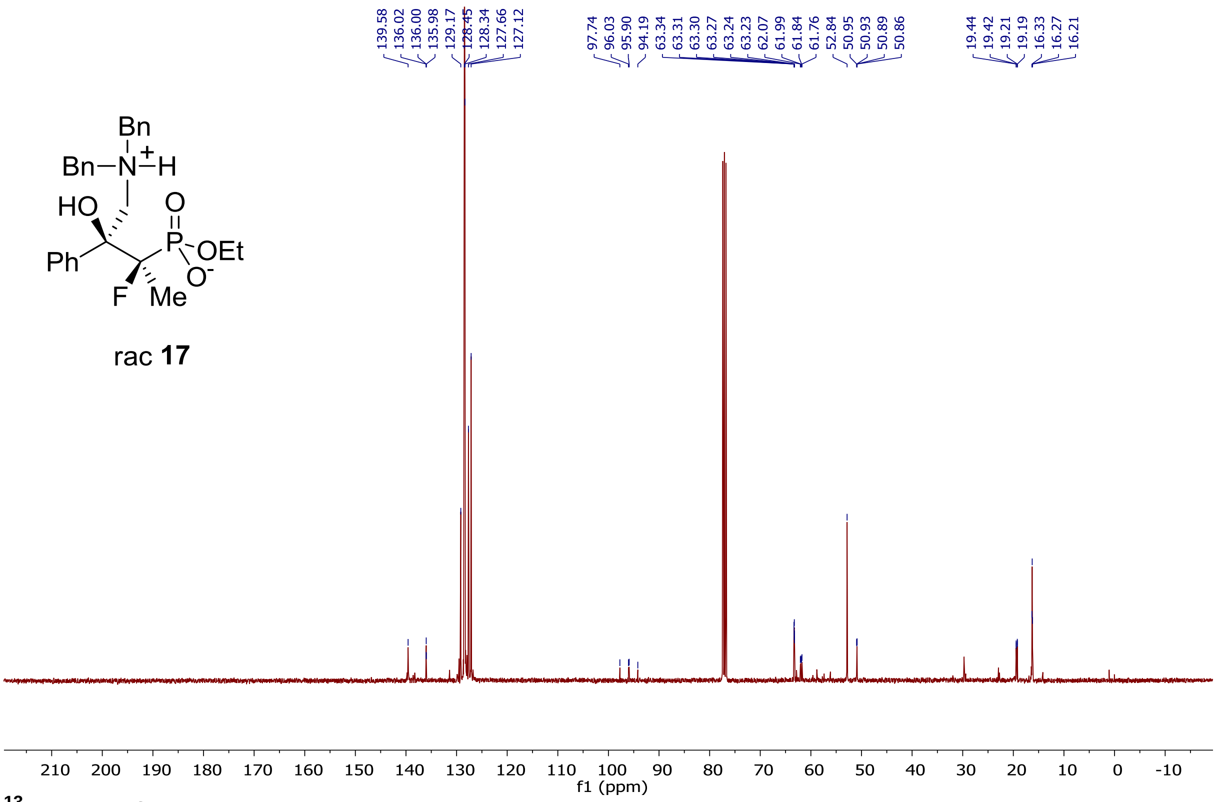

^{13}C NMR of rac 17

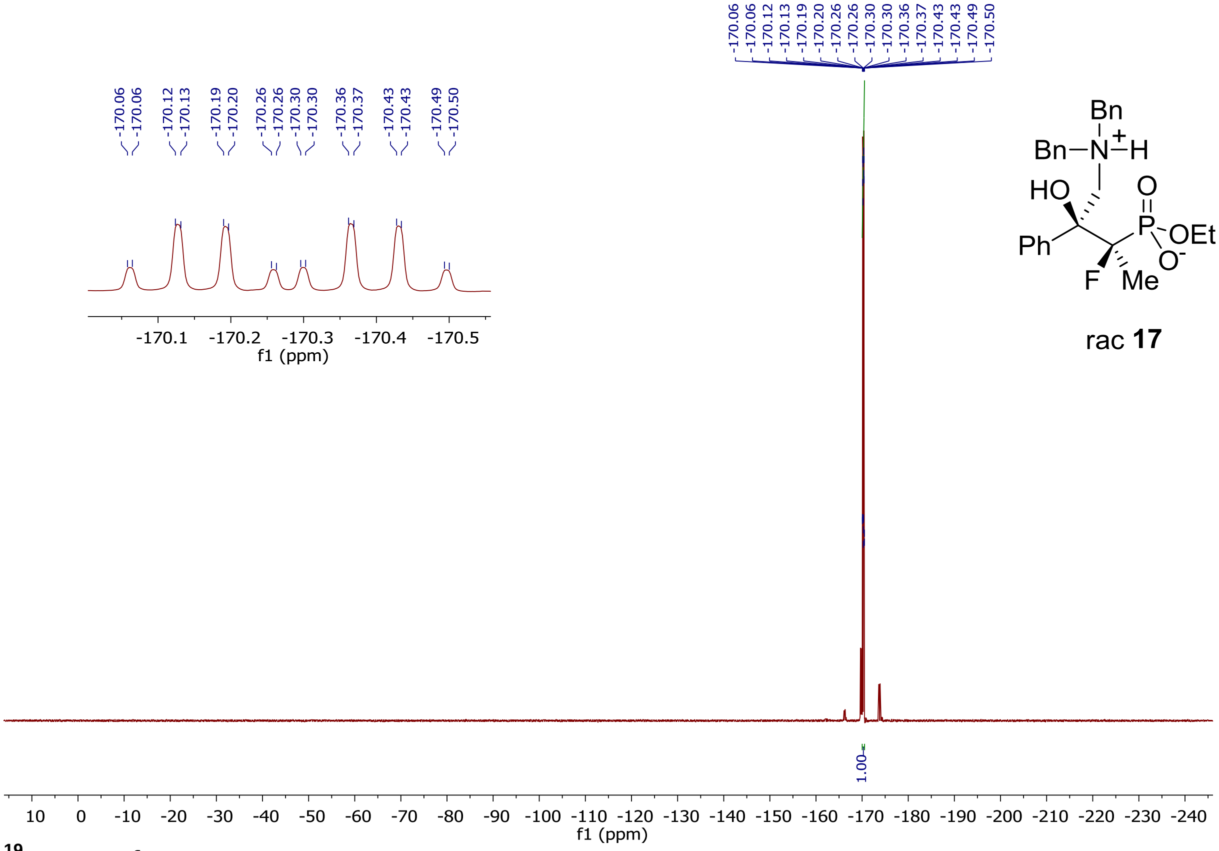

^{19}F NMR of rac 17

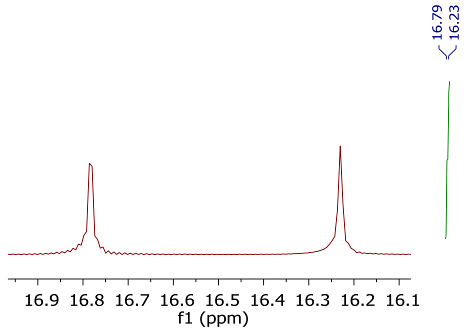

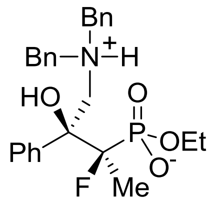

rac **17**

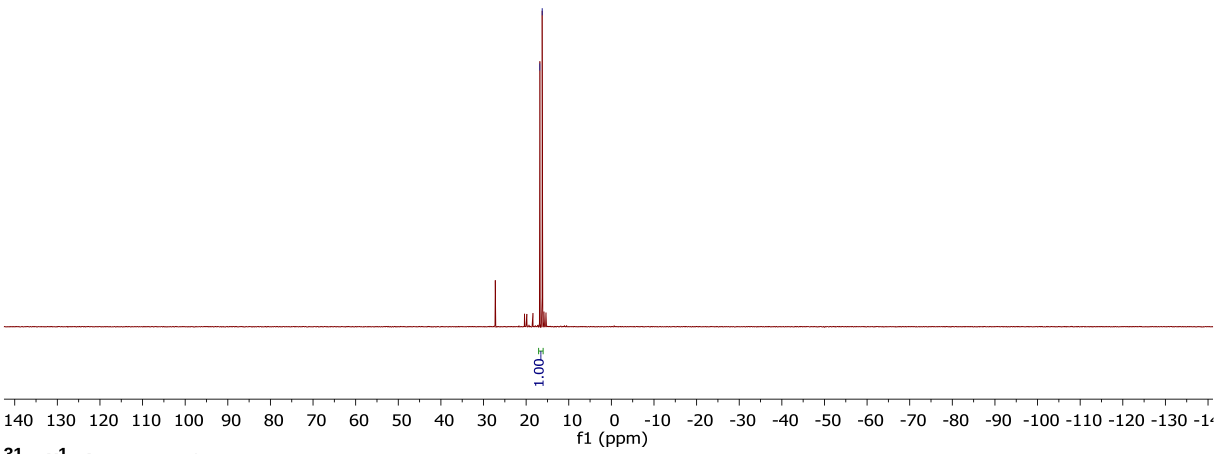

^{31}P{/^{1}H} NMR of rac **17**

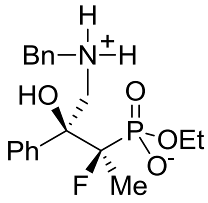

rac **18**

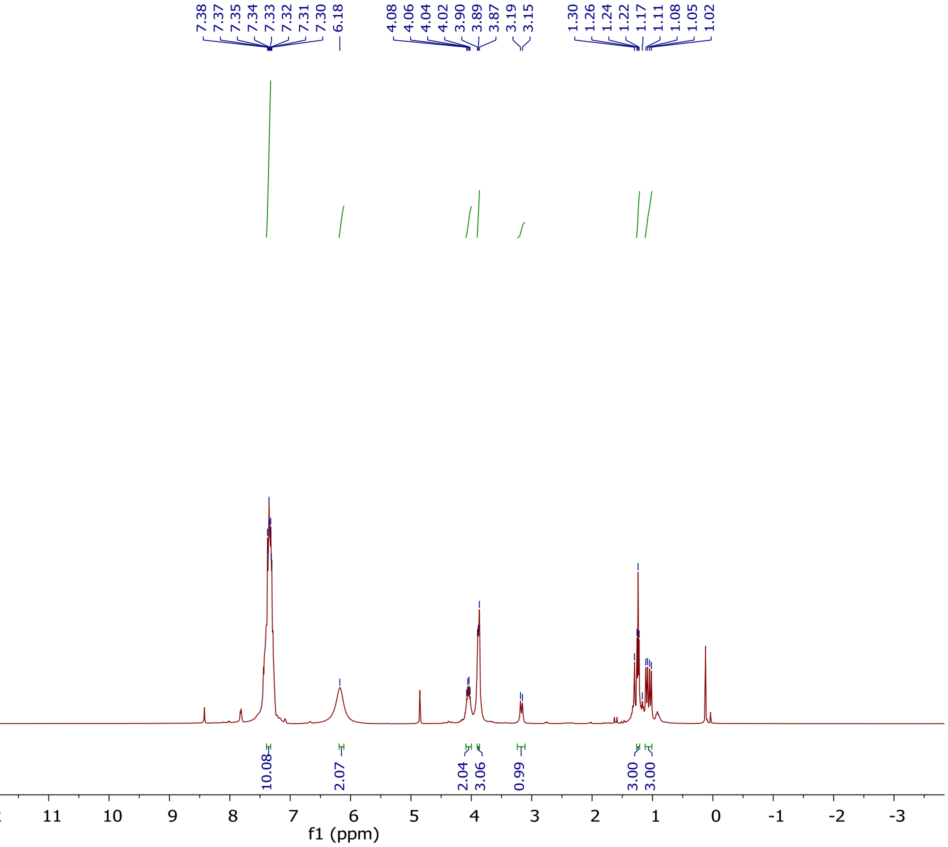

^{1}H NMR of rac **18**

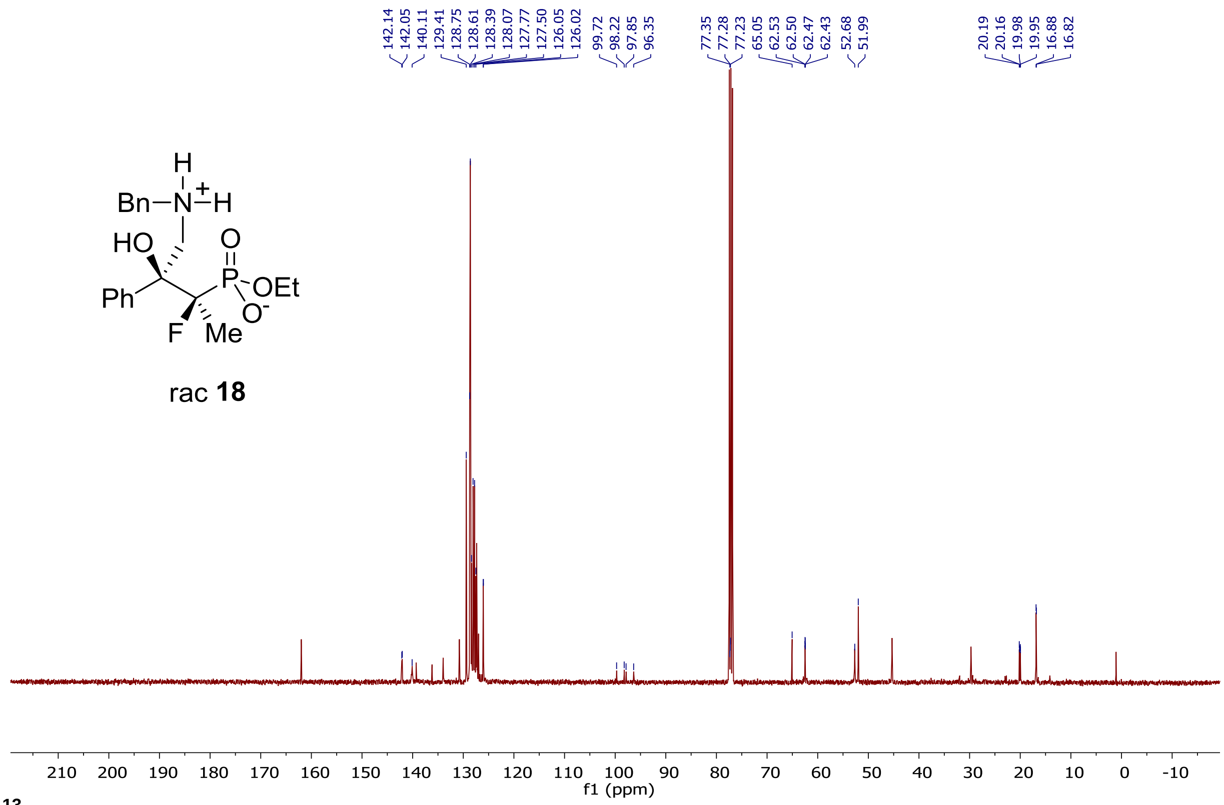

rac **18**

^{13}C NMR of rac **18**

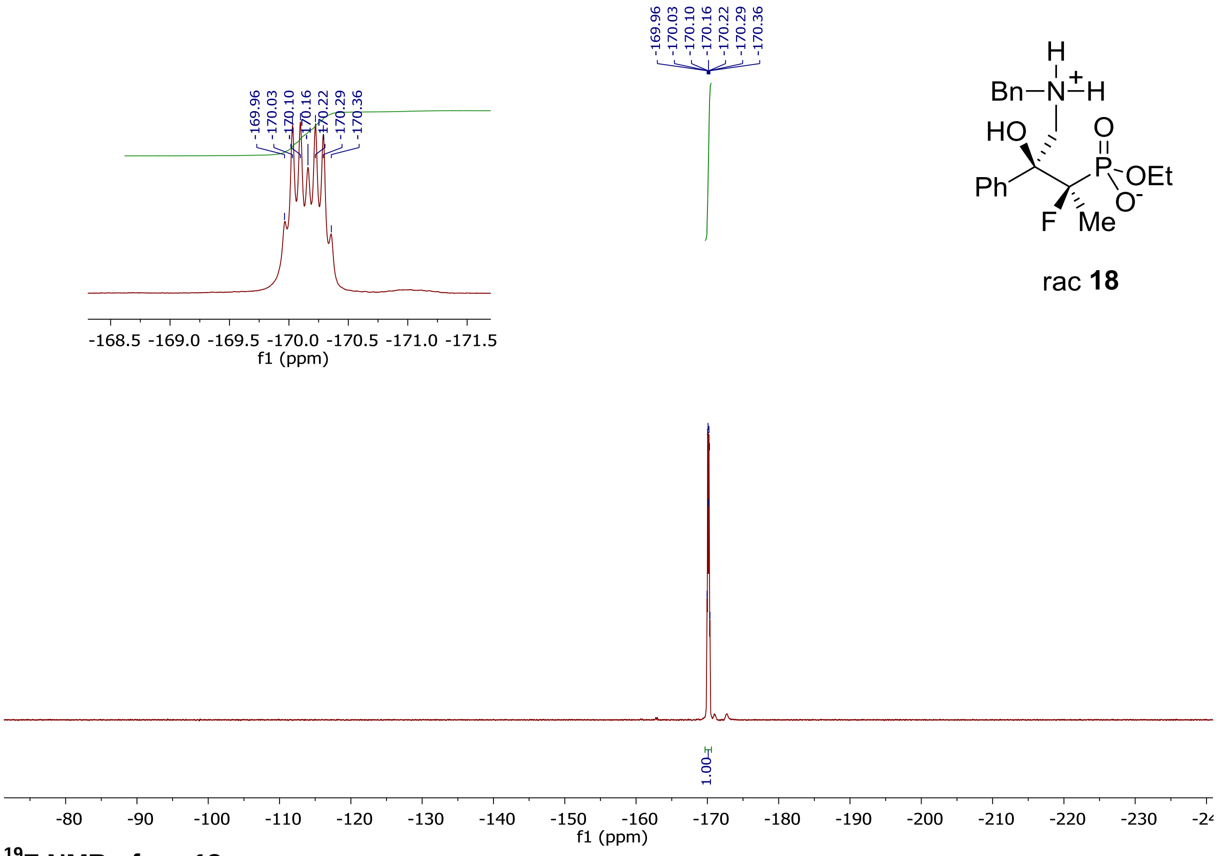

rac **18**

^{19}F NMR of rac **18**

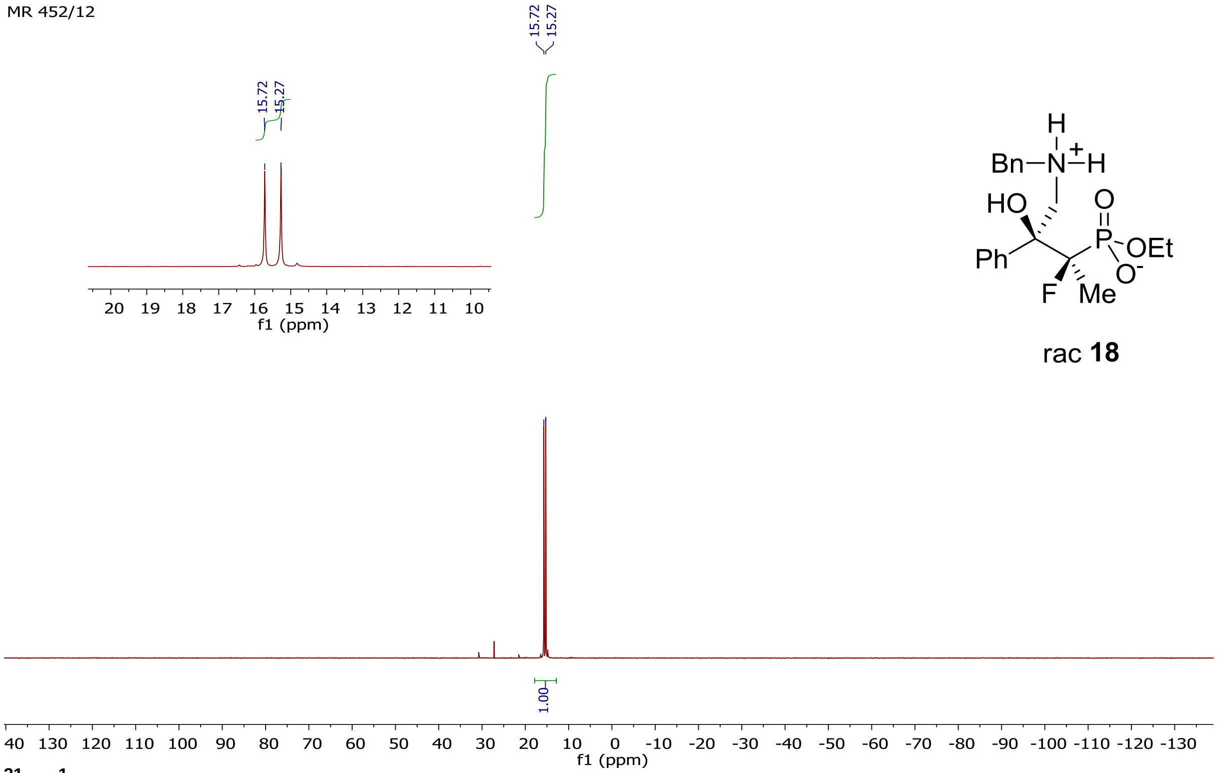

rac **18**

^{31}P{/^{1}H} NMR of rac **18**

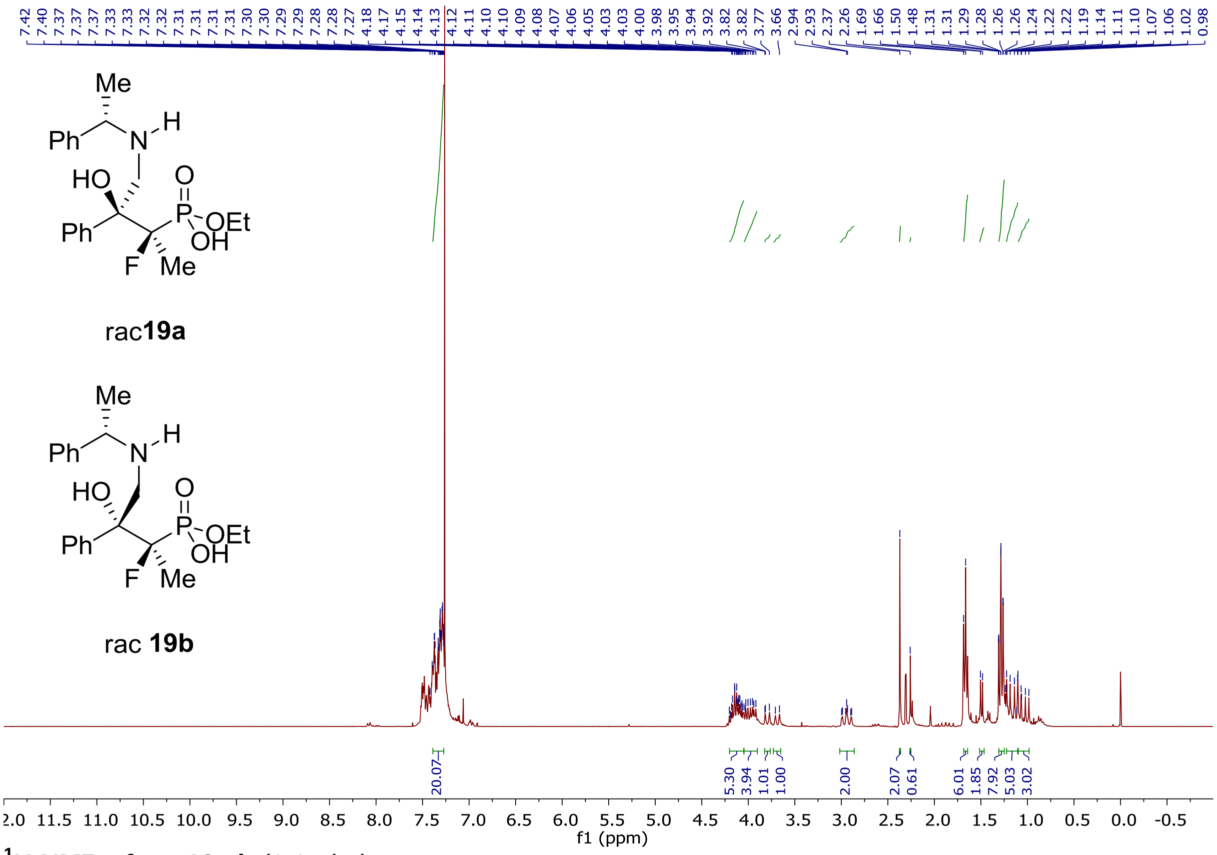

^{1}H NMR of rac **19a,b** (1:1, d.r.)

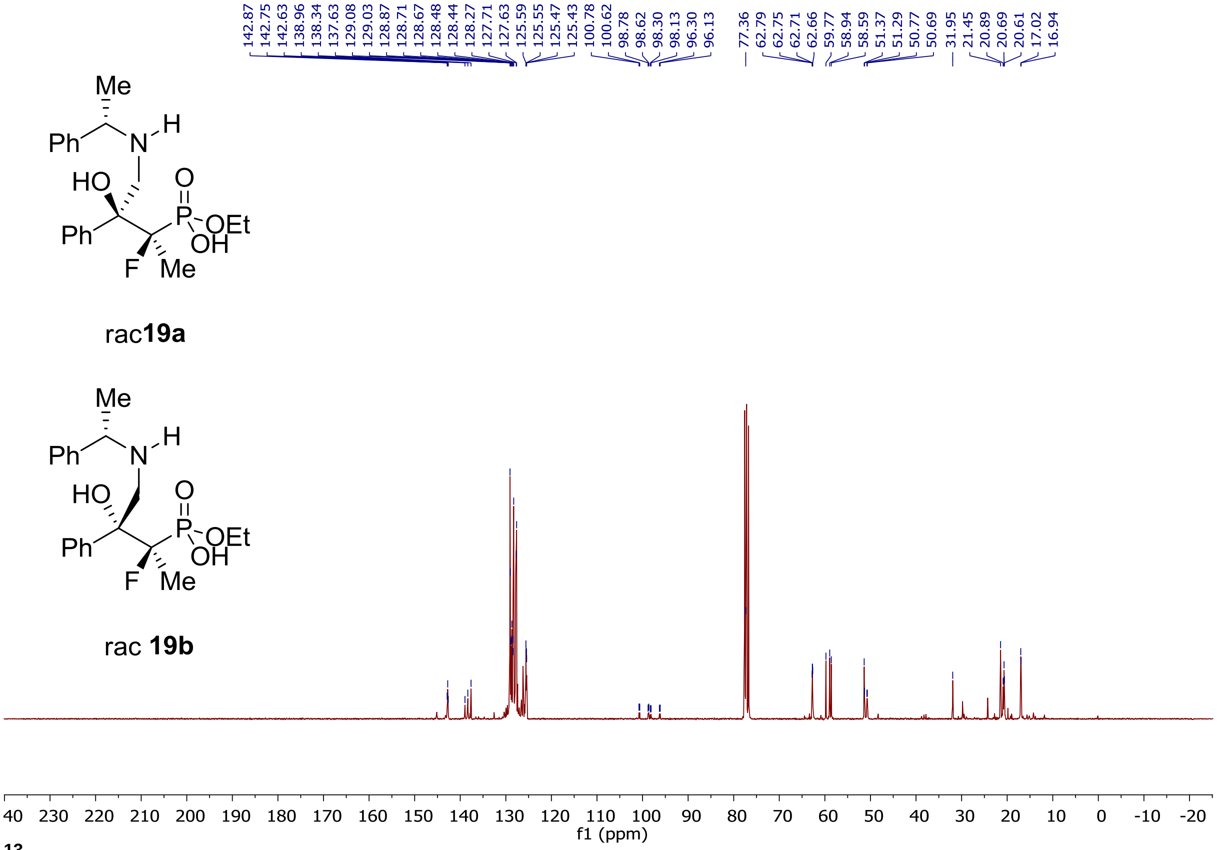

^{13}C NMR of rac **19a,b** (1:1, d.r.)

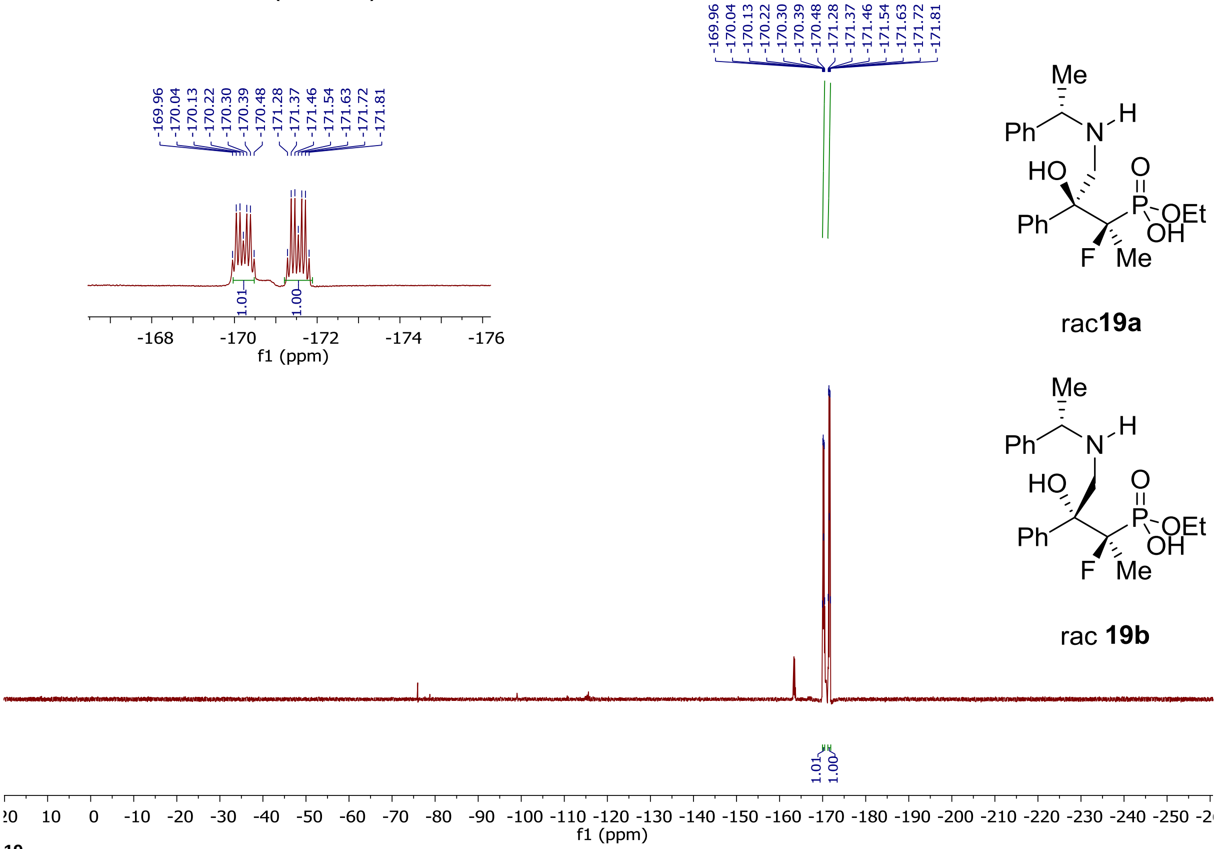

^{19}F NMR of rac **19a,b** (1:1, d.r.)

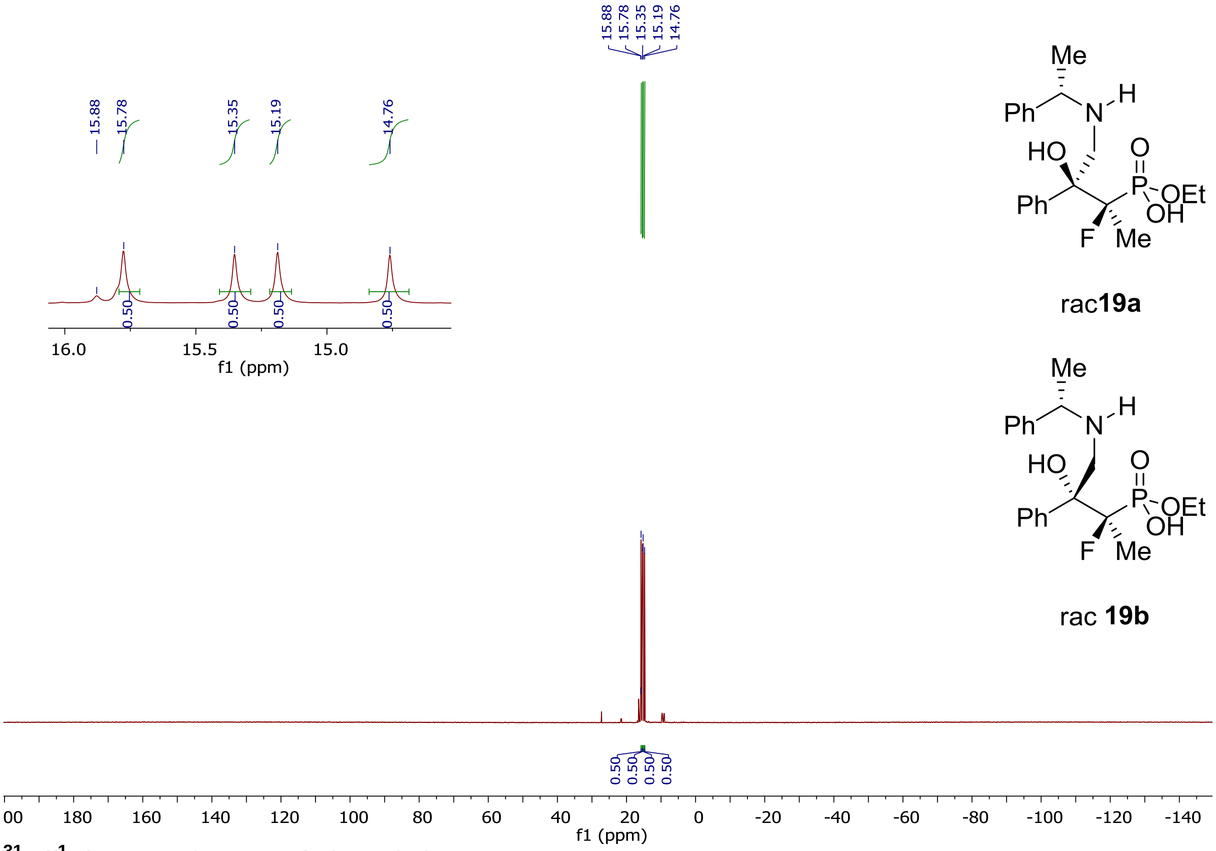

rac**19a**

rac **19b**

^{31}**P{/^{1}H} NMR of** rac **19a,b** (1:1, d.r.)

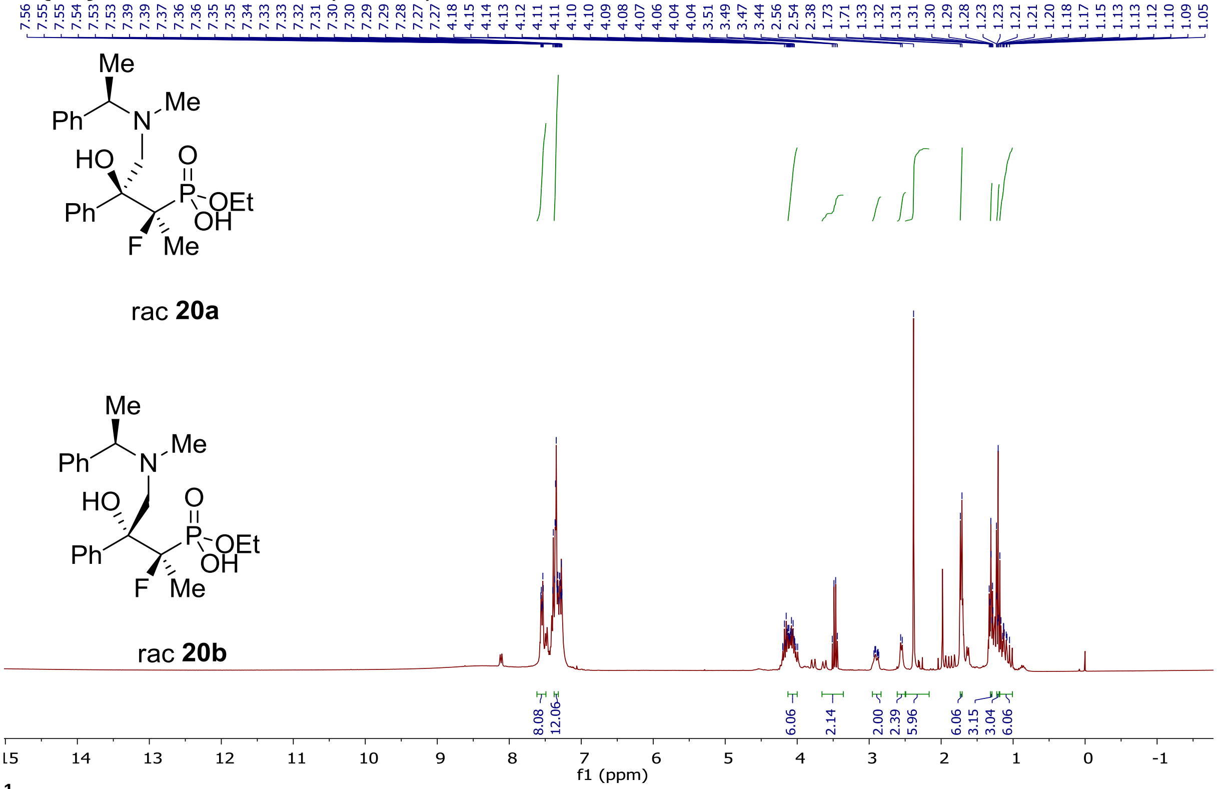

rac **20a**

rac **20b**

^{1}**H NMR of** rac **20a,b** (1:1, d.r.)

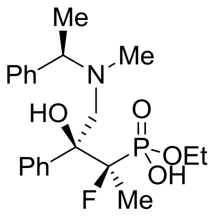

rac **20a**

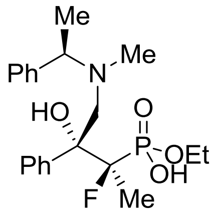

rac **20b**

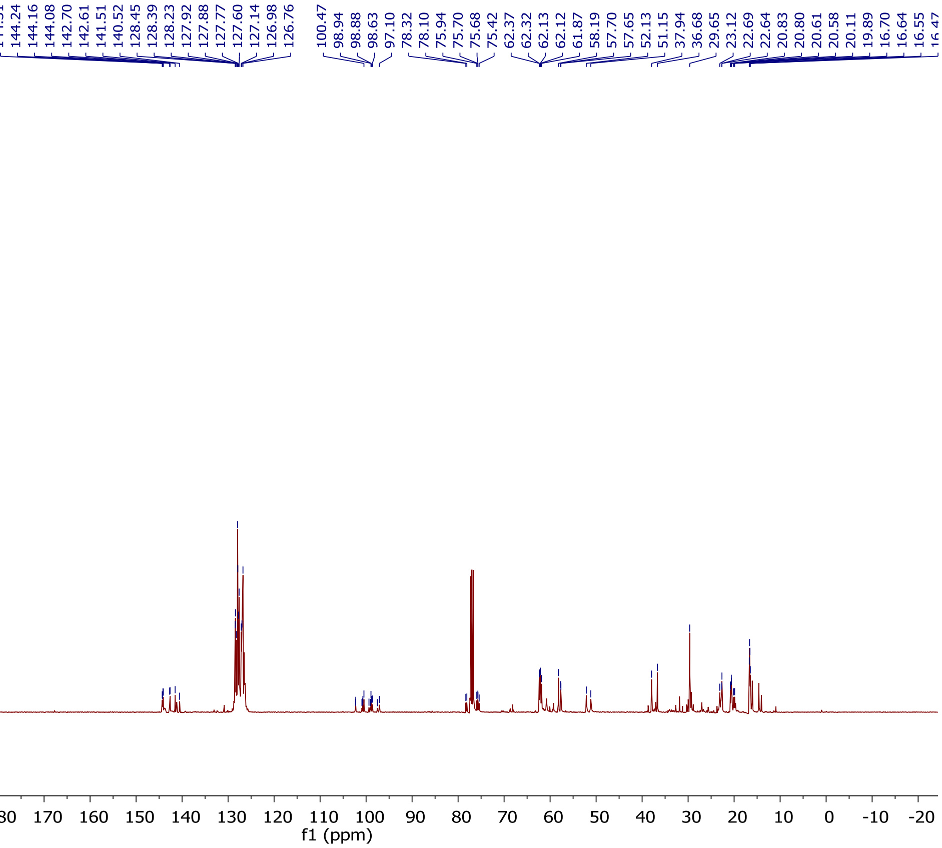

^{13}C NMR of rac **20a,b** (1.1:1, d.r.)

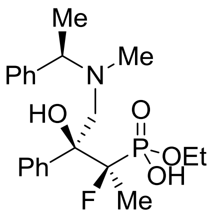

rac **20a**

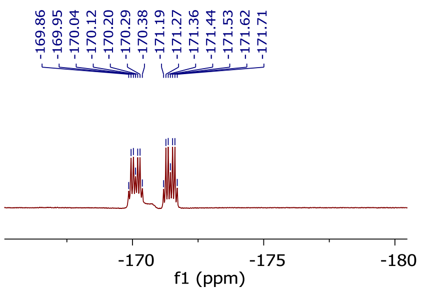

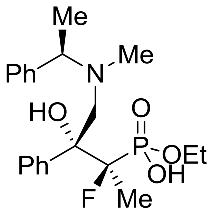

rac **20b**

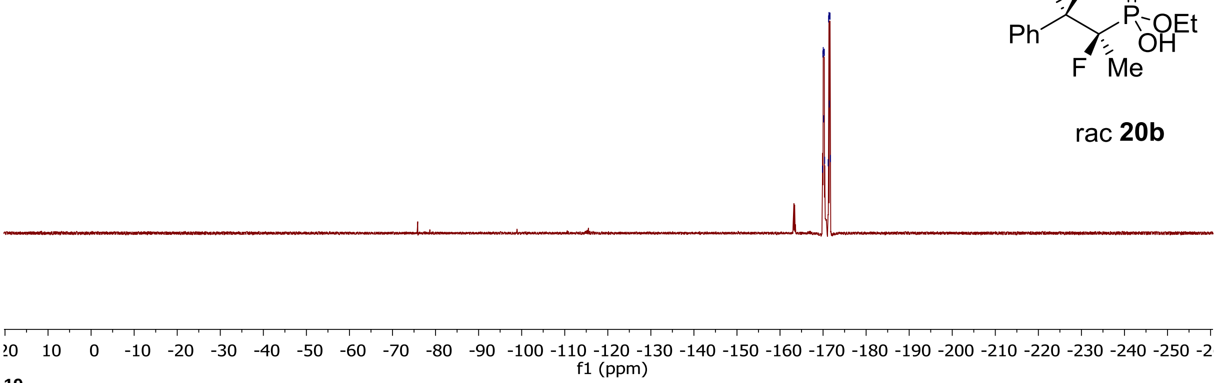

^{19}F NMR of rac **20a,b** (1.1:1, d.r.)

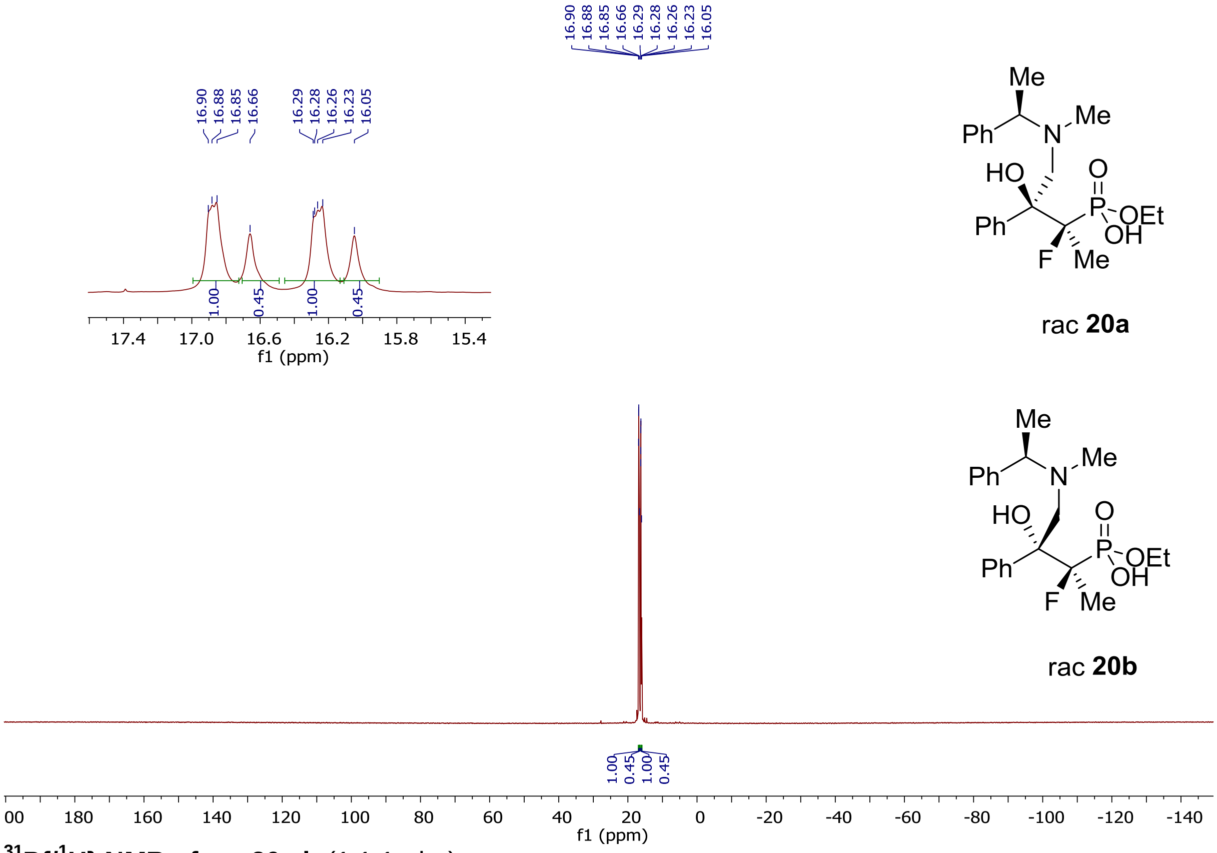

^{31}P{/^{1}H} NMR of rac **20a,b** (1.1:1, d.r.)

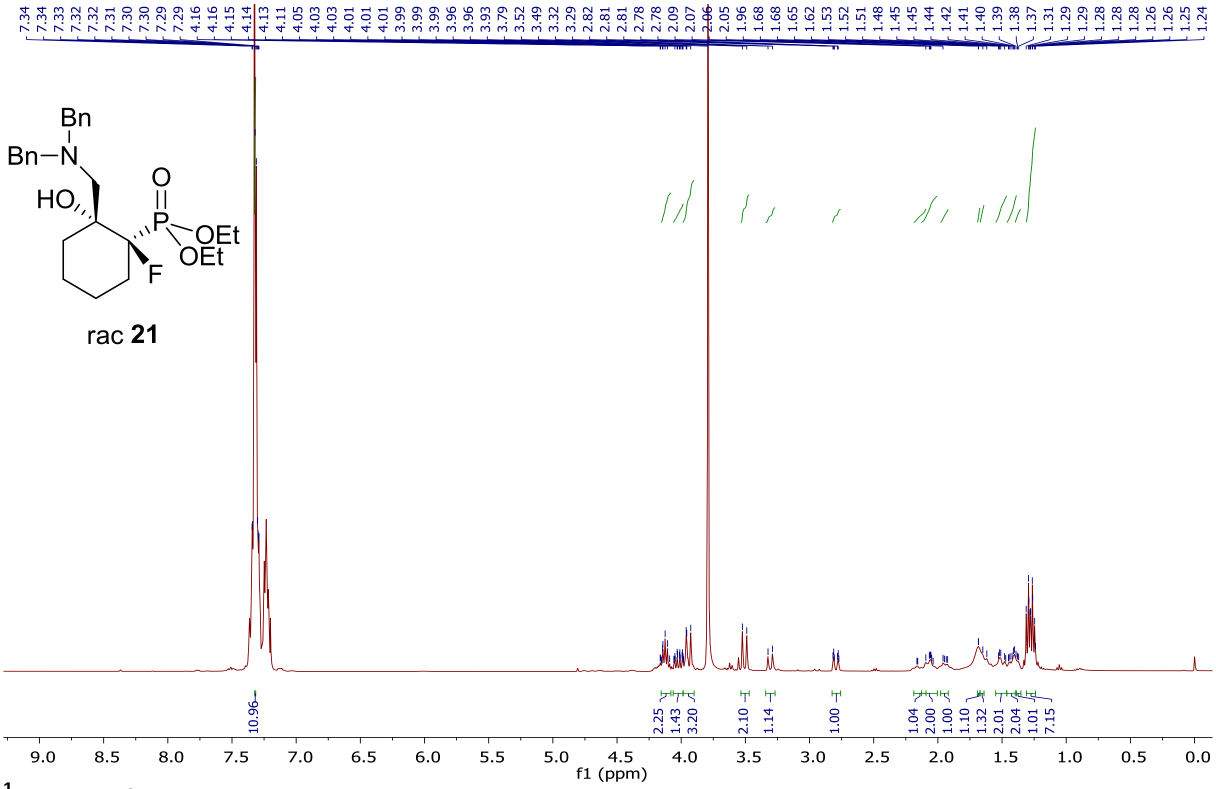

^{1}H NMR of rac **21**

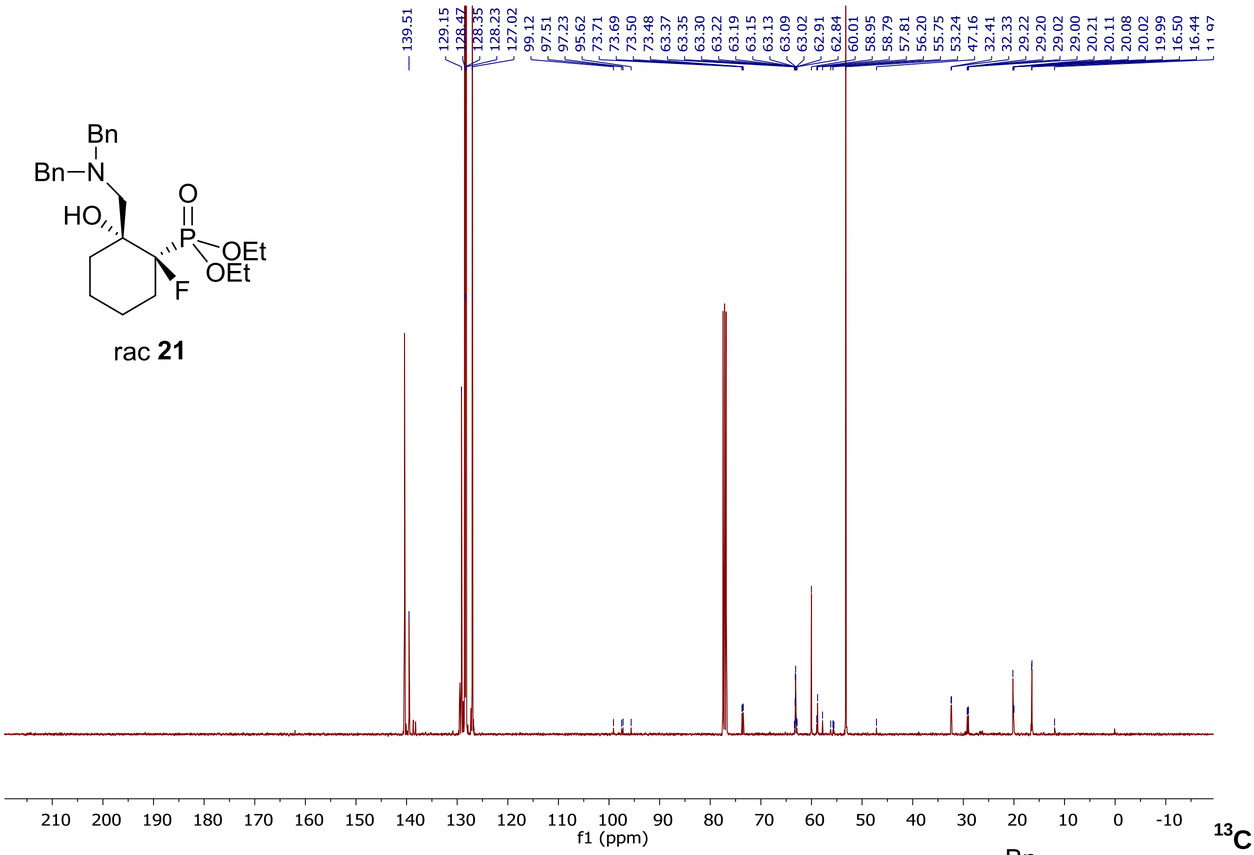

^{13}C NMR of rac **21**

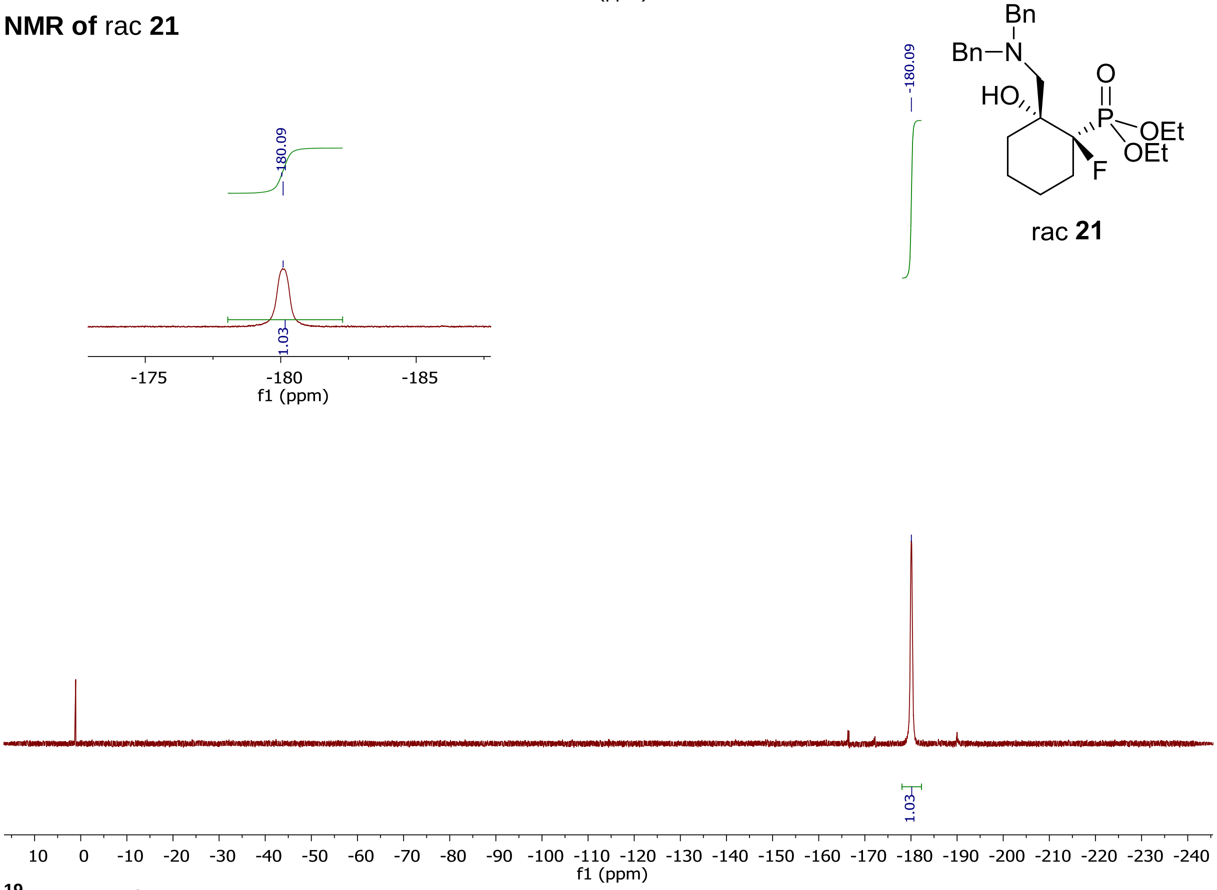

^{19}F NMR of rac **21**

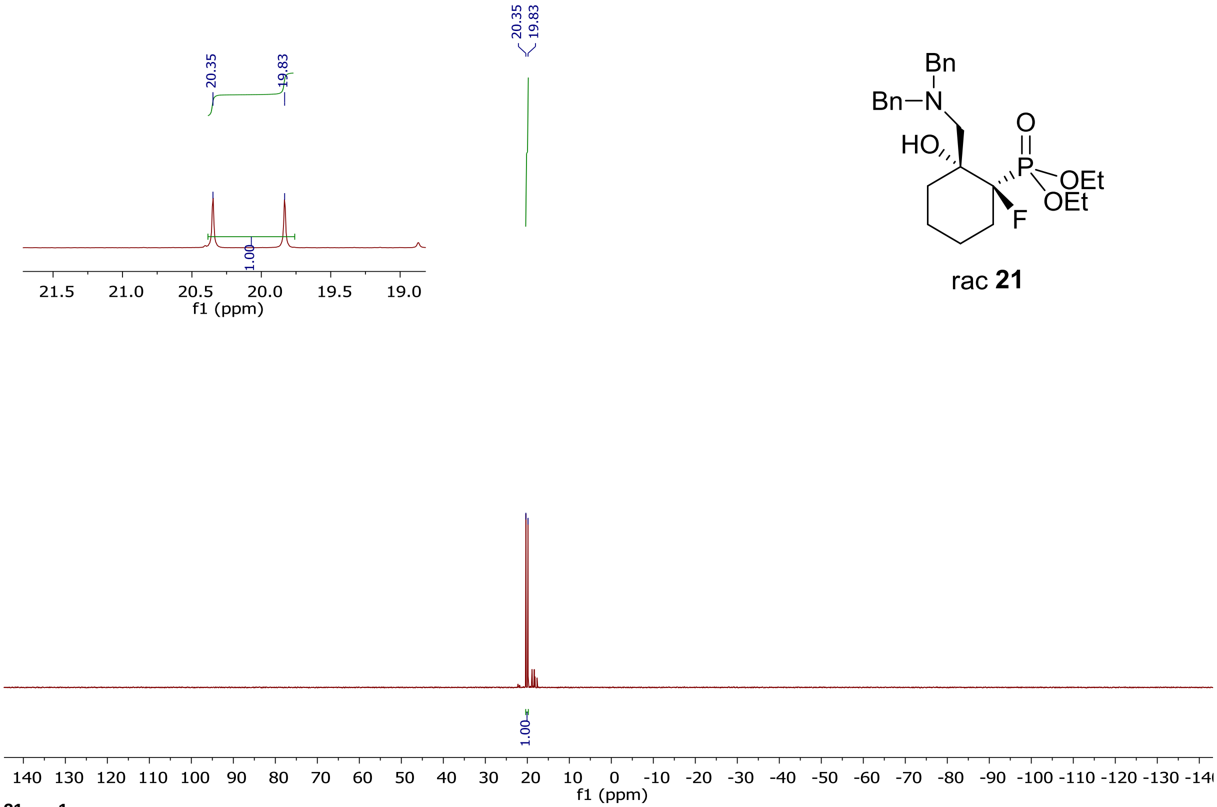

rac **21**

^{31}P{/^{1}H} NMR of rac **21**

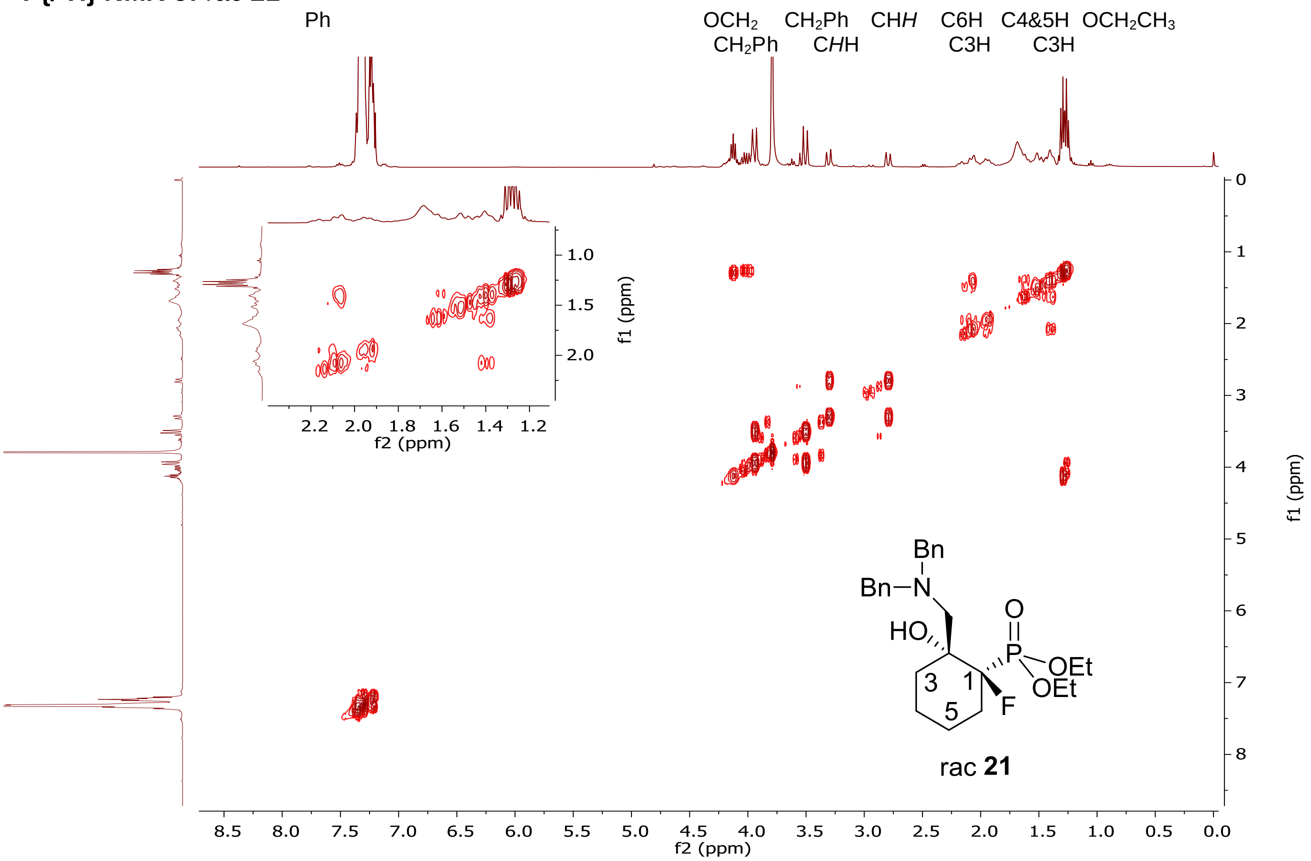

COSY of rac **21**

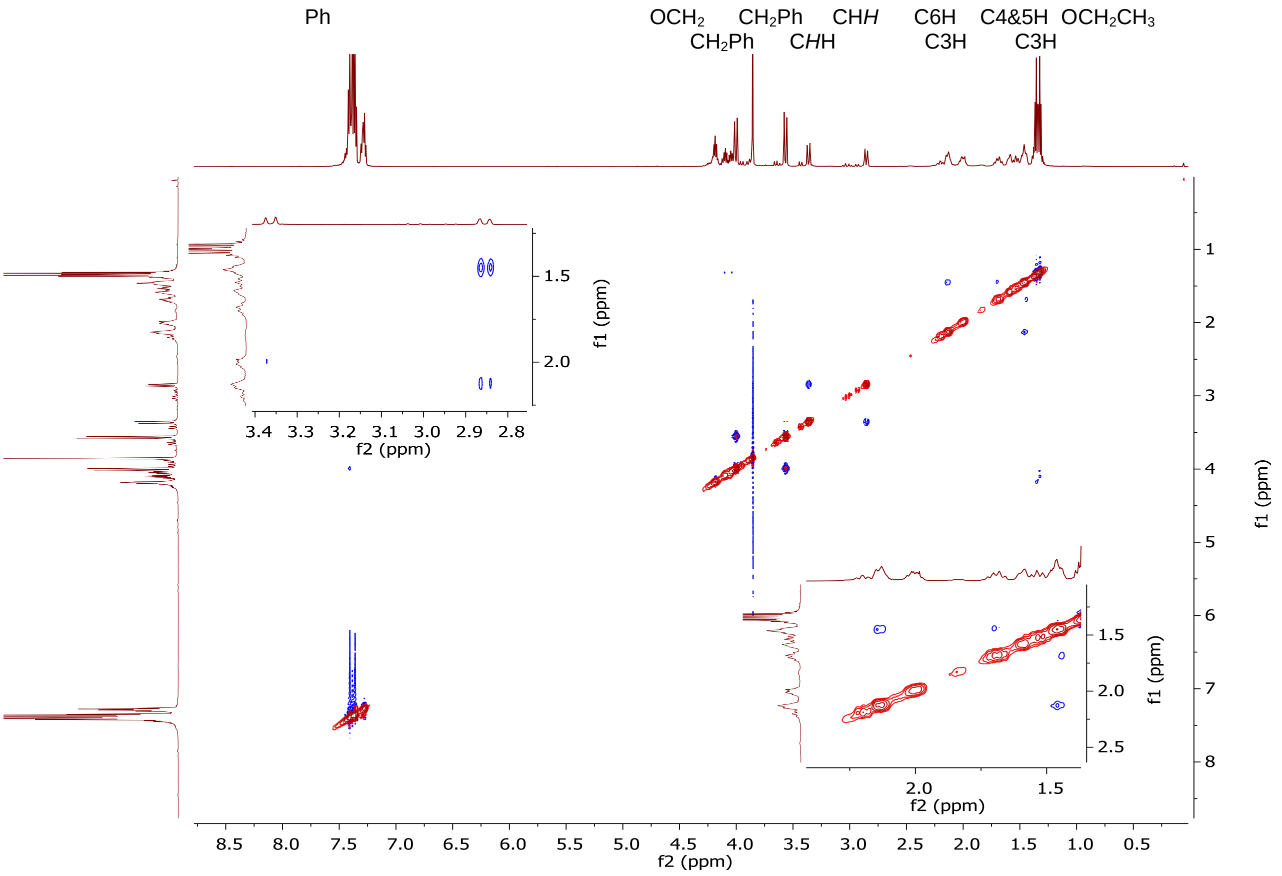

2D NOESY of rac **21**

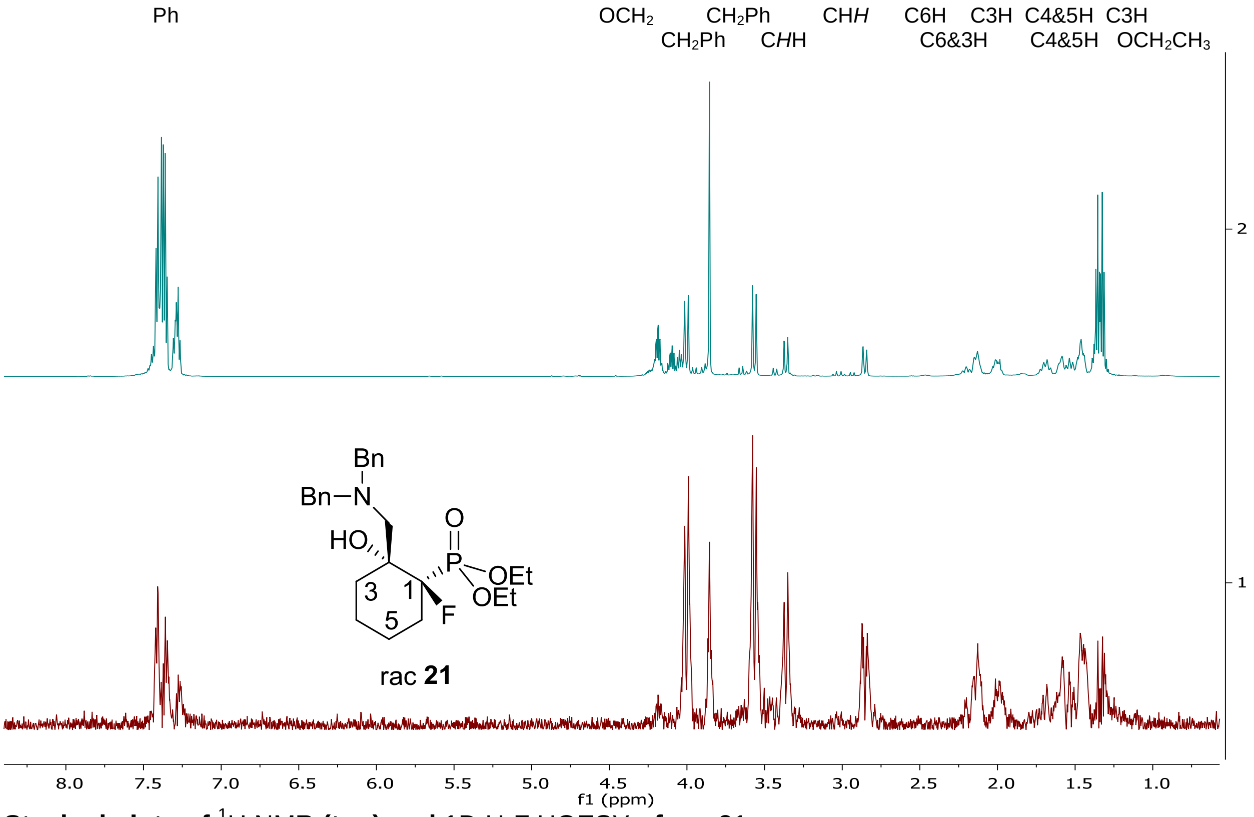

Stacked plots of ^{1}H NMR **(top) and** 1D H-F HOESY **of rac** 21

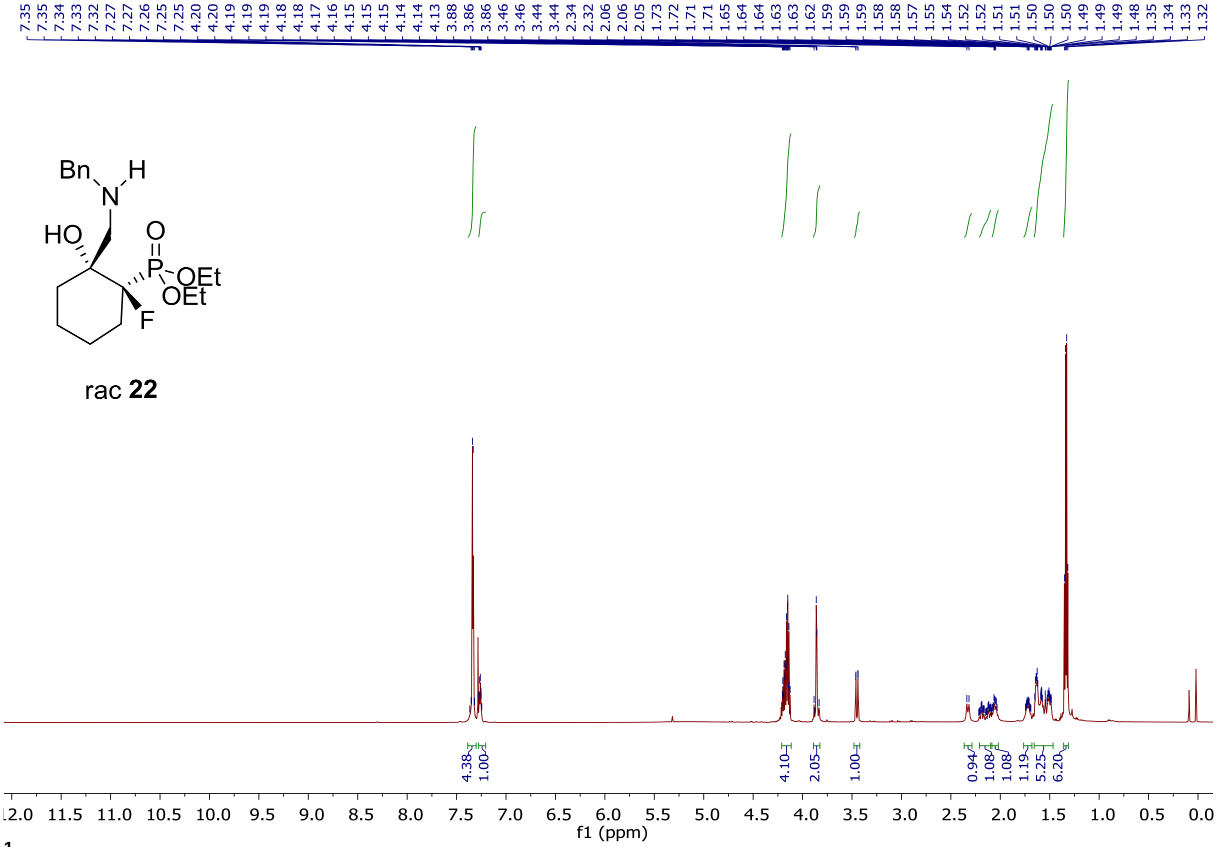

^{1}H NMR of rac **22**

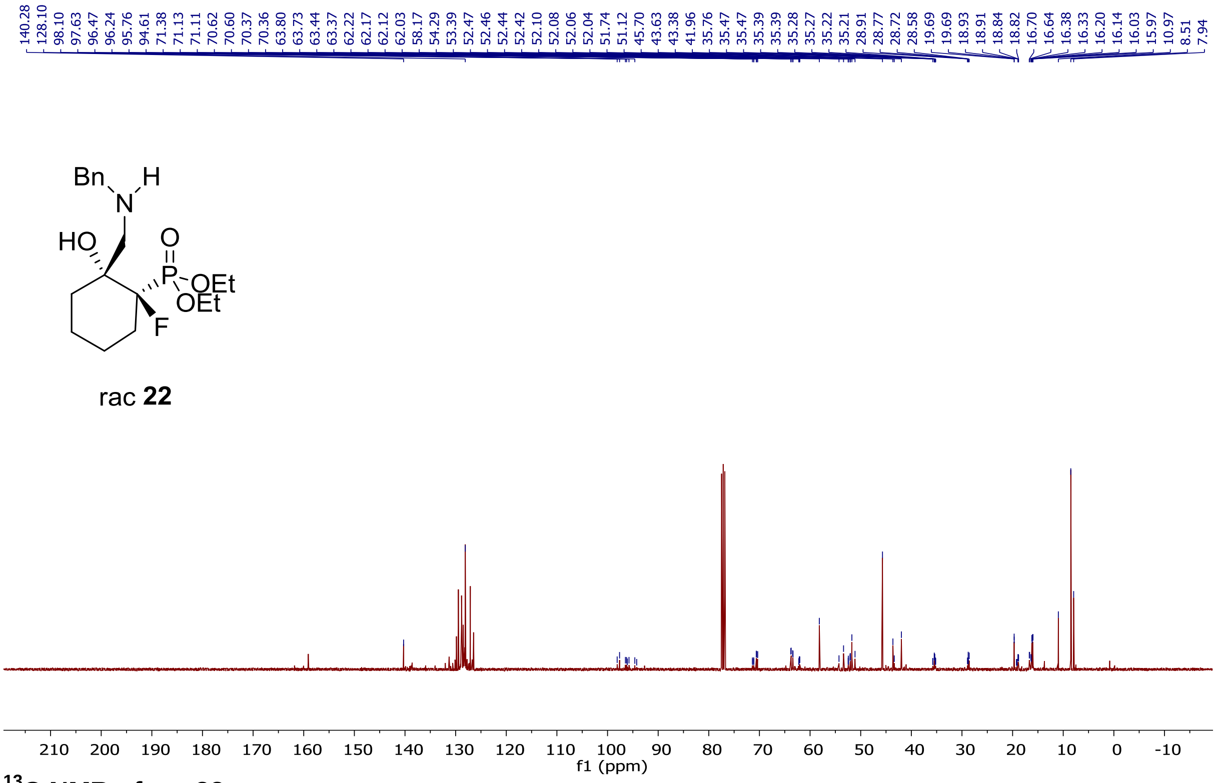

^{13}C NMR of rac **22**

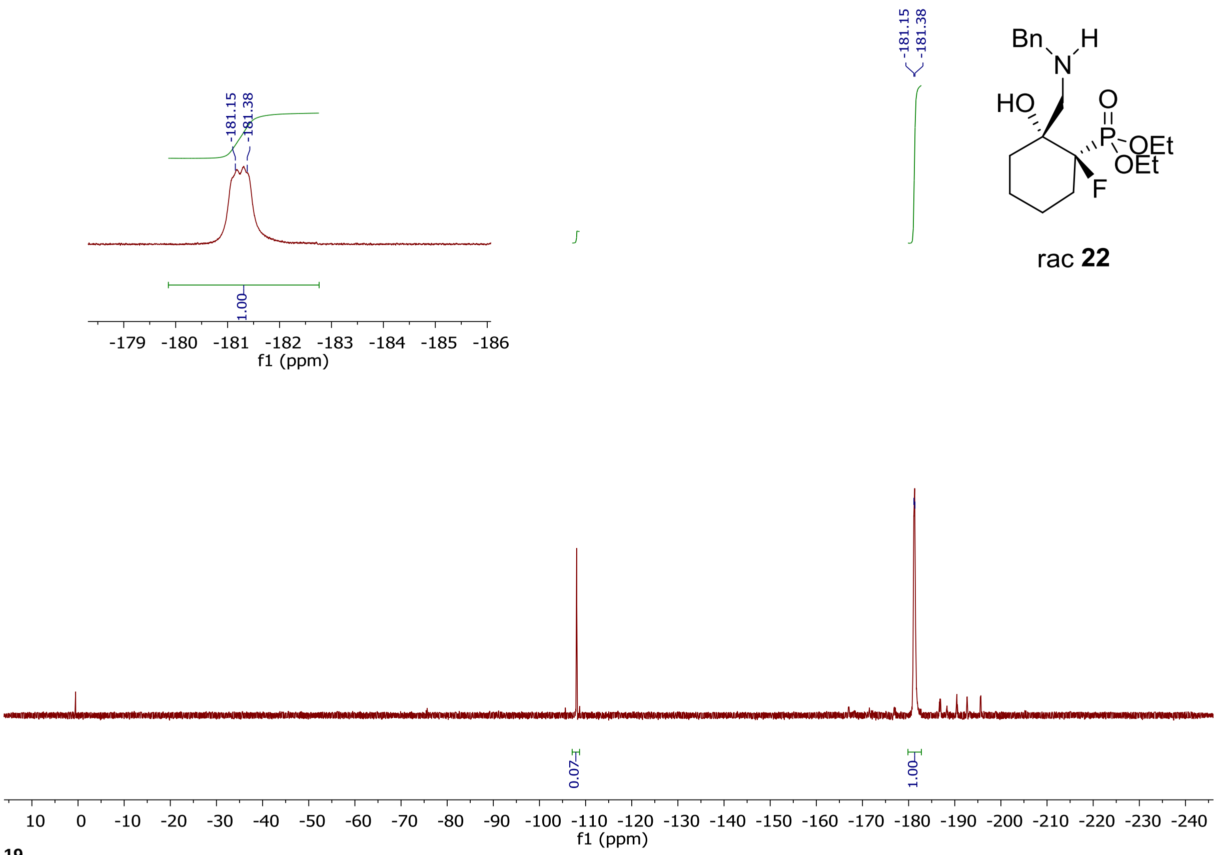

^{19}**F NMR of** rac **22**

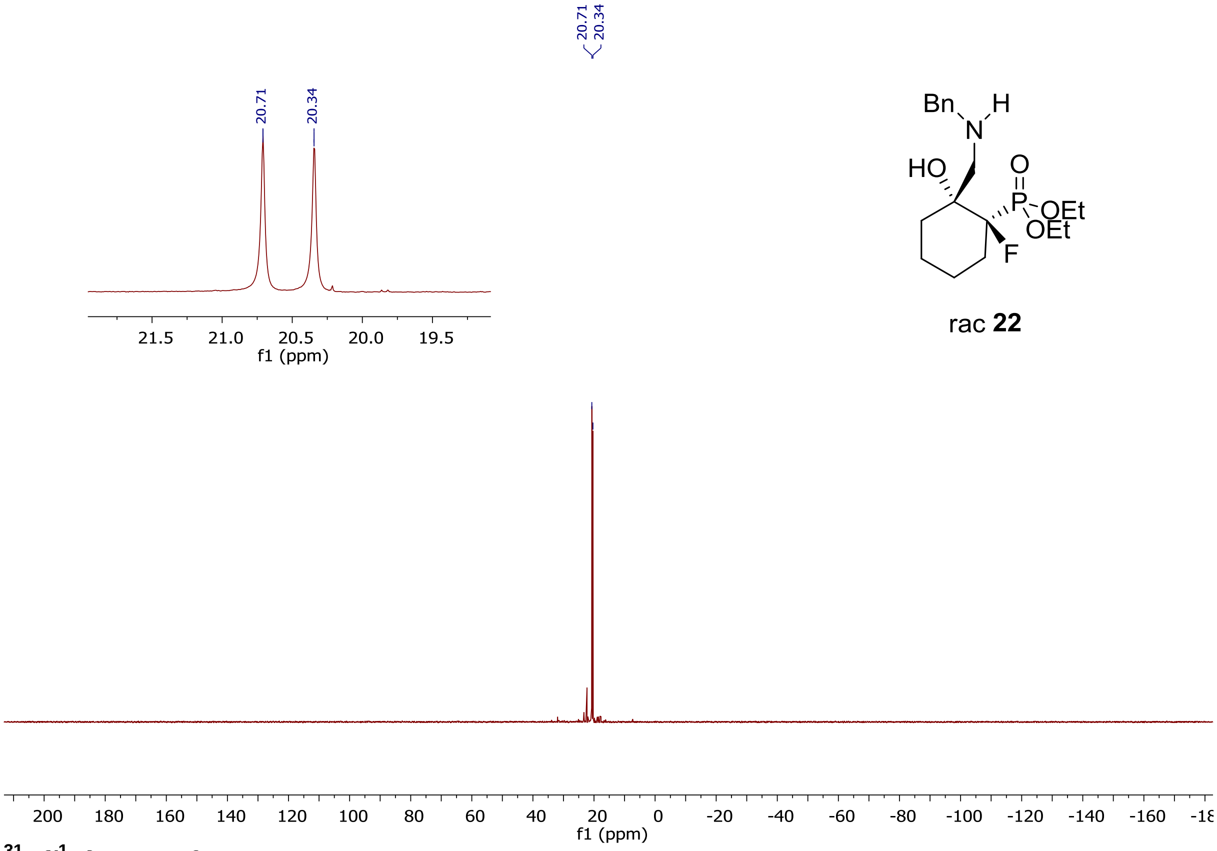

^{31}**P{/^{1}H} NMR of** rac **22**

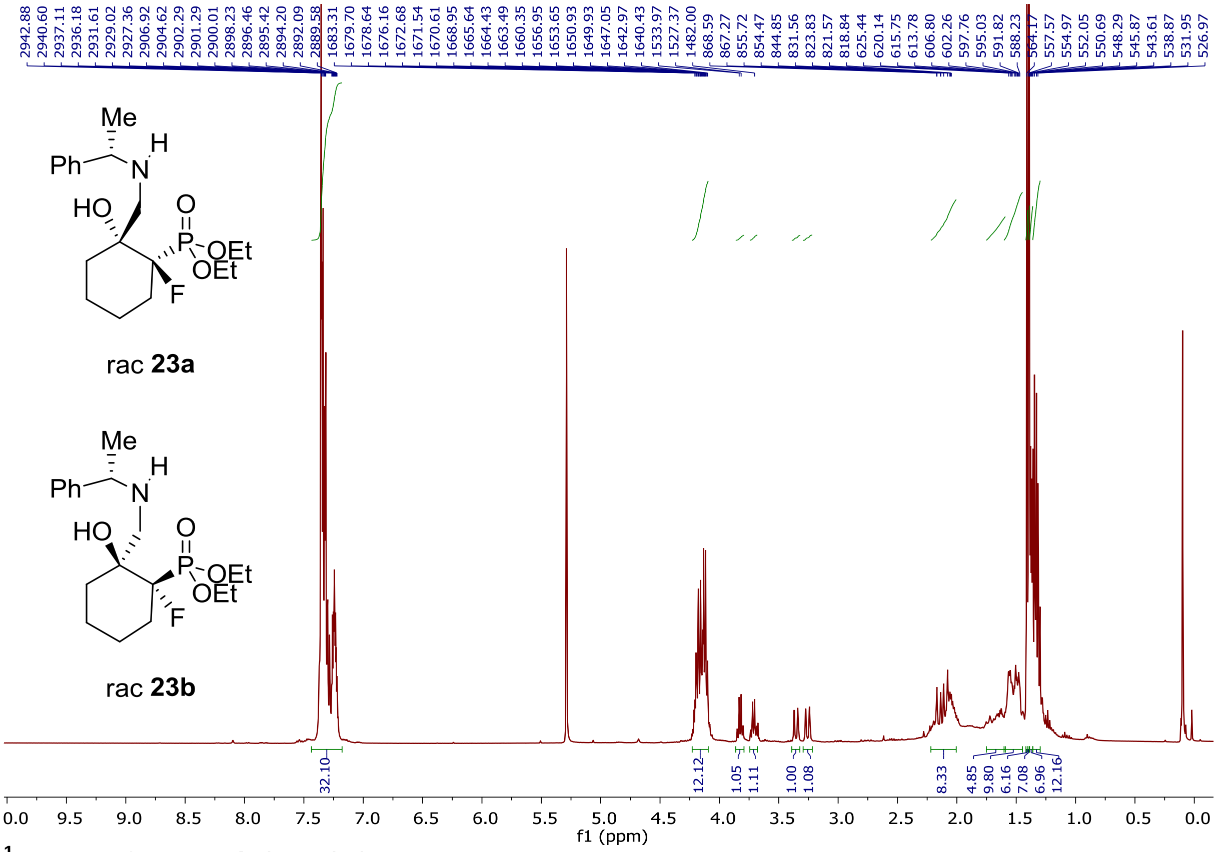

^{1}H NMR of rac **23a,b** (1:1, d.r.)

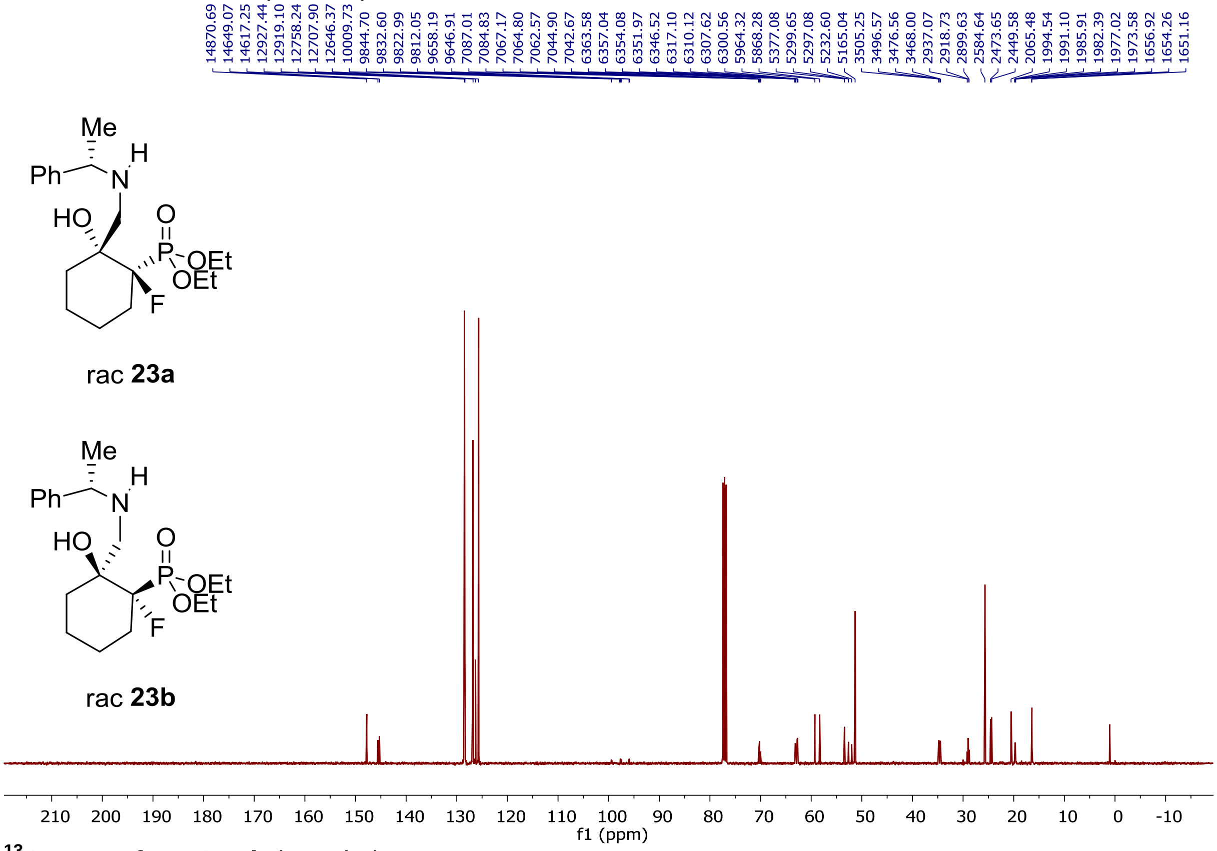

^{13}C NMR of rac 23a,b (1:1, d.r.)

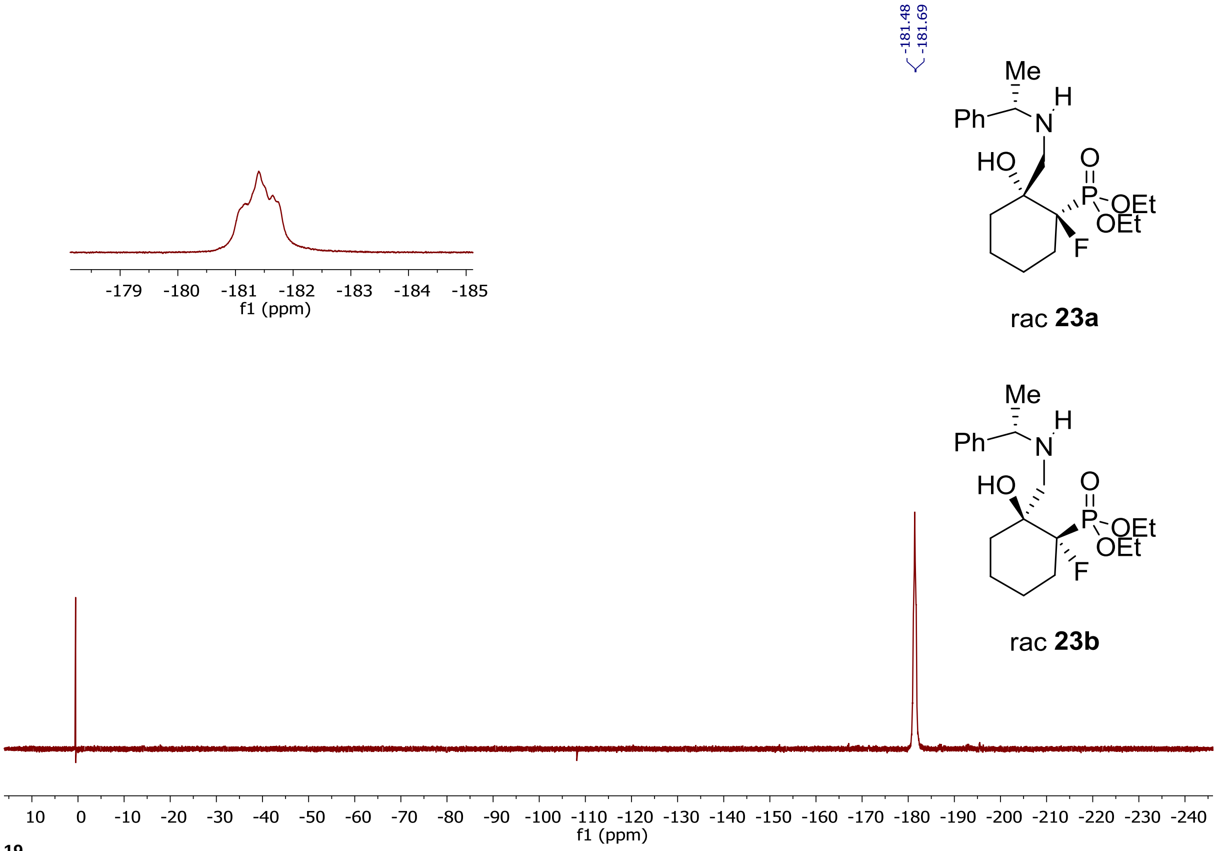

^{19}F NMR of rac **23a,b** (1:1, d.r.)

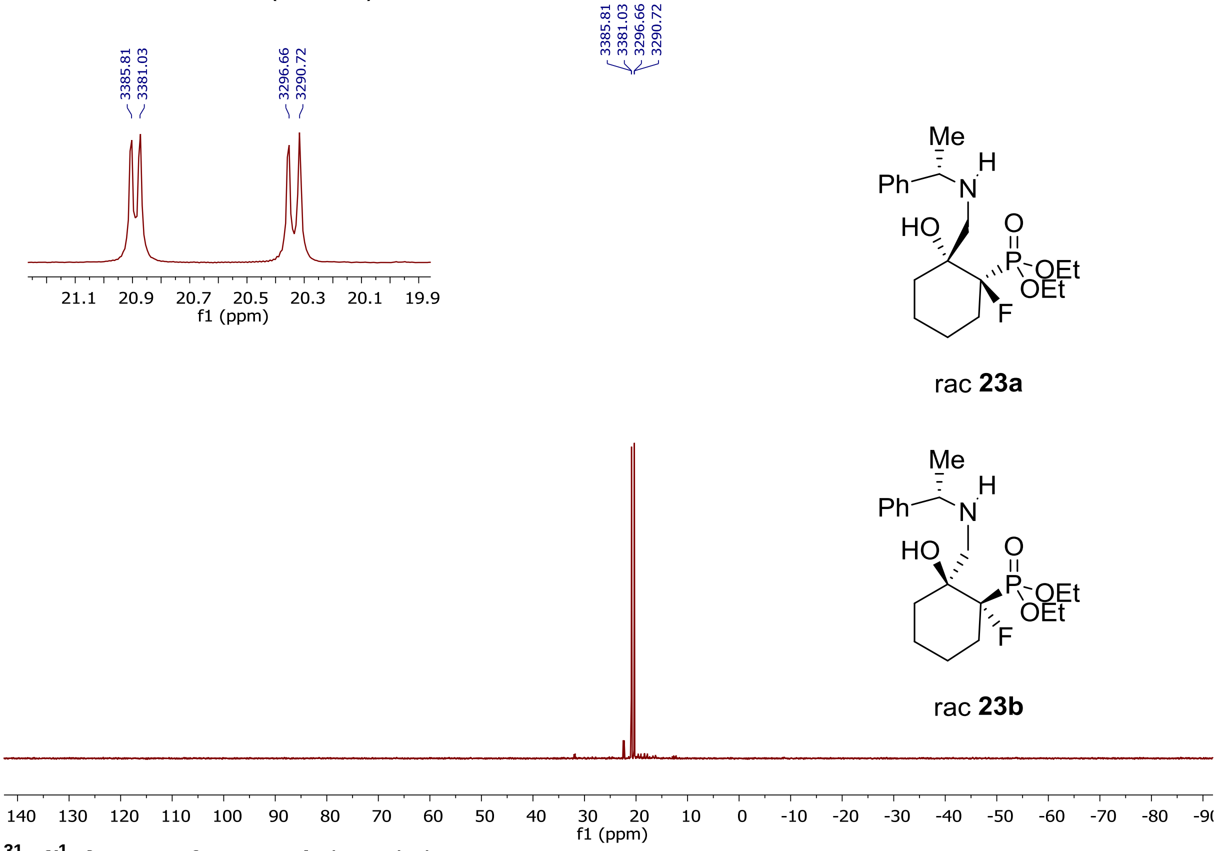

^{31}P{/^{1}H} NMR of rac **23a,b** (1:1, d.r.)

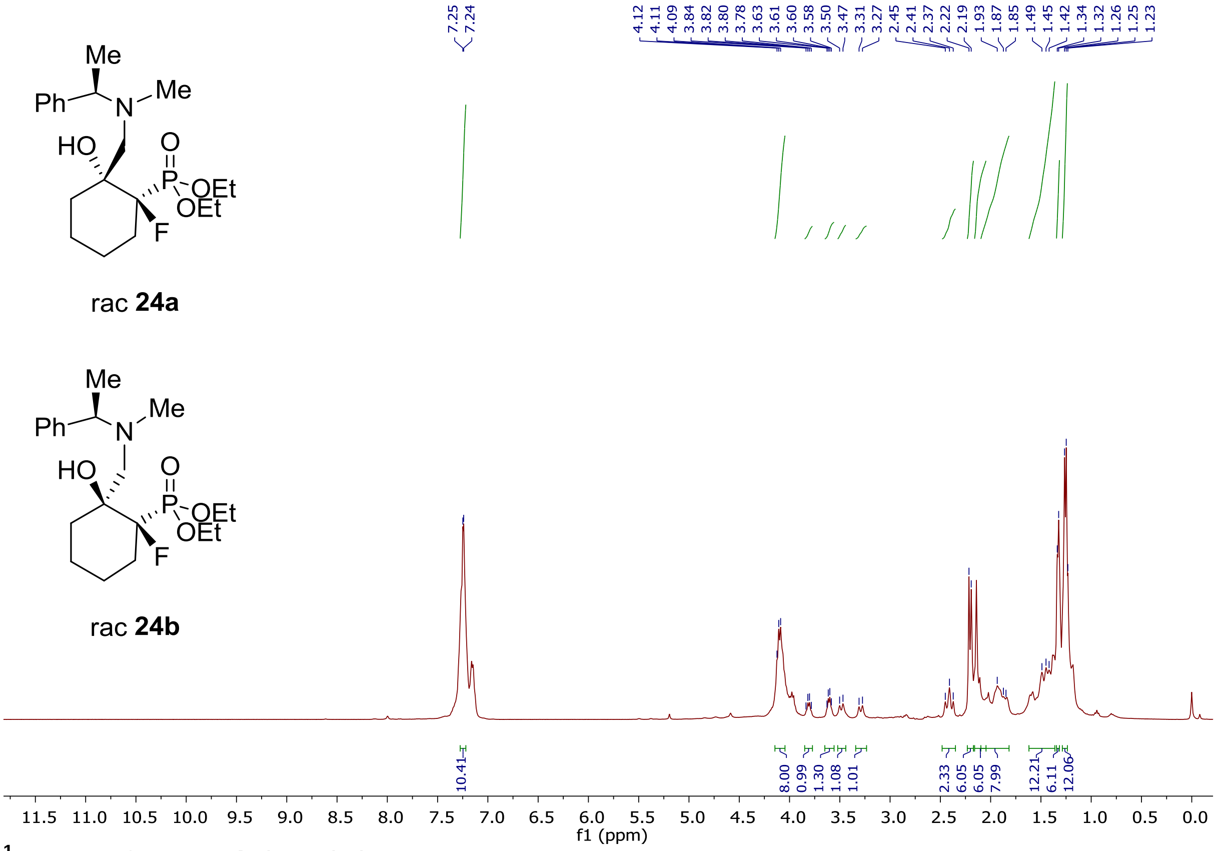

^{1}H NMR of rac **24a,b** (1:1, d.r.)

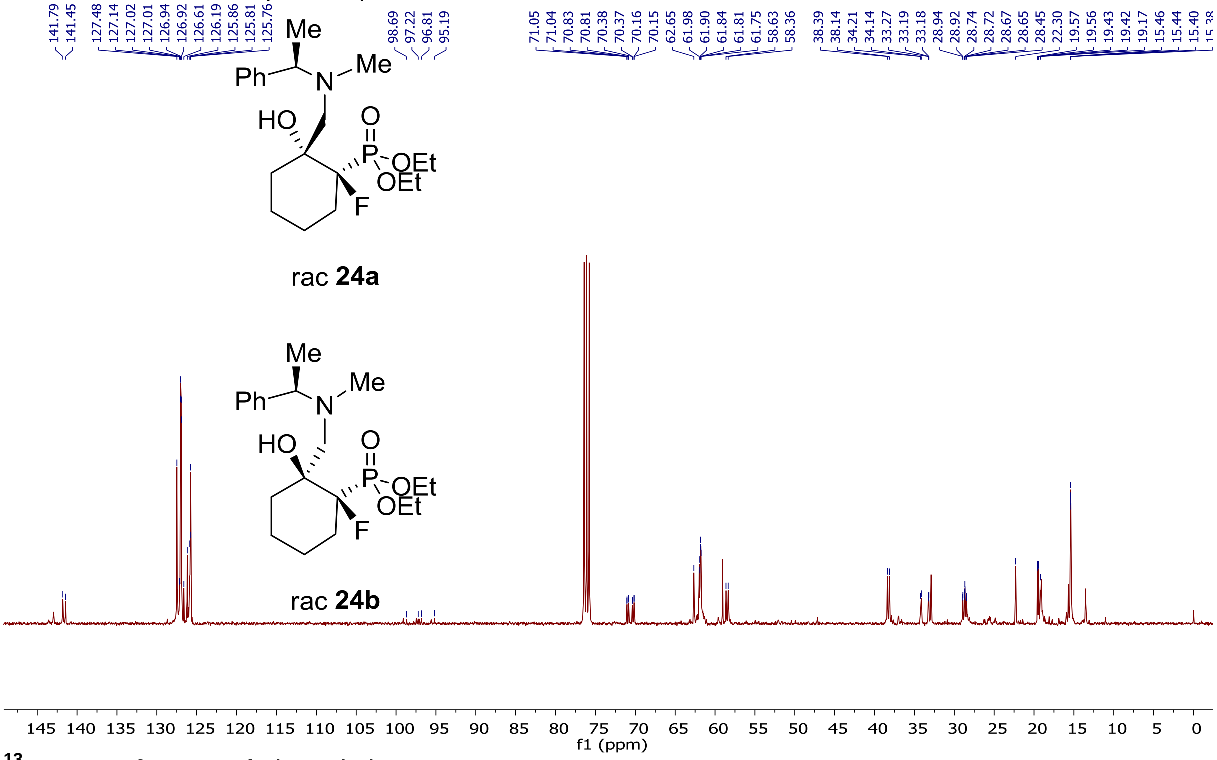

^{13}C NMR of rac **24a,b** (1:1, d.r.)

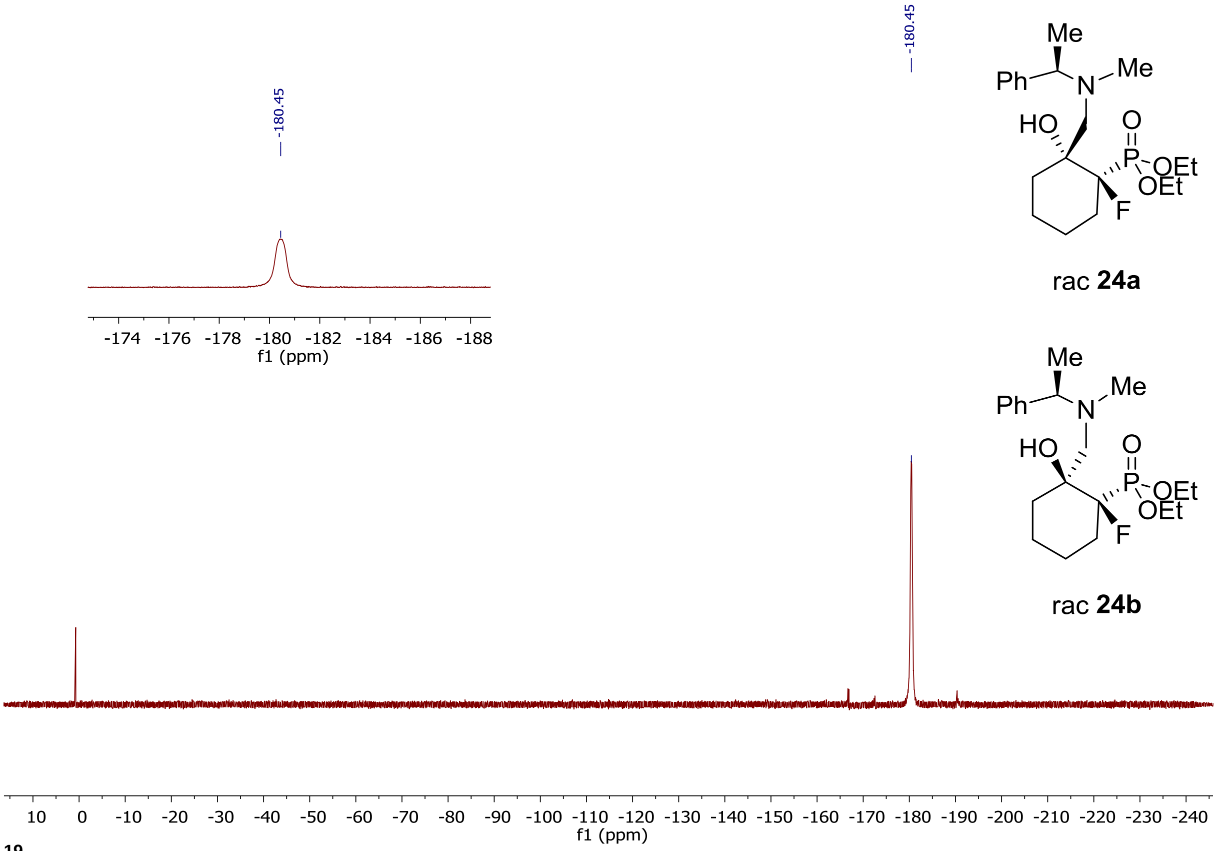

^{19}F NMR of rac **24a,b** (1:1, d.r.)

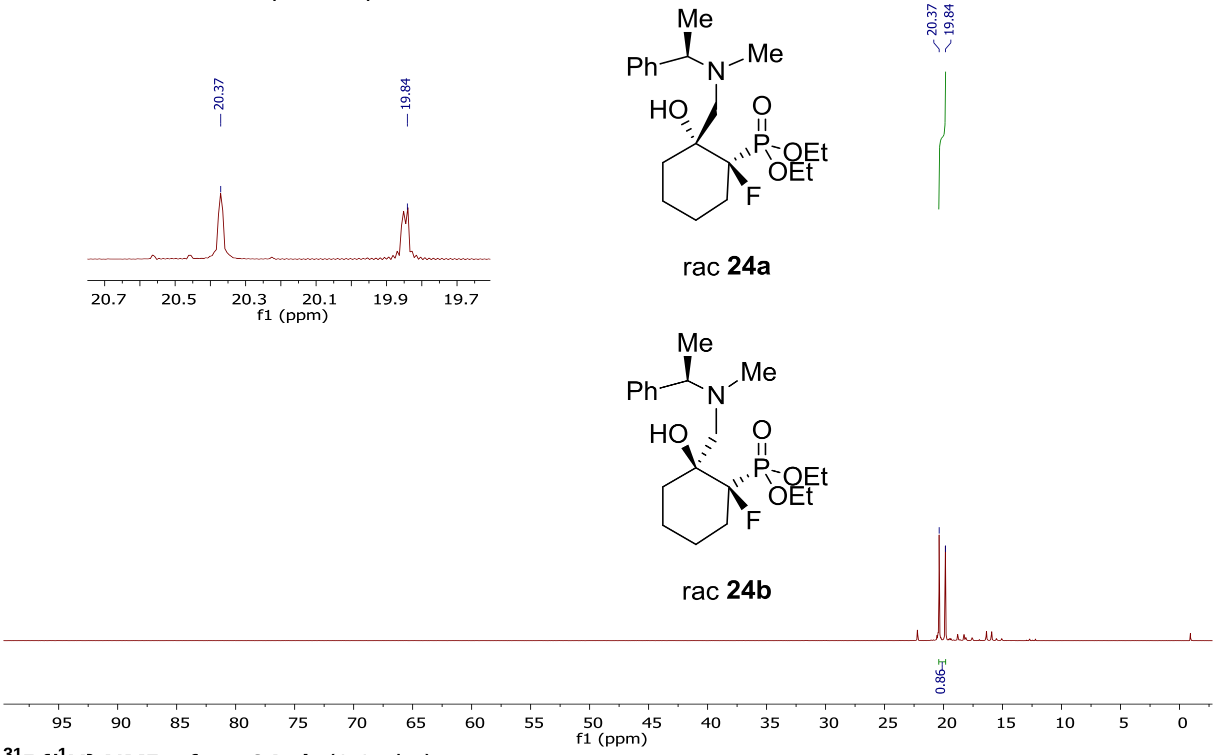

^{31}P{/^{1}H} NMR of rac **24a,b** (1:1, d.r.)

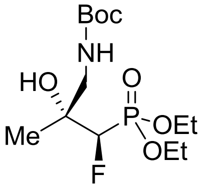

rac **26a**

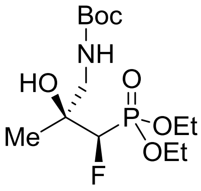

rac **26b**

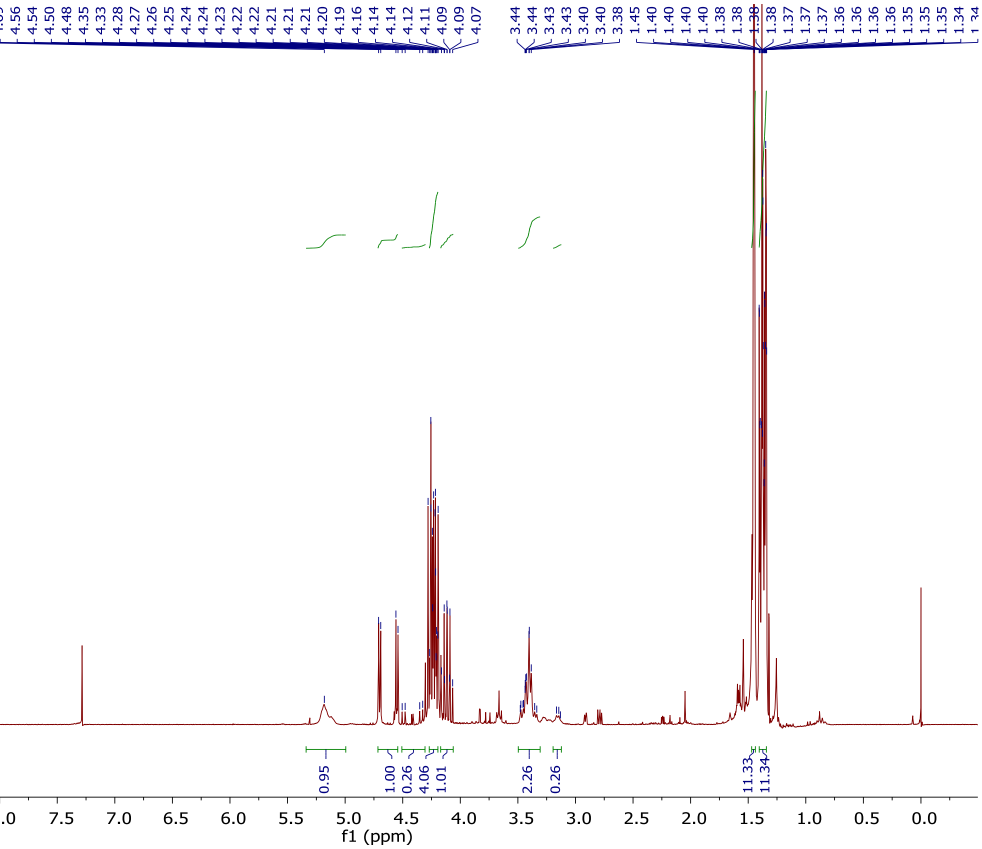

^{1}H NMR of rac **26a,b** (3.8:1, d.r.)

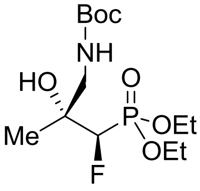

rac **26a**

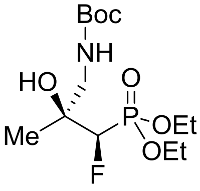

rac **26b**

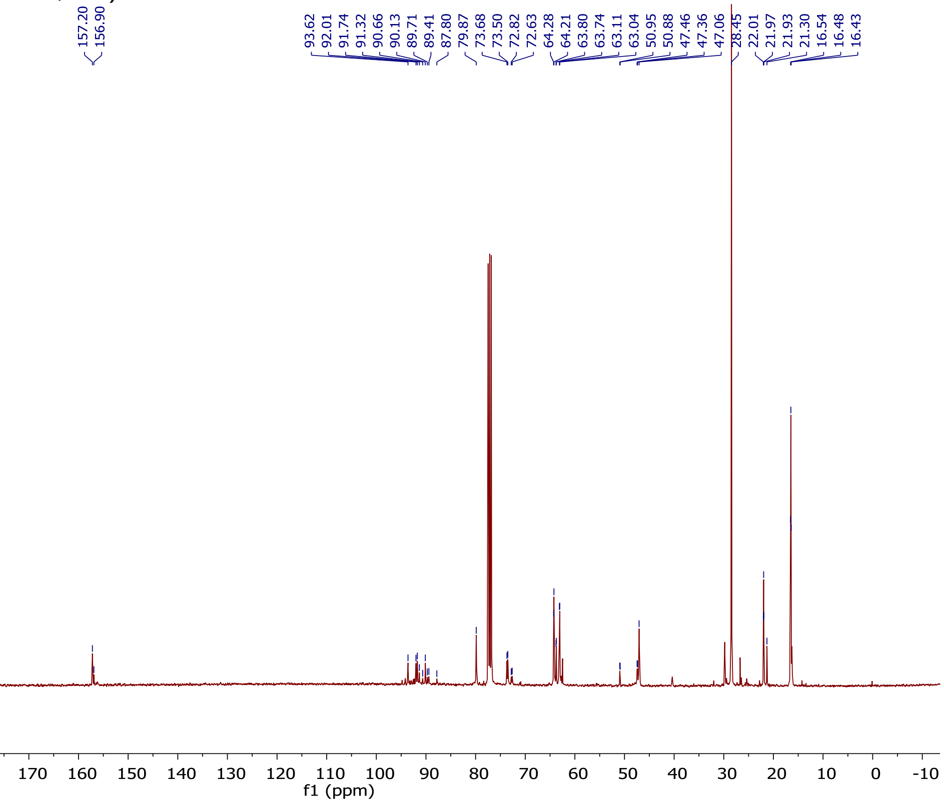

^{13}C NMR of rac **26a,b** (3.8:1, d.r.)

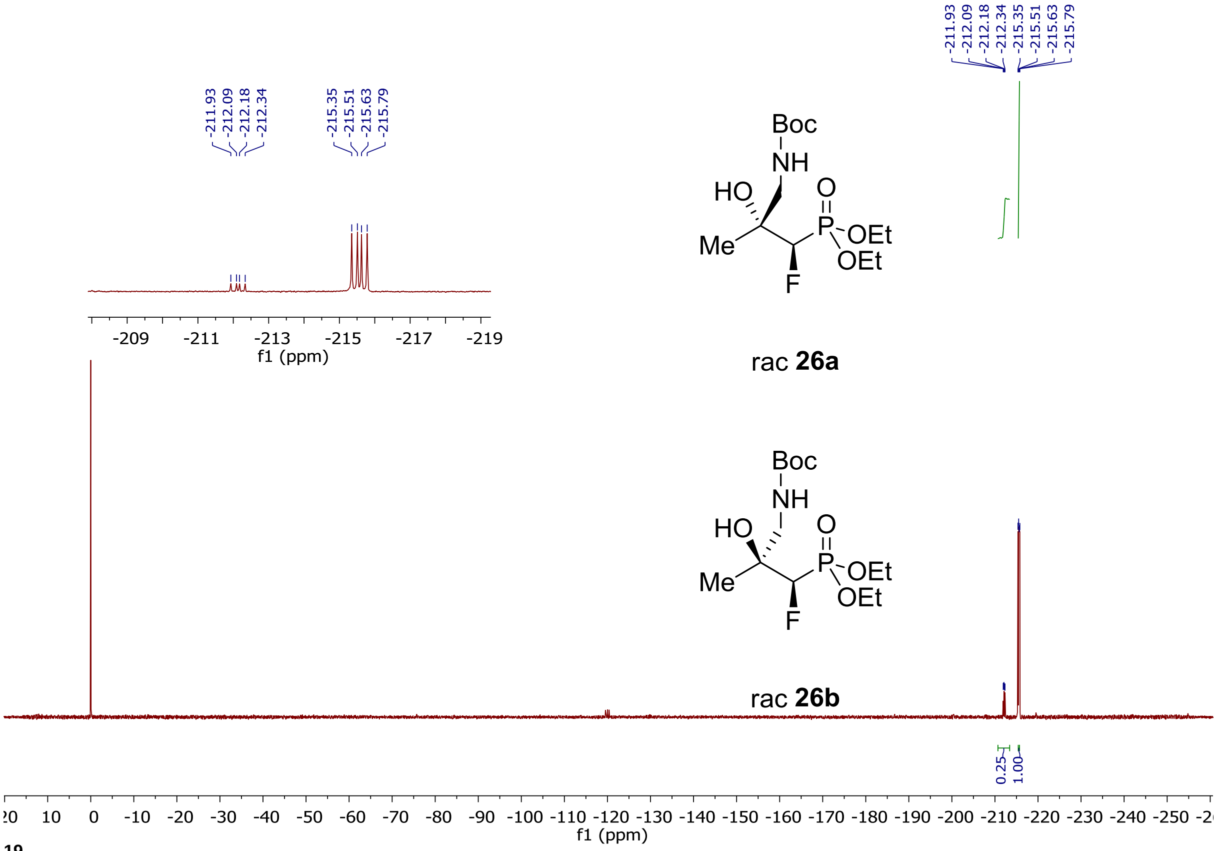

^{19}F NMR of rac **26a,b** (3.8:1, d.r.)

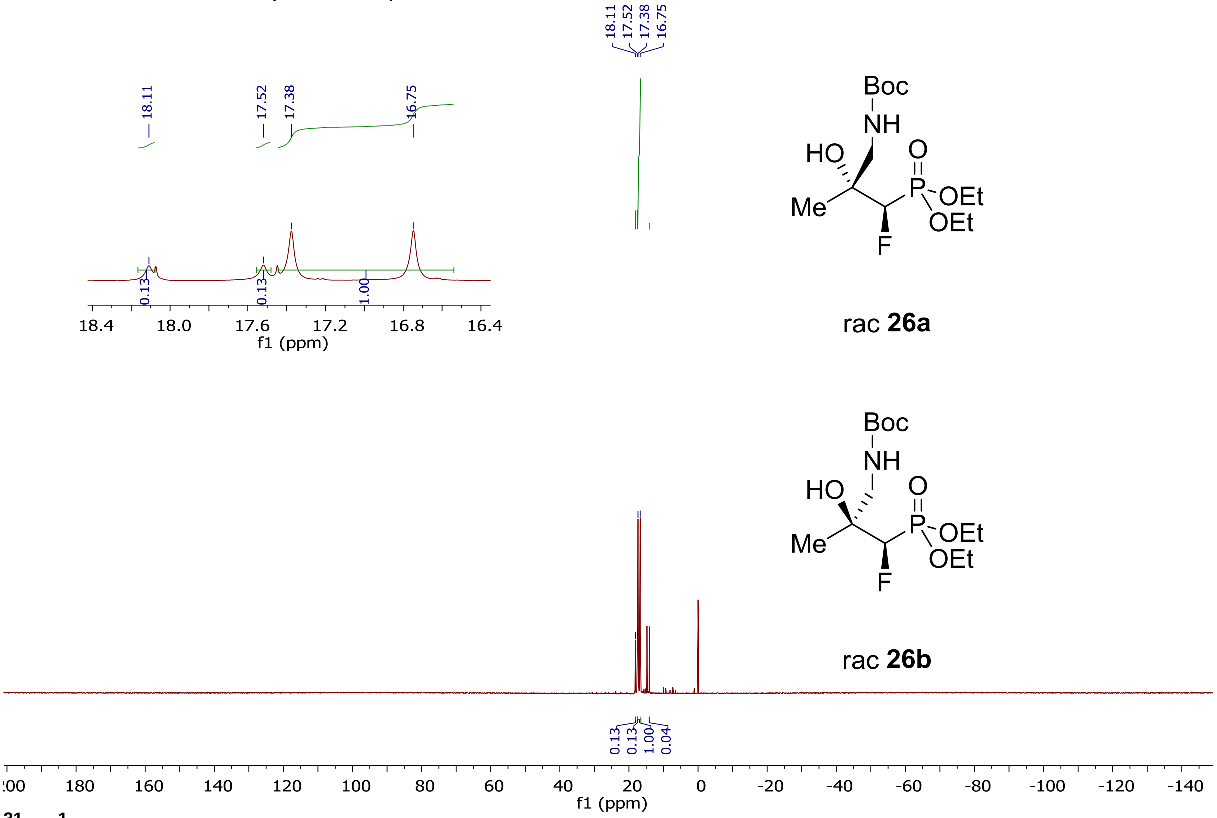

^{31}P{/^{1}H} NMR of rac **26a,b** (3.8:1, d.r.)

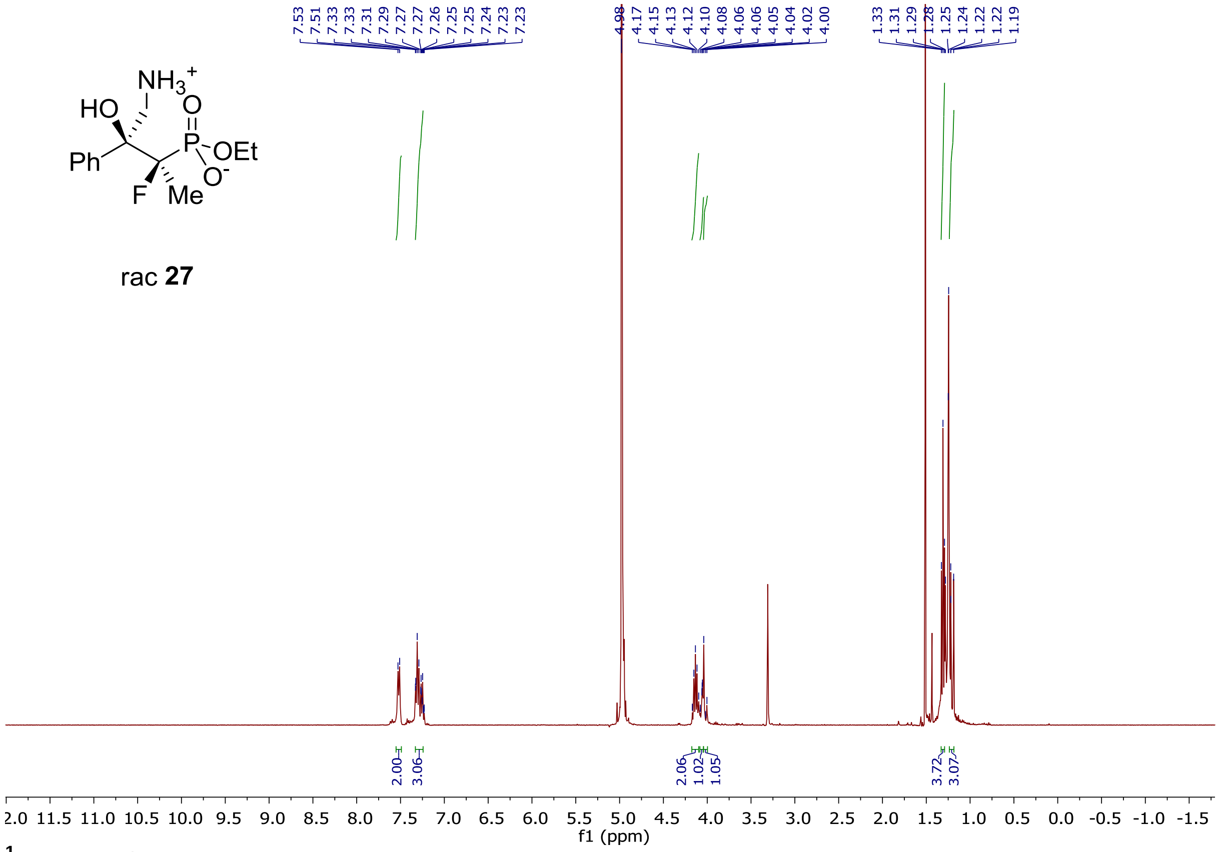

rac **27**

^{1}H NMR of rac **27**

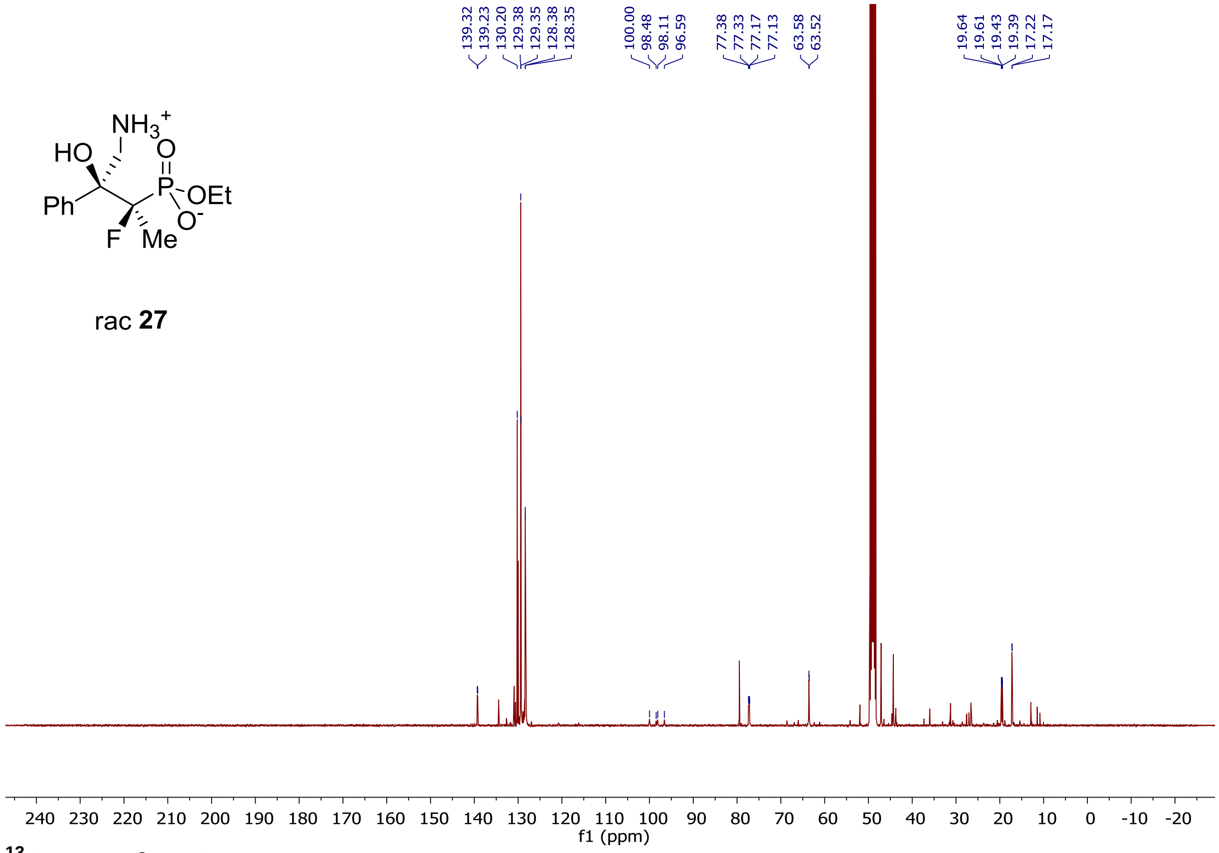

rac **27**

^{13}C NMR of rac **27**

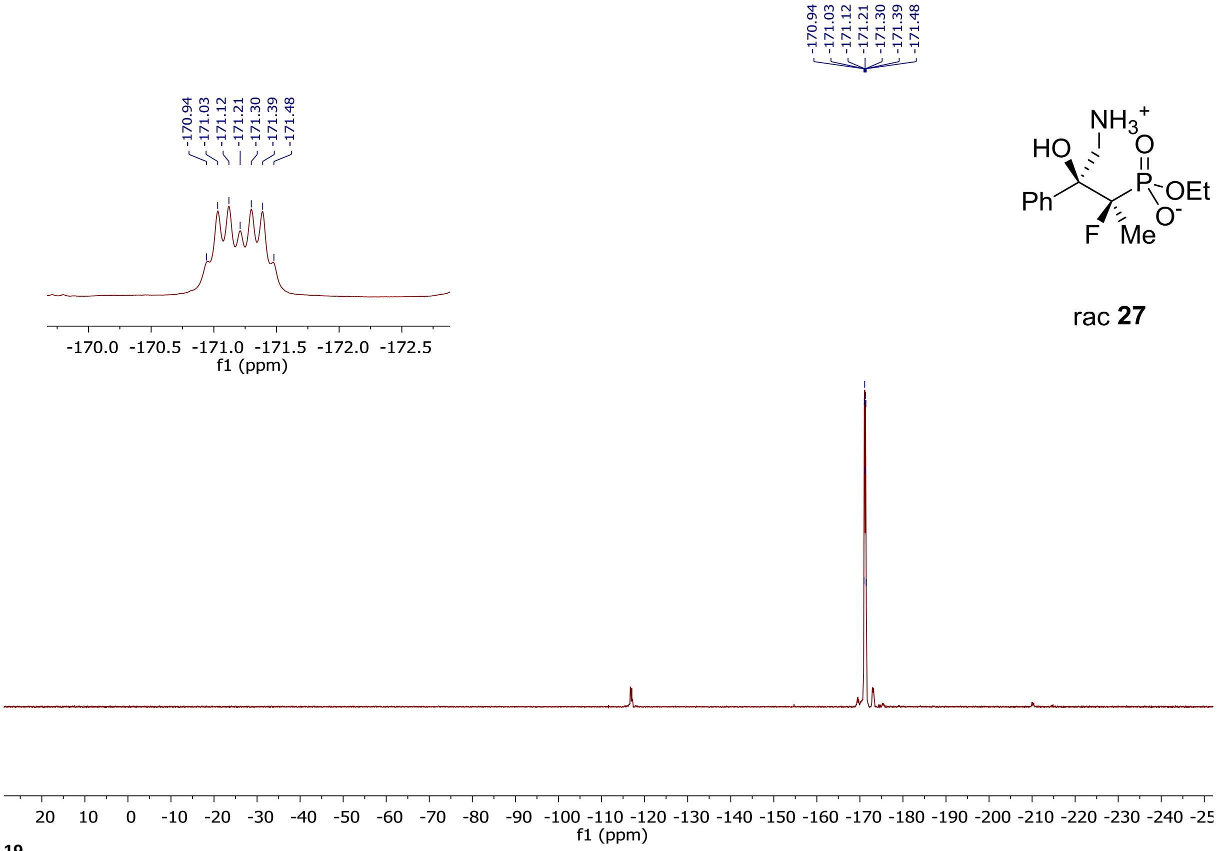

rac **27**

^{19}F NMR of rac **27**

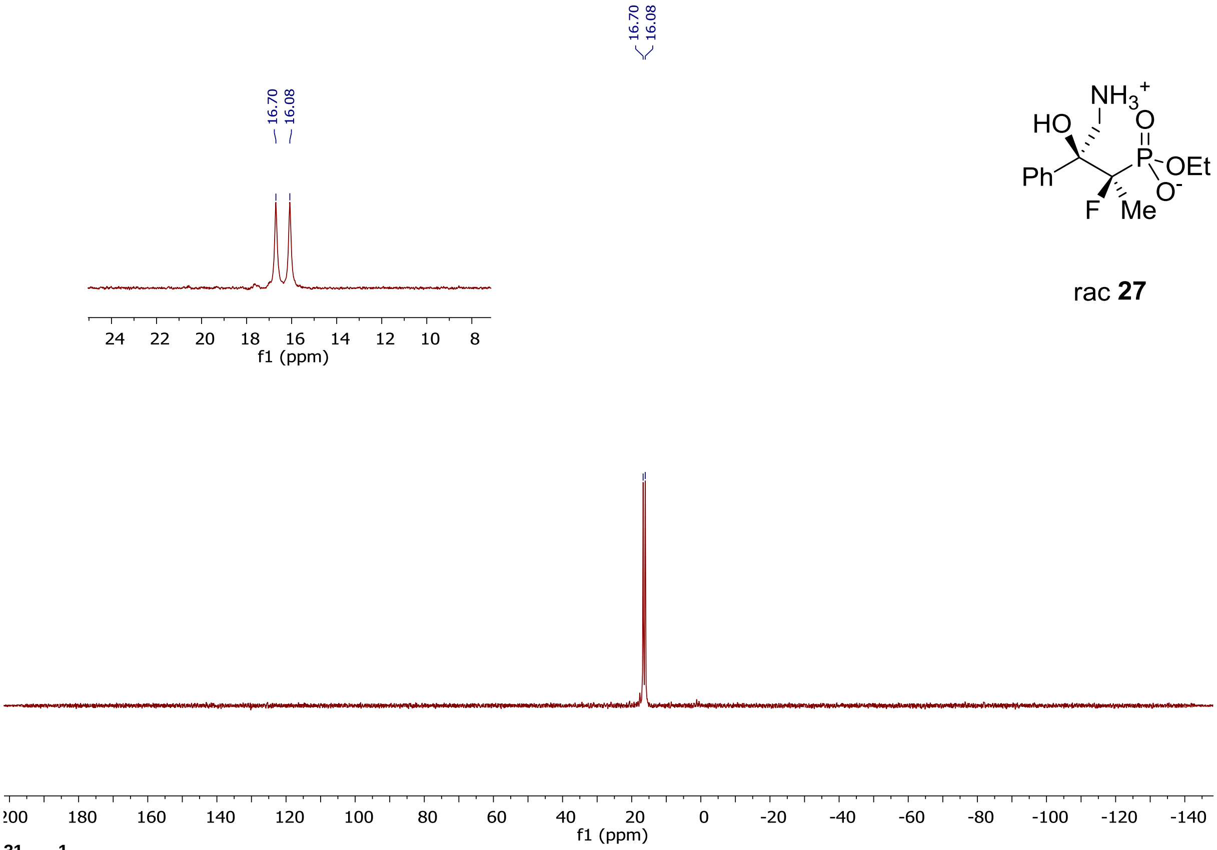

rac **27**

^{31}P{/^{1}H} NMR of rac **27**

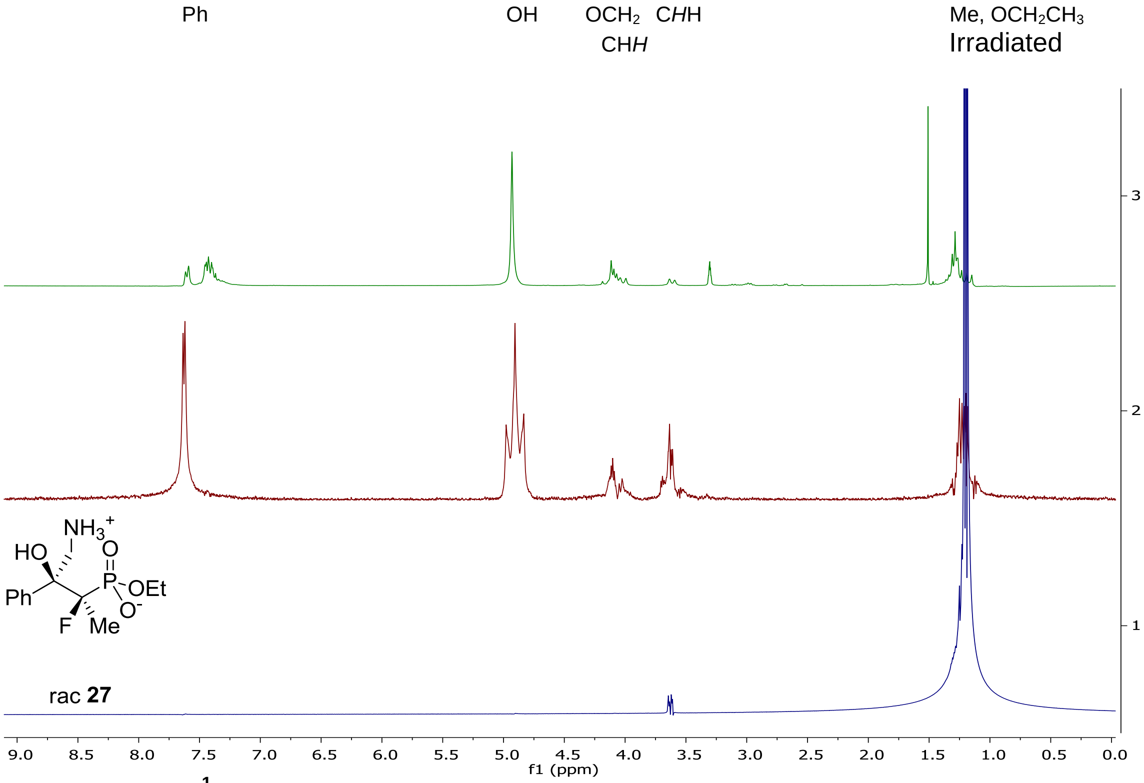

Stacked plots of ^{1}H NMR (top) and **1D H-F HOESY** (center) and **1D NOESY** of rac **27** (bottom).

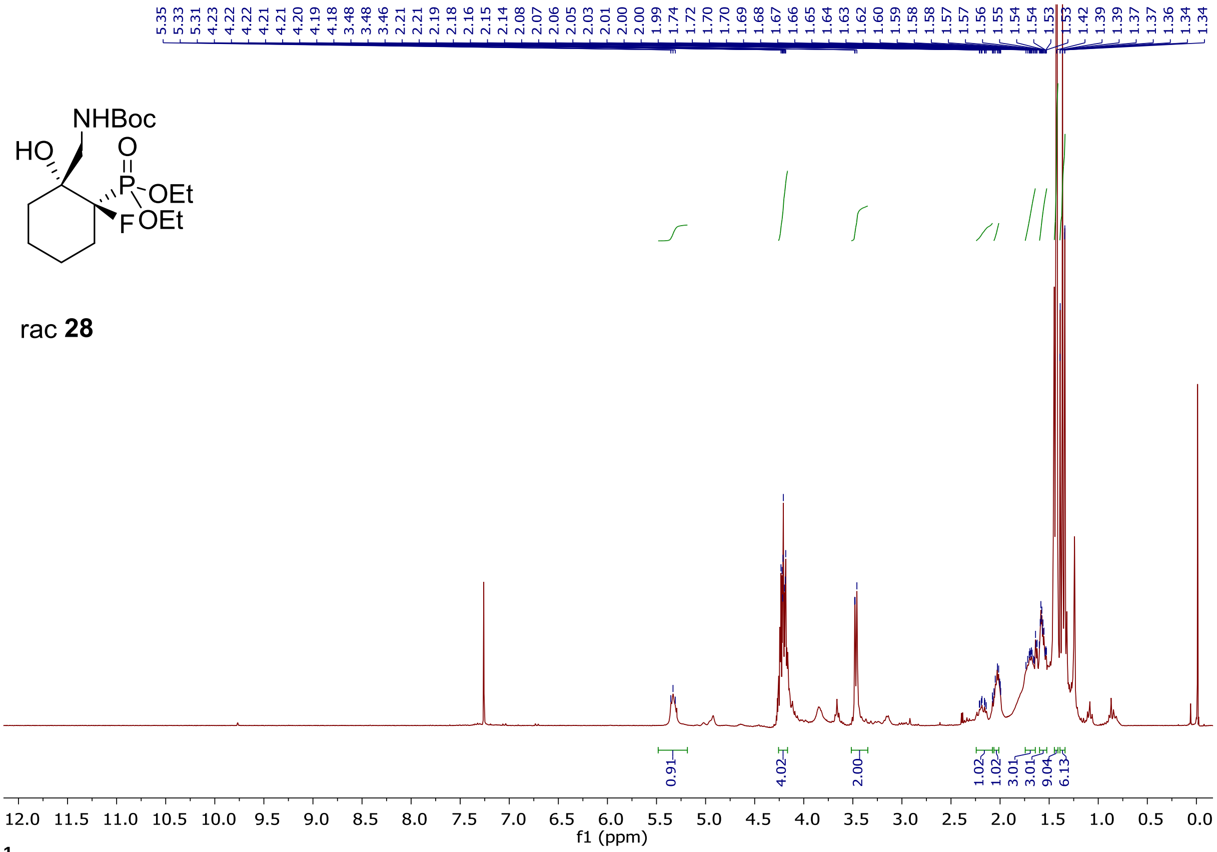

^{1}**H NMR of** rac **28**

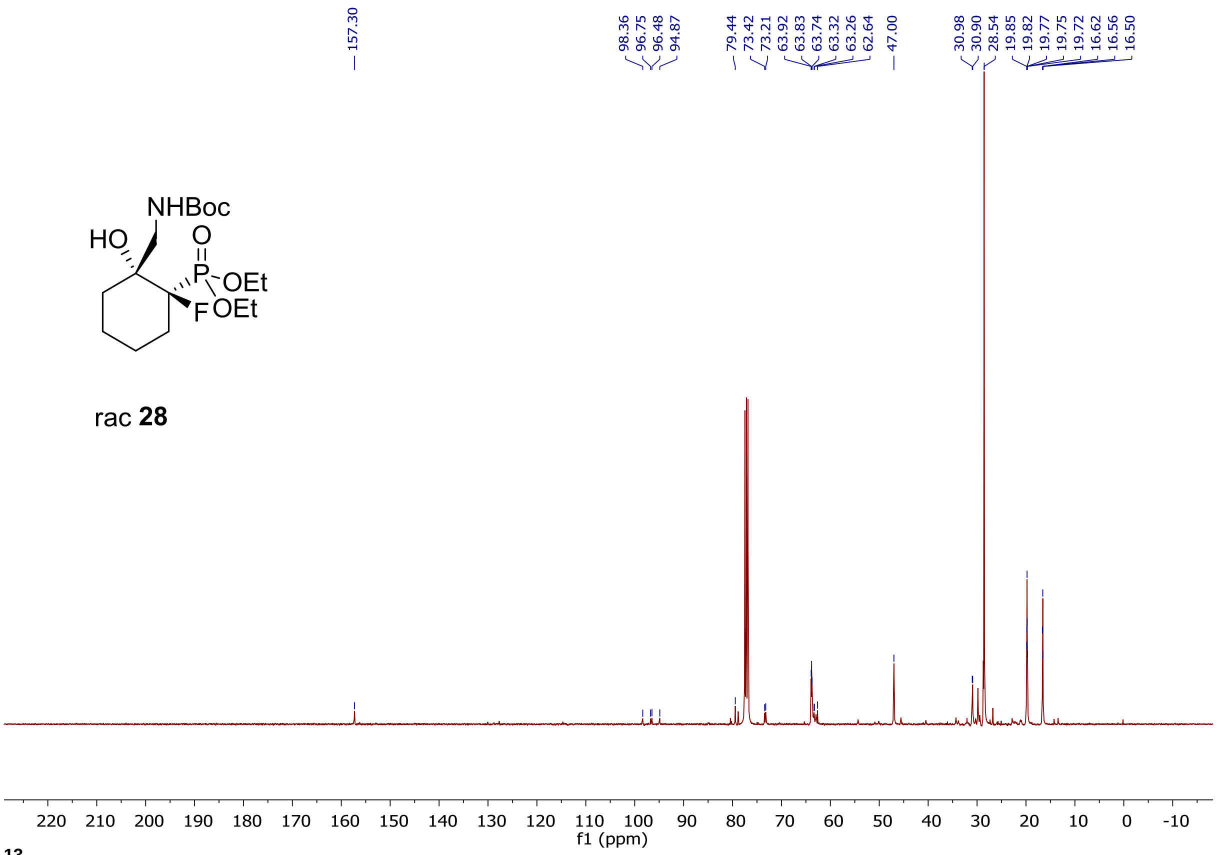

^{13}C NMR of rac **28**

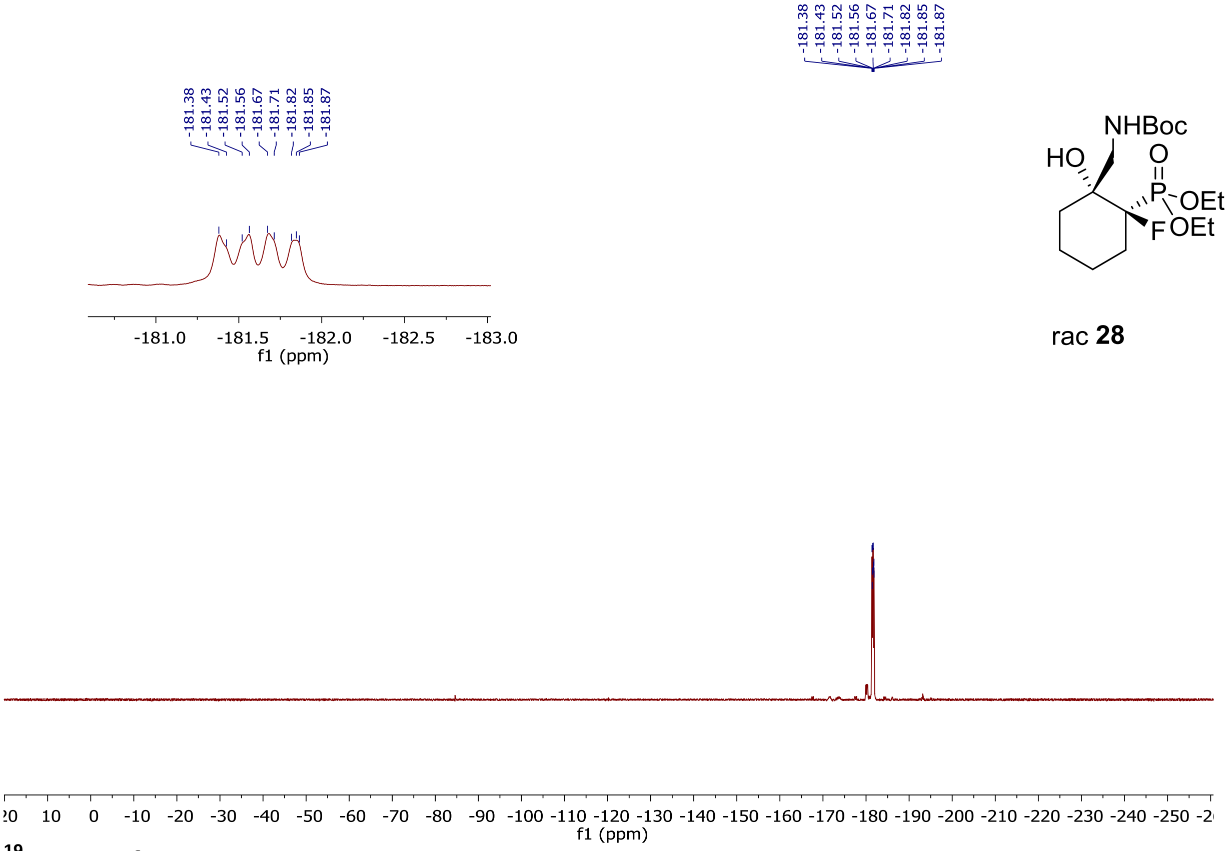

^{19}F NMR of rac **28**

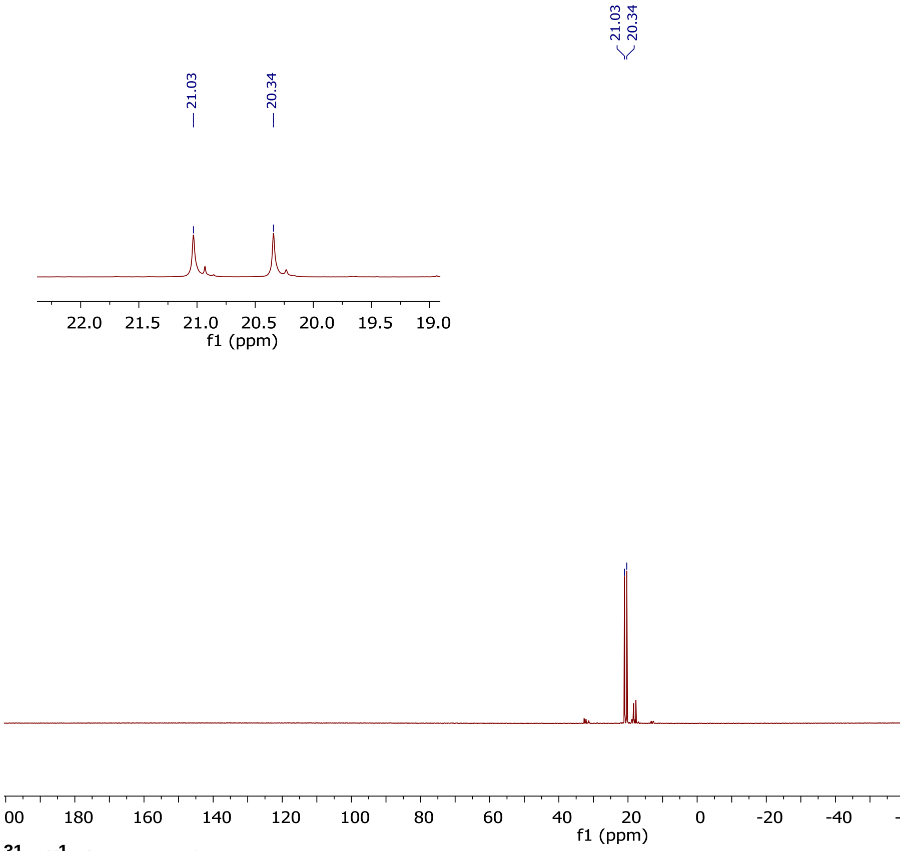

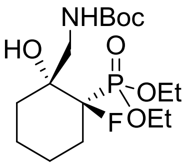

rac **28**

^{31}P{/^{1}H} NMR of rac **28**

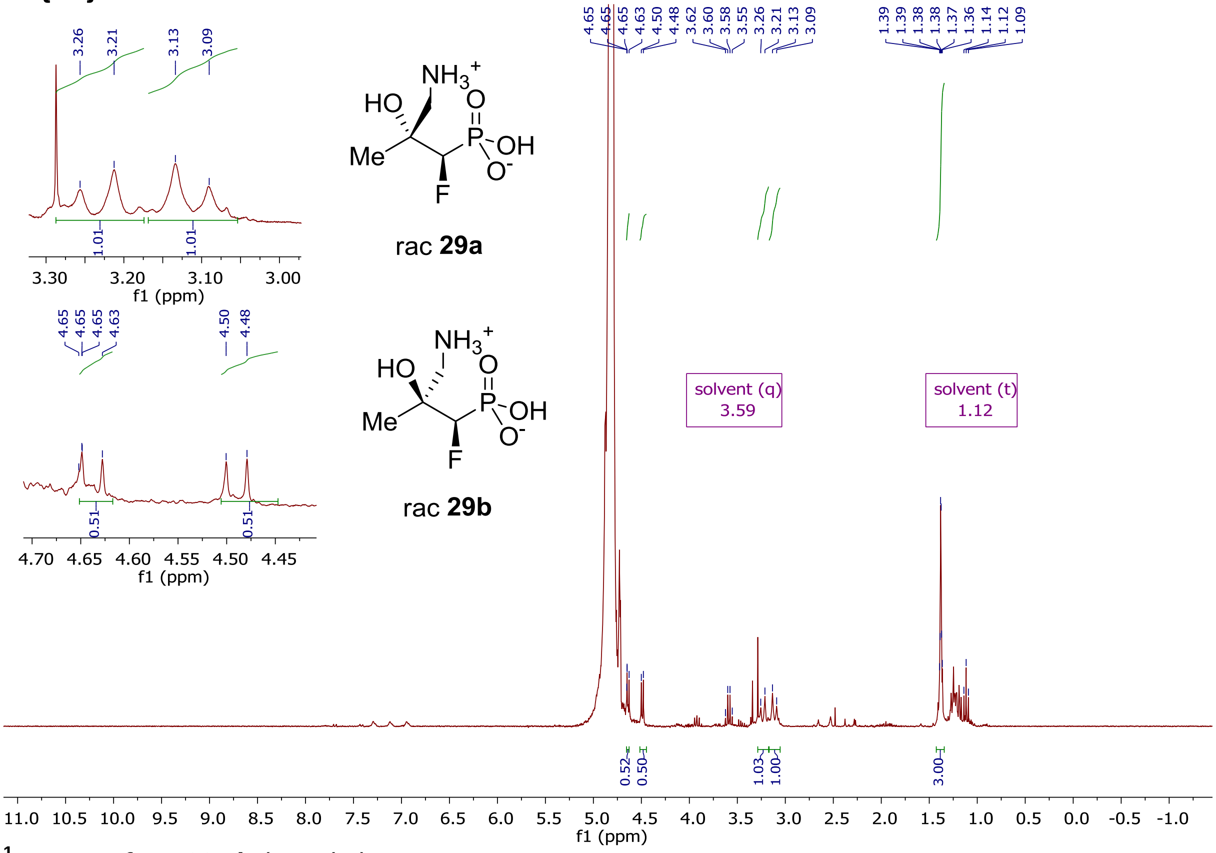

^{1}H NMR of rac **29a,b** (5:1, d.r.)

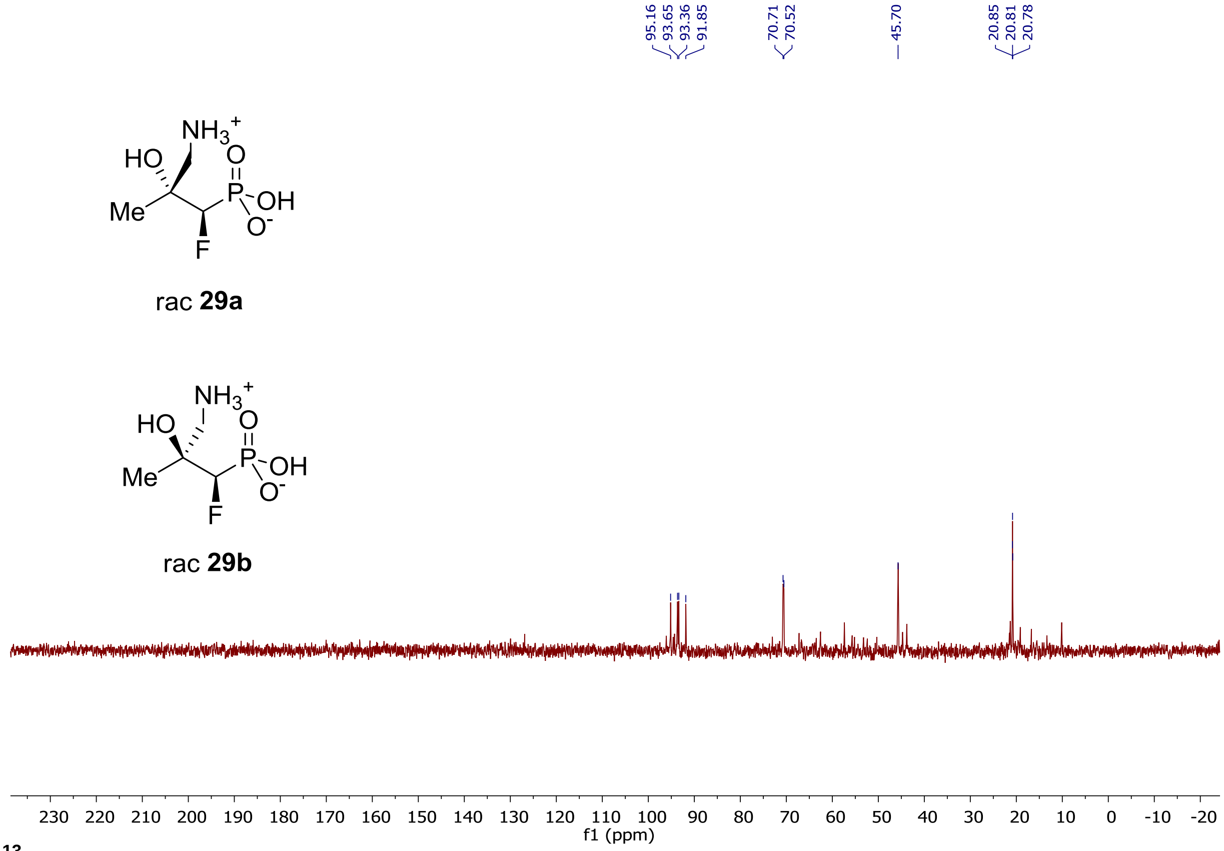

^{13}C NMR (D_2O) of rac **29a,b** (5:1, d.r.)

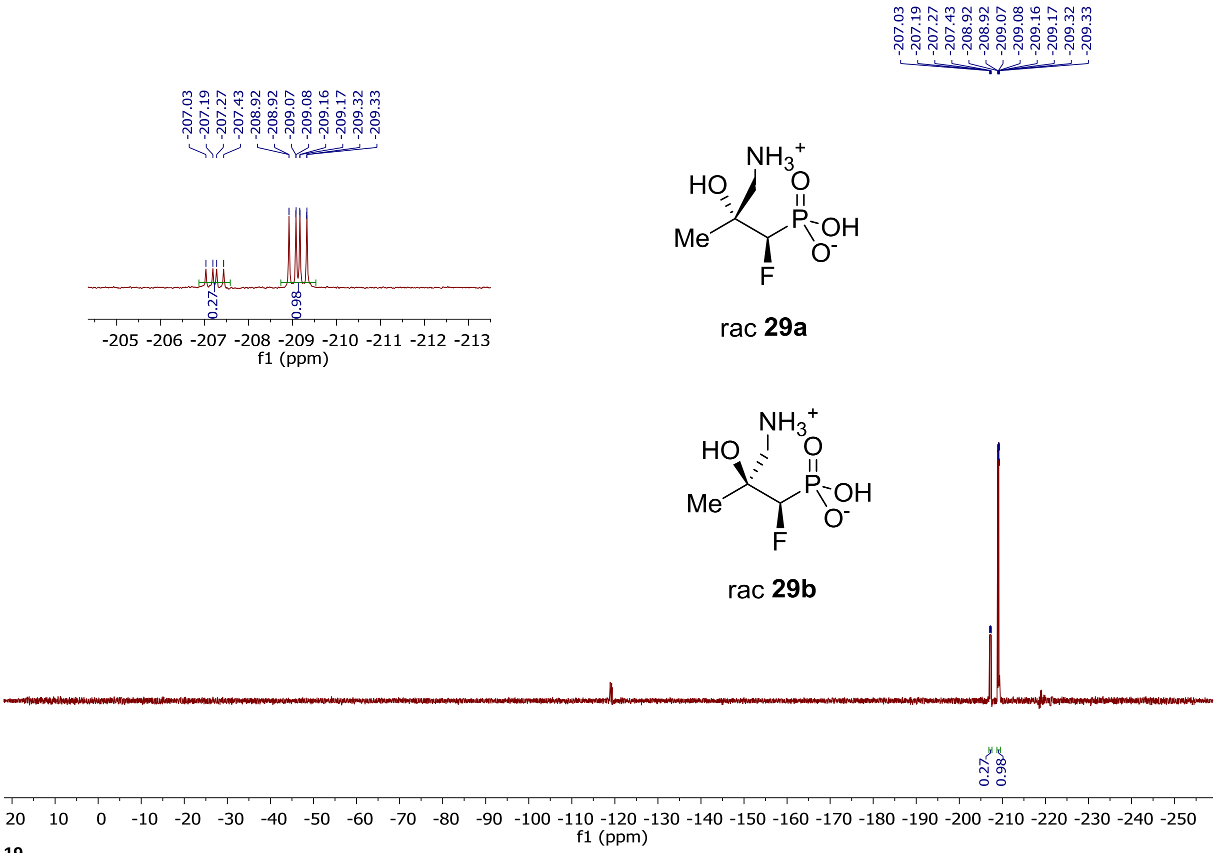

^{19}F NMR of rac **29a,b** (5:1, d.r.)

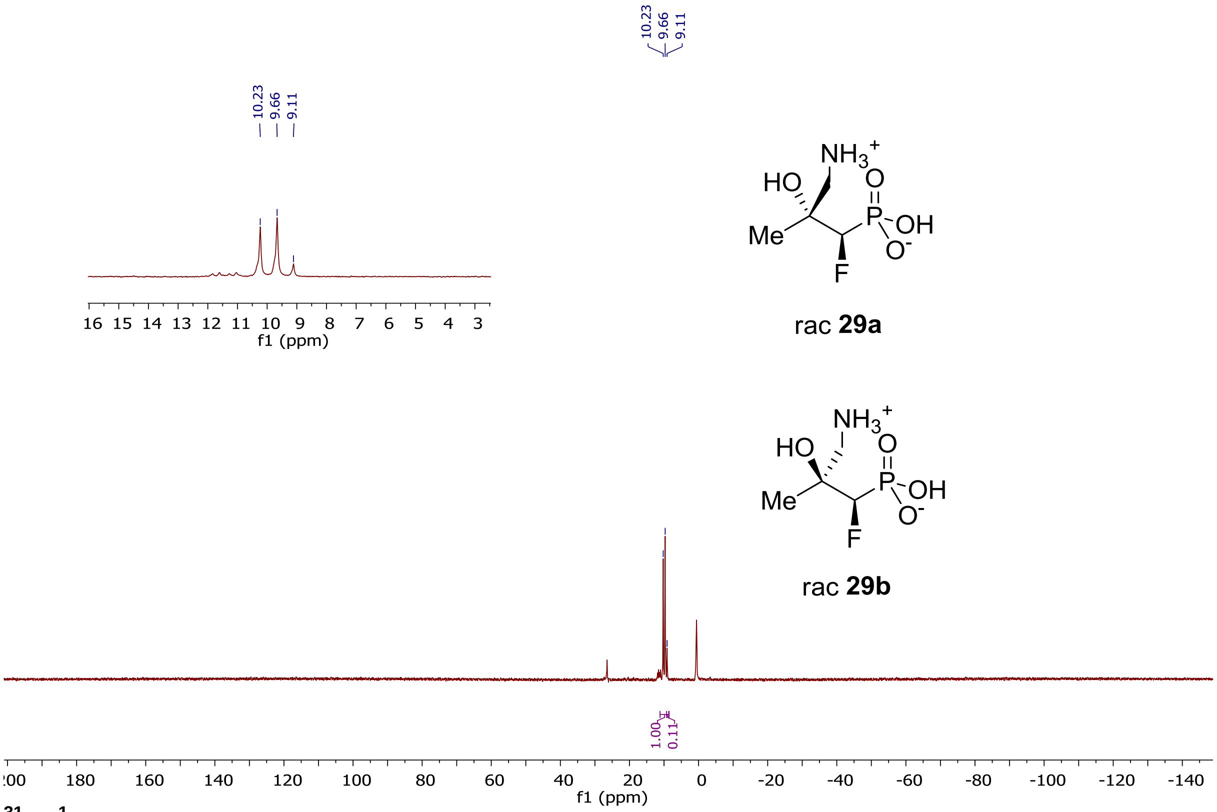

^{31}P{/^{1}H} NMR of rac **29a,b** (5:1, d.r.)

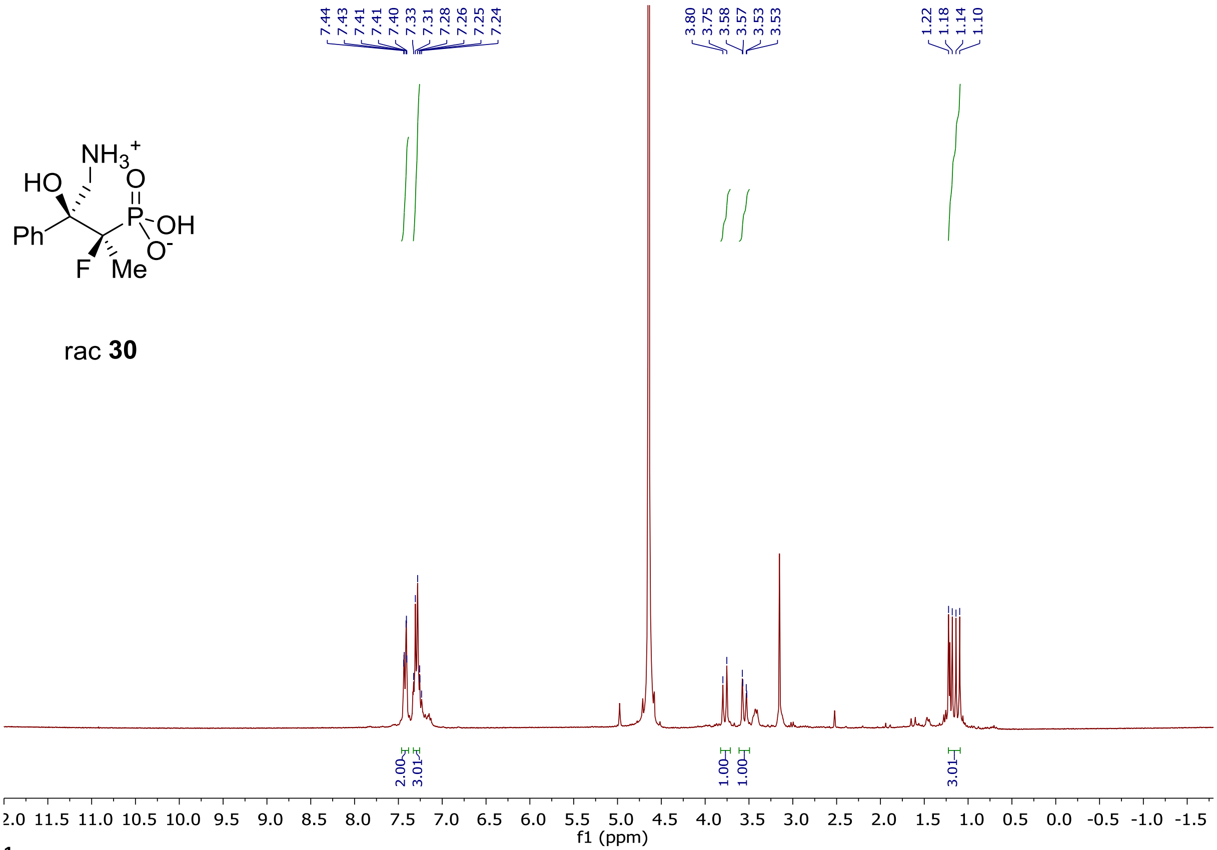

^{1}H NMR of rac **30**

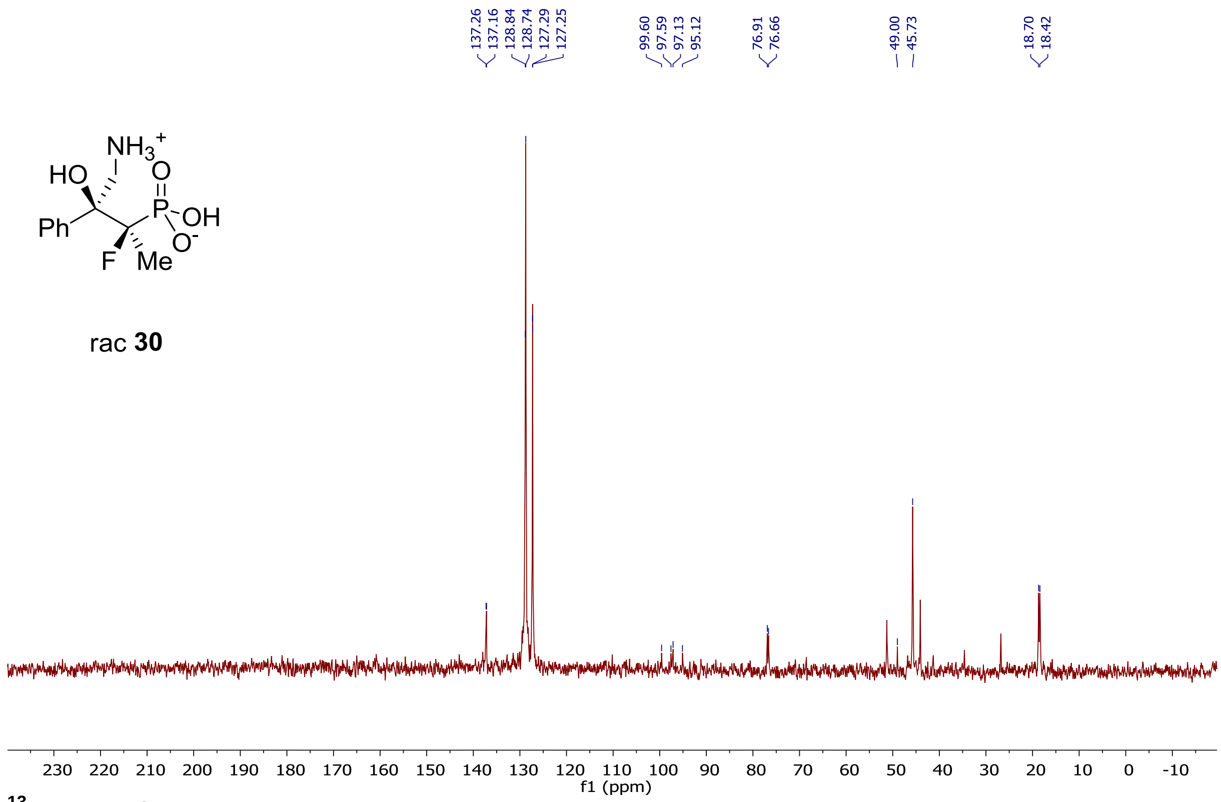

^{13}C NMR of rac 30

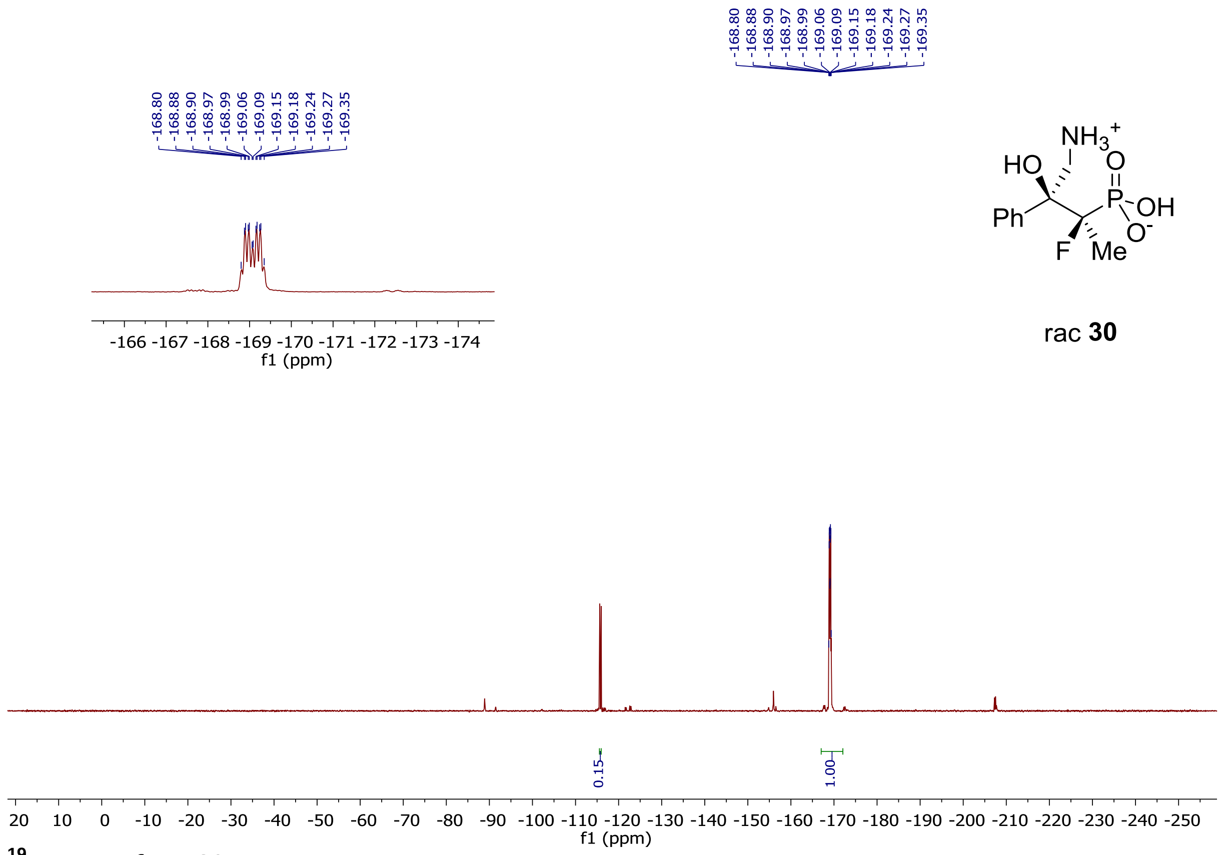

^{19}F NMR of rac 30

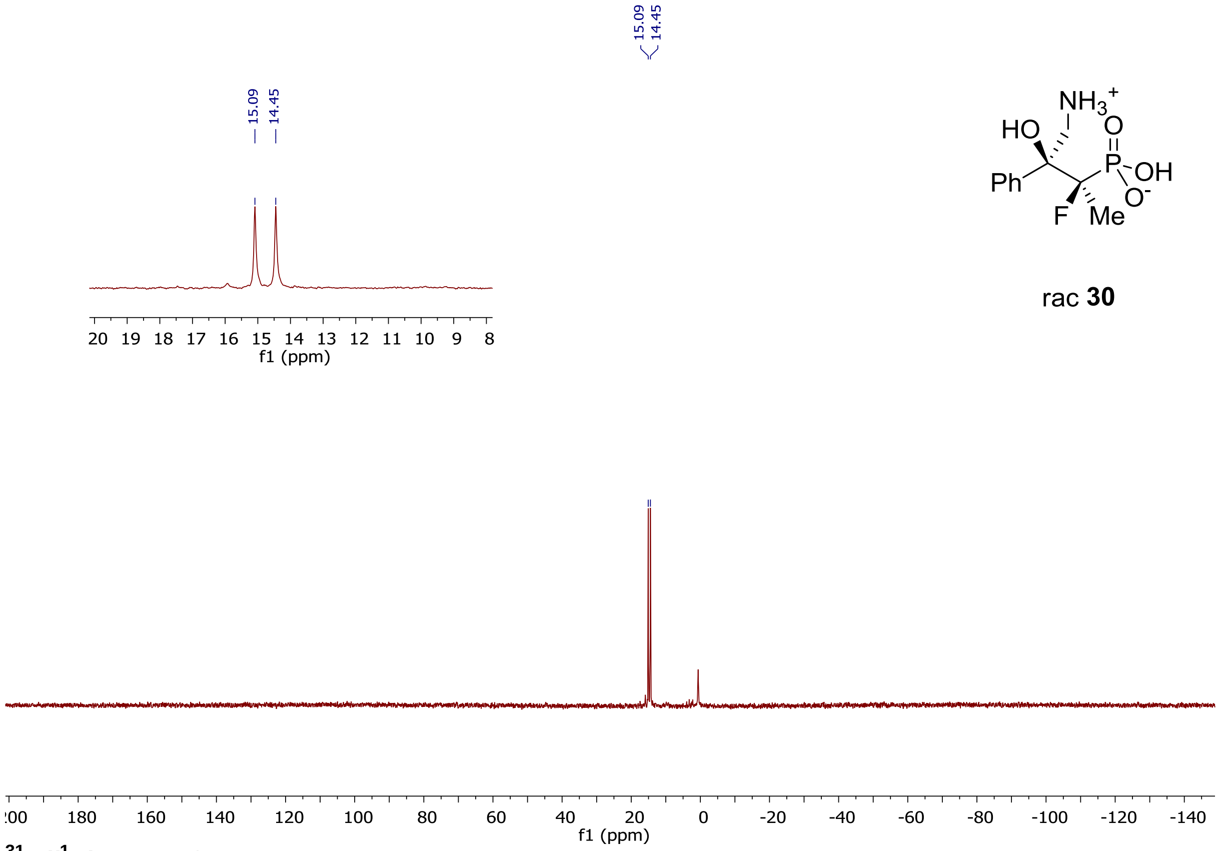

rac **30**

^{31}P{/^{1}H} NMR of rac **30**

AAD00003N0-H1

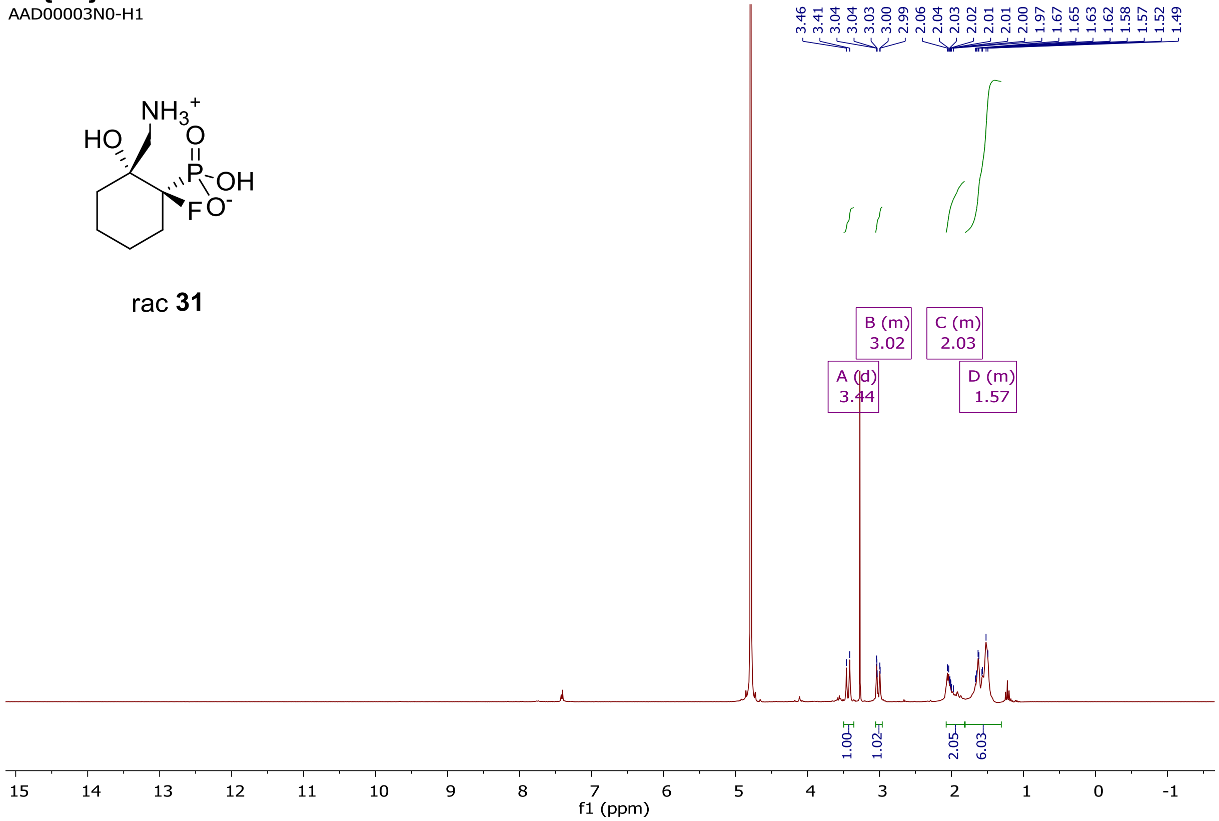

rac **31**

^{1}H NMR of rac **31** (D_2O)

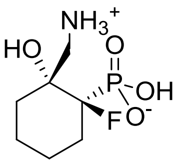

rac **31**

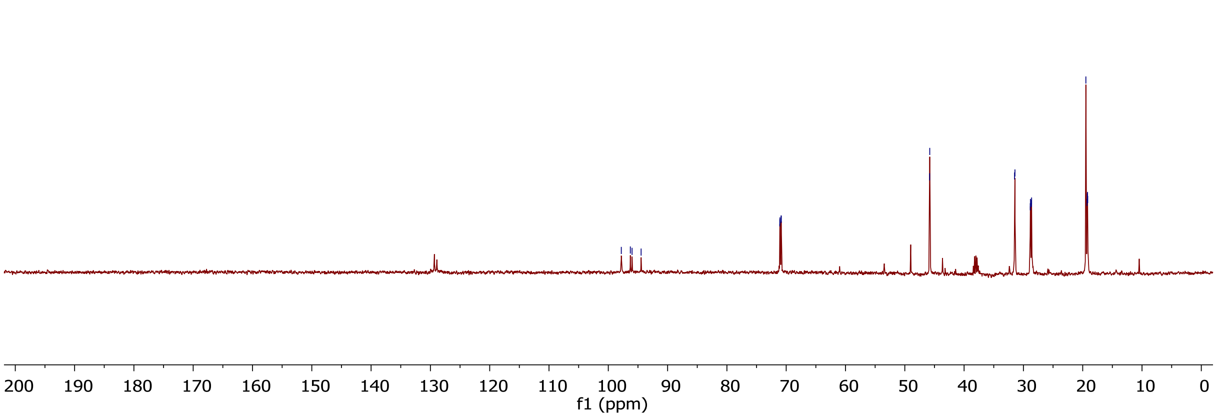

^{13}C NMR of rac **31** (D_2O)

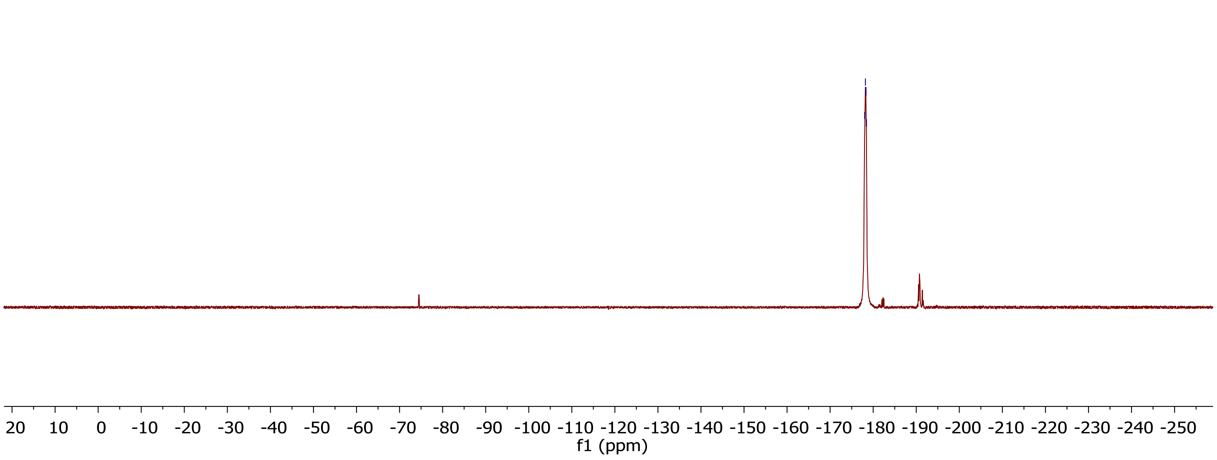

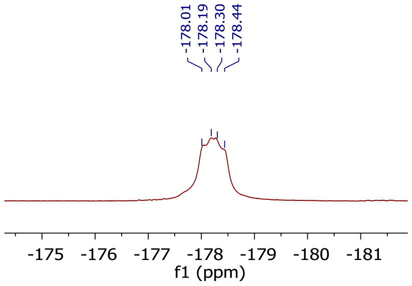

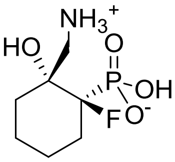

rac **31**

^{19}F NMR of rac **31** (D_2O)

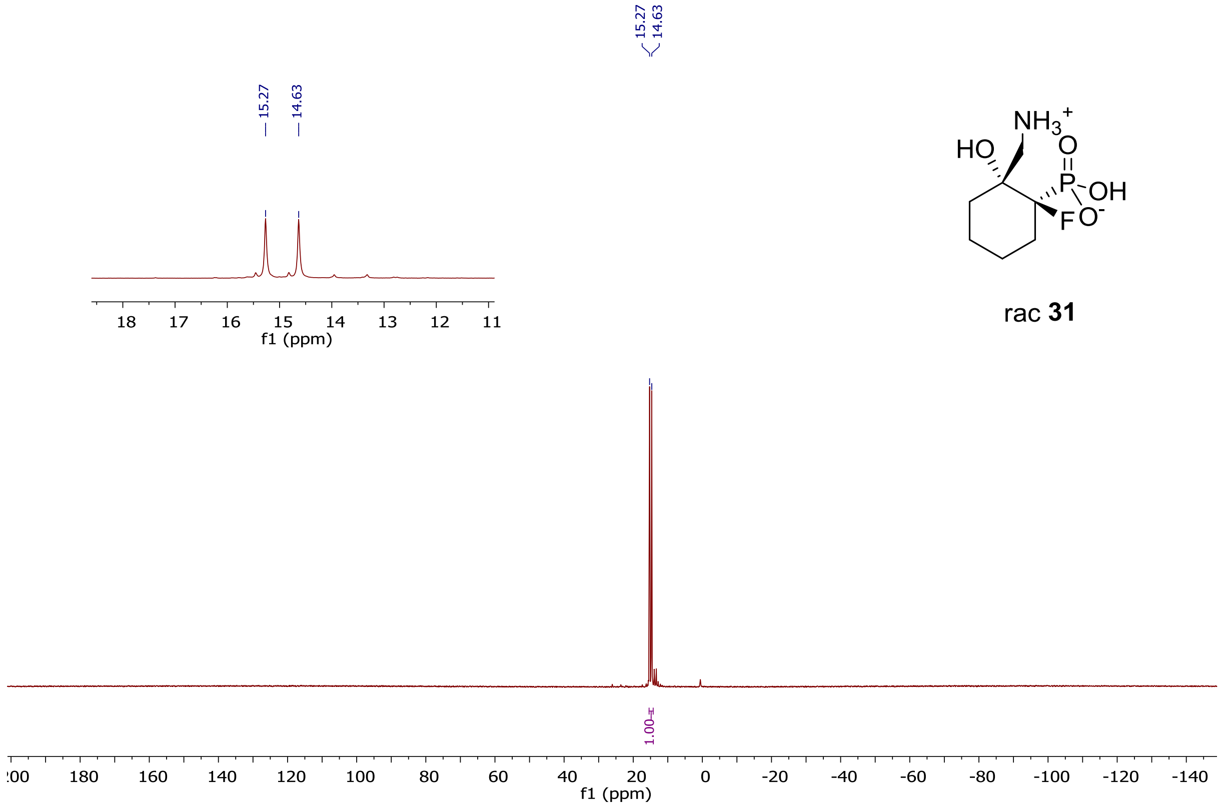

^{31}P{/^{1}H} NMR of rac **31** (D_2O)

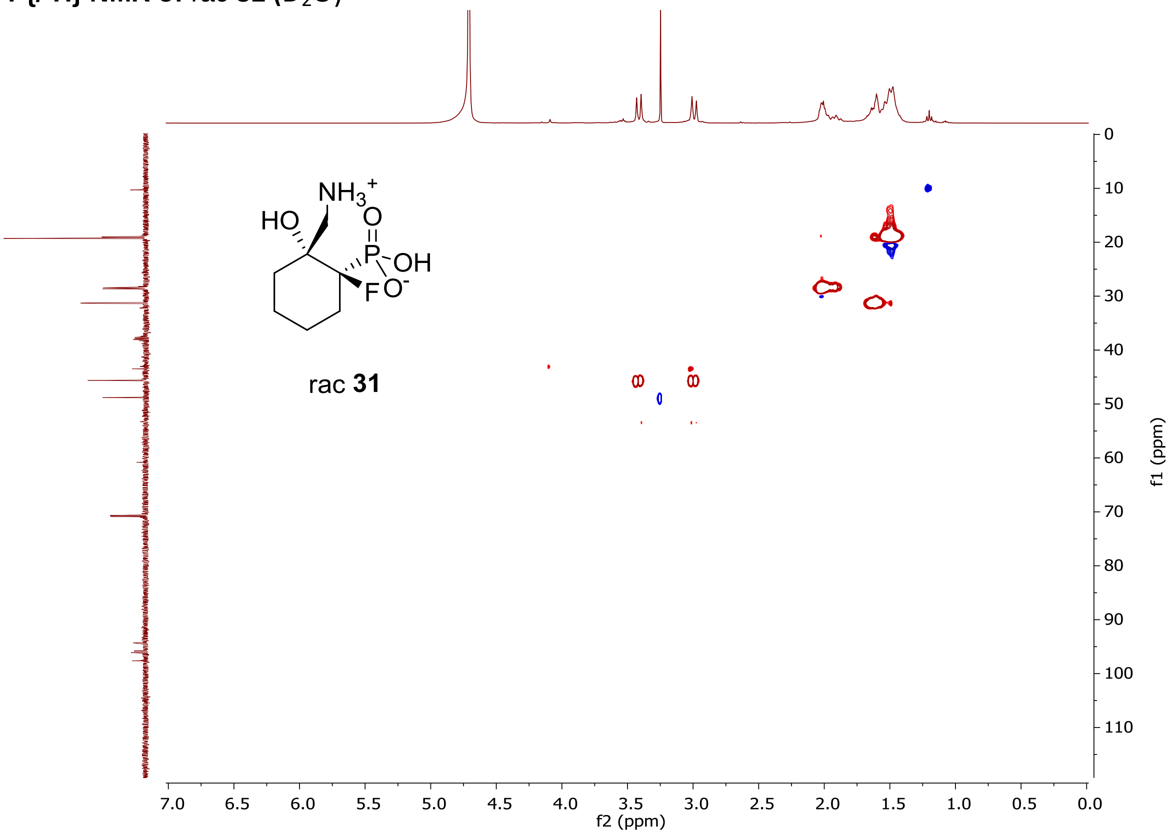

HSQC of rac **31** (D_2O)

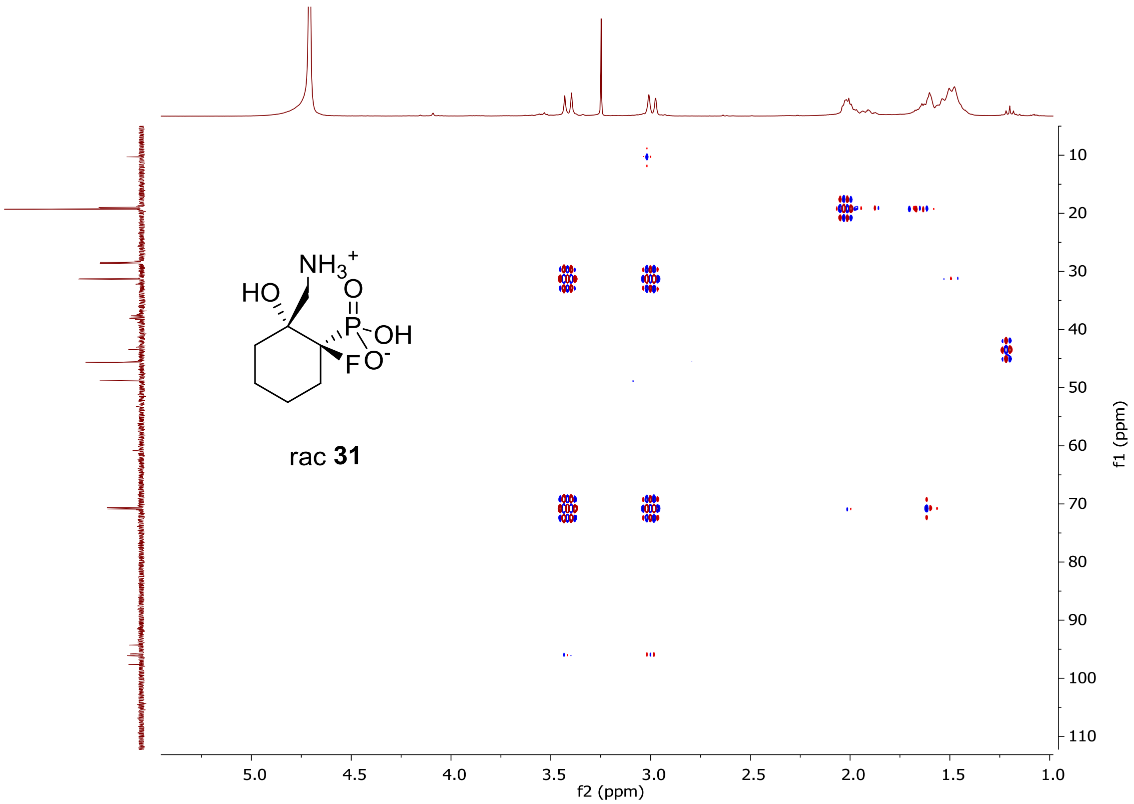

HMBC of rac **31** (D_2O)

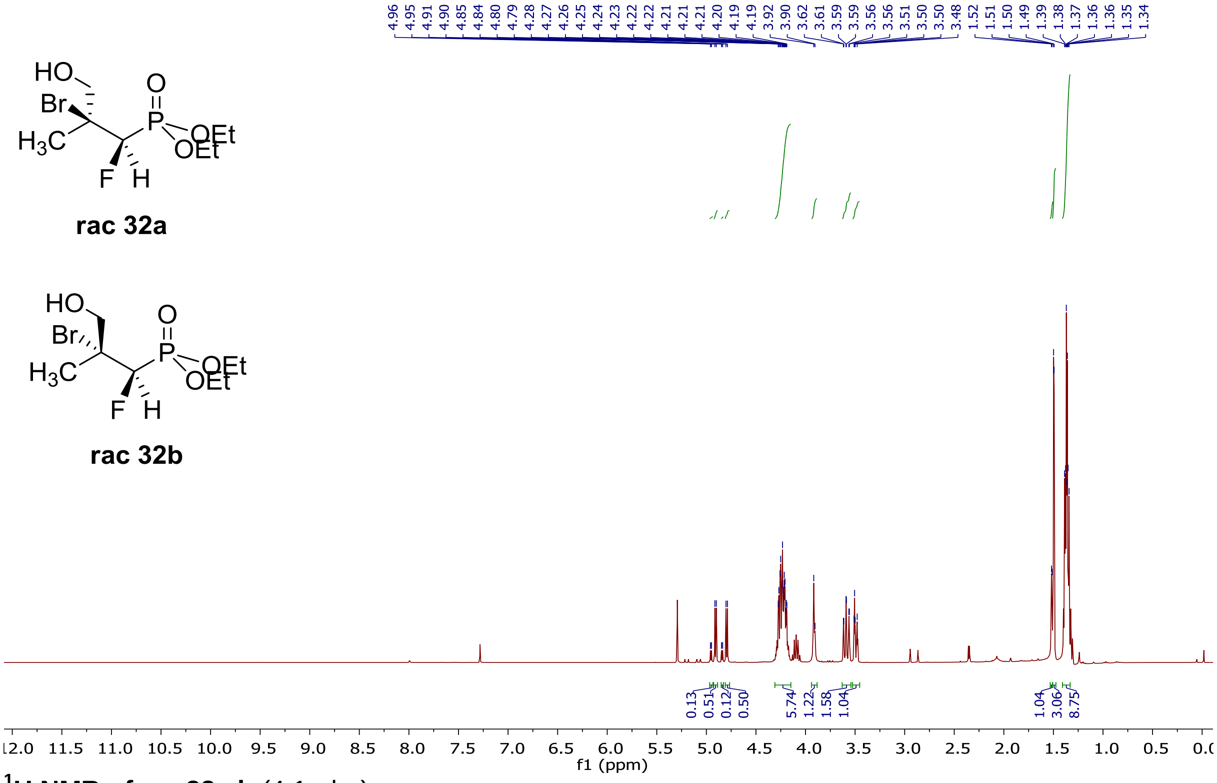

^{1}H NMR of rac **32a,b** (4:1, d.r.)

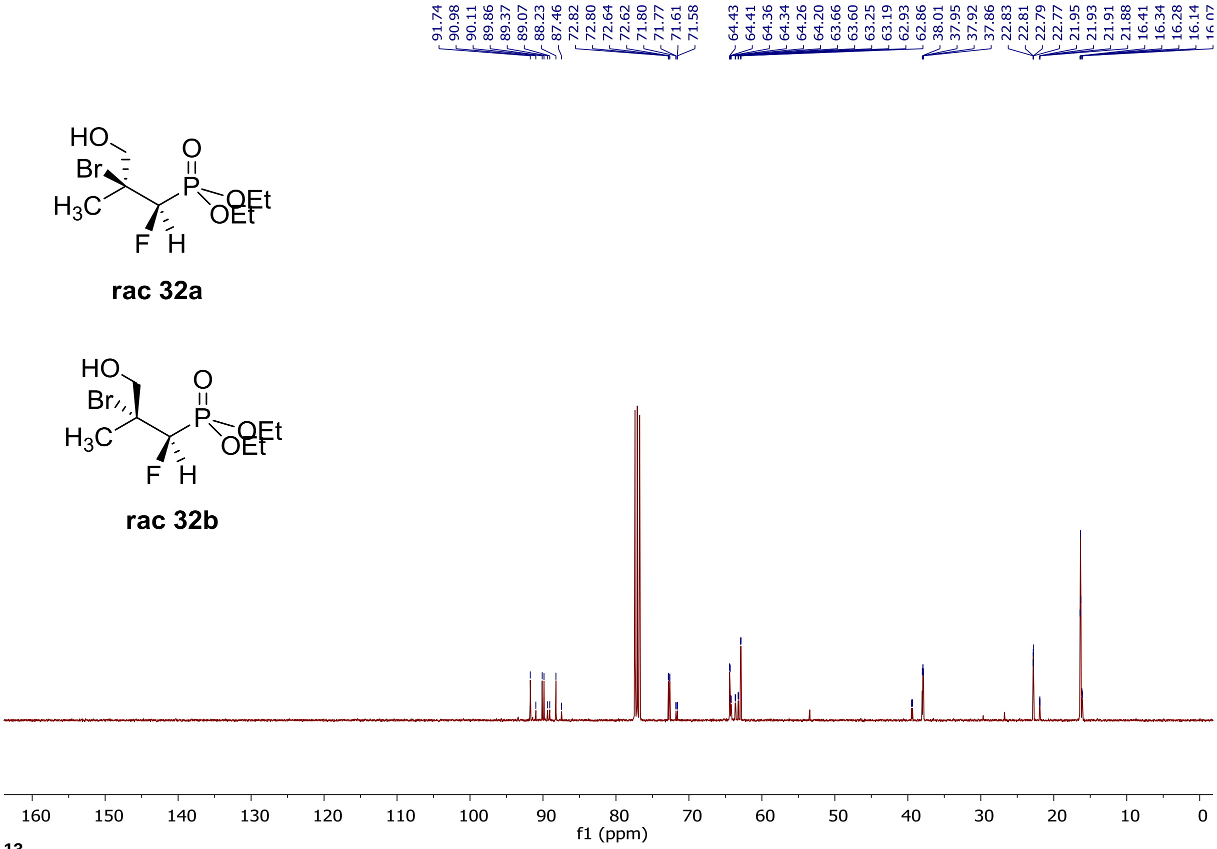

^{13}C NMR of rac **32a,b** (4:1, d.r.)

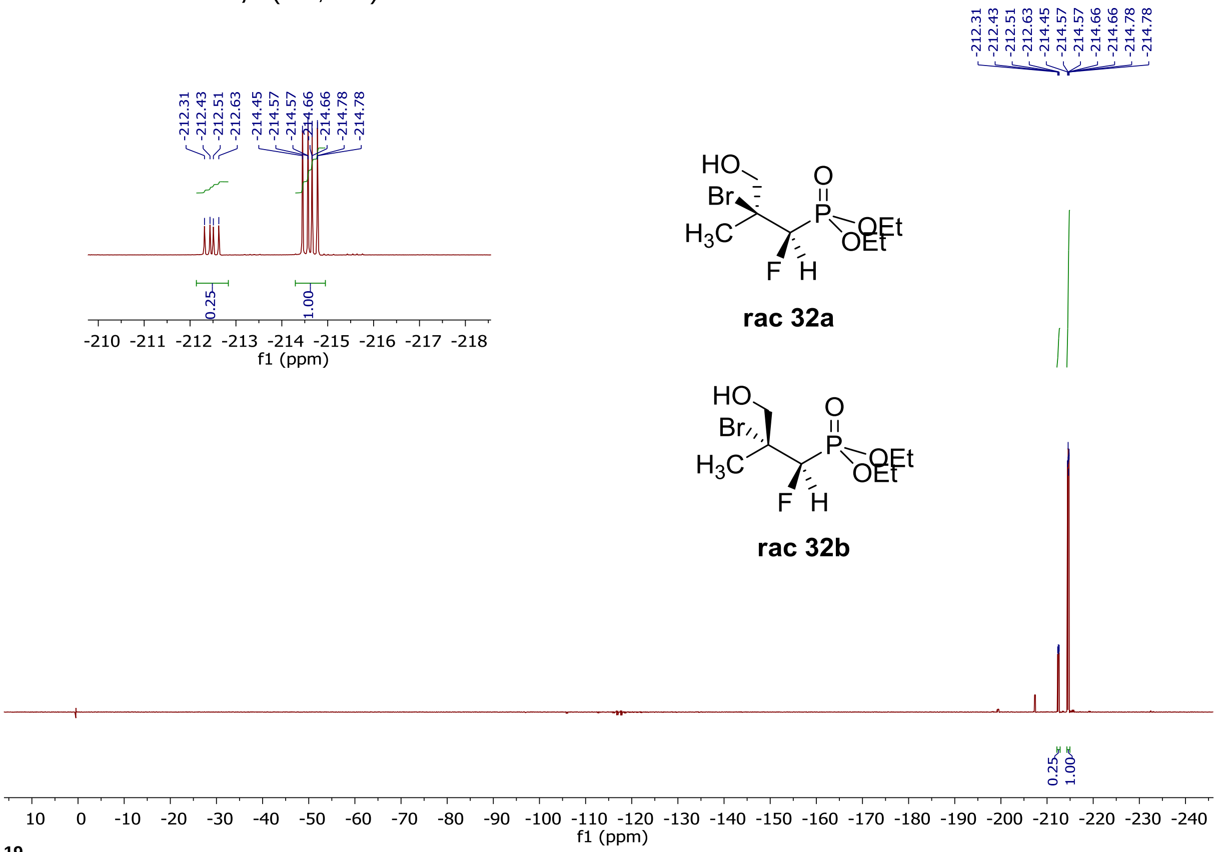

^{19}F NMR of rac **32a,b** (4:1, d.r.)

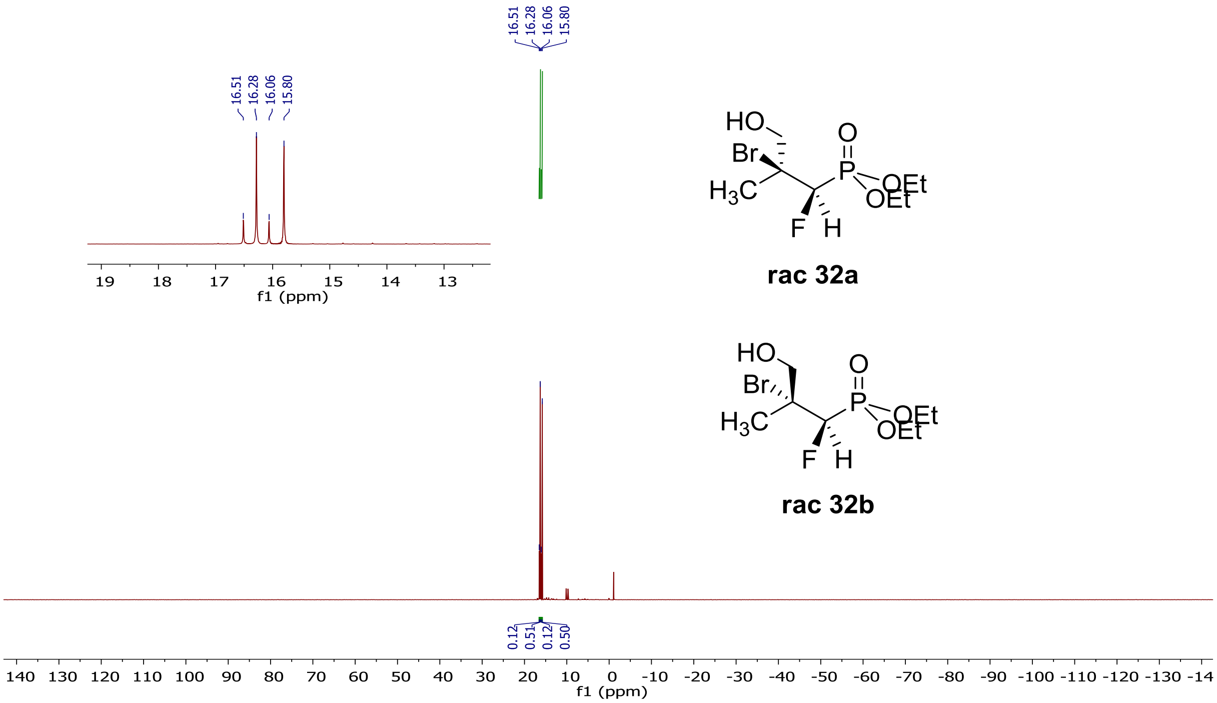

^{31}P{/^{1}H} NMR of rac **32a,b** (4:1, d.r.)

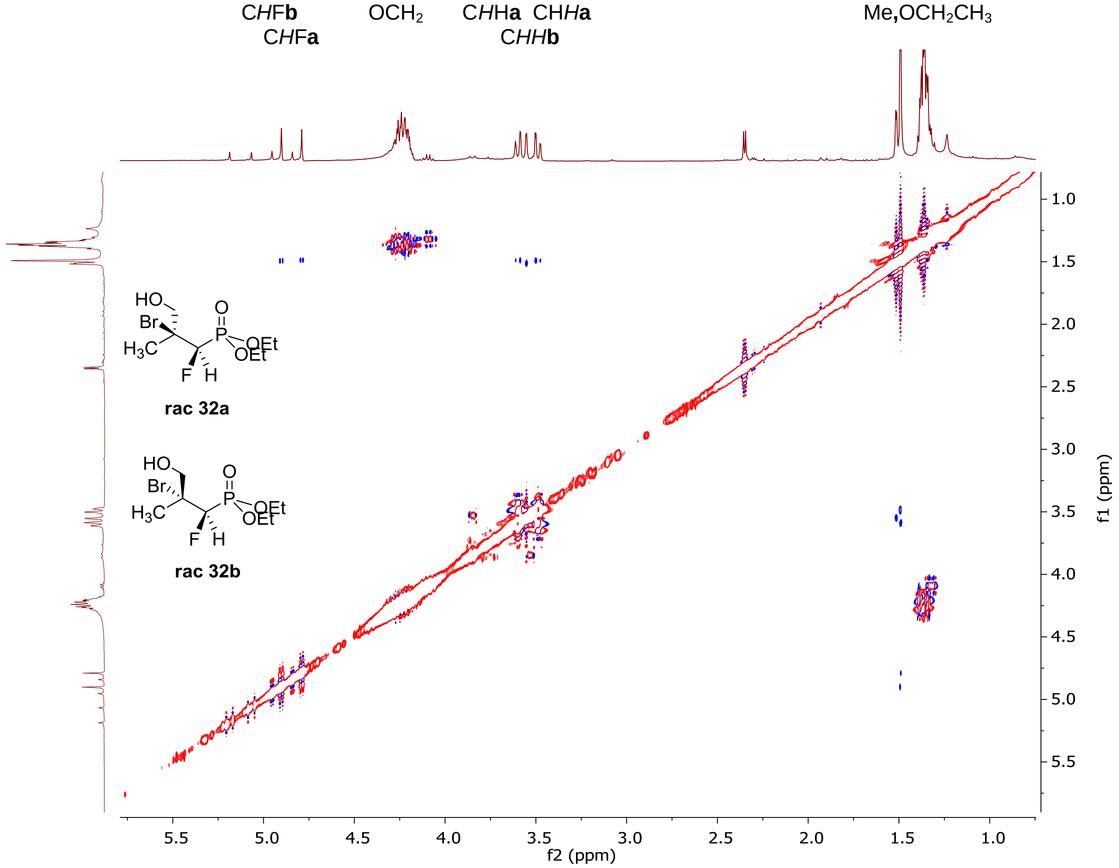

2D NOESY of rac **32a,b** (4:1, d.r.)

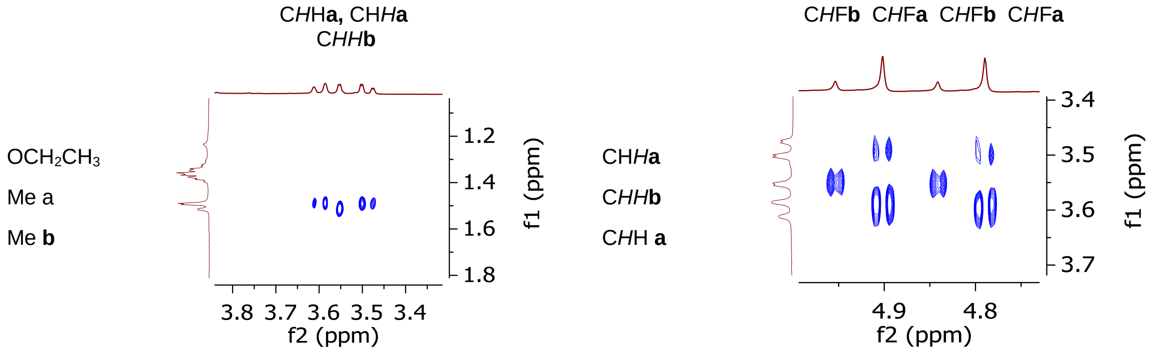

Diagnostic fragments of **2D NOESY** of rac **32a,b** (4:1, d.r.)

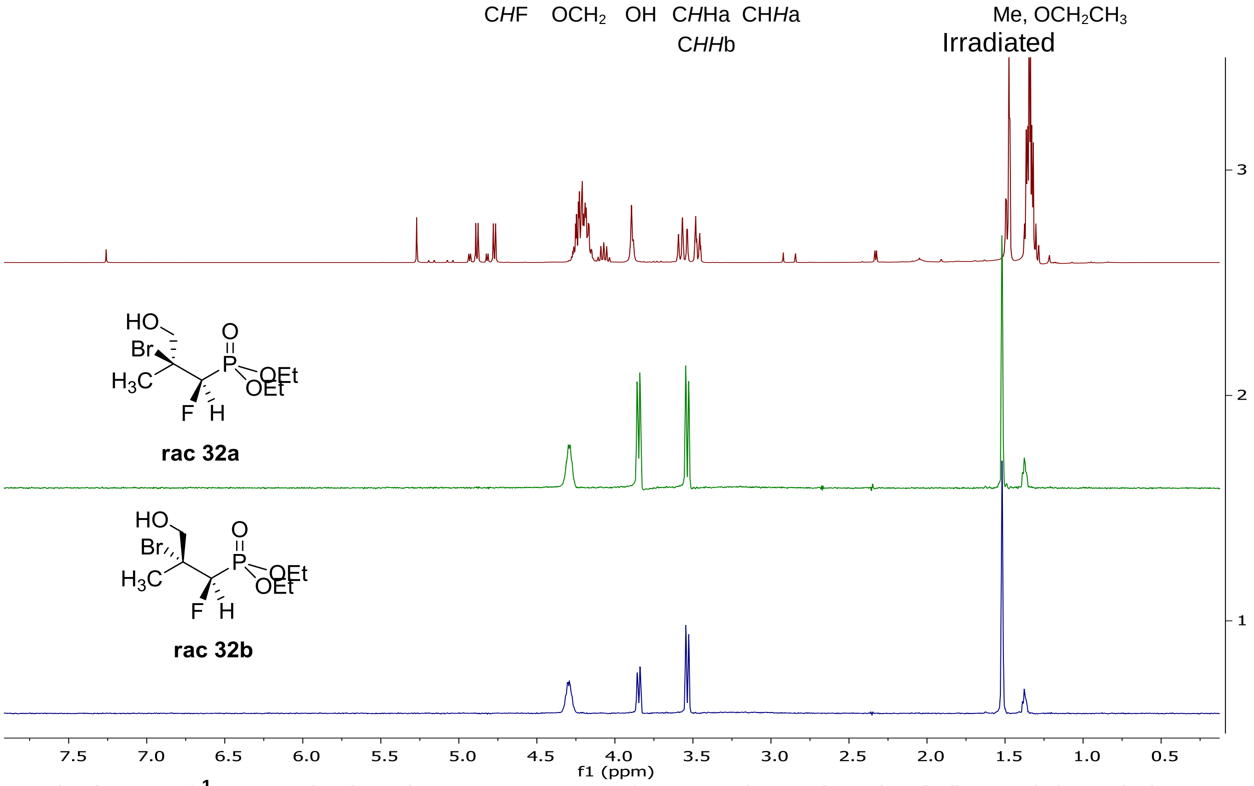

Stacked plots of ^{1}**H NMR** (top) and **1D H-F HOESY** of rac **32a** (center) and **32b** (bottom) (4:1, d.r.)

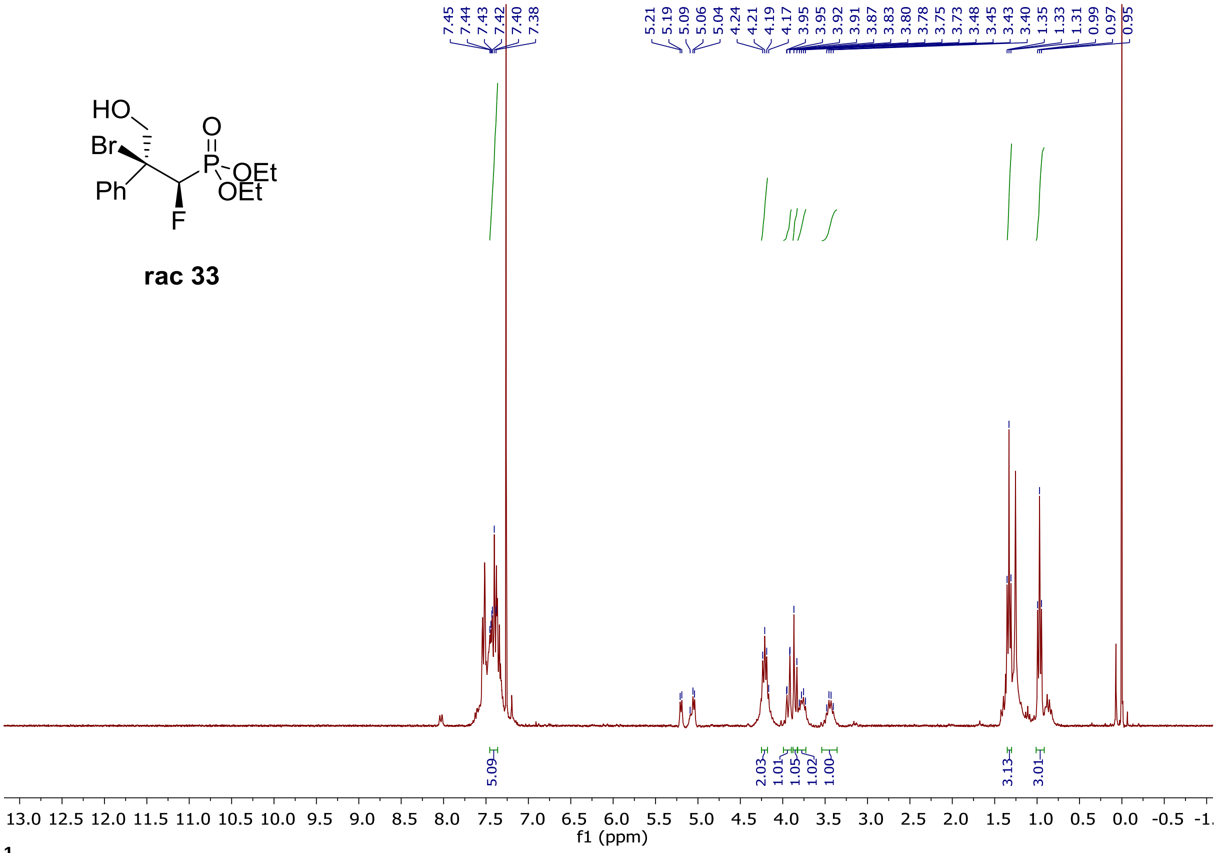

^{1}H NMR of rac **33**

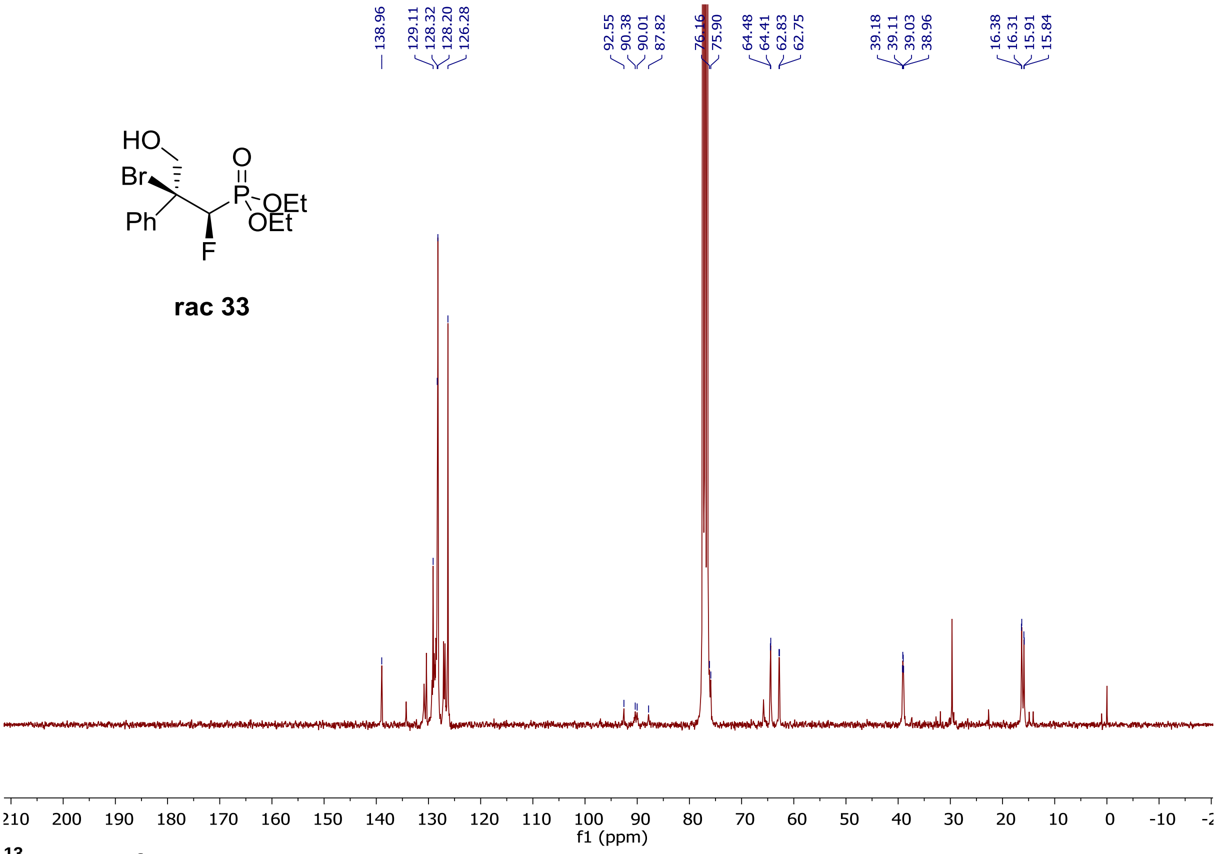

^{13}C NMR of rac **33**

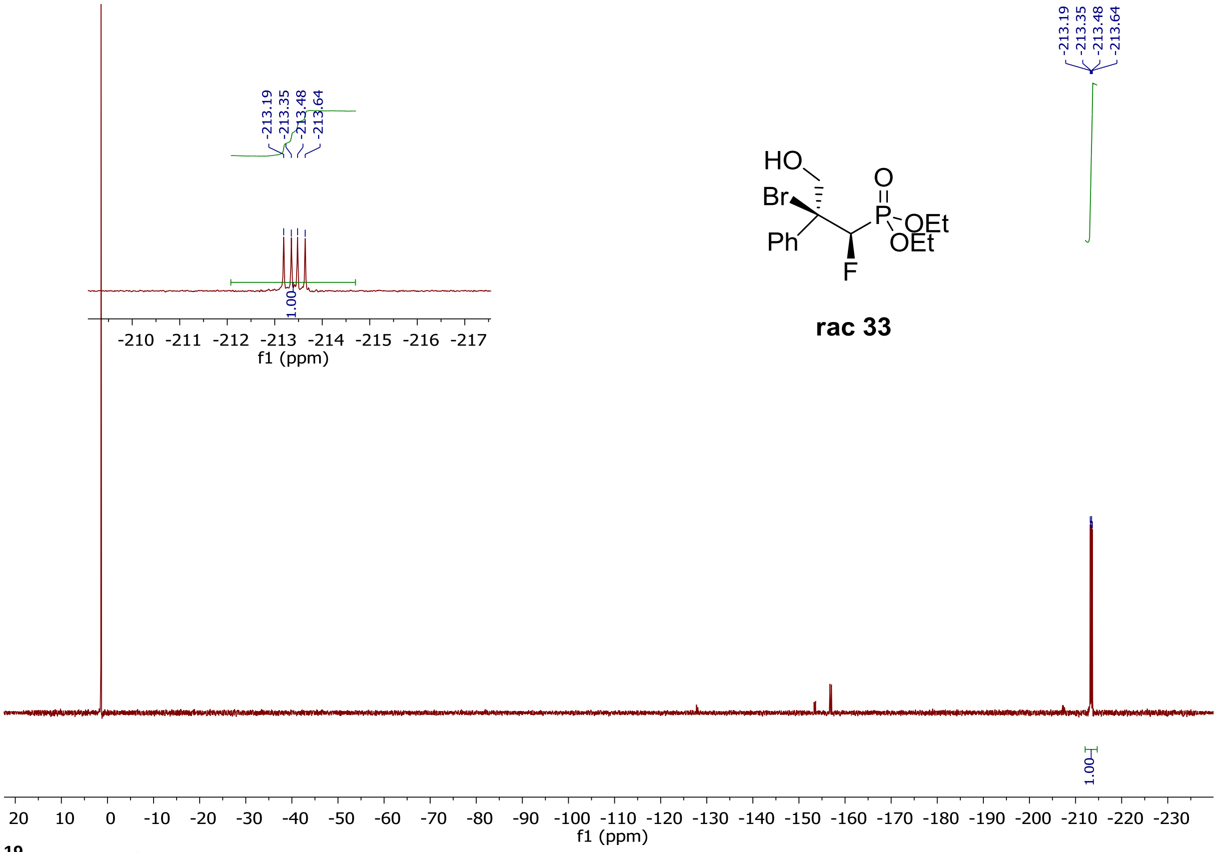

^{19}F NMR of rac **33**

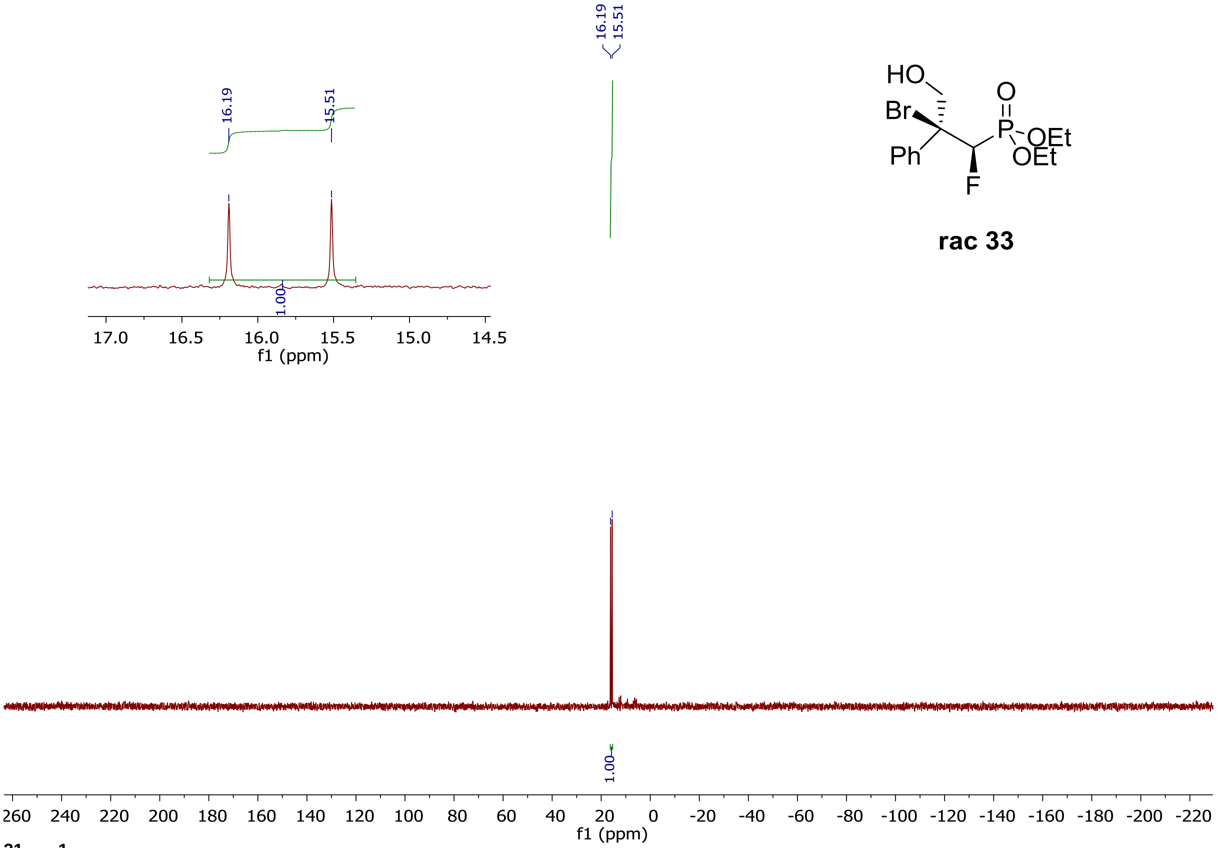

^{31}P{/^{1}H} NMR of rac **33**

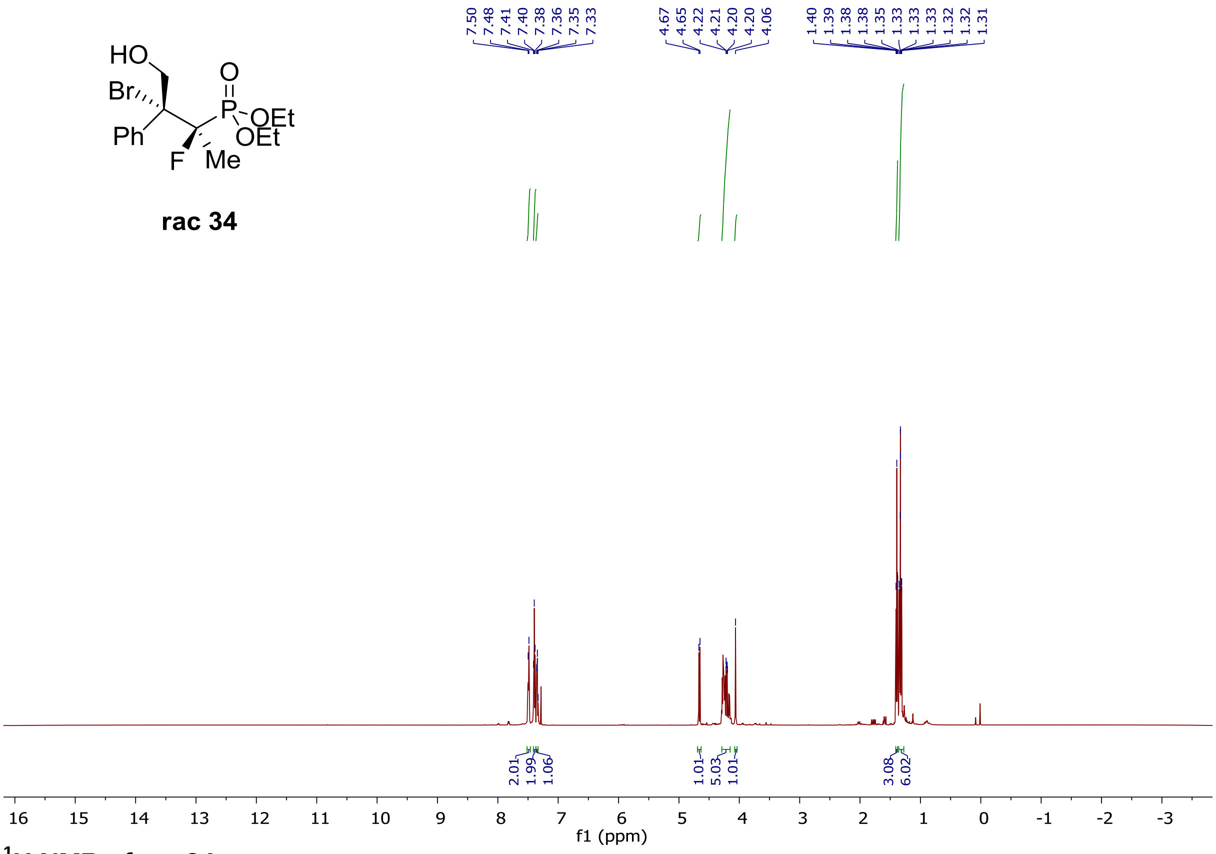

^{1}H NMR of rac **34**

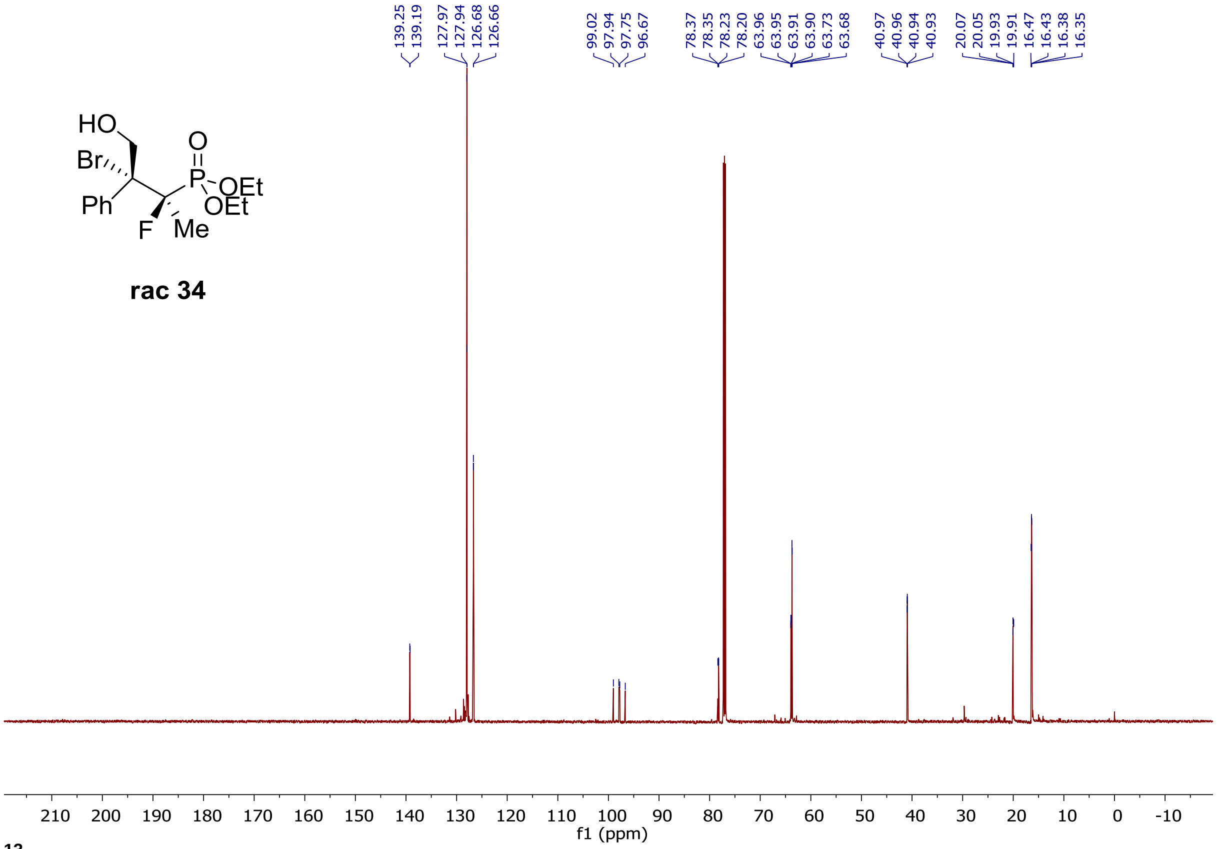

^{13}C NMR of rac **34**

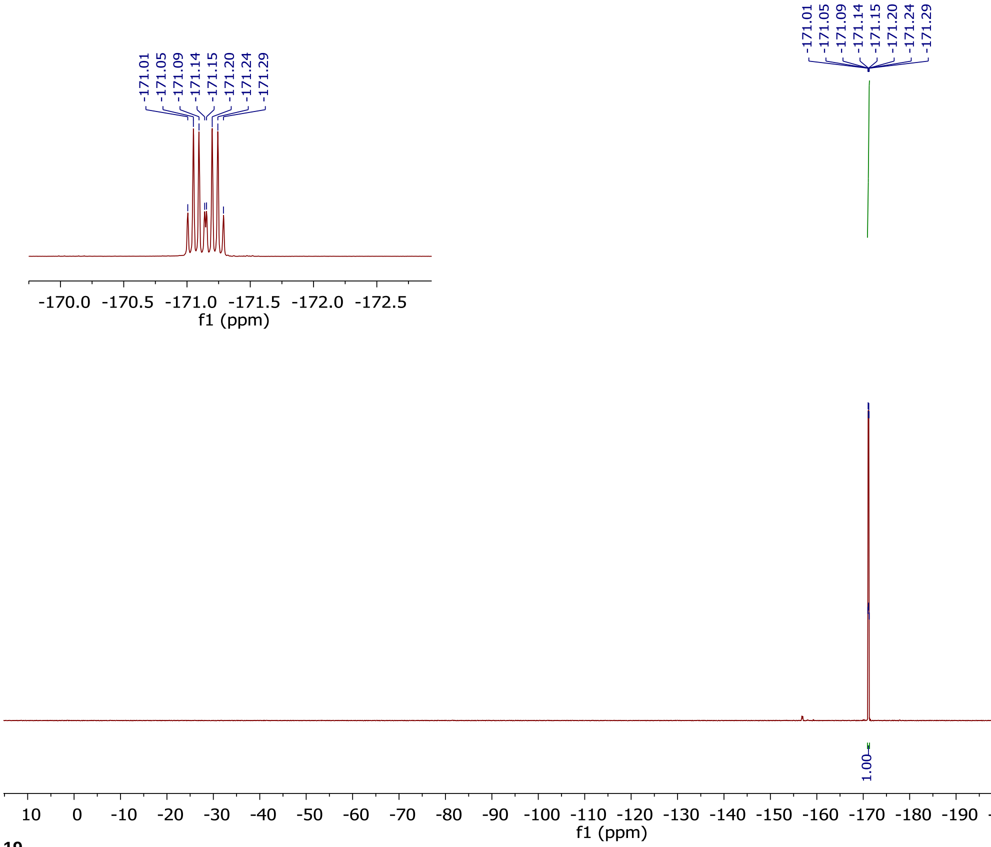

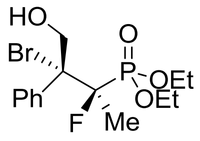

rac 34

^{19}F NMR of rac **34**

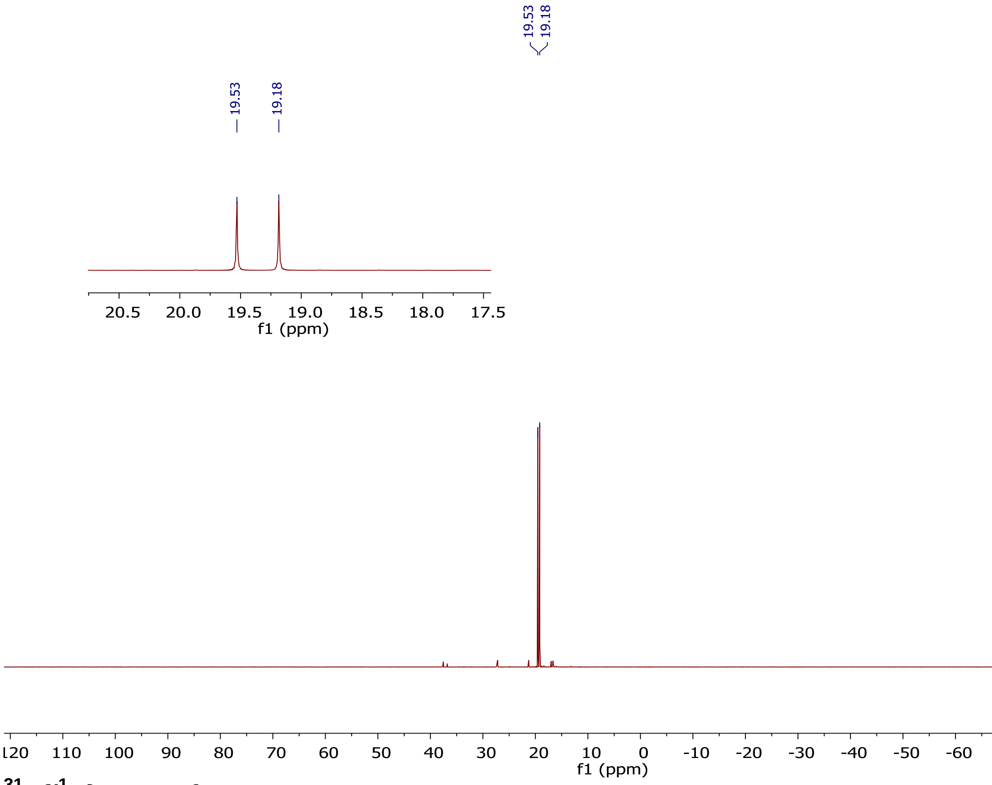

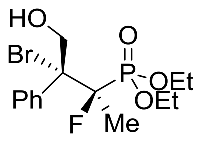

rac 34

^{31}P{/^{1}H} NMR of rac **34**

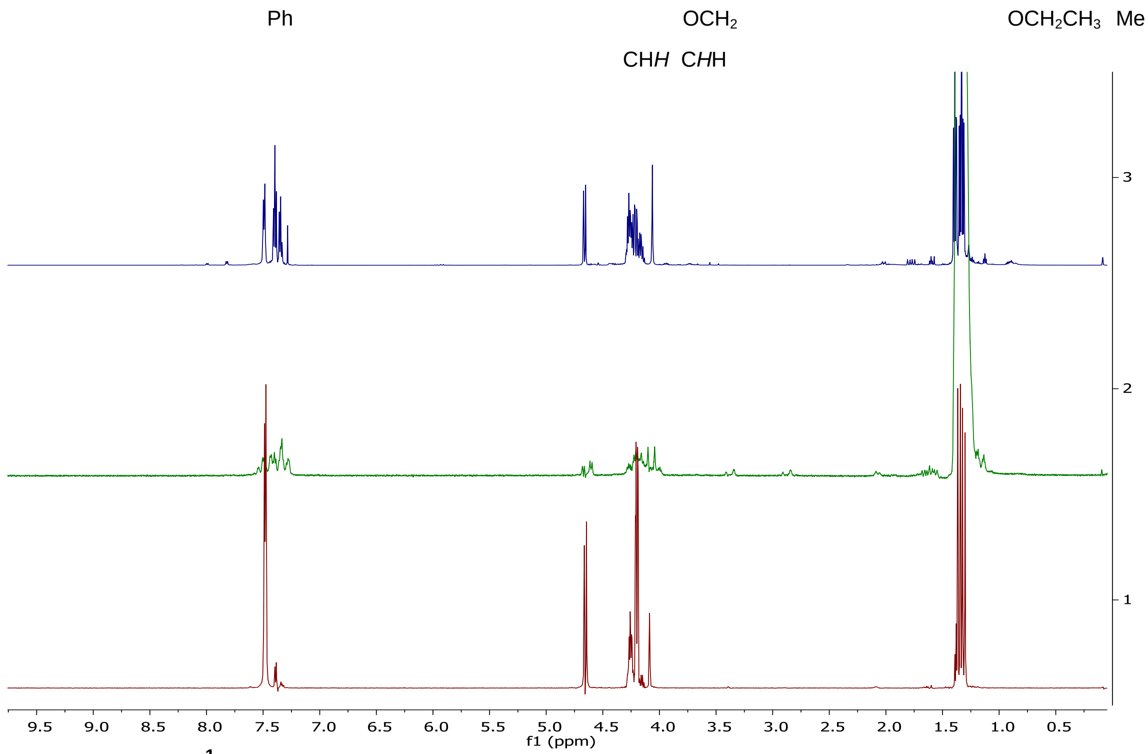

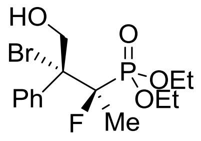

rac 34

Stacked plots of ^{1}**H NMR** (top) and **1D NOESY** (irradiated Me, center) and **1D H-F HOESY** (bottom)of rac **34**

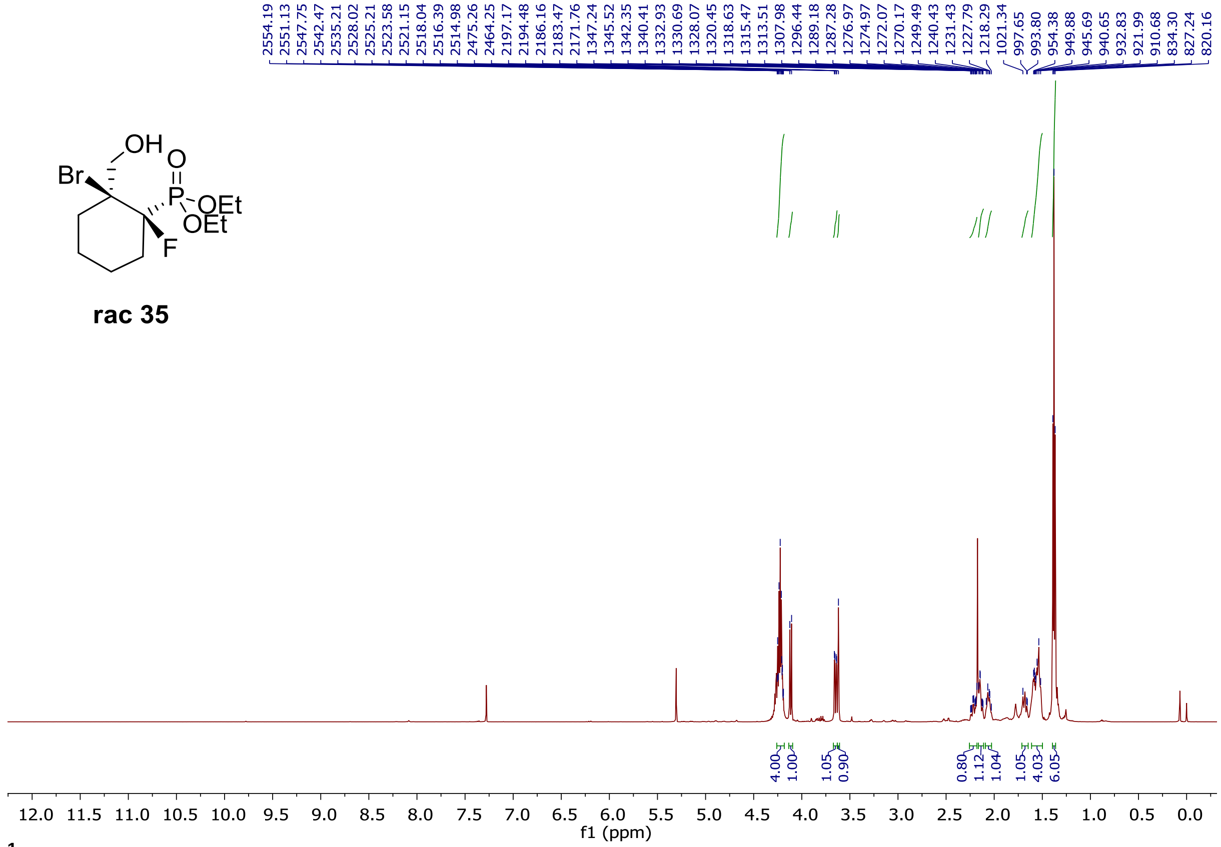

^{1}**H NMR of** rac **35**

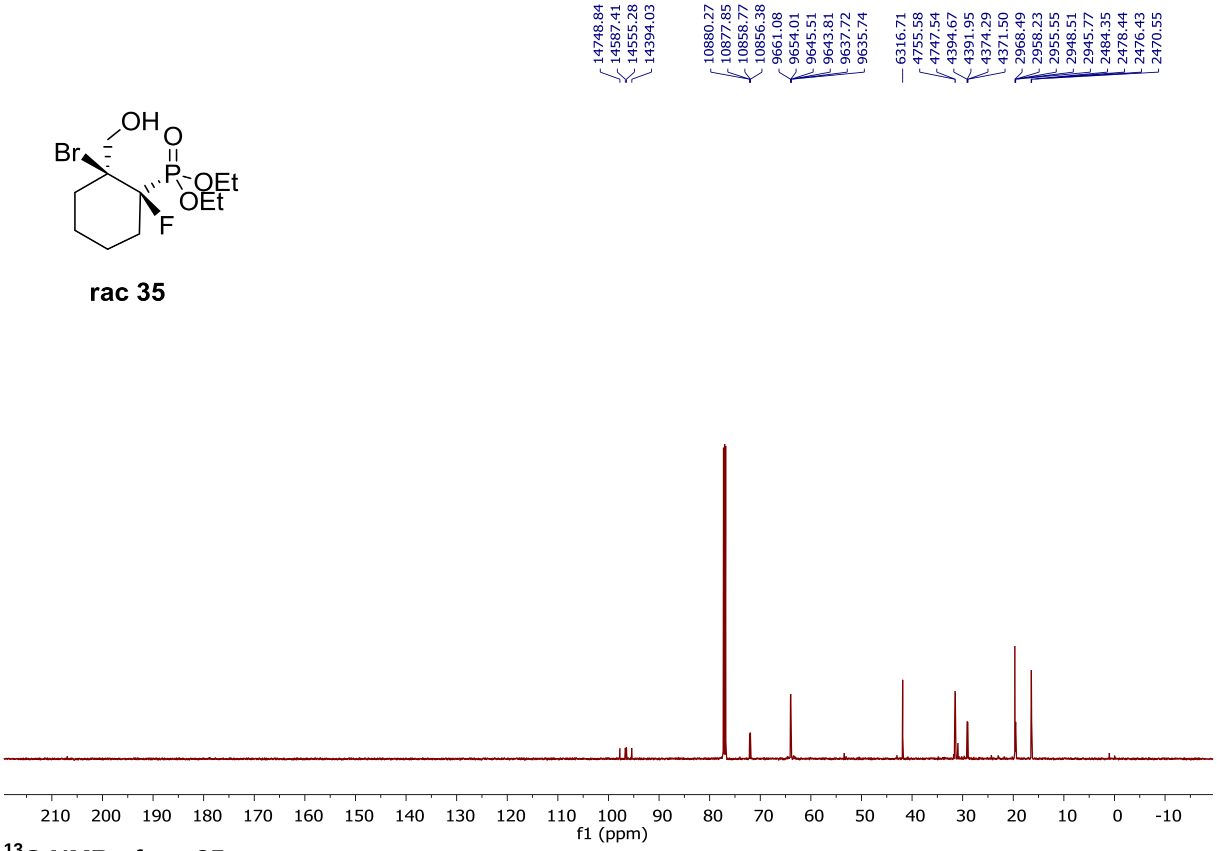

^{13}C NMR of rac **35**

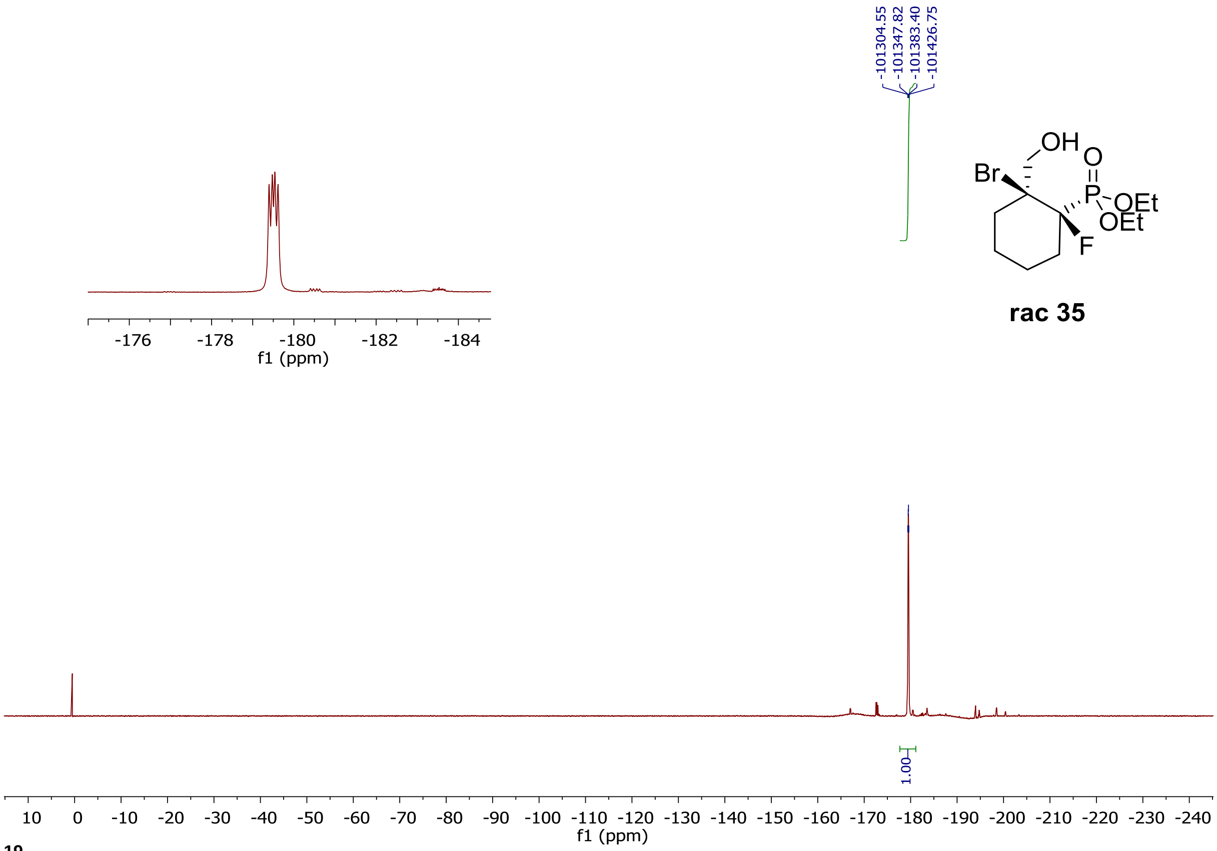

^{19}F NMR of rac **35**

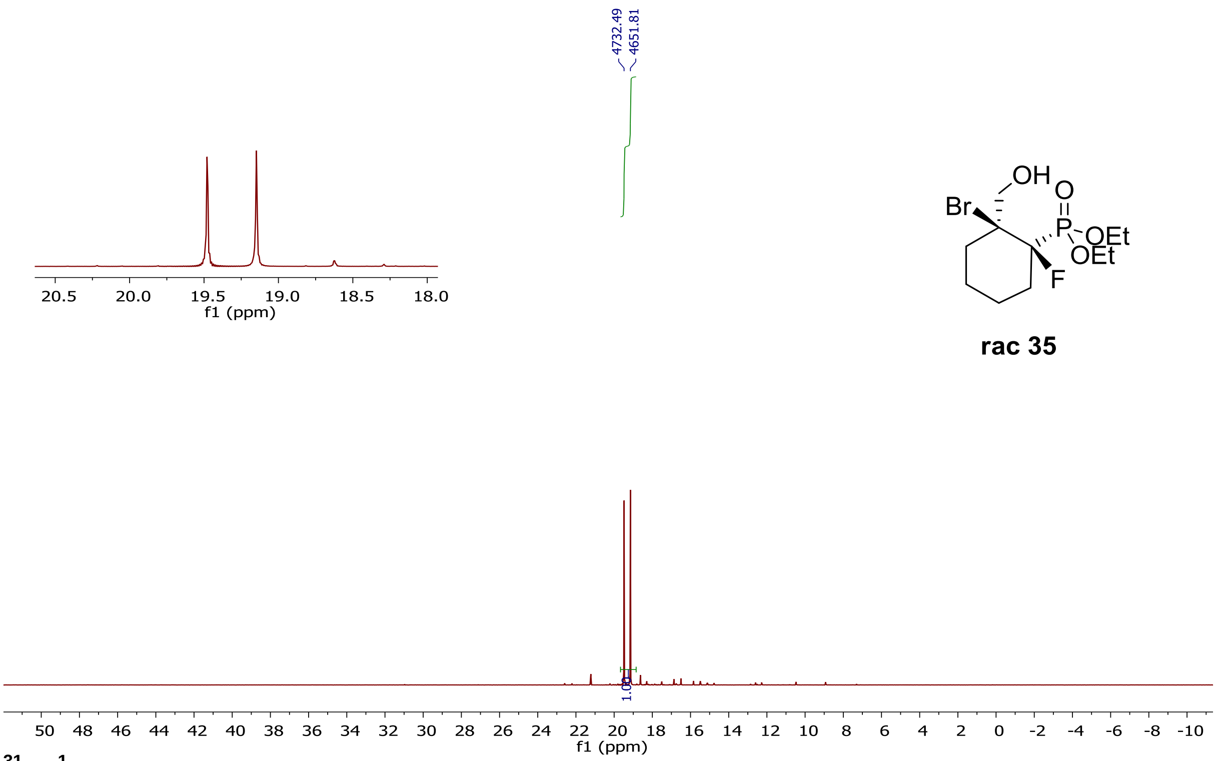

^{31}P{/^{1}H} NMR of rac **35**

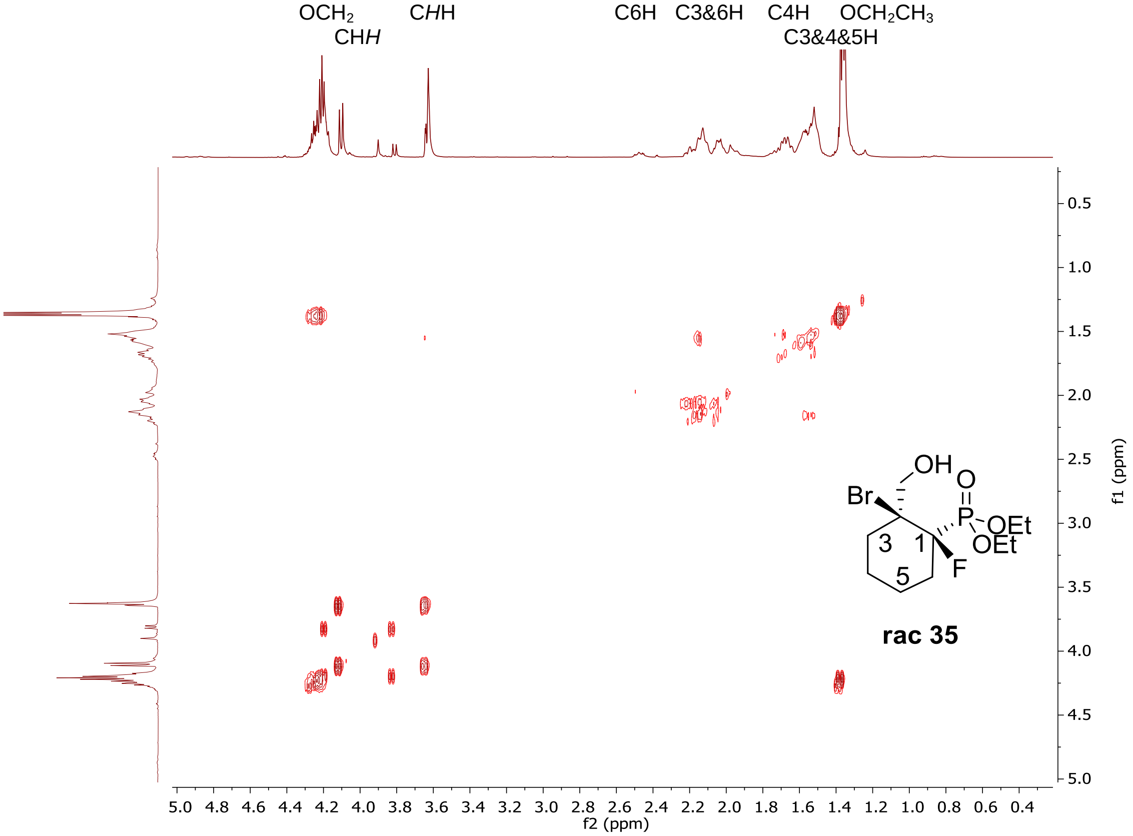

COSY of rac **35**

OCH$_2$ C*H*H C6H C3&6H C4H OCH$_2$CH$_3$

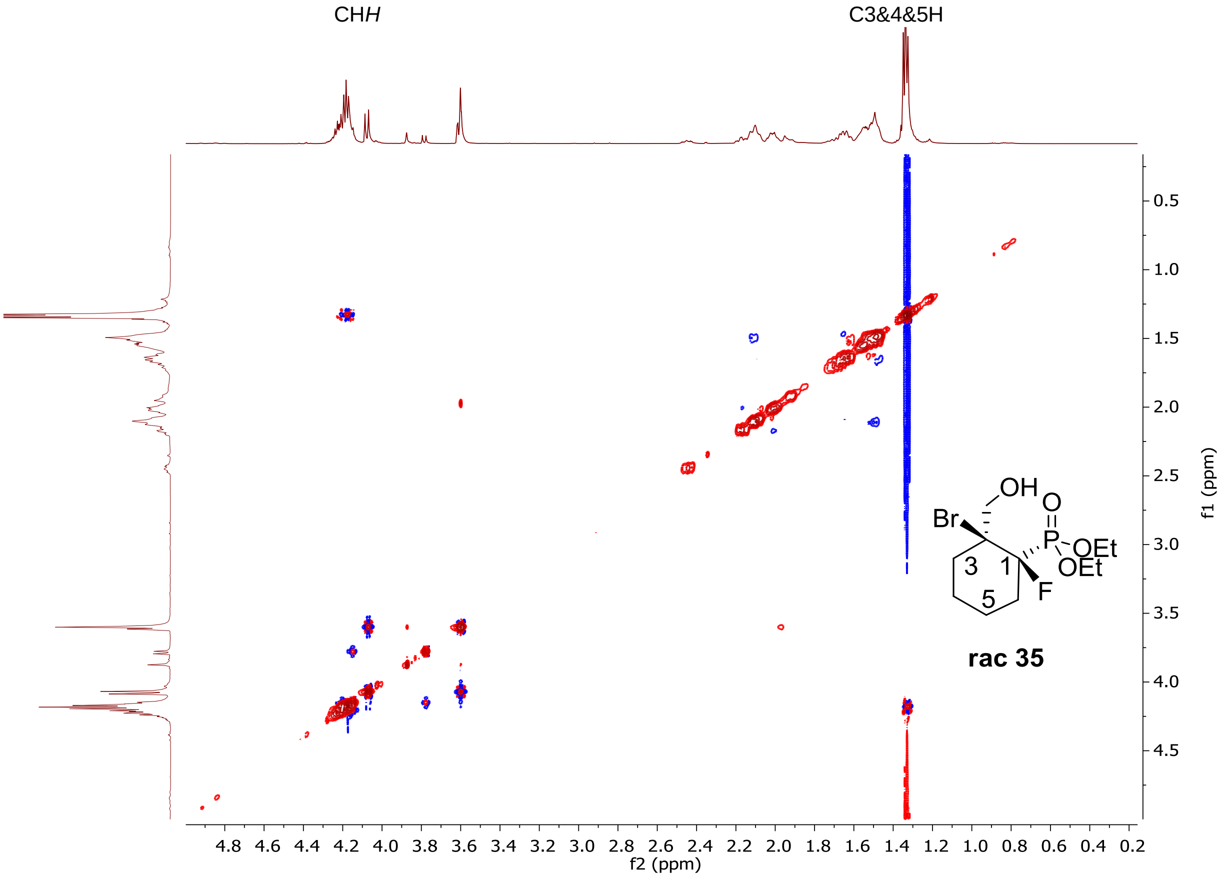

2D NOESY of rac **35**

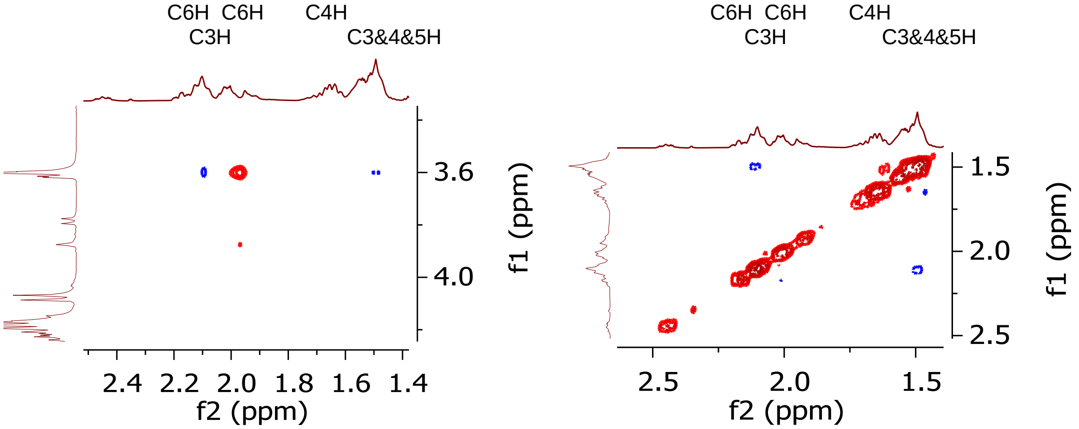

Diagnostic fragments of **2D NOESY of** rac **35**

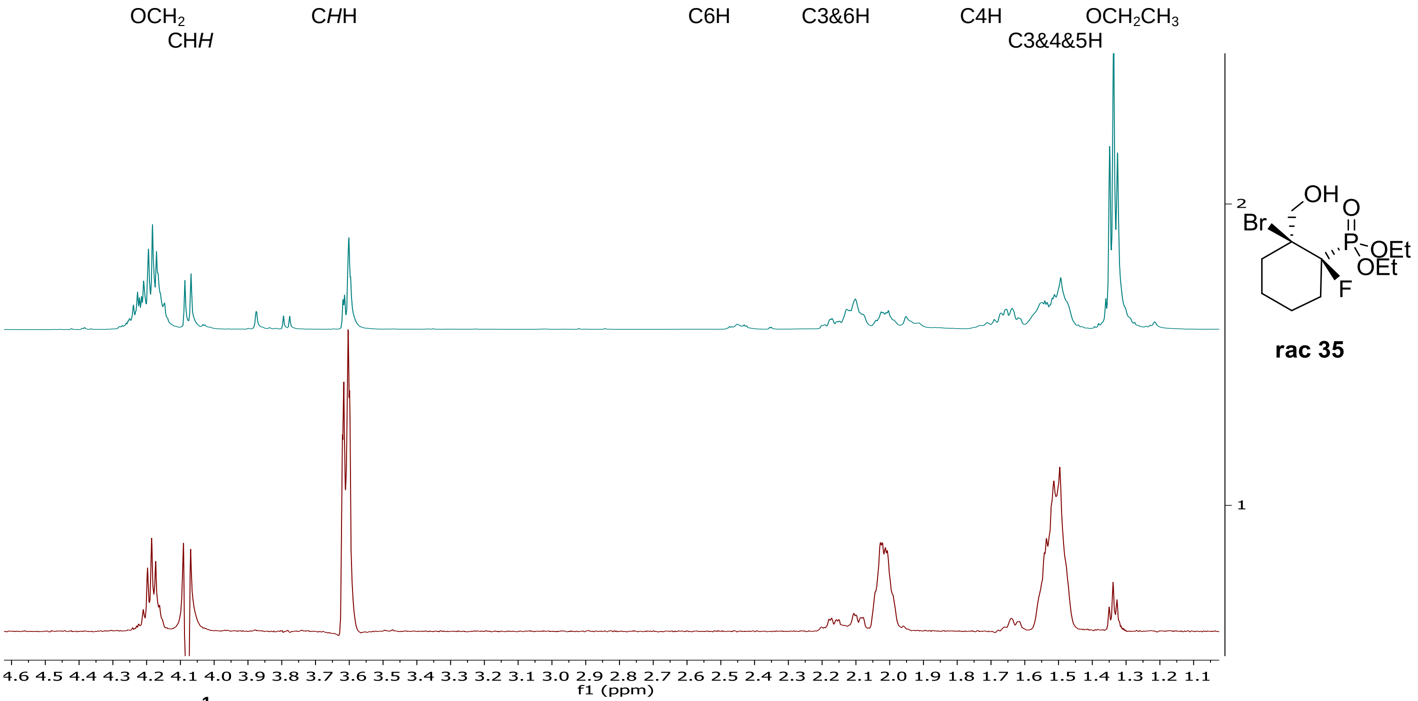

Stacked plots of [1]**H NMR** (top) and **1D H-F HOESY** (bottom) of rac **35**

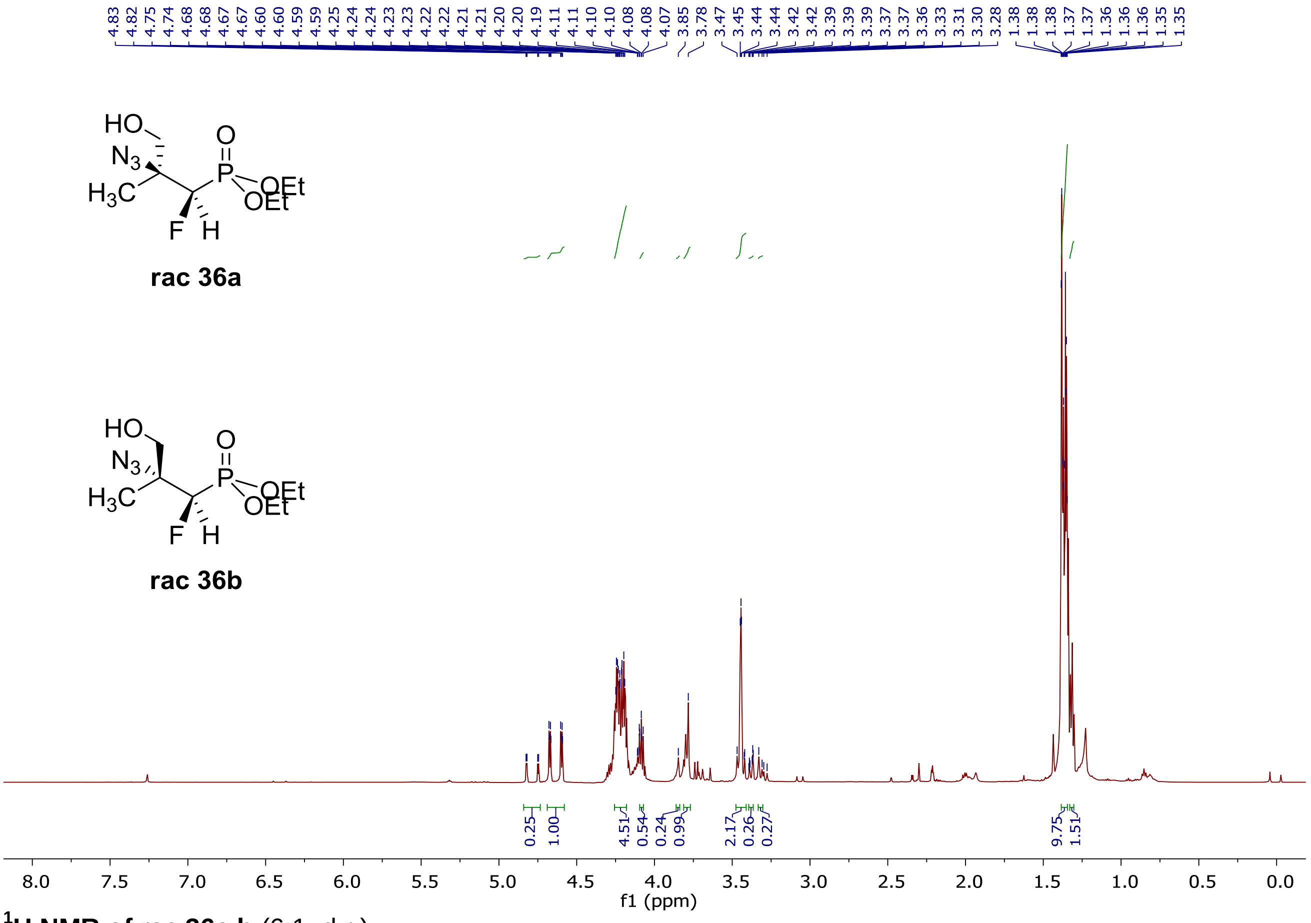

[1]**H NMR of** rac **36a,b** (6:1, d.r.)

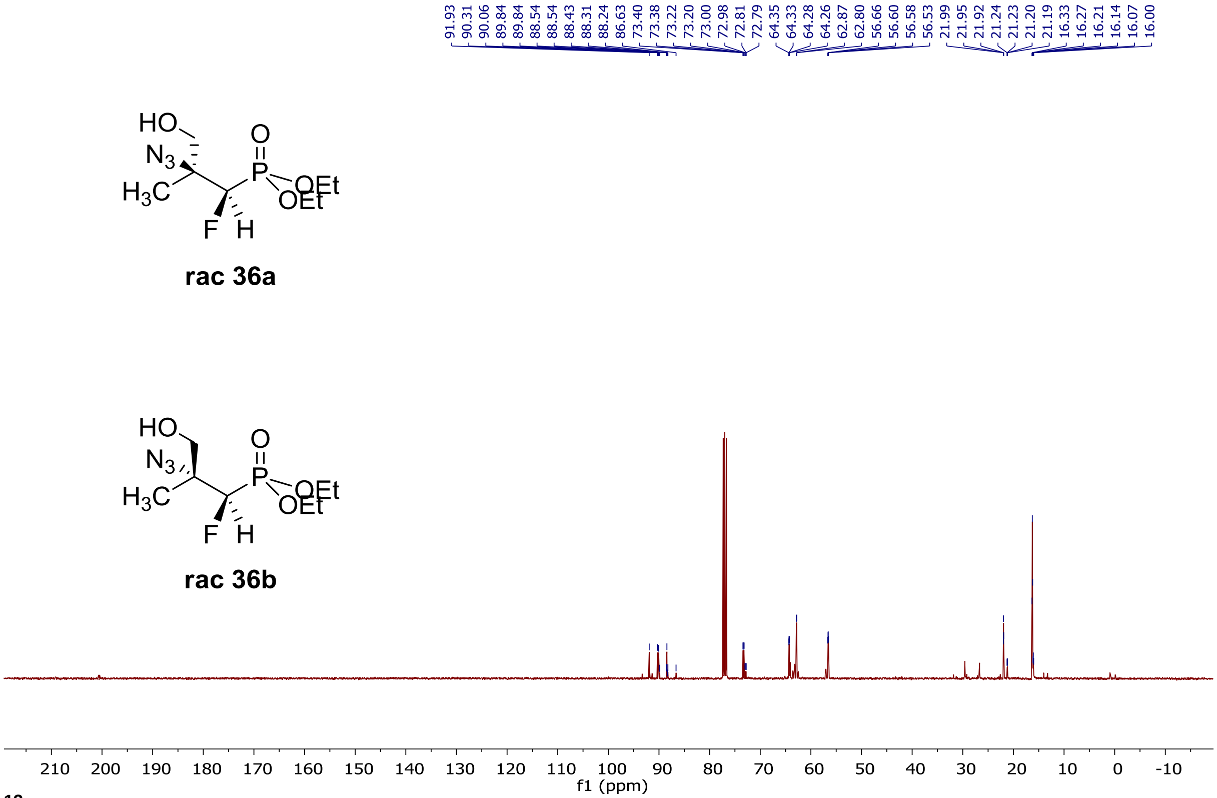

^{13}C NMR of rac **36a,b** (6:1, d.r.)

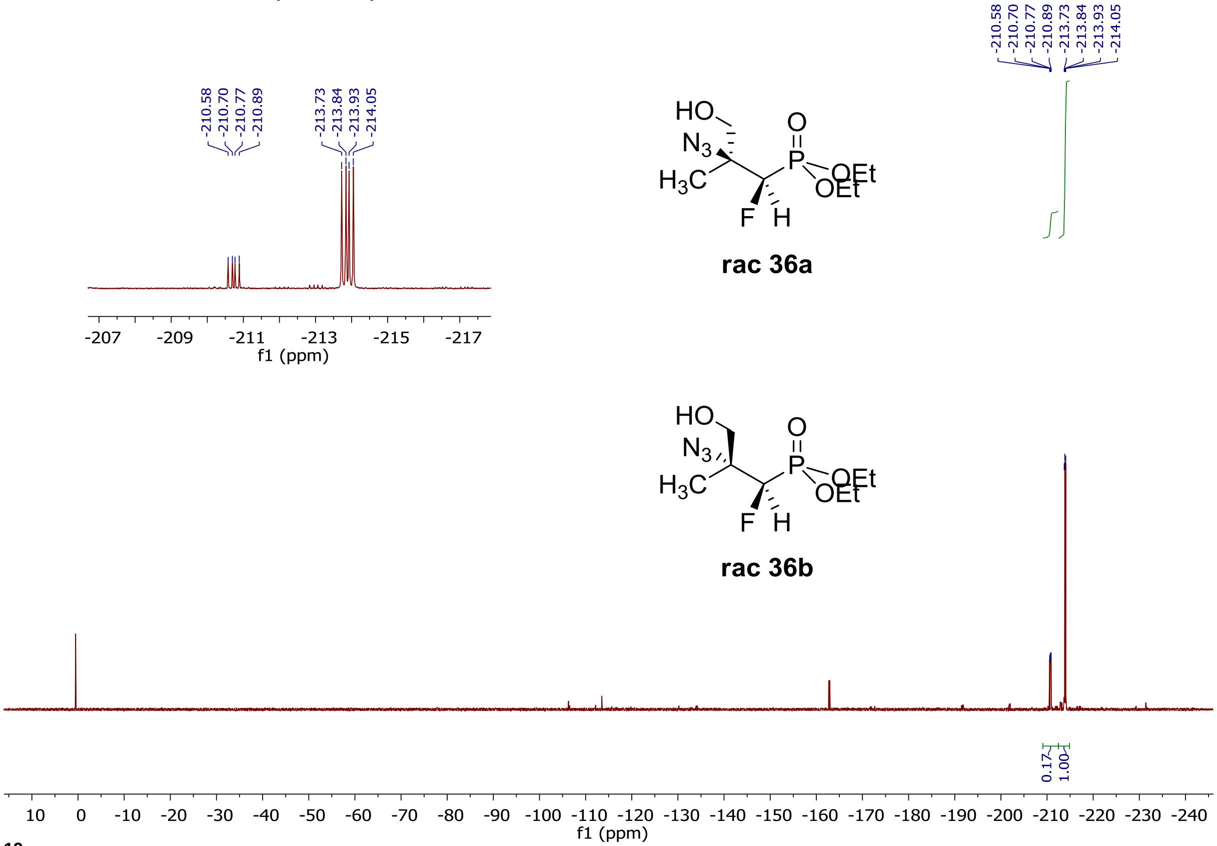

^{19}F NMR of rac **36a,b** (6:1, d.r.)

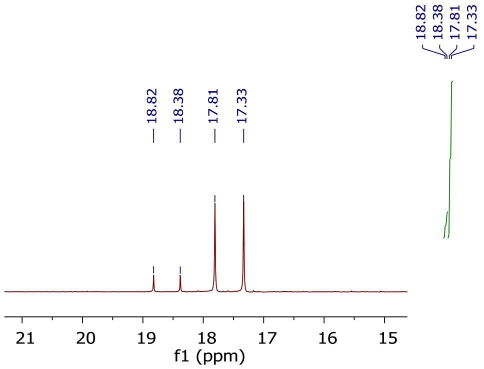

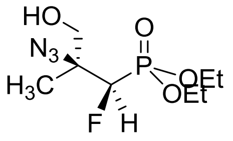

rac 36a

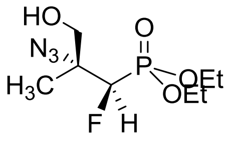

rac 36b

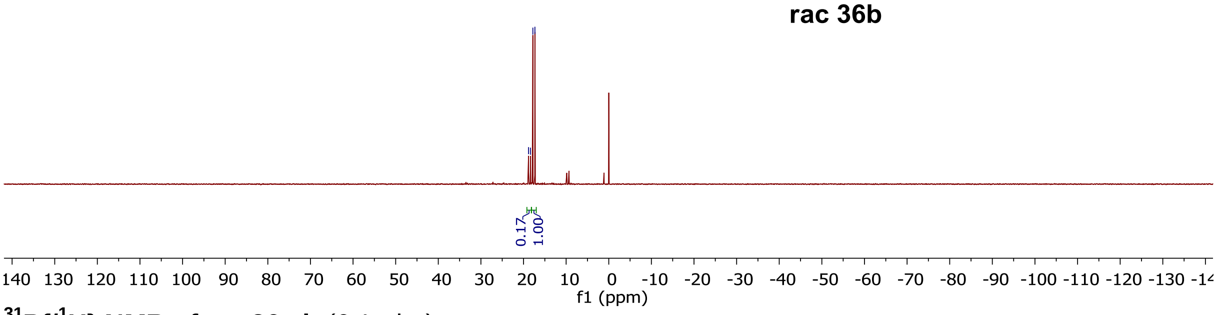

$^{31}P\{/^{1}H\}$ NMR of rac **36a,b** (6:1, d.r.)

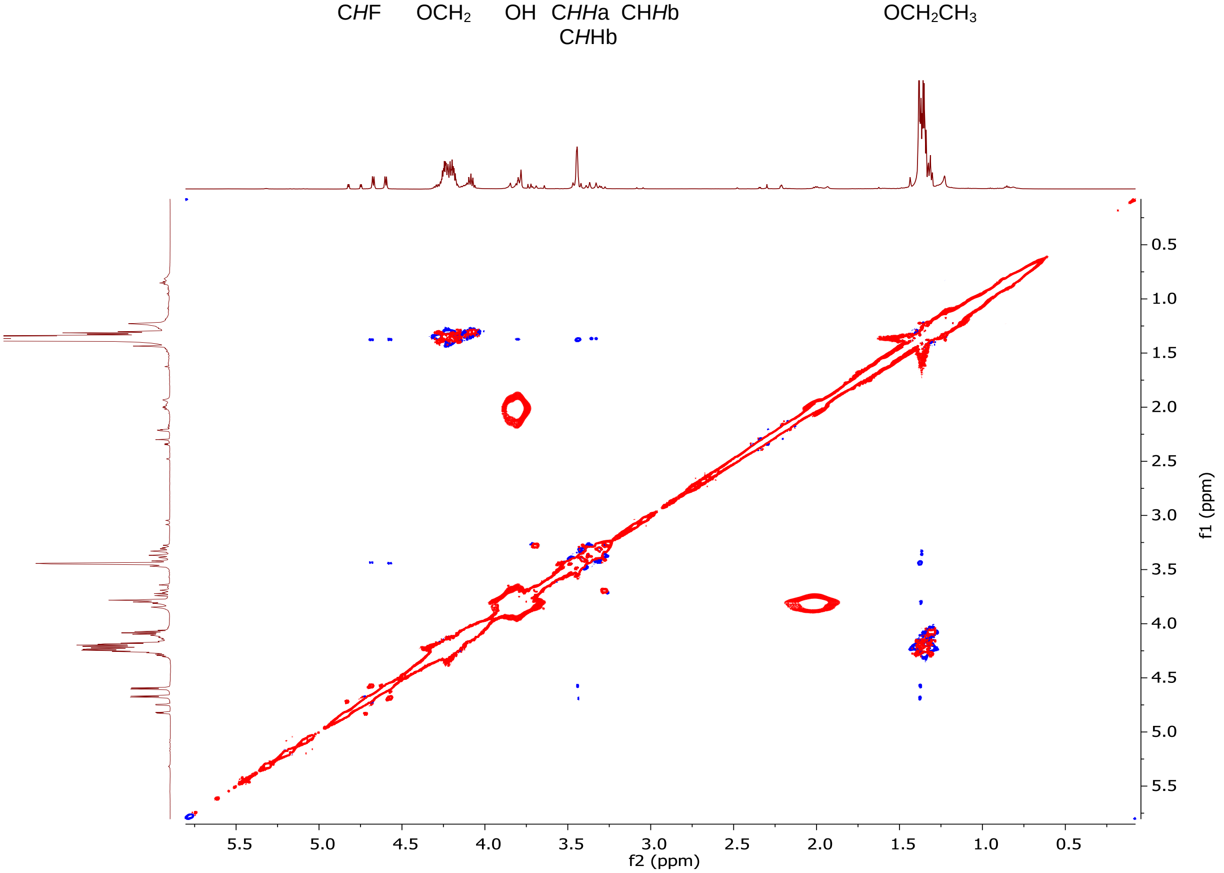

2D NOESY of rac **36a,b** (6:1, d.r.)

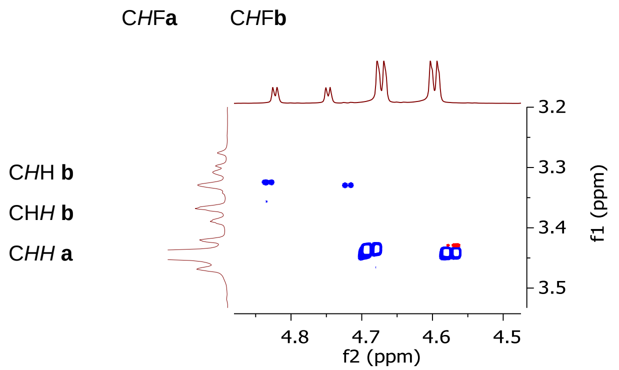

Diagnostic fragments of **2D NOESY of** rac **36a,b** (6:1, d.r.)

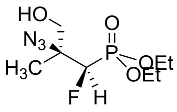

rac 36a

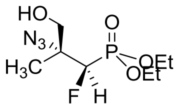

rac 36b

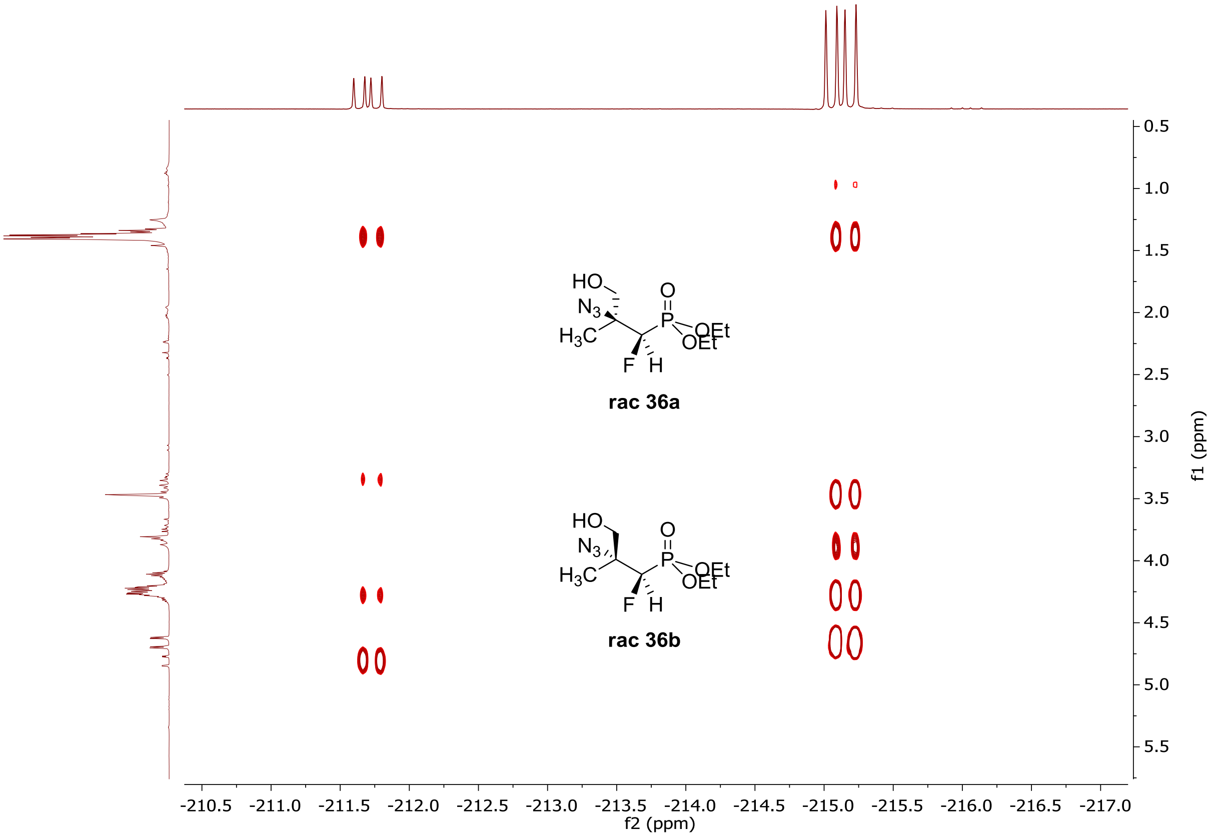

2D H-F HOESY of rac **36a,b** (6:1, d.r.)

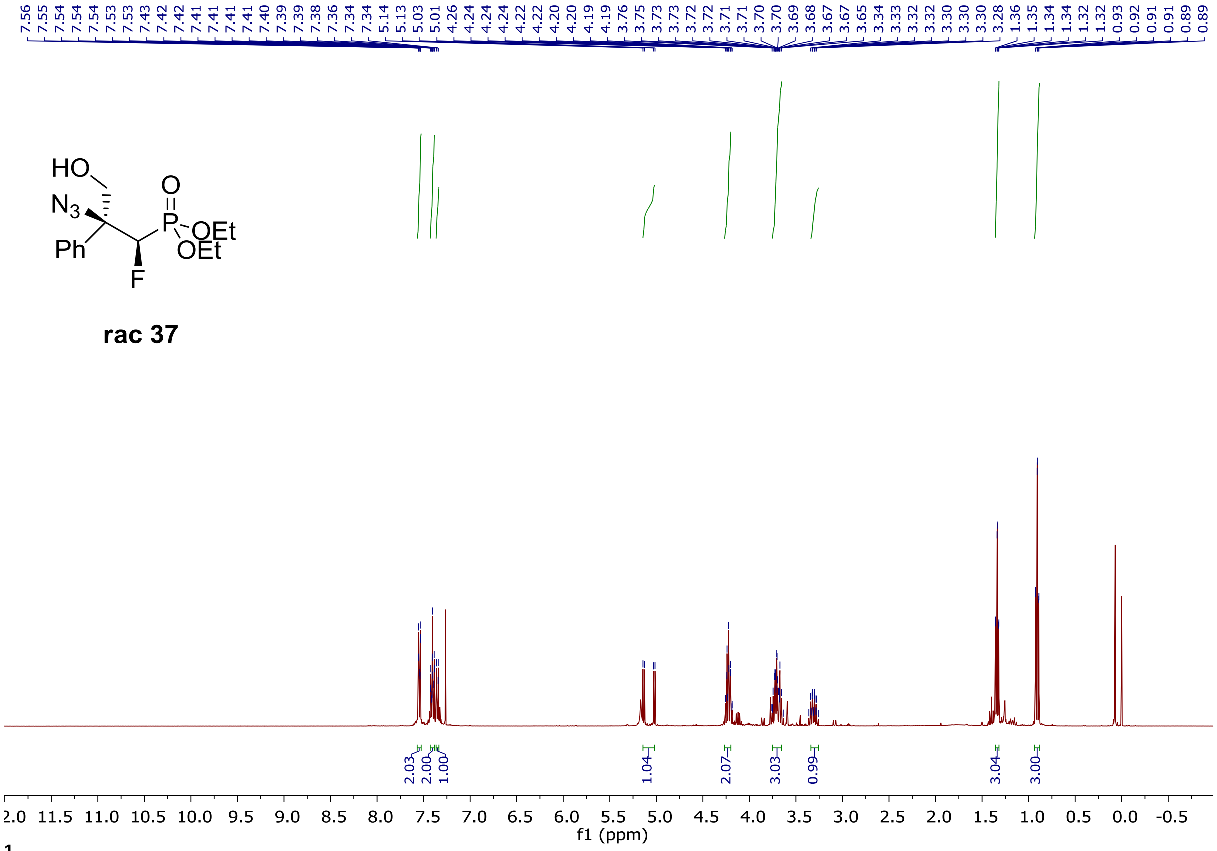

^{1}H NMR of rac **37**

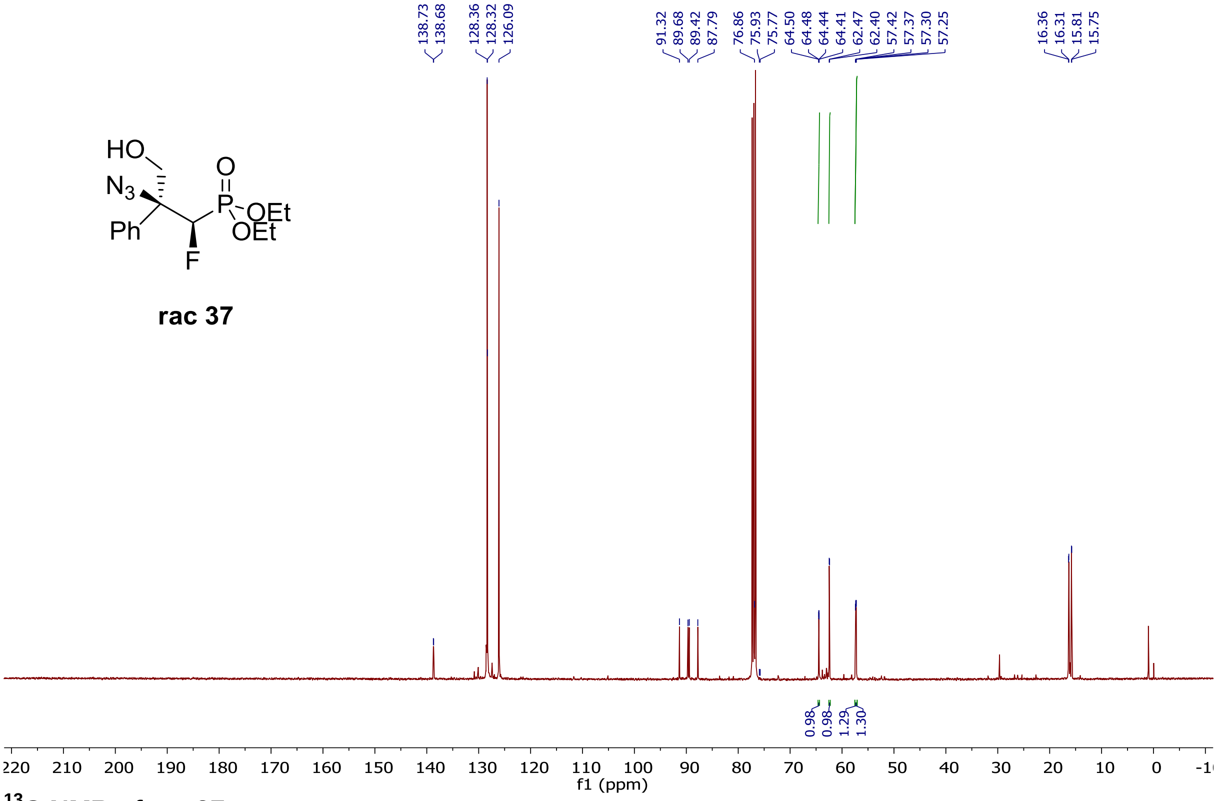

^{13}C NMR of rac **37**

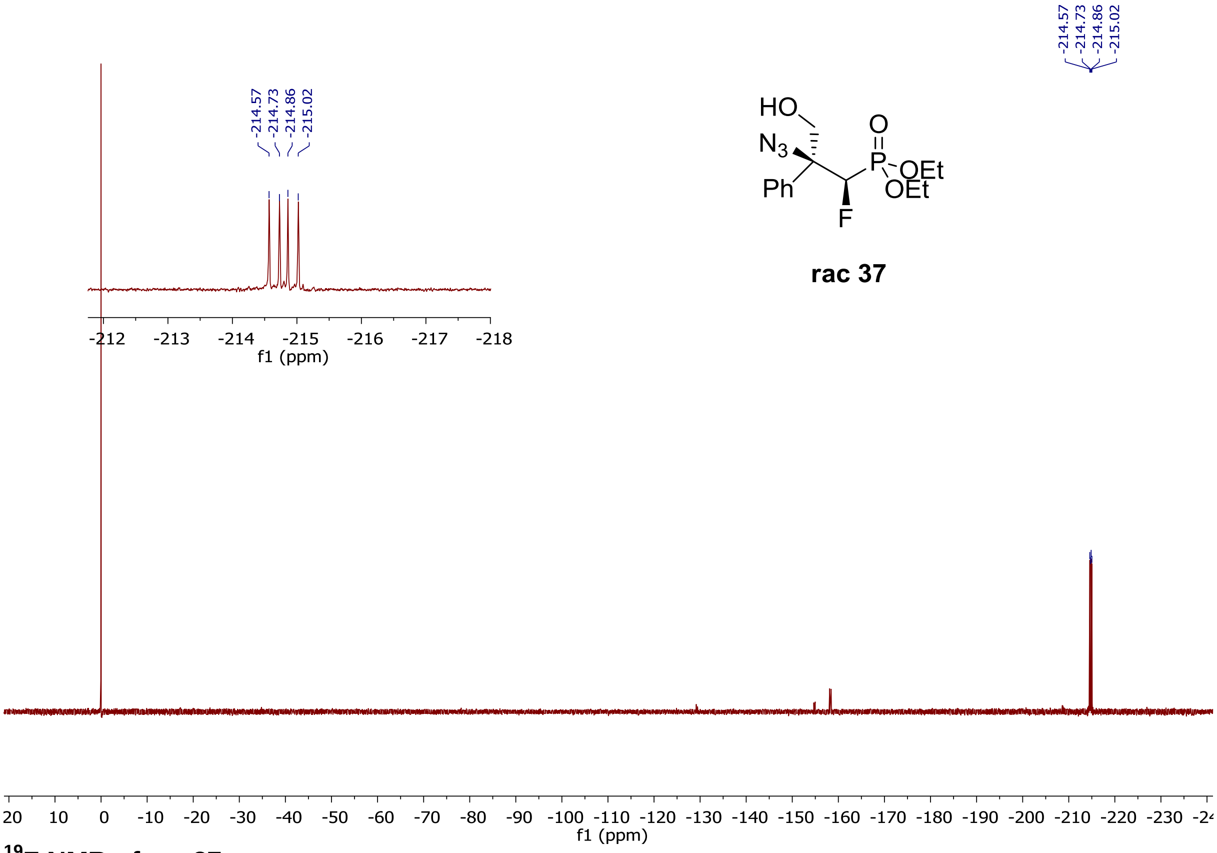

^{19}F NMR of rac **37**

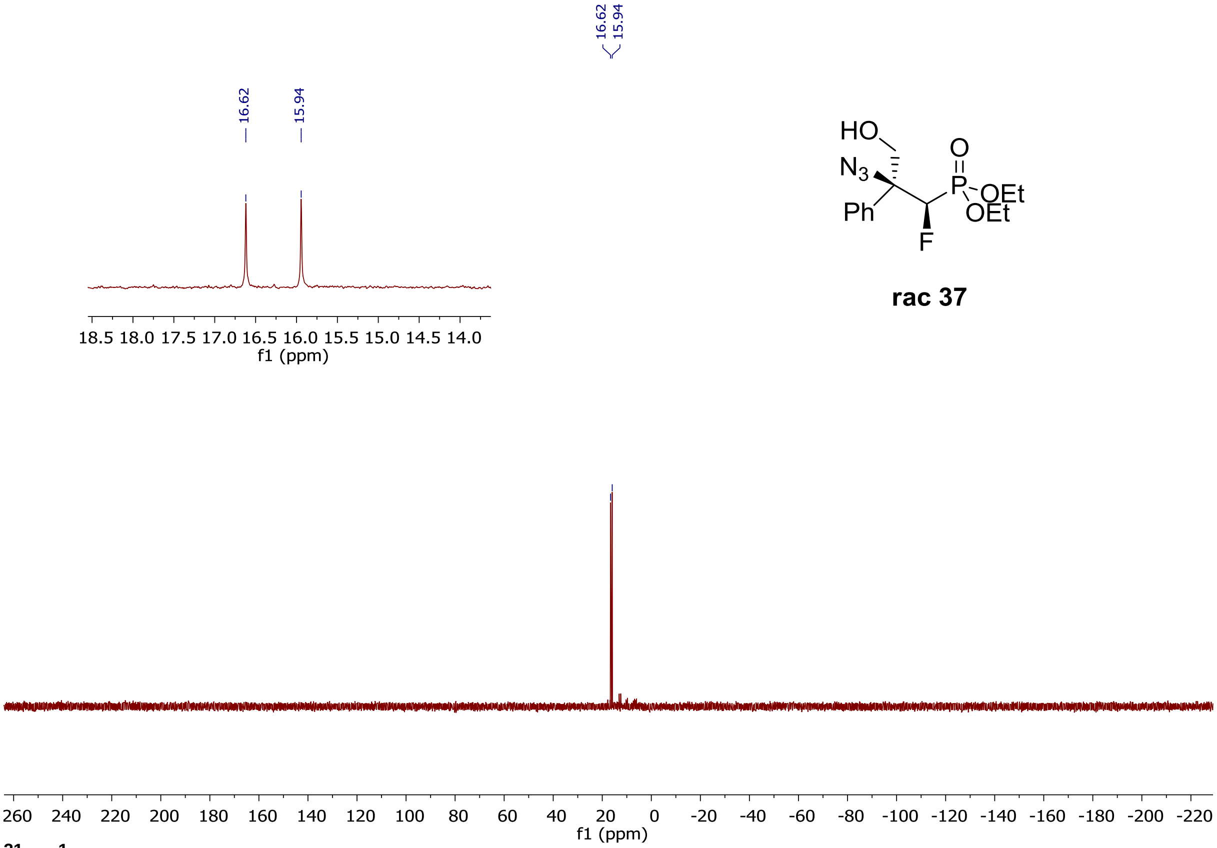

^{31}P{^{1}H} NMR of rac **37**

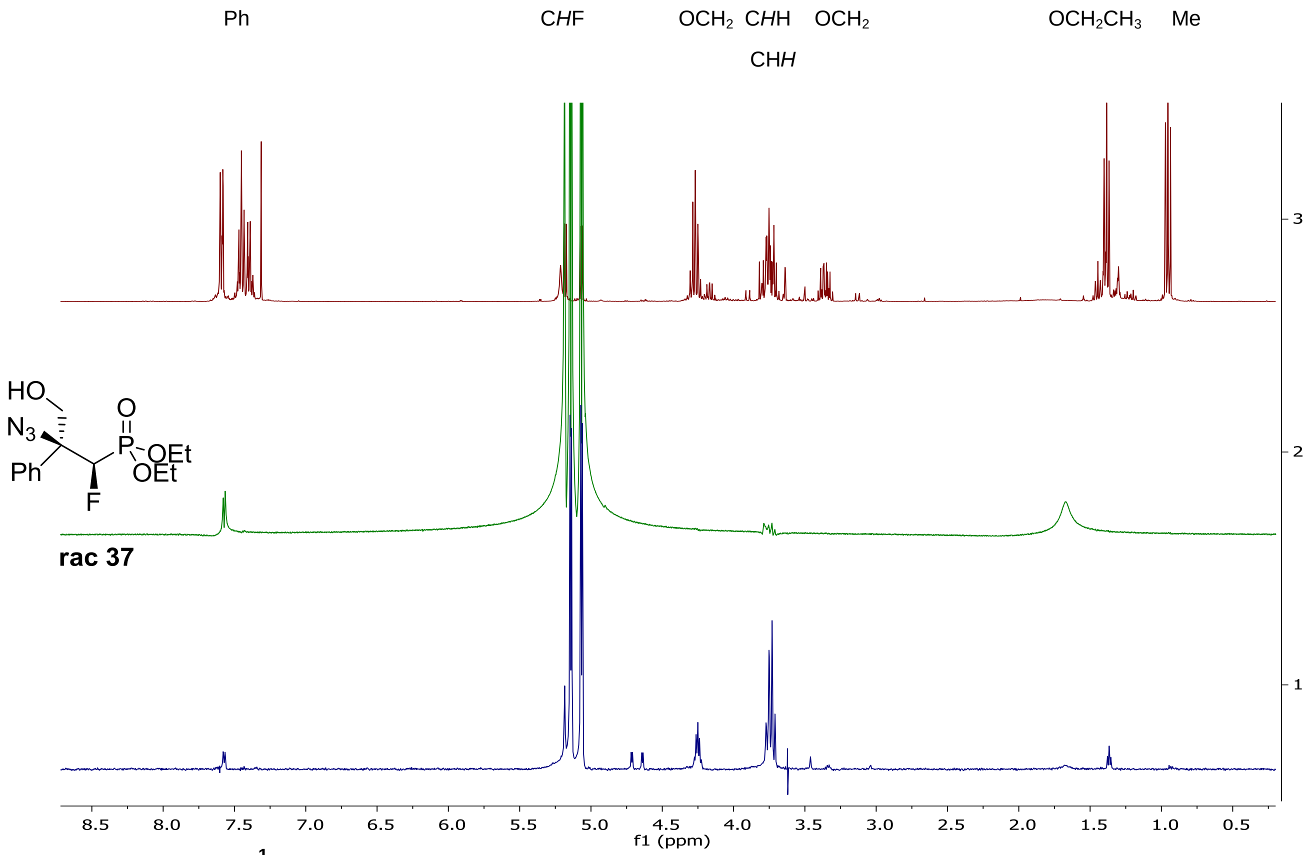

Stacked plots of ^{1}**H NMR** (top), **1D NOESY** (irradiated CHF, center) and **1D H-F HOESY** (bottom) of rac **37**

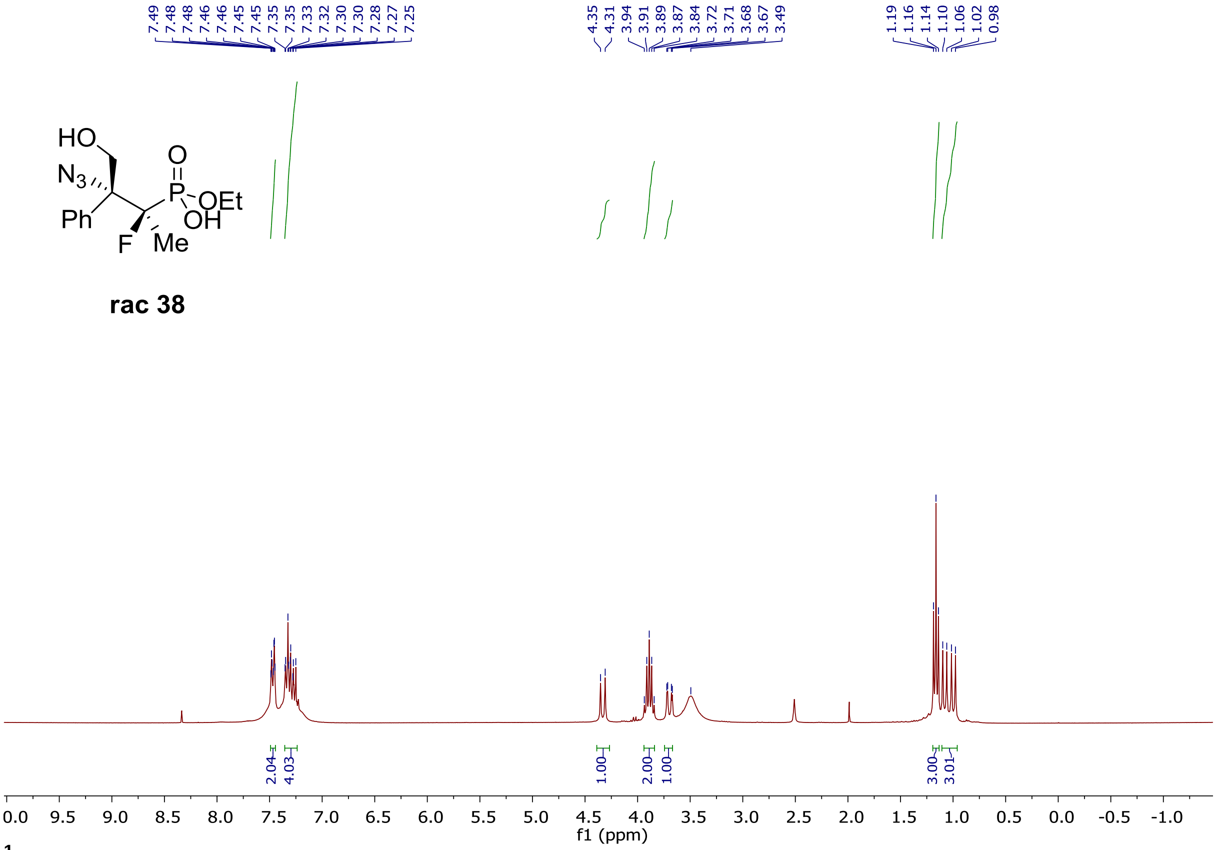

^{1}**H NMR of** rac **38**

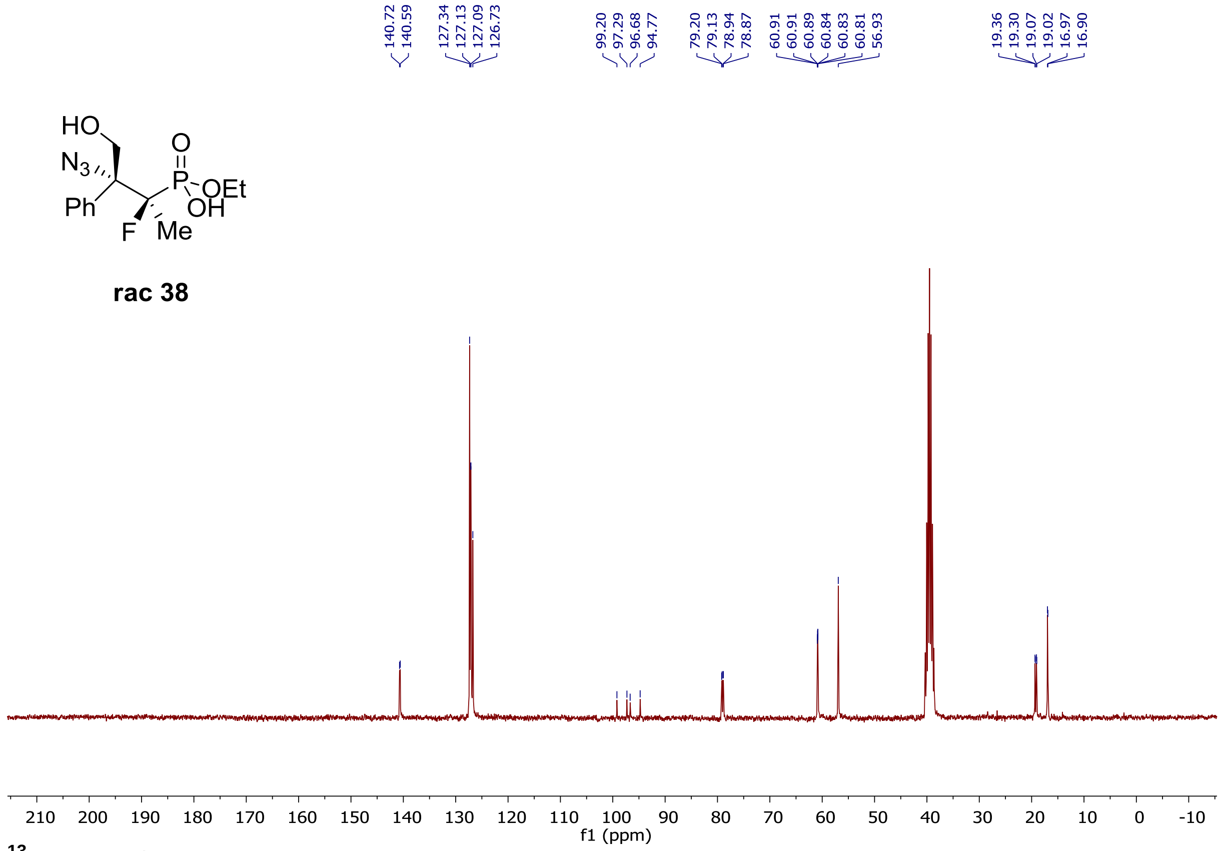

^{13}C NMR of rac **38**

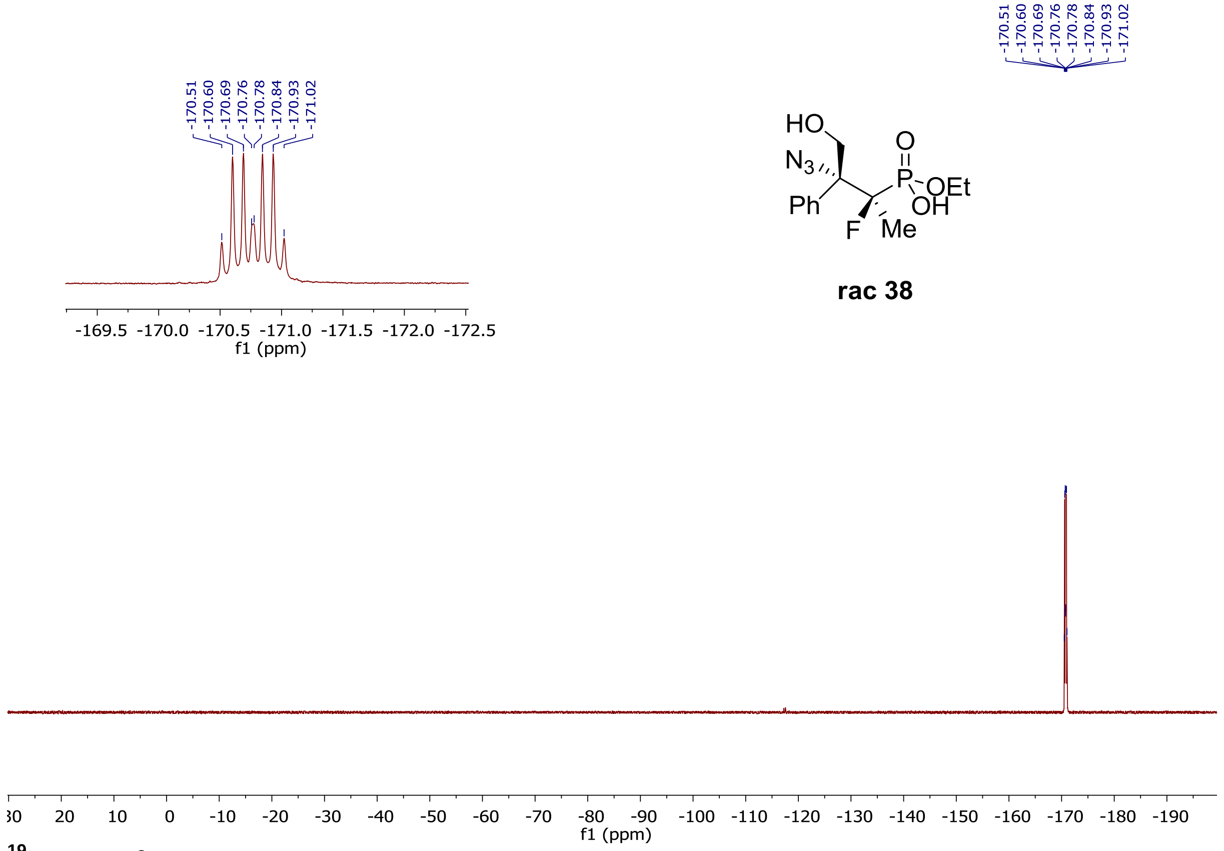

^{19}F NMR of rac **38**

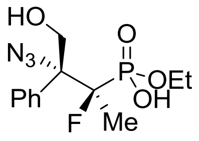

rac 38

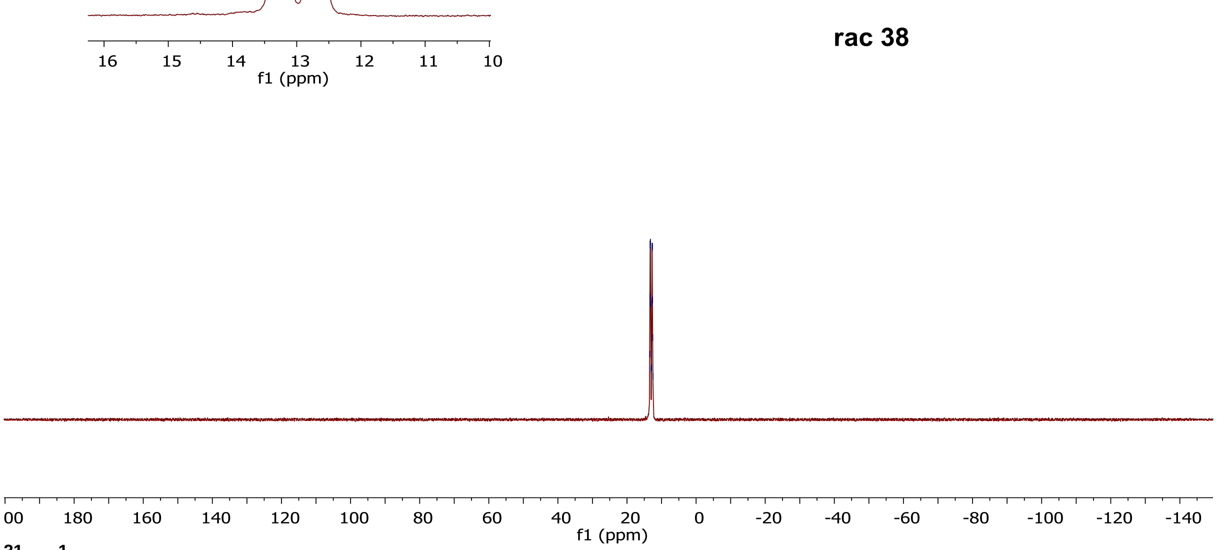

^{31}P{/^{1}H} NMR of rac **38**

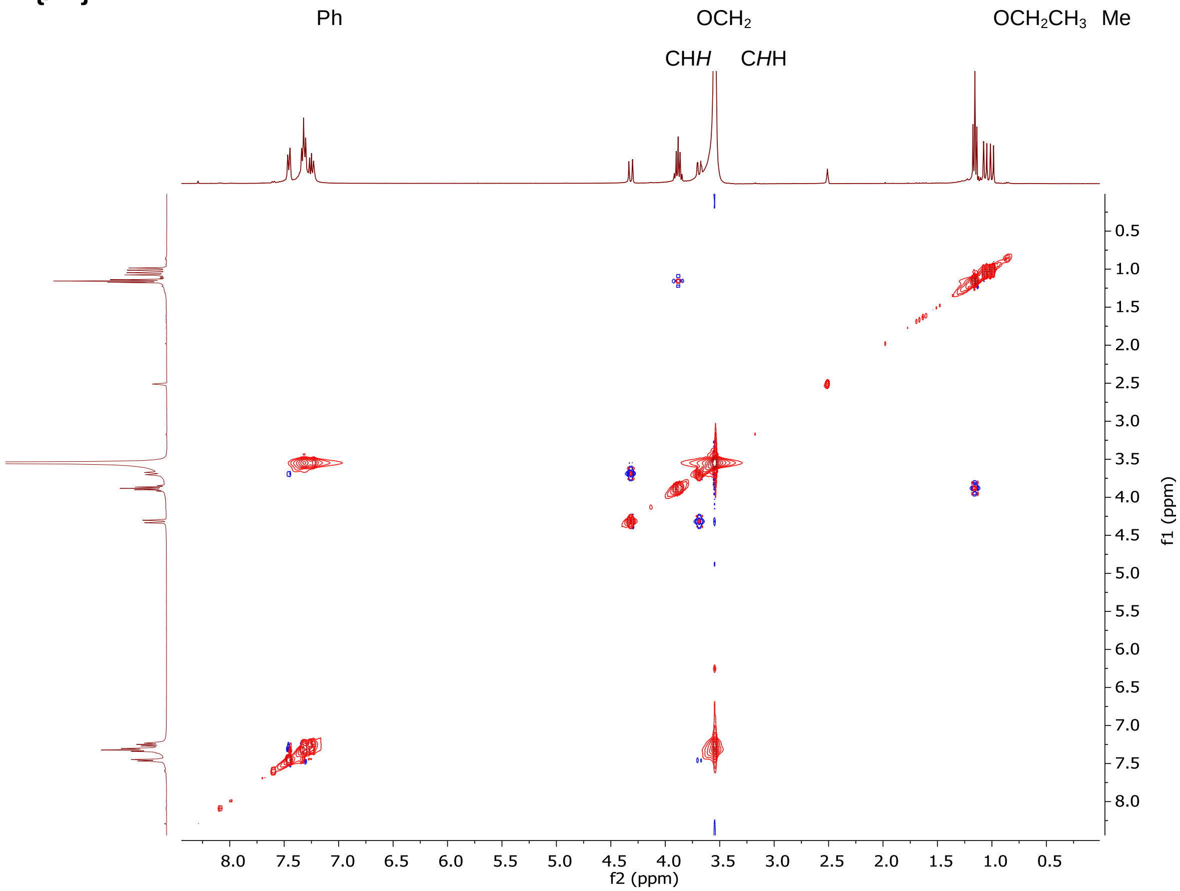

2D NOESY of rac **38**

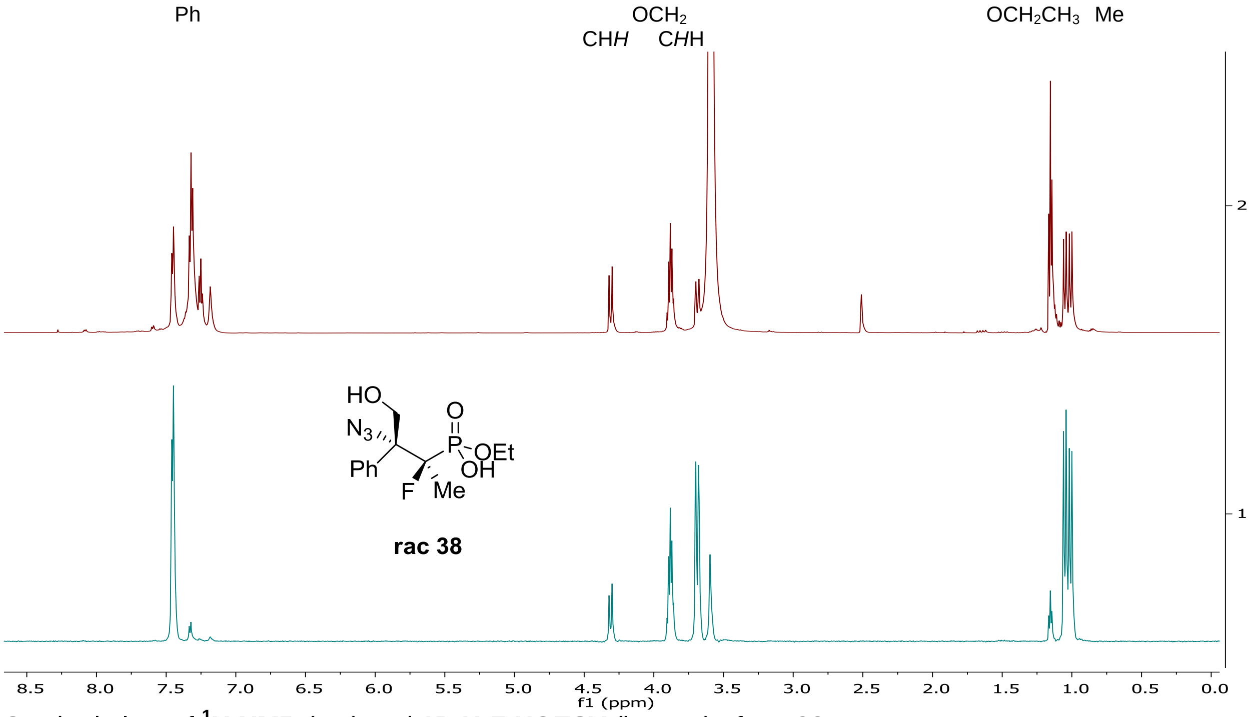

Stacked plots of ^{1}**H NMR** (top) and **1D H-F HOESY** (bottom) of rac **38**

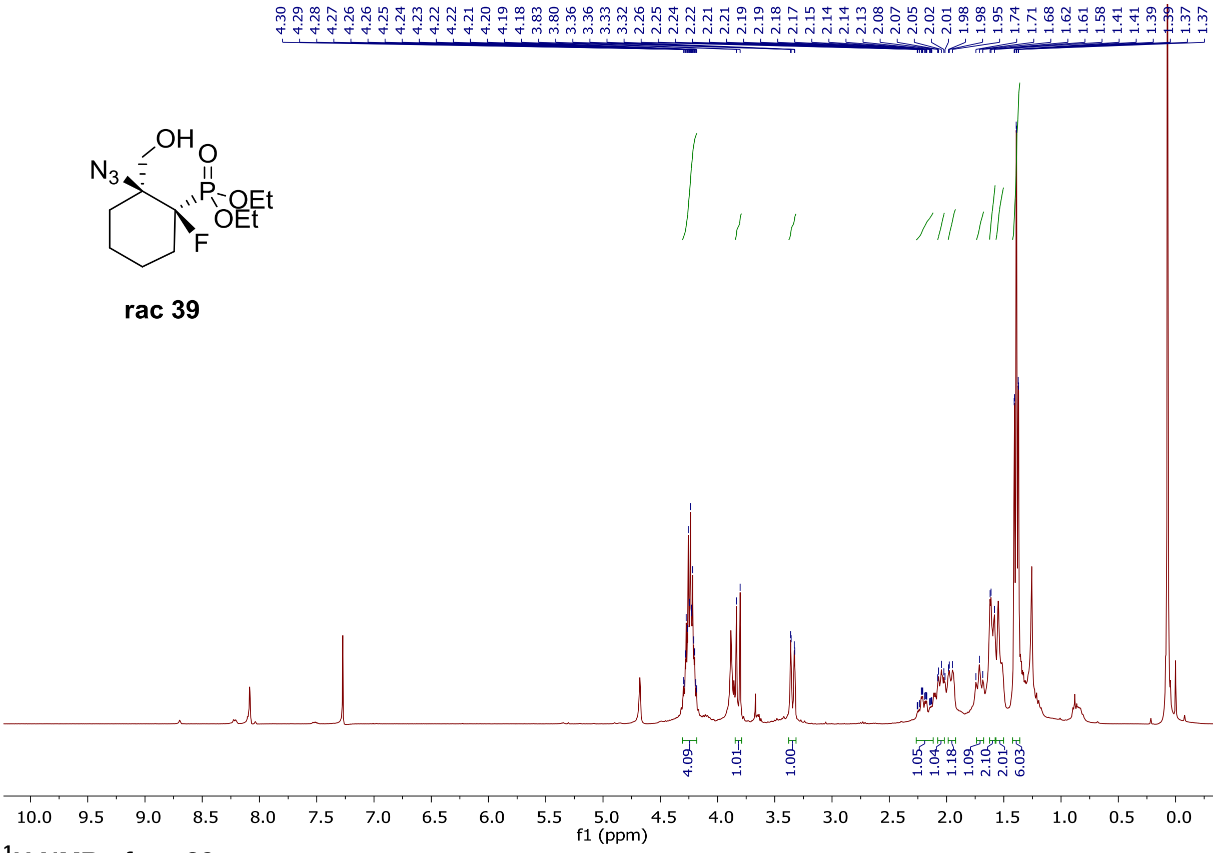

^{1}**H NMR of** rac **39**

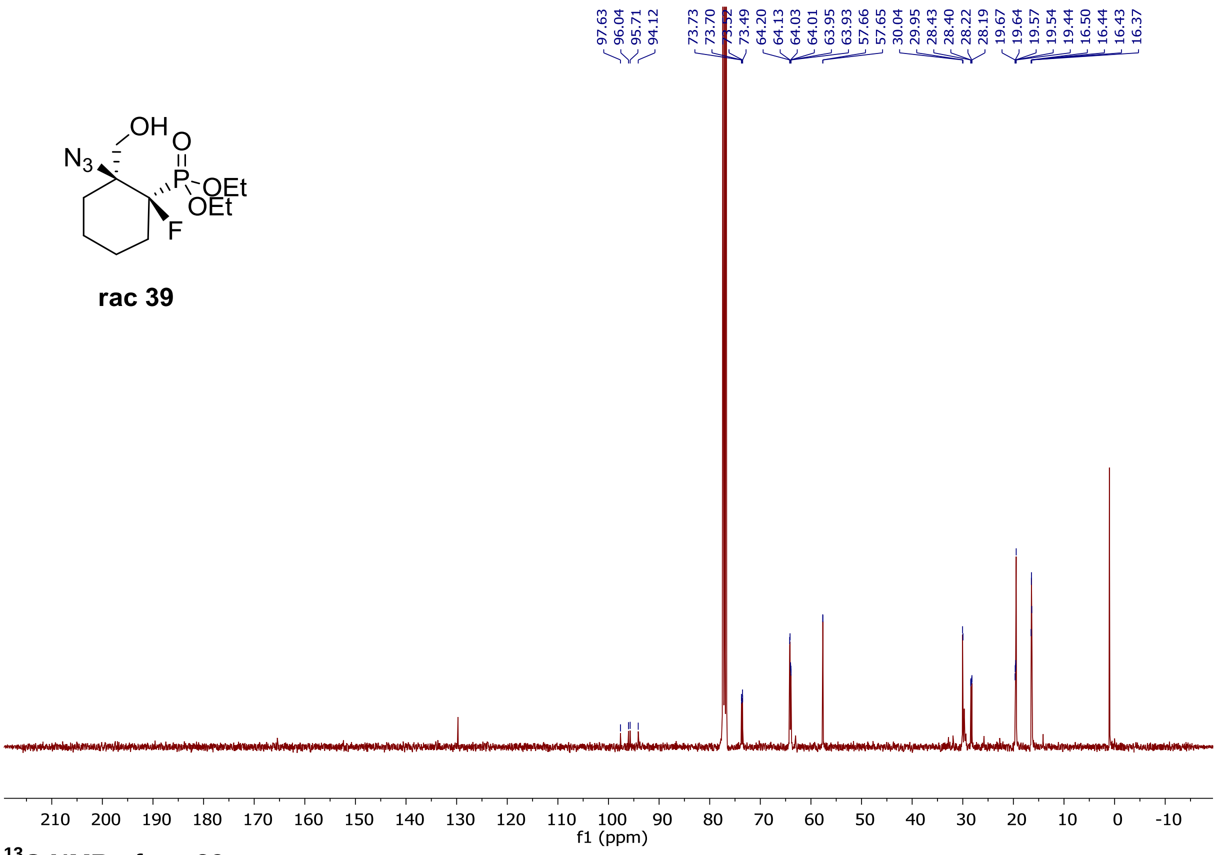

rac 39

^{13}C NMR of rac **39**

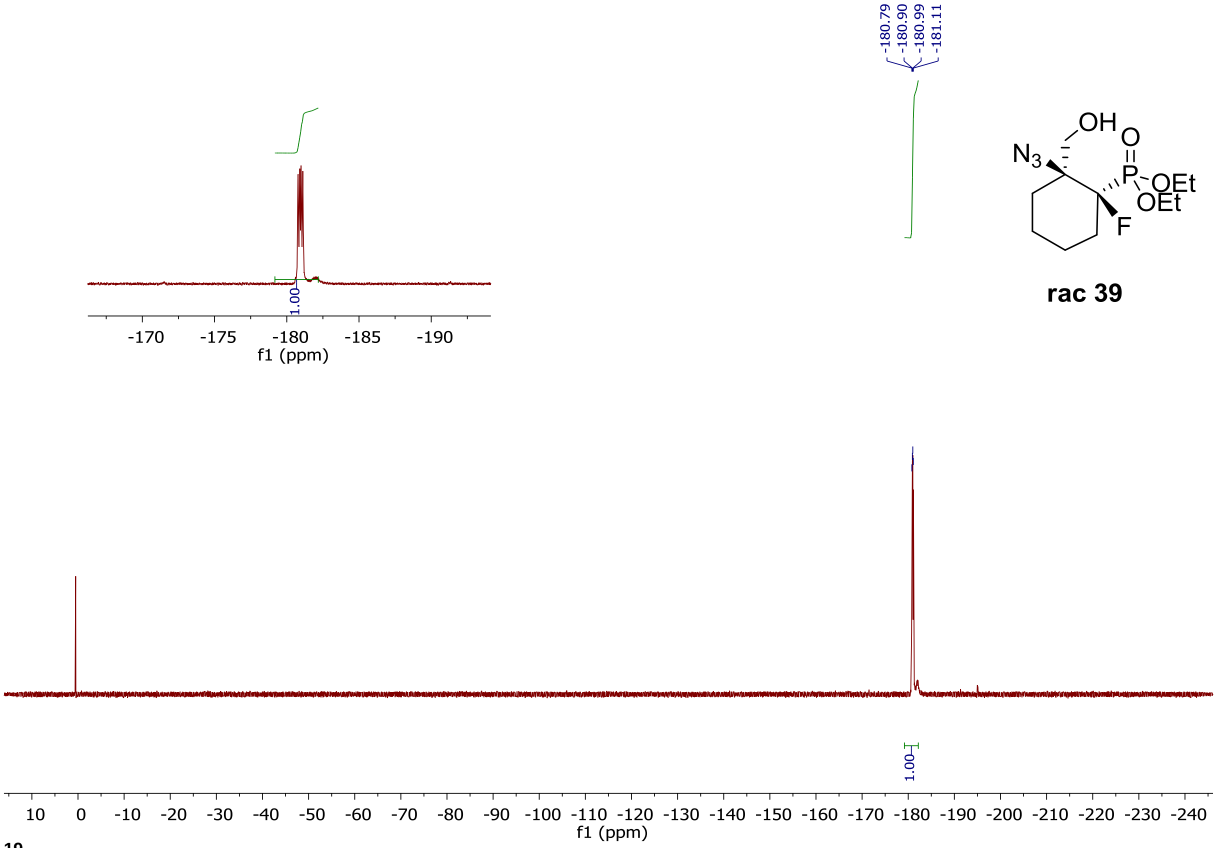

rac 39

^{19}F NMR of rac **39**

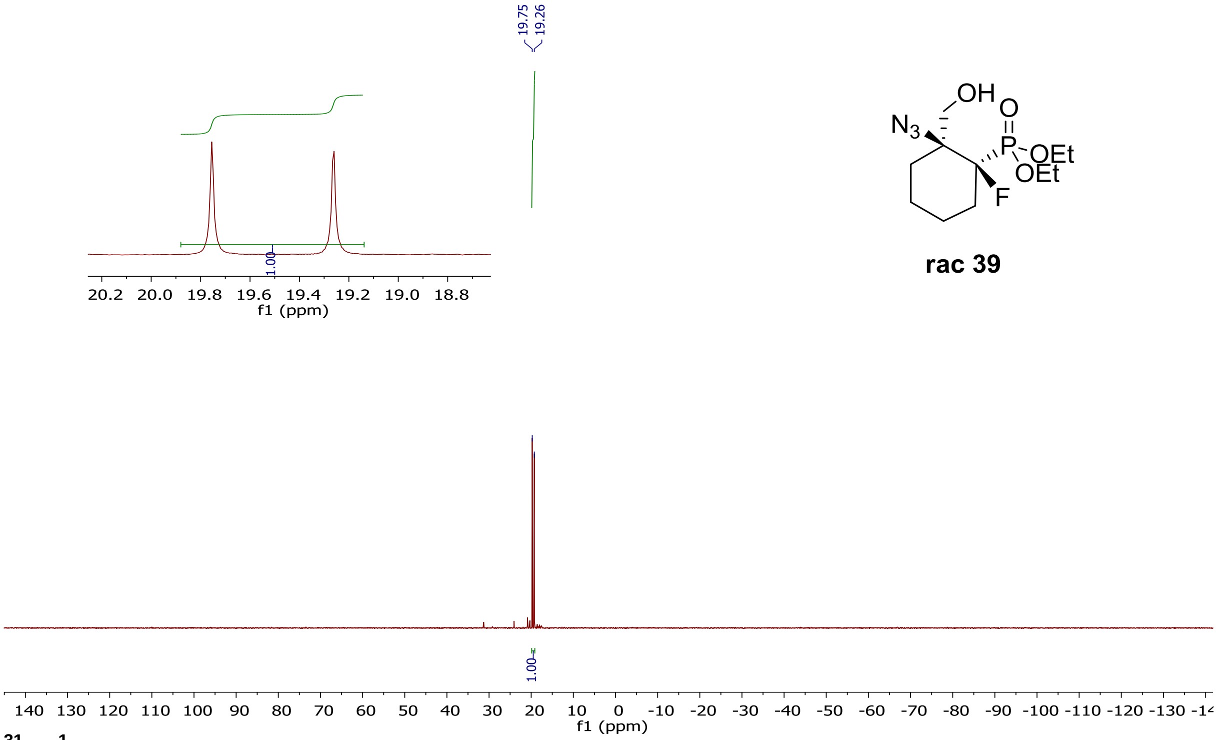

rac 39

^{31}P{/^{1}H} NMR of rac **39**

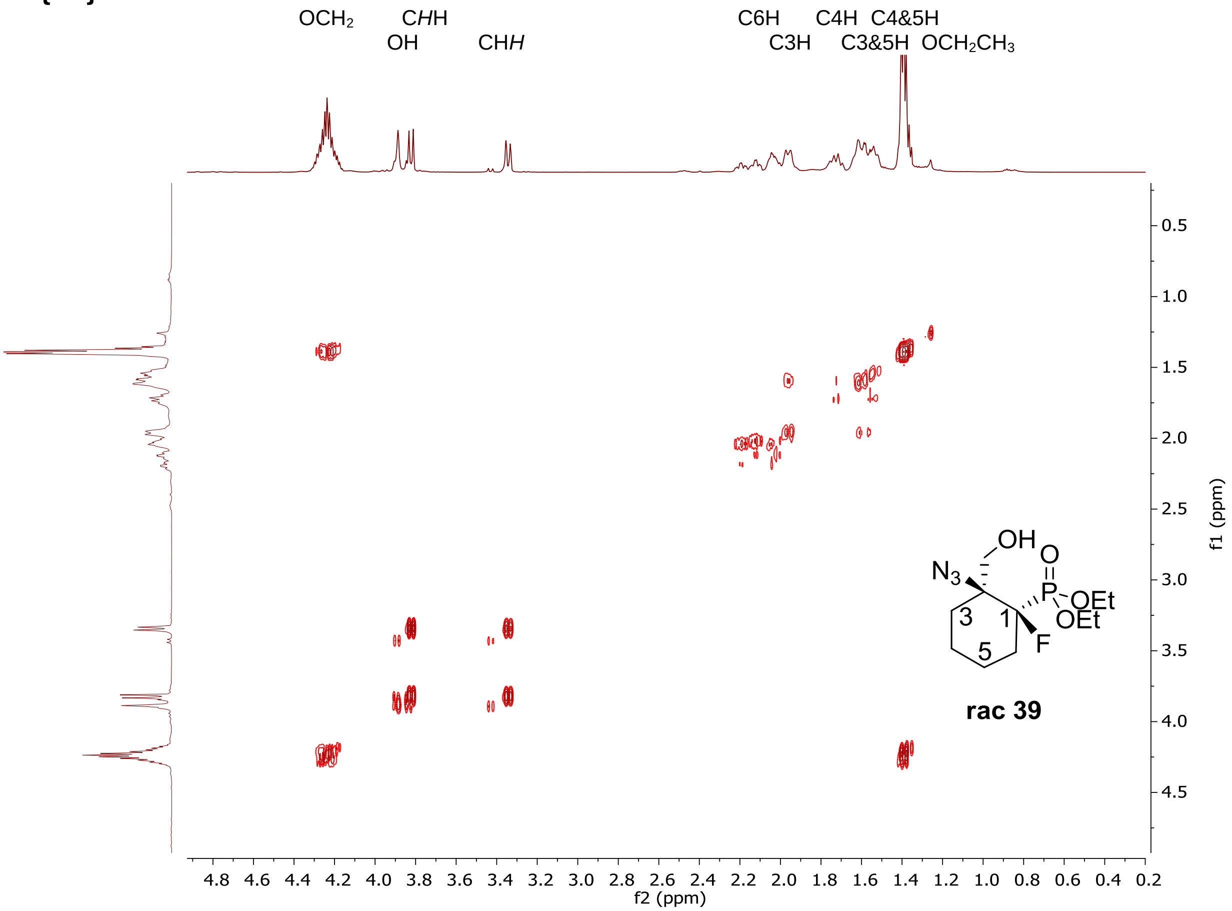

COSY of rac **39**

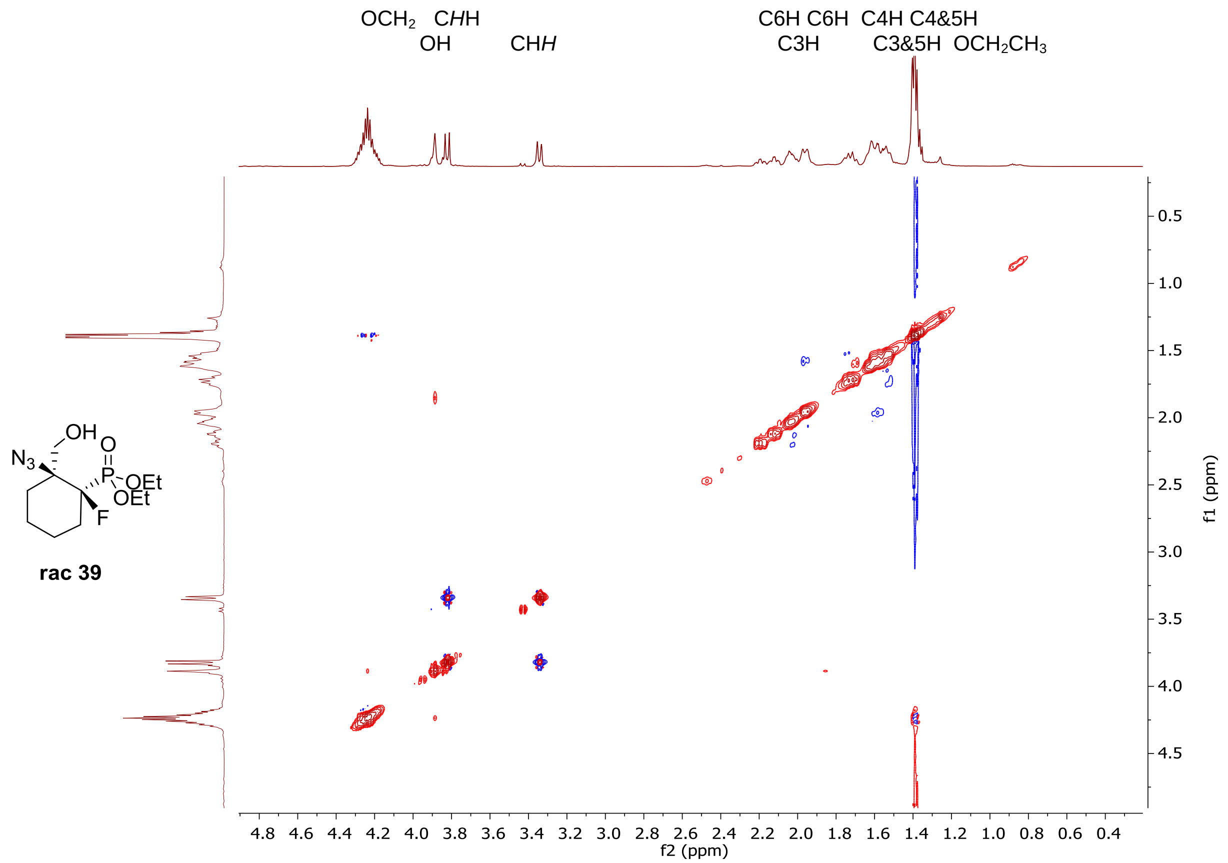

2D NOESY of rac **39**

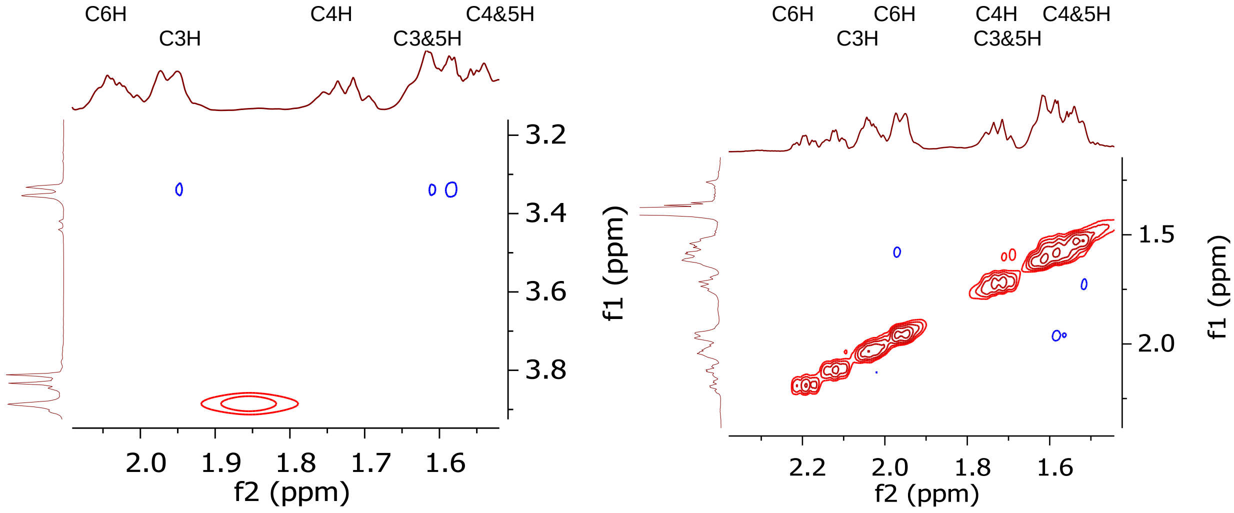

Diagnostic fragments of **2D NOESY of** rac **39**

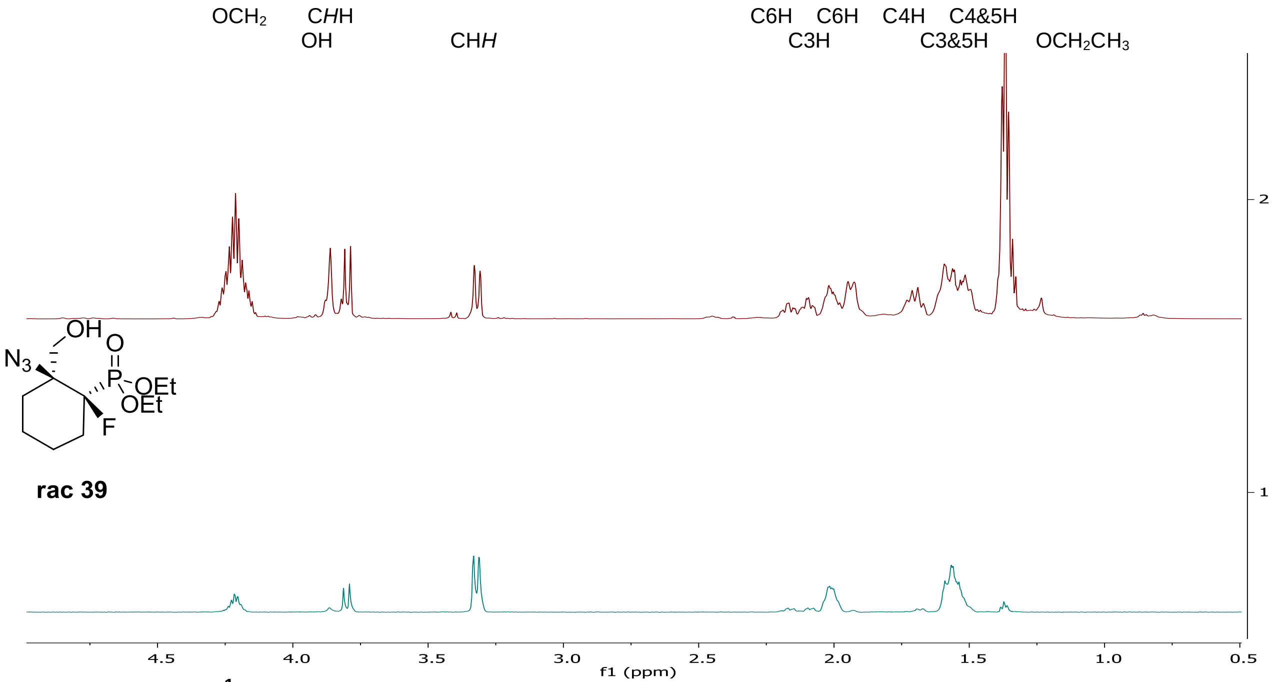

Stacked plots of ^{1}H NMR (top) and **1D H-F HOESY** (bottom) of rac **39**

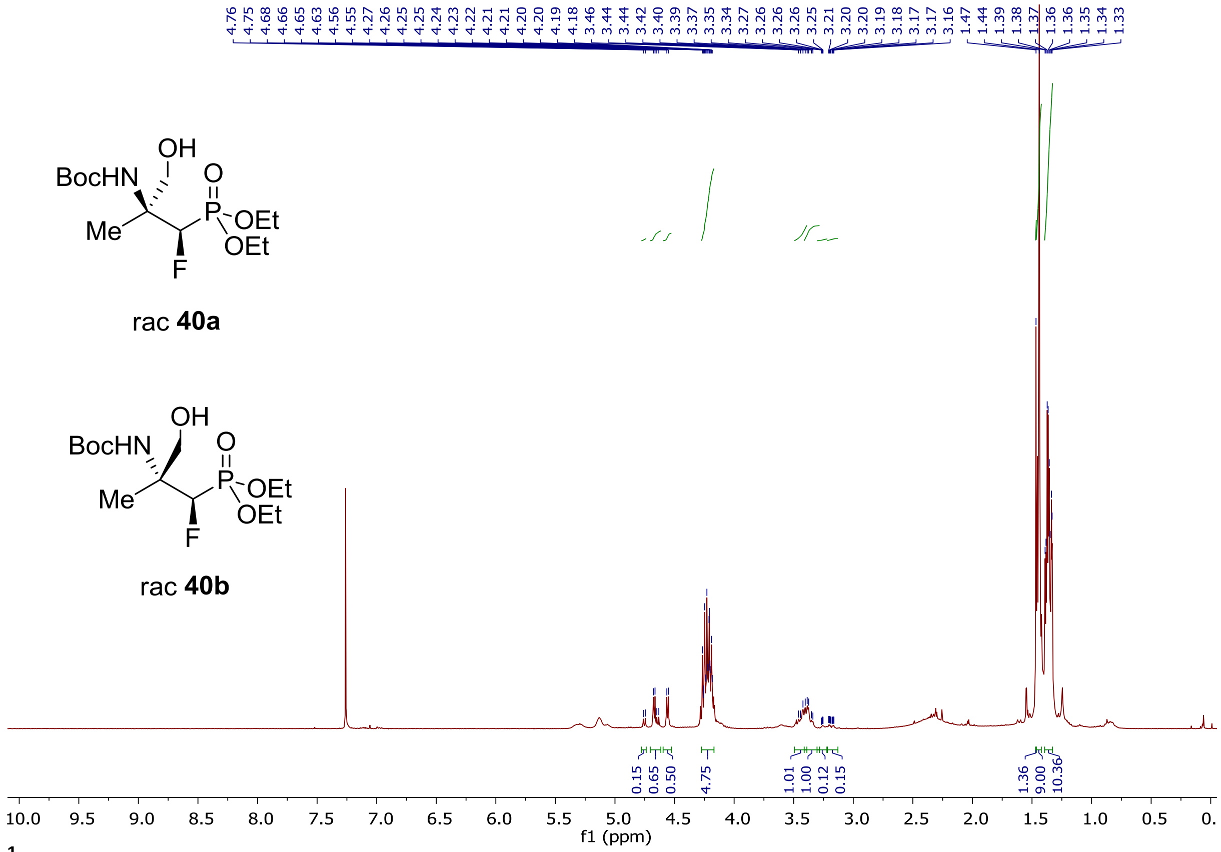

^{1}**H NMR of** rac **40a,b** (6.7:1, d.r.)

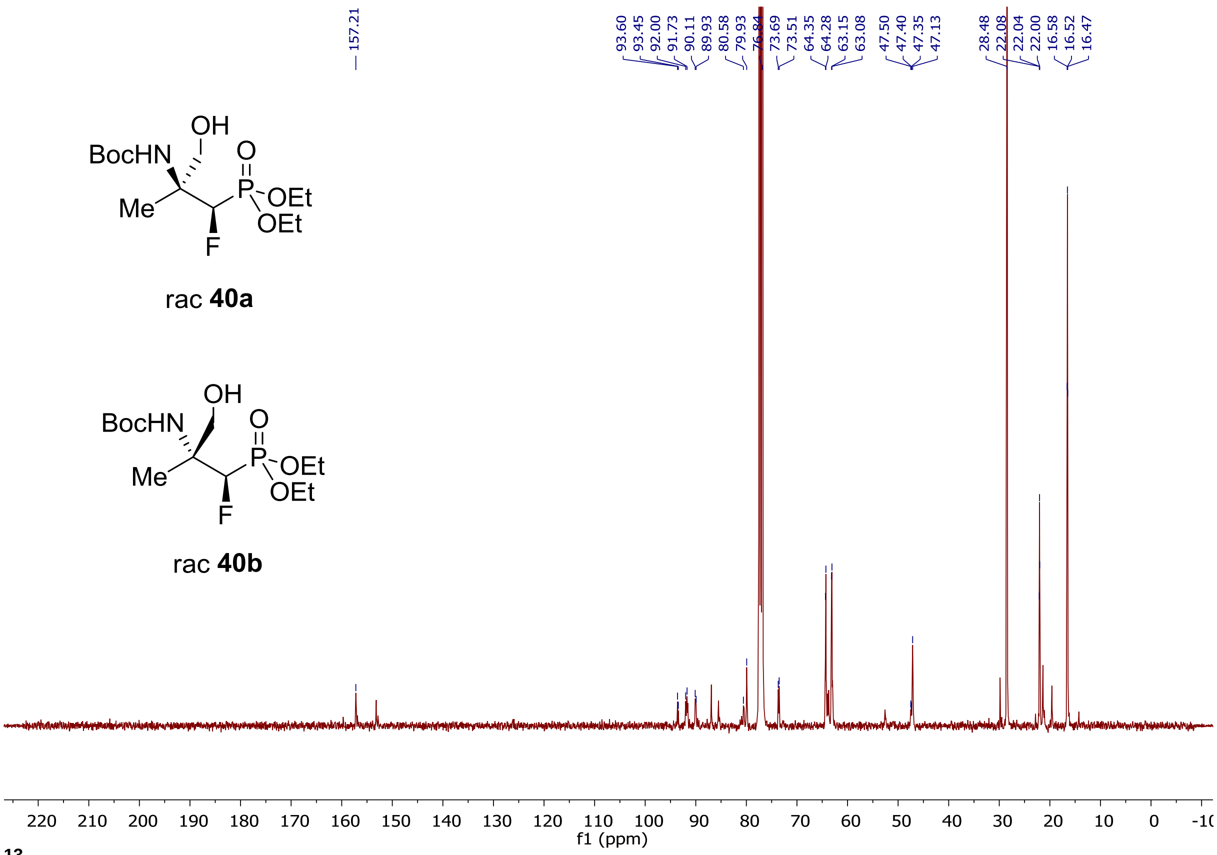

^{13}C NMR of rac **40a,b** (6.7:1, d.r.)

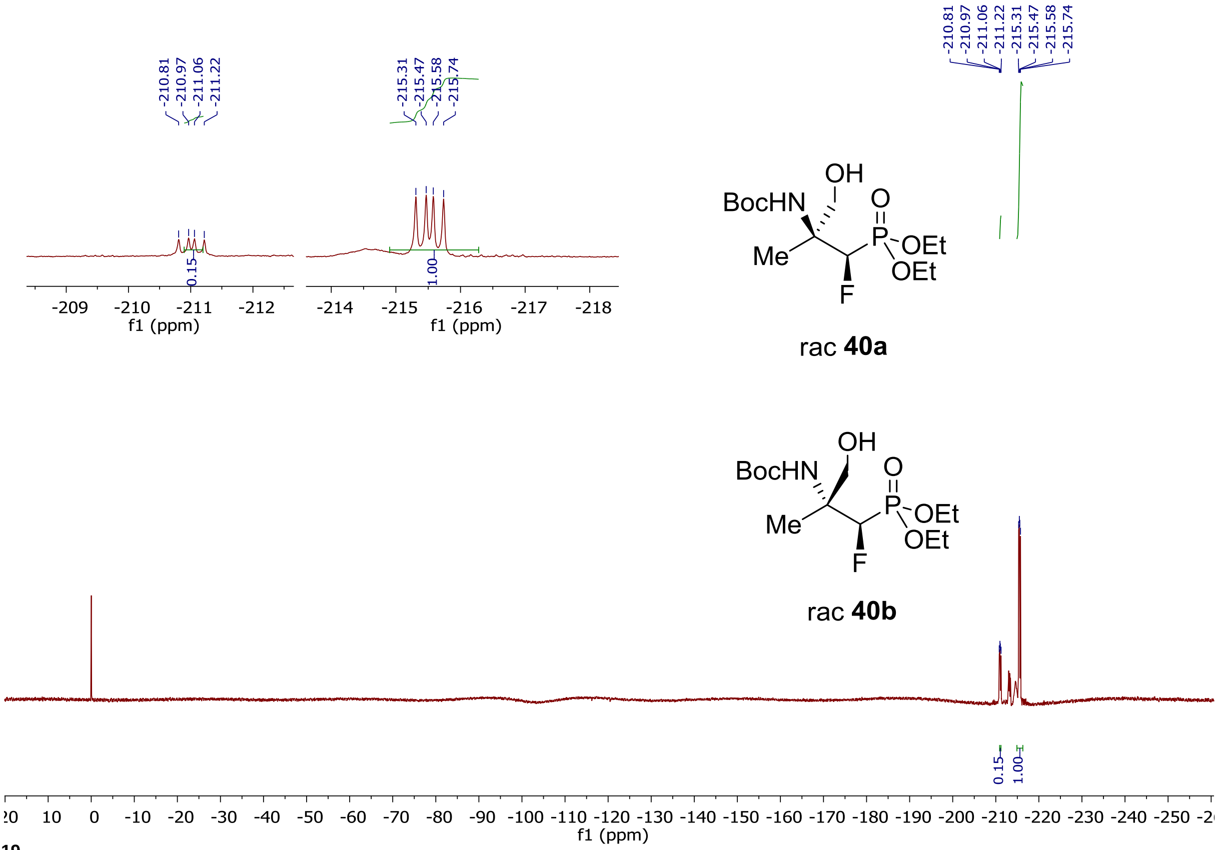

^{19}F NMR of rac **40a,b** (6.7:1, d.r.)

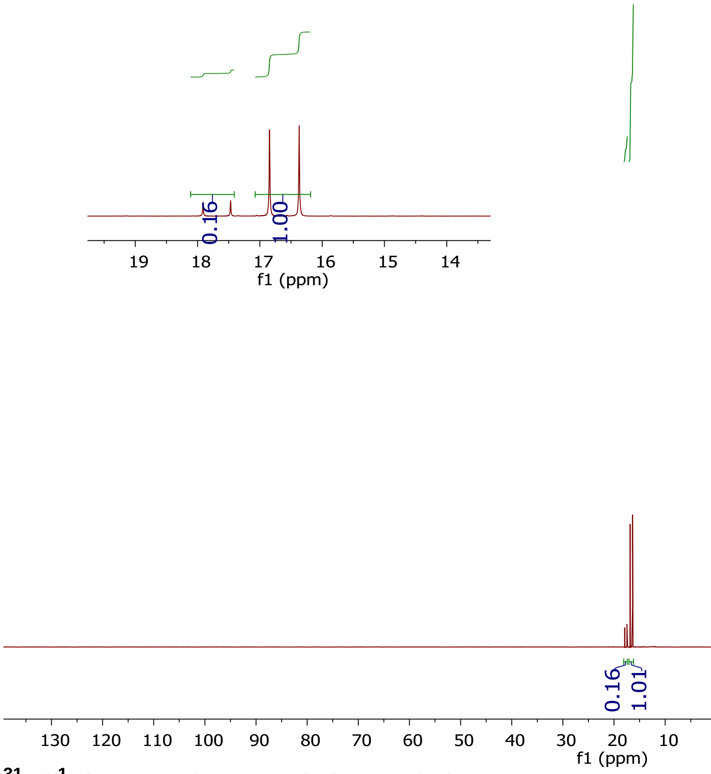

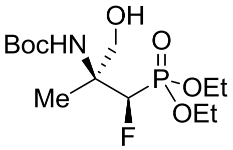

rac **40a**

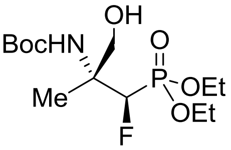

rac **40b**

^{31}P{/^{1}H} NMR of rac **40a,b** (6.7:1, d.r.)

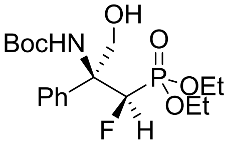

rac **41**

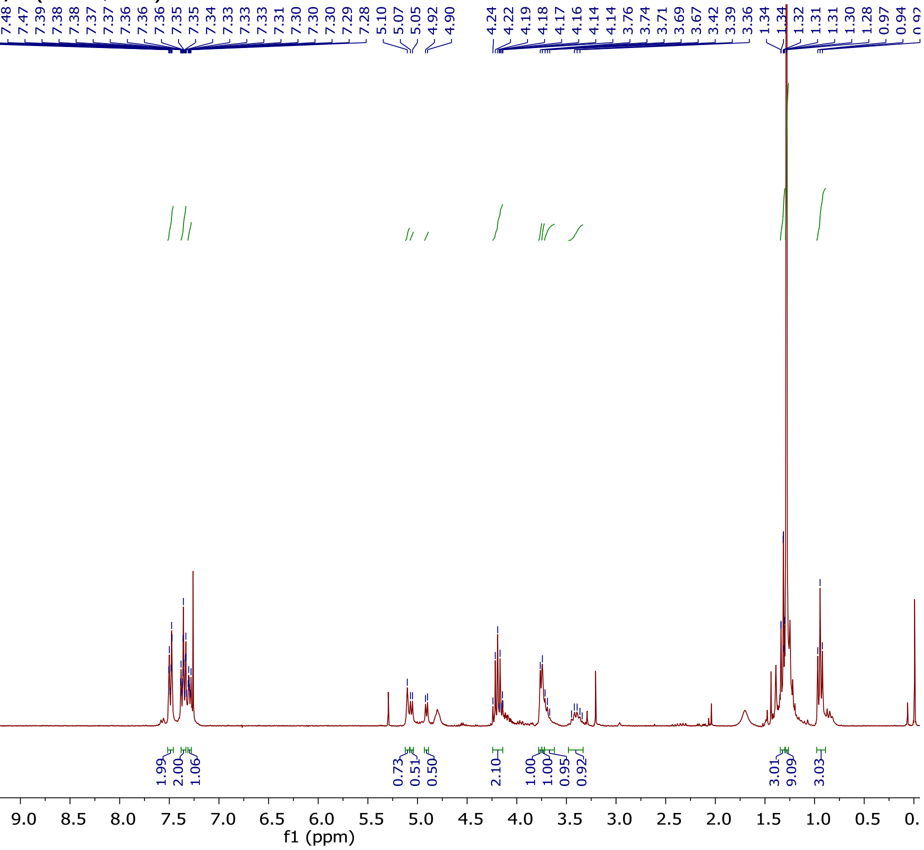

^{1}H NMR of rac **41**

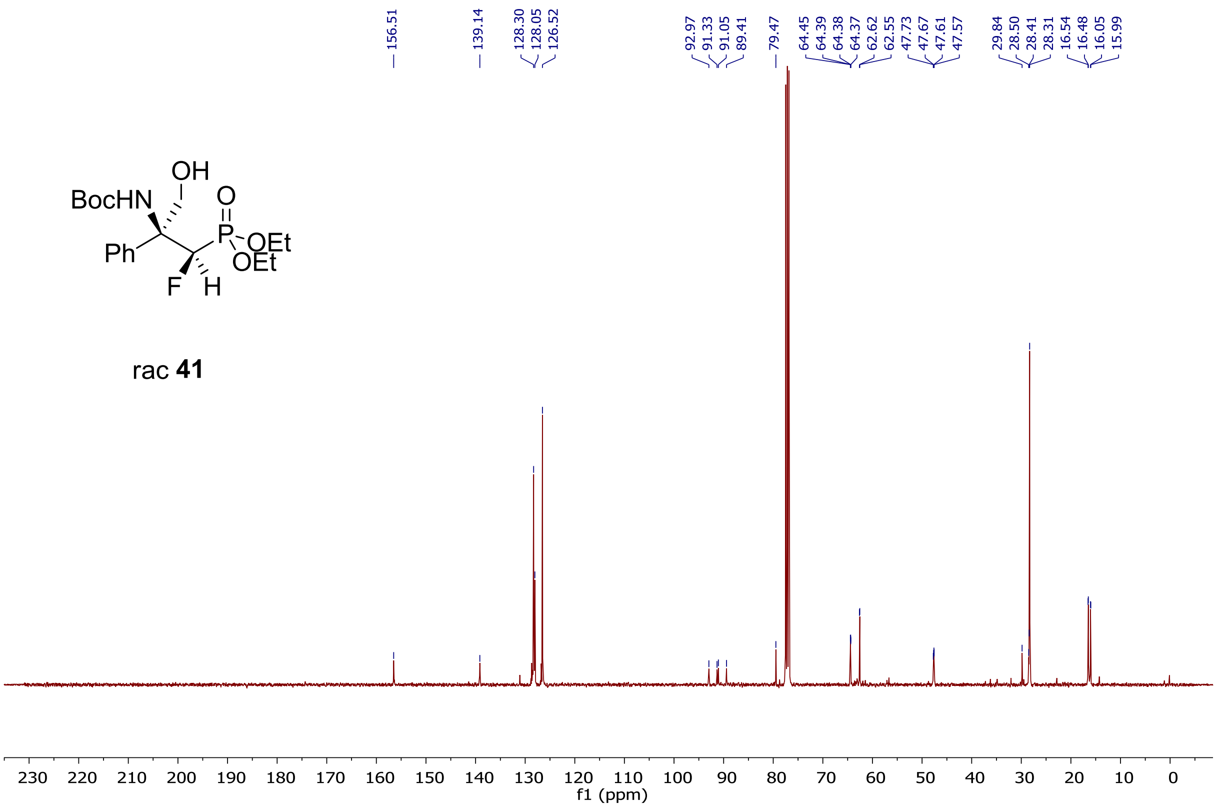

^{13}**C NMR of** rac **41** ($CDCl_3$)

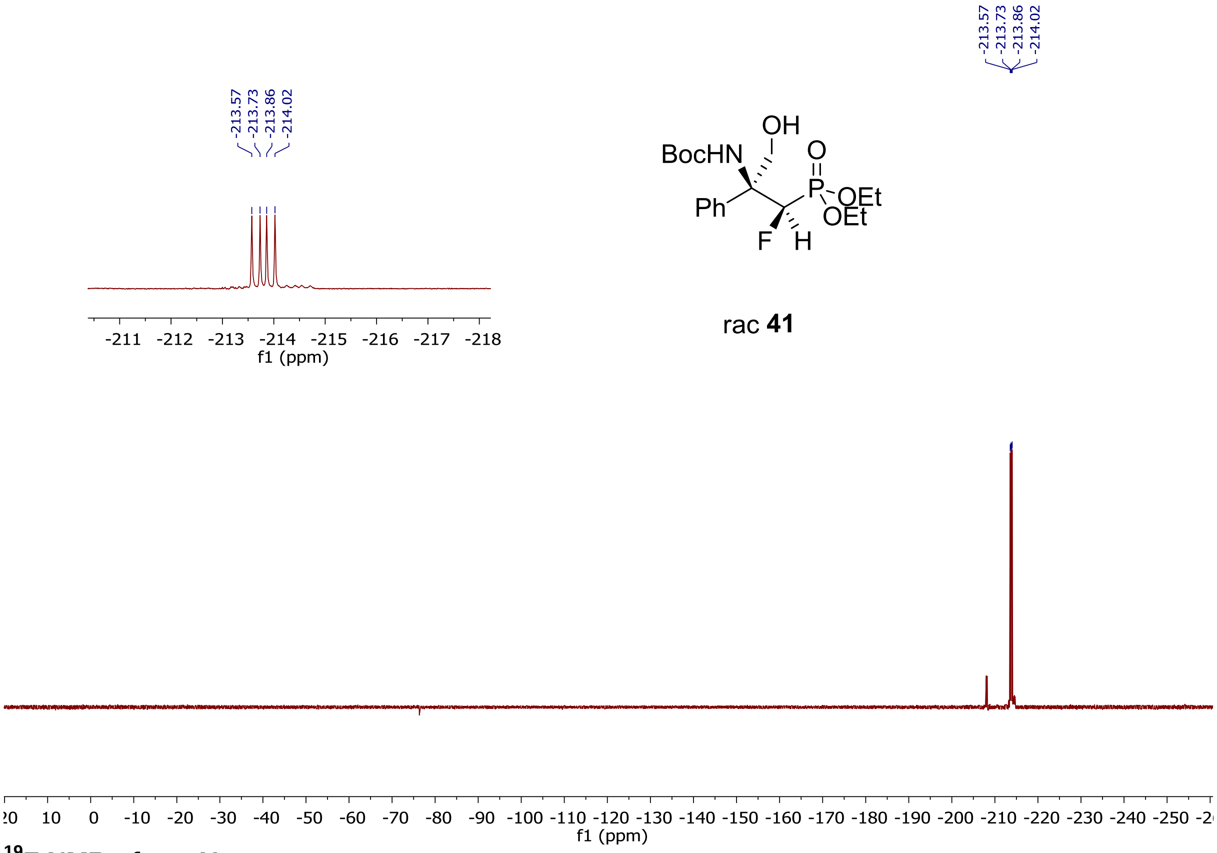

^{19}**F NMR of** rac **41**

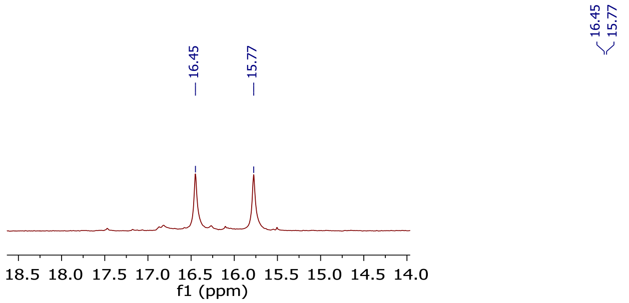

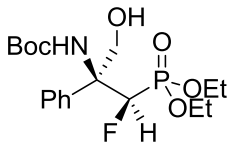

rac **41**

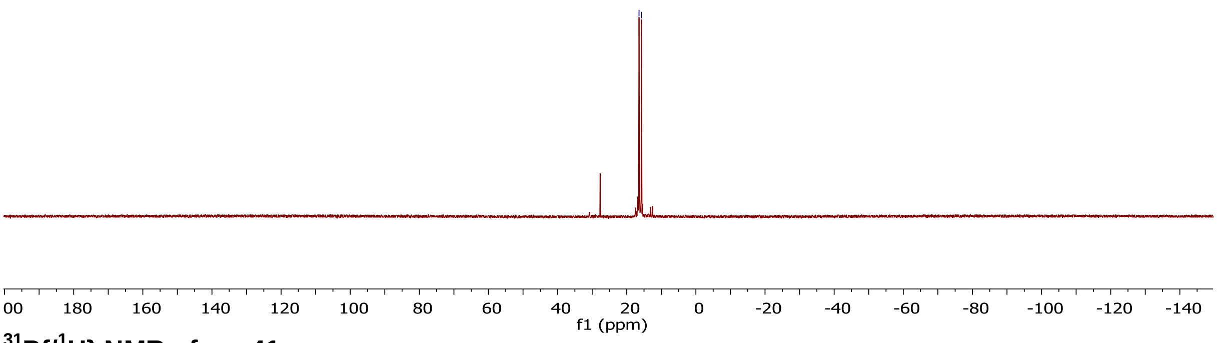

^{31}P{/^{1}H} NMR of rac **41**

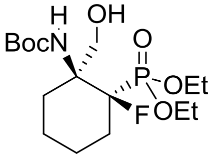

rac **42**

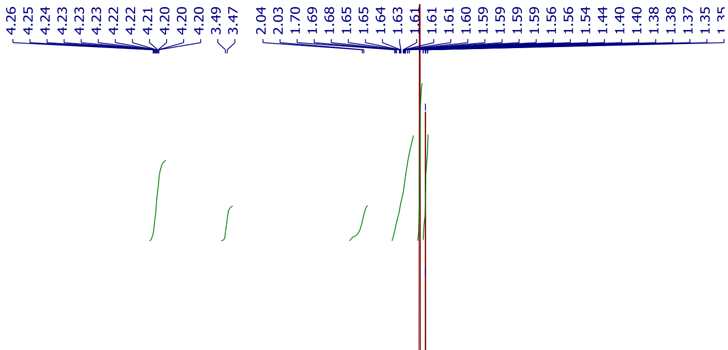

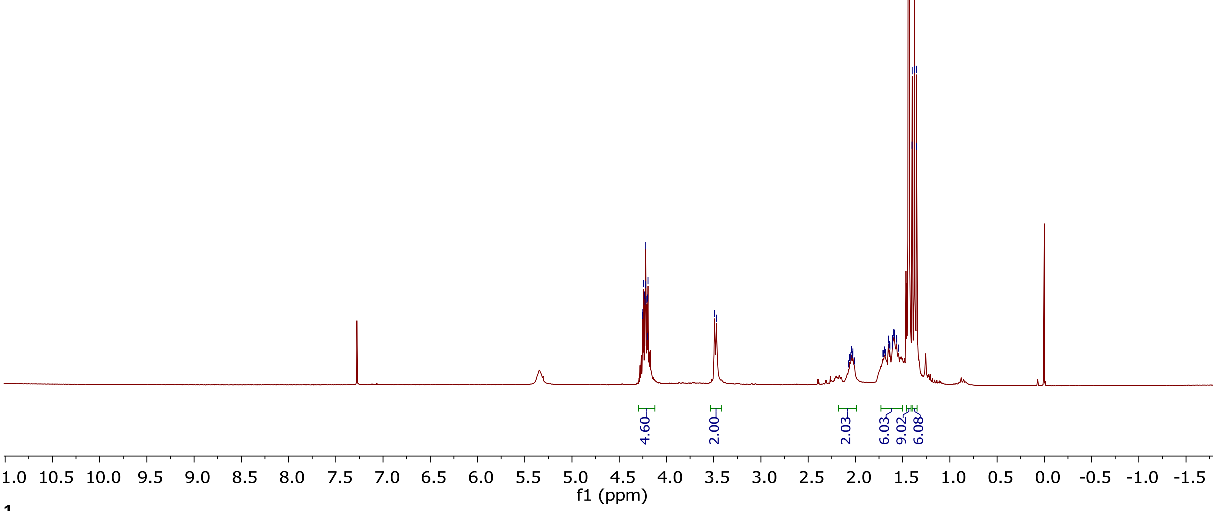

^{1}H NMR of rac **42**

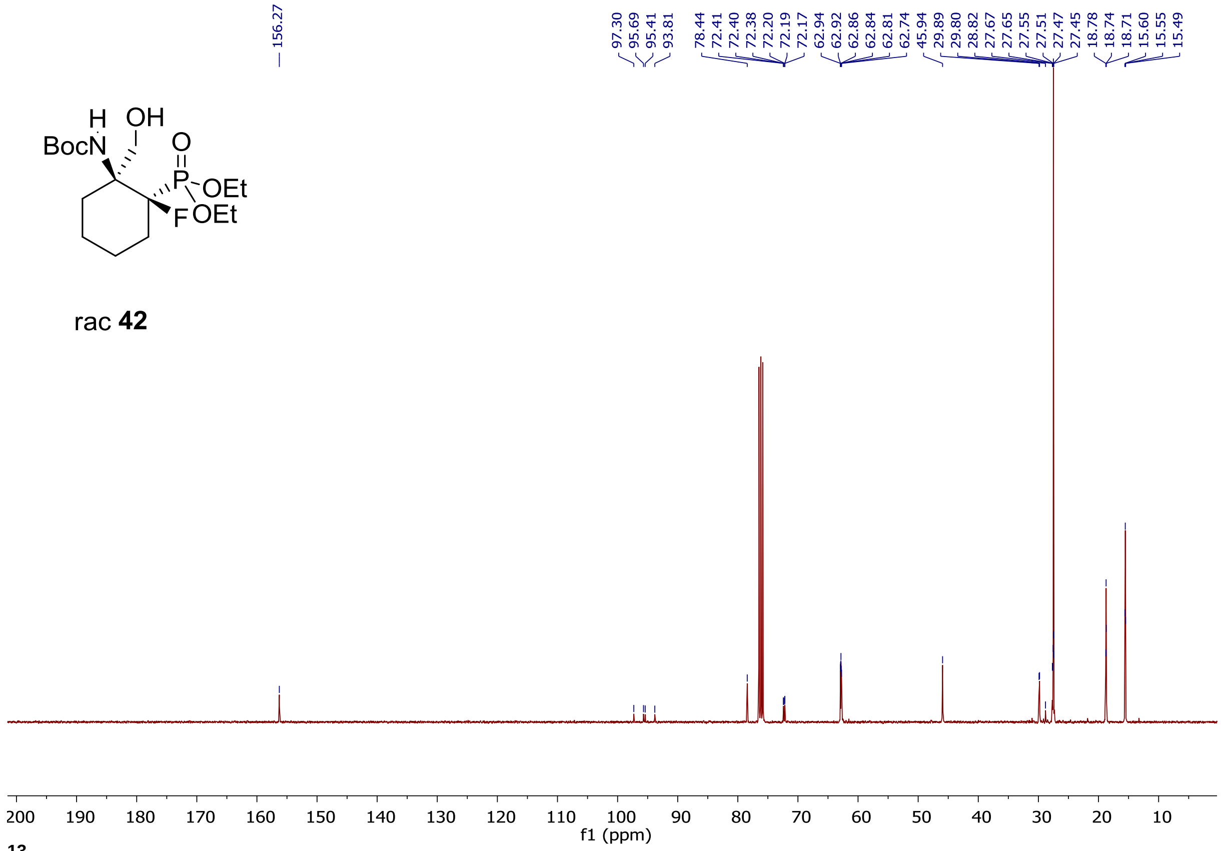

^{13}**C NMR of** rac **42**

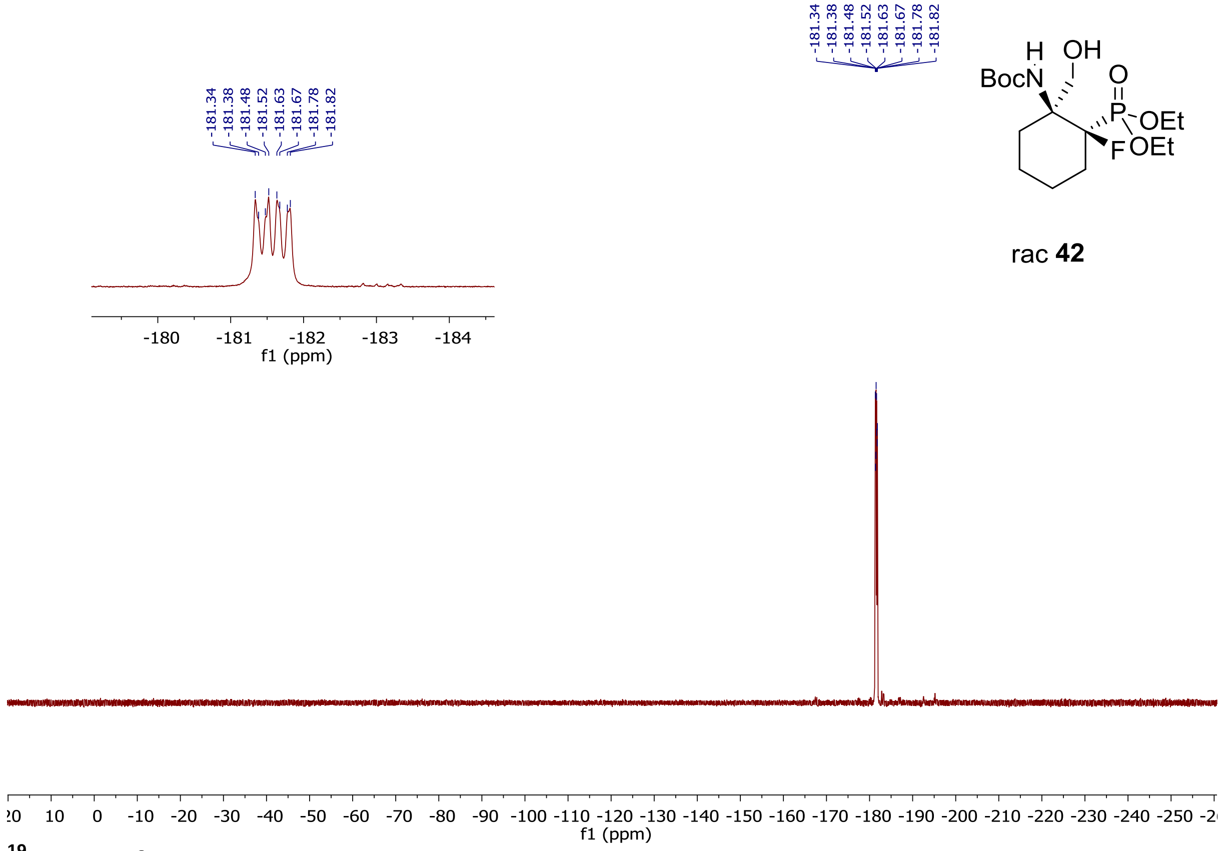

^{19}**F NMR of** rac **42**

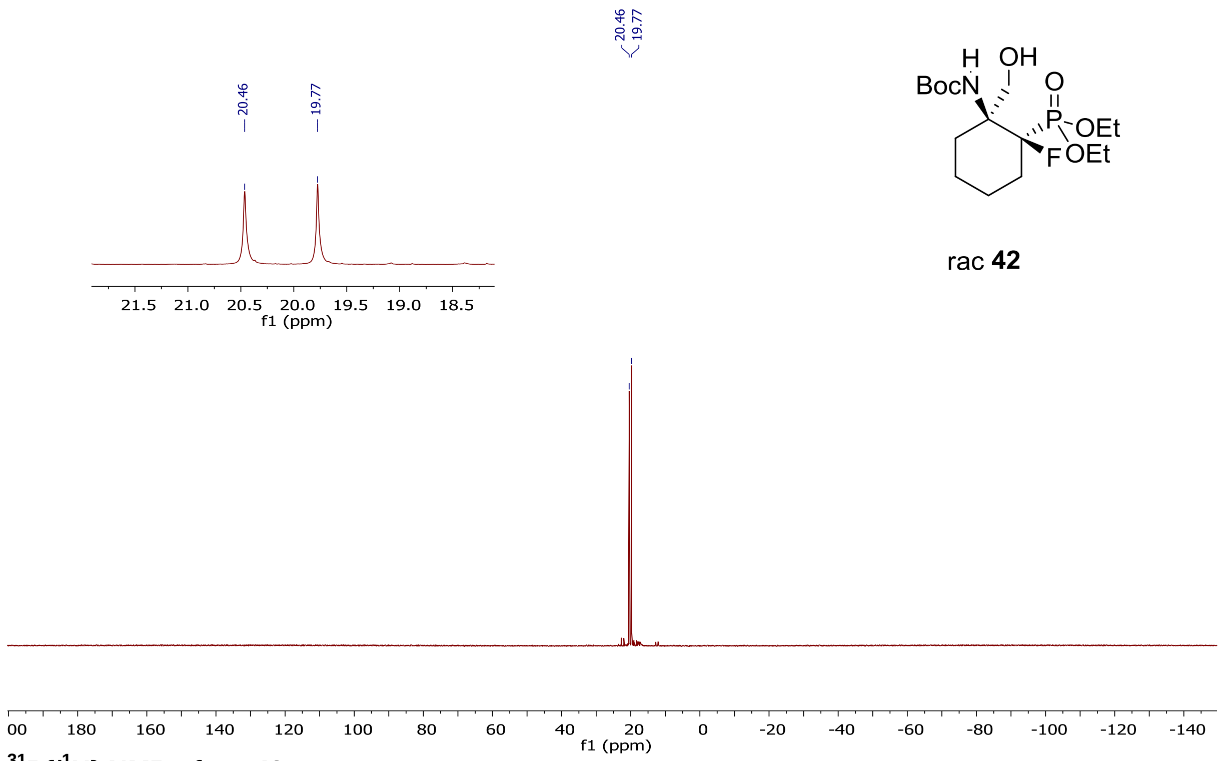

rac **42**

^{31}P{/^{1}H} NMR of rac **42**

rac **43**

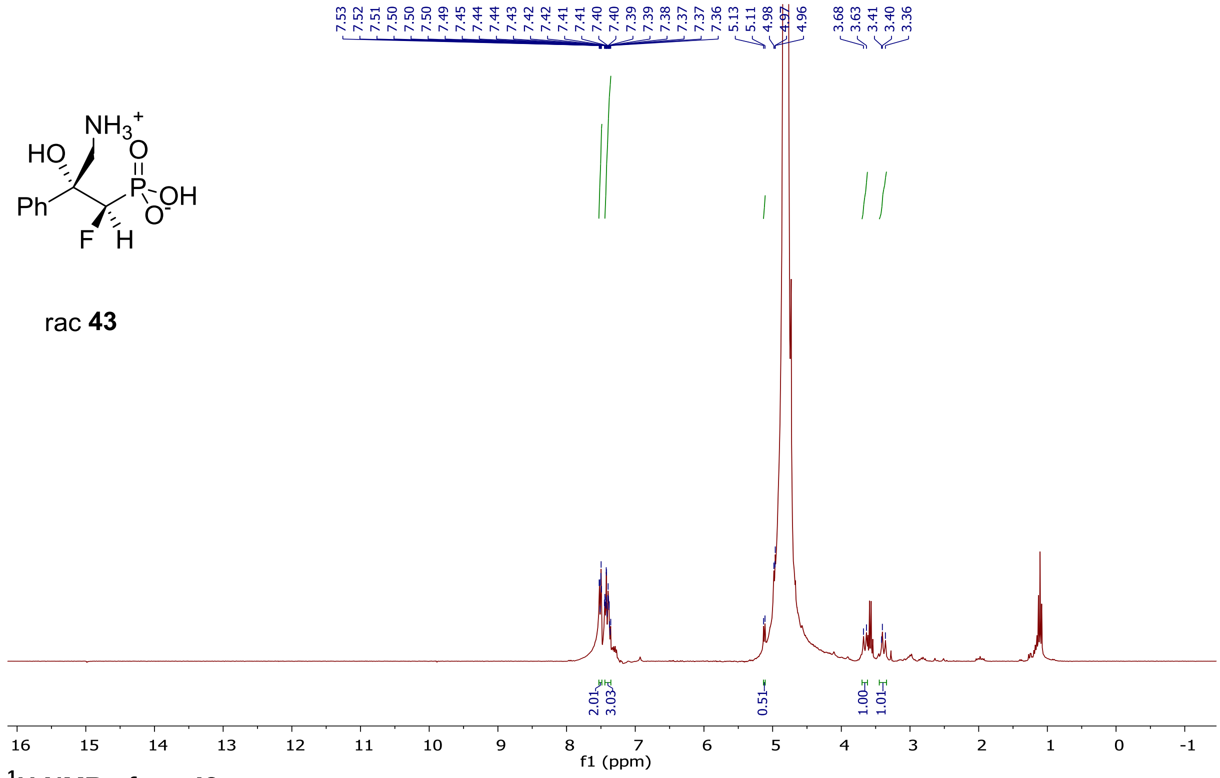

^{1}H NMR of rac **43**

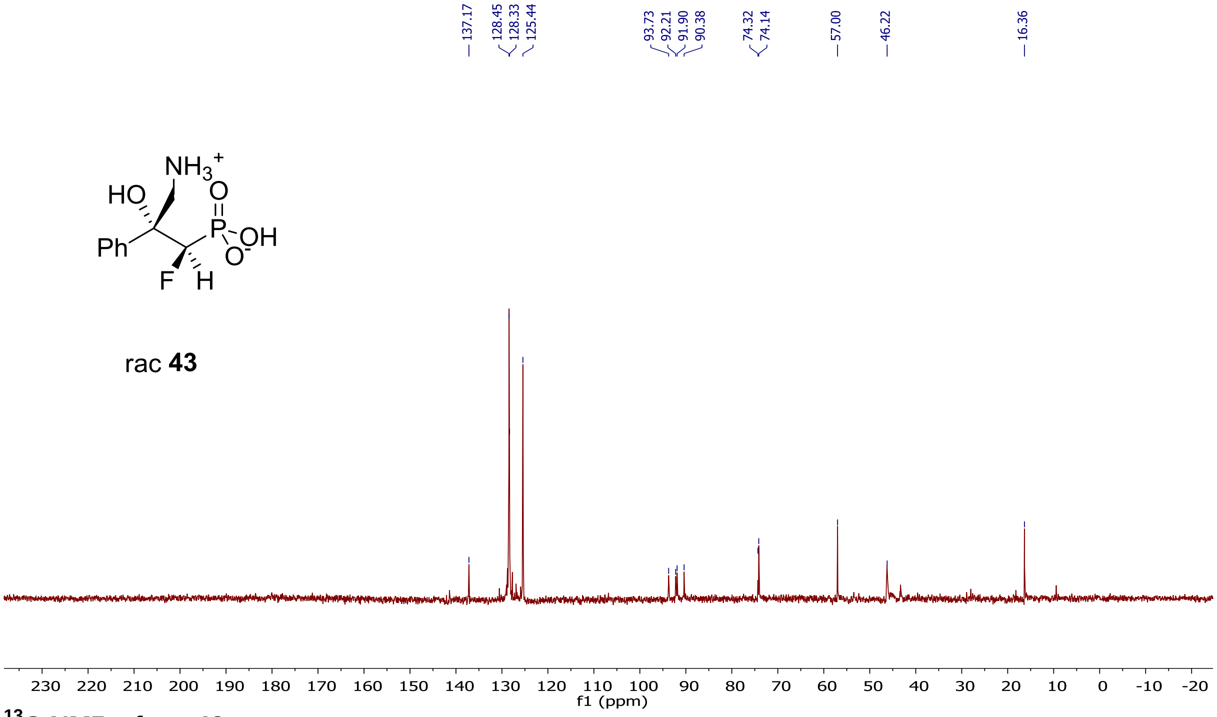

^{13}C NMR of rac **43**

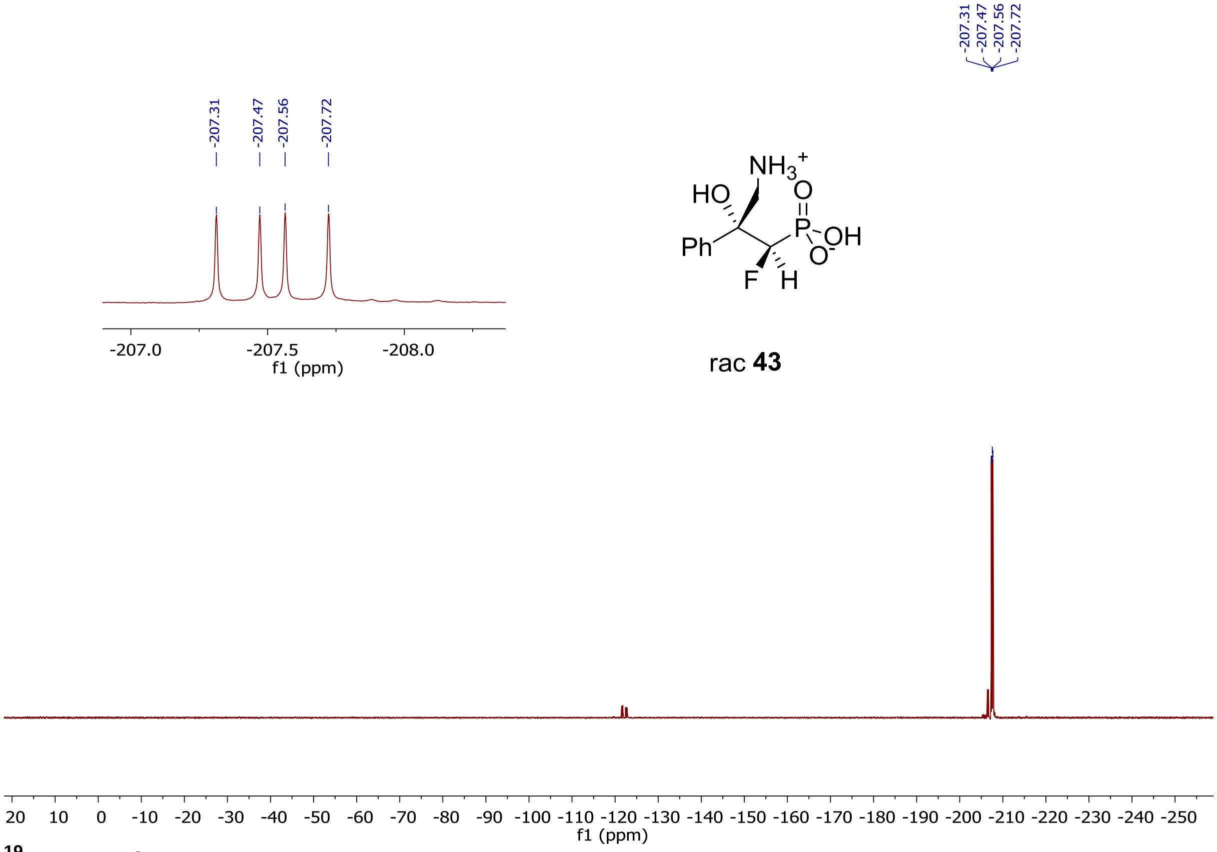

^{19}F NMR of rac **43**

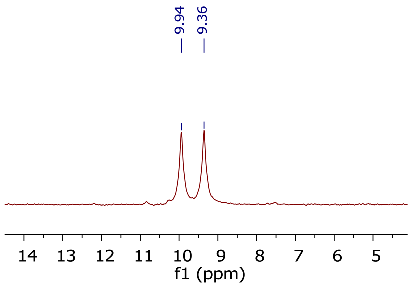

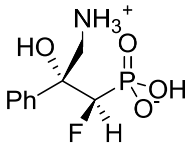

rac **43**

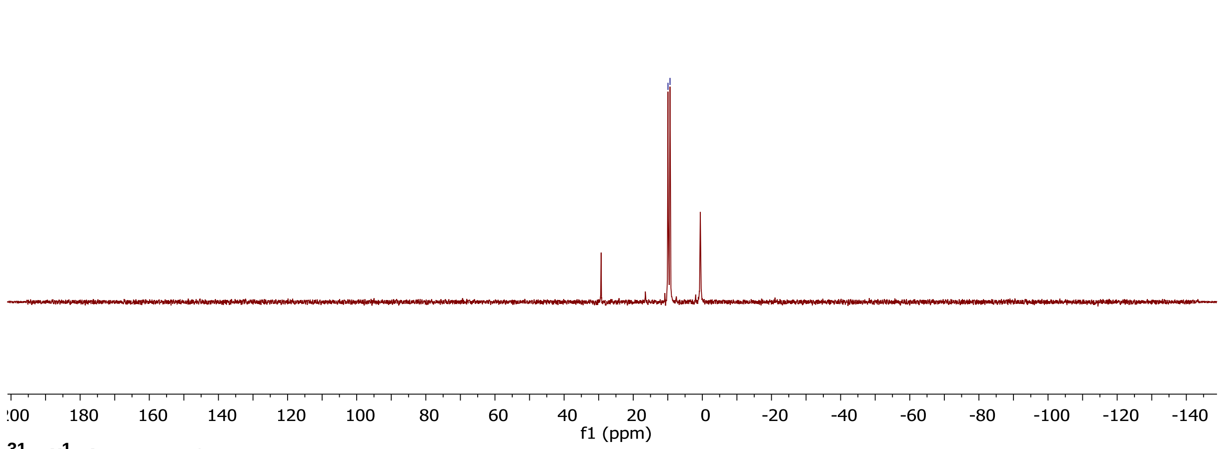

^{31}P{/^{1}H} NMR of rac **43**
